# A Draft Map of Rhesus Monkey Tissue Proteome for Biomedical Research

**DOI:** 10.1371/journal.pone.0126243

**Published:** 2015-05-14

**Authors:** Jin-Gyun Lee, Kimberly Q. McKinney, Yong-Yook Lee, Hae-Na Chung, Antonis J. Pavlopoulos, Kook Y. Jung, Woong-Ki Kim, Marcelo J. Kuroda, David K. Han, Sunil Hwang

**Affiliations:** 1 Proteomics Laboratory for Clinical and Translational Research, Carolinas HealthCare System, Charlotte, North Carolina, United States of America; 2 Department of Microbiology and Molecular Cell Biology, Eastern Virginia Medical School, Norfolk, Virginia, United States of America; 3 Tulane National Primate Research Center, Tulane University, Covington, Louisiana, United States of America; 4 Department of Cell Biology and Center for Vascular Biology, University of Connecticut Health Center, Farmington, Connecticut, United States of America; Nanjing Medical University, CHINA

## Abstract

Though the rhesus monkey is one of the most valuable non-human primate animal models for various human diseases because of its manageable size and genetic and proteomic similarities with humans, proteomic research using rhesus monkeys still remains challenging due to the lack of a complete protein sequence database and effective strategy. To investigate the most effective and high-throughput proteomic strategy, comparative data analysis was performed employing various protein databases and search engines. The UniProt databases of monkey, human, bovine, rat and mouse were used for the comparative analysis and also a universal database with all protein sequences from all available species was tested. At the same time, *de novo* sequencing was compared to the SEQUEST search algorithm to identify an optimal work flow for monkey proteomics. Employing the most effective strategy, proteomic profiling of monkey organs identified 3,481 proteins at 0.5% FDR from 9 male and 10 female tissues in an automated, high-throughput manner. Data are available via ProteomeXchange with identifier PXD001972. Based on the success of this alternative interpretation of MS data, the list of proteins identified from 12 organs of male and female subjects will benefit future rhesus monkey proteome research.

## Introduction

Since the human genome project was completed in 2003, proteomics has become a powerful tool for understanding the large and global characteristics of proteins within a broad range of biomedical research platforms [1–3]. To achieve automated peptide sequencing using tandem mass spectrometry (MS/MS), development of database search algorithms such as SEQUEST, Mascot and X!Tandem, have been geared toward the few species with well-annotated protein databases[4–7]. However, automated and high-throughput peptide sequencing with mass spectrometry analysis has been hindered for research in species with incomplete or uncertain database entries. The current approach to overcoming the challenge of the incomplete or uncertain protein databases is to include the use of redundant whole proteome databases from National Center for Biotechnology Information (NCBI) and/or Universal Protein Resource (UniProt). Usually, this approach requires an extensive amount of time and a high level of computational performance to deal with comparative MS data interpretation of over 3 million protein sequence entries. Alternatively, the *de novo* peptide sequencing strategy was introduced as a promising methodology for interpretation of LC-MS/MS data from unknown species. However, current software such as DeNoS [8], Lutefisk[9] and PEAKS[10] do not yet support a fully automated search function, so they eventually require much more time than automated database search engines.

The term “non-human primate” is used to describe a primate animal subject which possesses genetic similarities with humans. These primates are deemed the most appropriate animal models for use in human disease and physiology studies in fields such as aging, nutrition, neurodegenerative disease and human immunodeficiency virus (HIV) infection. A number of animal studies with rhesus monkeys have made remarkable progress in the field of clinical trials because of the high homology to the human genome. Recent studies of aging using rhesus monkey have suggested that caloric restriction considerably extends life span [11]. One pioneer study of transgenic non-human primate models for Huntington’s disease (HD) showed progress in developing a transgenic model of HD in the rhesus monkey[12], and others have demonstrated the use of *Macaca mulatta* in describing important clinical features of other diseases such as dystonia[13]. Most significantly, non-human primate models are essential in the research field of HIV because of HIV’s similarity of pathogenic characteristics to those of simian immunodeficiency virus (SIV) infection, which causes immune system dysfunction in the rhesus monkey. Among various monkey species, the rhesus monkey, *Macaca mulatta*, is the most commonly used animal model not only because of its genetic and proteomic similarities with human, but also due to size and manageability in the research facility.

Recent proteomic studies have presented the feasibility of global proteomic research in monkey models using whole proteome database searches and *de novo* sequencing strategies [14–22]. However, these approaches have limitations to overcome for automated and high-throughput processing in global proteomic investigations. In this study we utilized an alternative strategy employing human protein database searches for multi-organ proteome profiling of the rhesus monkey. Employing the SEQUEST algorithm for searching human protein databases, the identified proteins derived from 12 organs of male and female rhesus monkeys were integrated into a suggested prototype monkey proteome databank to be used as a resource for biomedical, animal model-based research.

## Materials and Methods

### Chemicals and reagents

All solvents for mass spectrometry analysis, 0.1% formic acid in water and 0.1% formic acid in ACN were of LC-MS grade purchased from EMD (Gibbstown, NJ, USA). Sequencing grade modified trypsin was from Promega (Madison, WI, USA) and Gelcode Blue stain reagent was from Pierce (Rockford, IL, USA). Complete protease inhibitor cocktail tablet was obtained from Roche (Mannheim, Germany). Ammonium bicarbonate, ammonium acetate, DTT, iodoacetamide, Tris-HCl, bromophenol blue, beta-mercaptoethanol, Tween 20, formic acid and SDS were obtained from Sigma-Aldrich (St. Louis, MO, USA). Glycerol was from Life Technologies (Gaithersburg, MD, USA). All buffers and solutions were prepared using deionized water by Milli-Q, Millipore (Bedford, MA, USA). Primary antibodies against human vimentin and heat shock protein-70 (HSP-70) were purchased from BD Biosciences (Franklin Lakes, NJ, USA); those against beta-actin and glyceraldehyde 3-phosphate dehydrogenase (GAPDH) were purchased from Santa Cruz (Santa Cruz, CA, USA). Primary antibody against human beta-catenin was from Cell Signaling (Danvers, MA, USA). Unless stated otherwise, all other chemicals were extra-pure grade or cell culture tested.

### Tissue collection from animal subjects

Tissues from twelve organs of rhesus monkeys were provided from Dr. Kuroda of Tulane University. Experimental procedures described for tissue collection were approved by the Tulane Institutional Animal Care and Use Committee (Protocol Number: P0162) and performed according to the ARRIVE guidelines (S1 Table). In detail, all animals were housed either outdoors or indoors prior to euthanasia at the Tulane National Primate Research Center (TNPRC), an Association for the Assessment and Accreditation of Laboratory Animal Care, International (AAALAC)-accredited facility, in accordance with standard husbandry practices following the Guide for the Care and Use of Laboratory Animals (NIH). Indoor animals were kept in temperature-controlled facilities with a 12:12 light:dark cycle. Animals were fed LabDiet Fiber-Plus Monkey Diet (LabDiet; St. Louis, MO). Additional feeding enrichment and forage items were given as part of a comprehensive environmental enrichment program that also uses social housing to promote species-typical behavior.

Animals were humanely euthanized by the veterinary staff at the TNPRC in accordance with endpoint policies. Euthanasia was conducted by anesthesia with ketamine HCl (10 mg/kg) followed by an overdose with sodium pentobarbital. This method is consistent with the recommendation of the Panel on Euthanasia of the American Veterinary Medical Association. Tissues were collected from subjects involved in other studies. Animals were euthanized as part of those studies and/or for humane reasons, such as in the case of injury or behavioral issues.

Nine tissues, namely, frontal cortex, cerebellum, right ventricle, mesenteric lymph node, proximal bile duct, liver, pancreas, prostate (apex) and penis were collected from a 6.54 year’s old male subject, and ten tissues, namely, frontal cortex, cerebellum, right ventricle, mesenteric lymph node, proximal bile duct, liver, pancreas, breast, ovary and clitoris were collected from a 10.55 year’s old female subject. Tissues were snap frozen in liquid nitrogen prior to be stored at −80°C.

### Preparation of Proteomic Samples

50 mg of frozen tissues were transferred into clean tubes with ice-cold PBS, then washed briefly by flicking tubes with one additional change of PBS. The tissues were homogenized mechanically in 1 mL of RIPA buffer containing 50 mM Tris-HCl, 150 mM NaCl, 1% NP-40, 0.5% sodium deoxycholate and 0.1% SDS (pH 8.0) with a protease inhibitor cocktail using a TH115 homogenizer (Omni International, Kennesaw, GA, USA). Crude lysates were centrifuged at 12,000 x g at 4°C for 15 min after incubation on an ice bath for 30 min. Then supernatants were transferred into clean tubes and put into -80°C for long-term storage. The protein quantitation was carried out using a BCA protein assay kit purchased from Thermo Pierce (Rockford, IL, USA).

Lysates were reduced and denatured by heating with 6X sample buffer containing 300 mM Tris-HCl, 0.01% bromophenol blue (w/v), 15% glycerol (v/v), 6% SDS (w/v) and 1% beta-mercaptoethanol (v/v). 30 μg of total proteins were separated on a 10% Bis-Tris SDS-PAGE gel (Invitrogen, Carlsbad, CA, USA). Gels were stained with GelCode blue stain reagent after fixation using 50% methanol (v/v) with 7% acetic acid (v/v) for 5 min. After destaining with water, each gel lane was excised into twenty slices, which were then chopped into 1-mm^3^ pieces. The gel pieces were de-stained with 50% ACN (v/v) and 25 mM ammonium bicarbonate at room temperature for 30 min three times. Once Coomassie stain was removed, gel pieces were dehydrated using 100% ACN at room temperature for 30 min, then dried in a Centrivap (Labconco, Kansas City, MO, USA). The gel pieces were re-hydrated and incubated at 37°C overnight in 50 mM ammonium bicarbonate containing 12.5 ng/mL of trypsin. Peptides from the gel pieces were extracted by the addition of 50% ACN (v/v) with 5% formic acid (v/v) 3 times. Extract was vacuum-dried in the Centrivap and residues were resuspended in 20 μL of 5% ACN (v/v) with 3% formic acid (v/v) for LC-MS/MS analysis.

### Liquid chromatography and tandem mass spectrometry

The LC-MS/MS system used for comprehensive tissue proteomics consisted of an LTQ-XL mass spectrometer (Thermo Scientific, Rockford, IL, USA) employing a nanoscale electrospray ionization source (PicoView, New Objective, Woburn, MA, USA) in combination with ACQUITY UPLC system (Waters, Milford, MA, USA). An in-house made trap column (0.15 x 30 mm) and analytical column with needle tip (0.1 mm x 100 mm) were employed for peptide separation. Magic C_18_ (100 Å, 5 μm, New Objective, Woburn, MA, USA) was used for the stationary phase, which was packed into a fused silica capillary using high pressure nitrogen gas. A customized double-split system was used to achieve nanoliter per minute flow rates. Good chromatographic separation was observed with a 65 minute linear gradient consisting of mobile phases solvent A (0.1% formic acid in water) and solvent B (0.1% formic acid in ACN) where the gradient was 0min, 5%B ➔ 65min, 40%B). MS spectra were acquired by data dependent scans consisting of MS/MS scans of the eight most intense ions from the full MS scan with dynamic exclusion of 60 seconds. The mass spectrometry proteomics data have been deposited to the ProteomeXchange Consortium [23] via the PRIDE partner repository with the dataset identifier PXD001972.

Also, a high-resolution LC-MS/MS (LTQ/Orbitrap-XL mass spectrometer, Thermo Scientific, Rockford, IL, USA) equipped with Nanoacquity UPLC system (Waters, Milford, MA, USA) was used for the experiments which included *de novo* sequencing. Peptides were separated on a reversed phase analytical column (Nanoacquity BEH C_18_, 1.7μm, 150mm, Waters, Milford, MA, USA) combined with trap column (Nanoacquity, Waters, Milford, MA, USA). Good chromatographic separation was observed with a 75 min linear gradient consisting of mobile phases solvent A (0.1% formic acid in water) and solvent B (0.1% formic acid in ACN) where the gradient was from 5% B at 0 min to 40% B at 65 min. MS spectra were acquired by data dependent scans consisting of MS/MS scans of the eight most intense ions from the full MS scan with dynamic exclusion of 30 seconds.

### Proteomics data analysis

Spectra were searched using the SEQUEST search algorithm within Proteome Discoverer v1.4 (Thermo Scientific, Rockford, IL, USA) using the annotated UniProt FASTA database of human (20,162 entries), mouse (17,123 entries), rat (8,016 entries) and bovine (6,859 entries). In addition, UniProt database of total species (547,357 entries with annotation for the whole species) and UniProt database of canonical and isoform sequences (TrEMBL) for *Macaca mulatta* (71,058 entries) were used for comparison. Also, the annotated *Macaca mulatta* database (358 entries) was employed for the generation of a peptide list for further data analysis. Search parameters for LTQ-XL were as follows: parent mass tolerance of 2.0 Da, fragment mass tolerance of 1.0 Da (monoisotopic), variable modification on methionine of 16 Da (oxidation) and maximum missed cleavage of 2 sites using the digestion enzyme trypsin. 0.5 Da of parent mass tolerance and 10 ppm of fragment mass tolerance were used for FT-MS. Search results were entered into Scaffold software (v4.0.1, Proteome Software, Portland, OR) for compilation, normalization, and comparison of spectral counts. Protein identifications were made at the 95% of peptide probability and 99% of protein probability of with at minimum one identified peptides. Shared and semi-tryptic peptides were excluded from spectral counts. Protein probability and redundancy were assigned by the Protein Prophet algorithm[24]. Proteins that contained similar peptides and multiple isoforms, which could not be differentiated based on MS/MS spectra, were grouped into primarily assigned proteins. The intersection of datasets were acquired using a Microsoft Excel program with modified macro equations.

Alternatively, *de novo* sequencing was tested for data search and was performed using PEAKS Studio v.5.1 (Bioinformatics Solutions, Ontario, Canada) with the following parameters: FT-trap instrument, parent mass error tolerance of 10.0 ppm, fragment mass error tolerance of 0.5 Da (monoisotopic), trypsin enzyme, variable modification on methionine of 16 Da (oxidation) and maximum missed cleavage of 2 sites assuming the digestion enzyme trypsin.

For gene ontology analysis, compiled datasets from each tissue sample including protein name, accession number and spectral count were put into pathway analysis by uploading them into Ingenuity Pathway Analysis (IPA) v9.0 (Ingenuity Systems, Mountain View, CA, USA). Analysis settings included the reference set of the Ingenuity Knowledge Base (genes only). Ingenuity Pathway Analysis is an algorithm enabling network assembly and interrogation of the scientific literature that documents direct and indirect relationships between genes. Identified proteins were analyzed in order to obtain the most significant biological functions and physiological functions.

### Western blot analysis

Proteomic datasets from computational data analysis were validated by western blot assay. In brief, 20 μg of protein with sample buffer was loaded onto a Bolt 4 ~ 12% Bis-Tris Plus (Invitrogen, Carlsbad, CA, USA) gel and separated at 165 V for 35 min. Then proteins were transferred from the gel to 0.45 μm nitrocellulose membrane (BioRad, Hercules, CA, USA) using the Xcell-II blot module (Invitrogen, Carlsbad, CA, USA) at 25 V for 2 hr. The membrane was blocked for one hour at room temperature with blocking buffer, 5% dried non-fat milk (BioRad, Hercules, CA. USA) dissolved in Tris-buffered saline containing 0.1% Tween-20 (TBST). Membranes were incubated with primary antibody at 1:1,000 ~ 1:5,000 dilutions in blocking buffer overnight at 4°C. Blots were washed with TBST for 15 min 3 times. Membranes were incubated in the appropriate secondary antibody conjugated to HRP for 1 hr at room temperature. Blots were washed with TBST 3 times for 15 min. Chemiluminescent detection was accomplished using Amersham ECL prime western blotting detection reagent (GE Healthcare, Fairfield, CT, USA) and the UVP Biospectrum 500 Imaging System (Upland, CA, USA).

### Immunohistochemistry

Tissue sections were prepared from formalin-fixed, paraffin-embedded tissue blocks by deparaffinization with xylene and hydration using alcohol and deionized water. The rest of the procedure was performed using the Autostainer Plus. After blocking of endogenous peroxidase with 3% (v/v) hydrogen peroxide, tissue slides were incubated with primary antibody at the dilution of 1:100. Secondary antibody was followed by peroxidase-conjugated streptavidin for 10 min and 3’-diaminobenzidine for 5 min. Tissue slides were rinsed with water and counterstained with hematoxylin, dehydrated, cleared and mounted with resin matrix. Slides were visualized using an Olympus BX51 microscope equipped with an Olympus DP70 camera and DP controller imaging software (Olympus Corporation, Tokyo, Japan).

## Results and Discussion

### Comparison of database search methods

**Fig 1:**
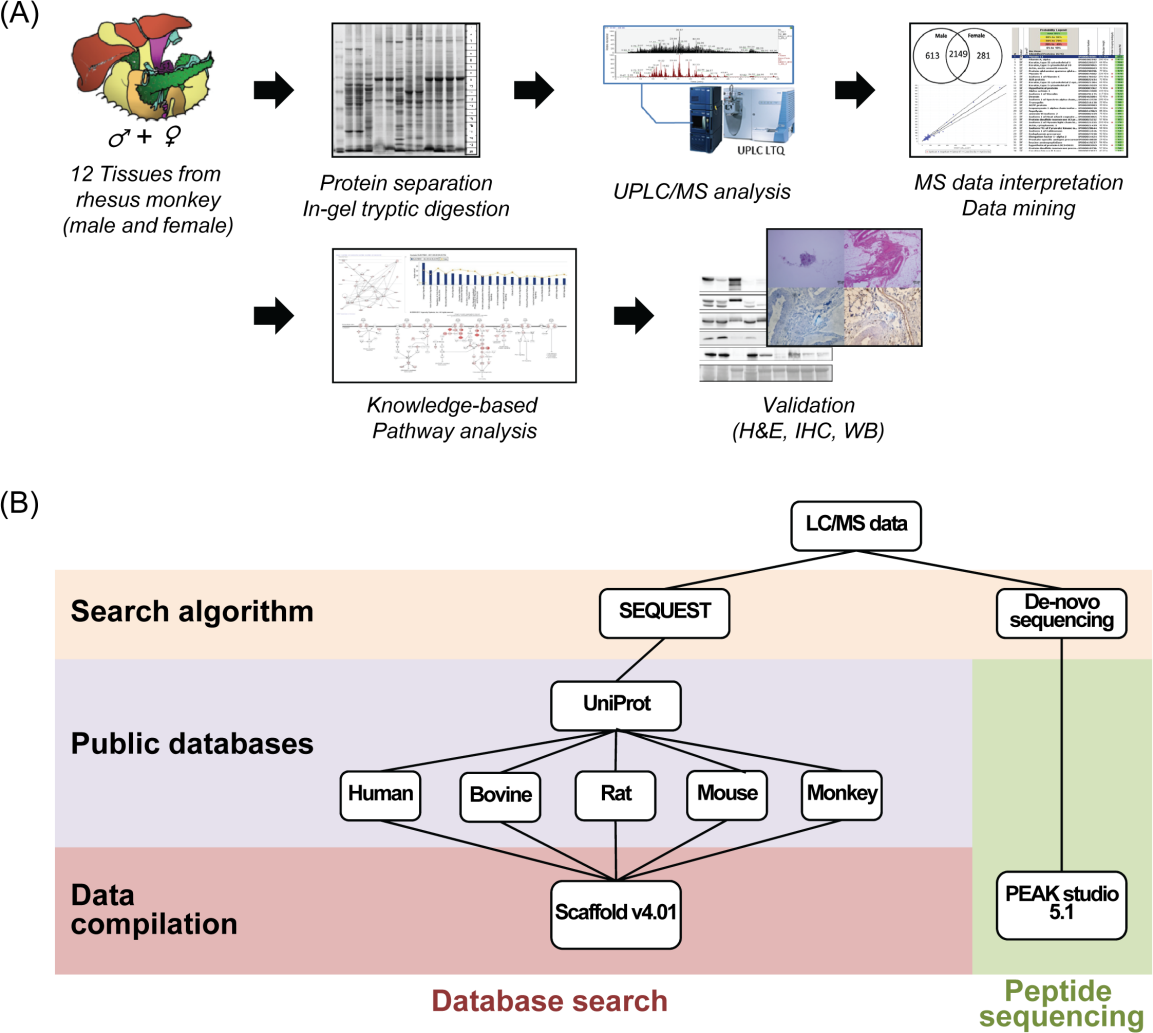
Summary of proteomic analysis. (A) Schematic procedure of large-scale monkey proteome research, (B) Work flow for the interpretation of MS data of monkey tissues using various databases.

MS spectra acquired from instrumental analysis were imported into data search algorithms with various public databases to achieve comparative protein identification. The annotated protein FASTA databases (Swiss-Prot) of four mammal species including human from UniProt were compared to establish the most effective and alternative data processing work flow. (Fig 1)

**Fig 2:**
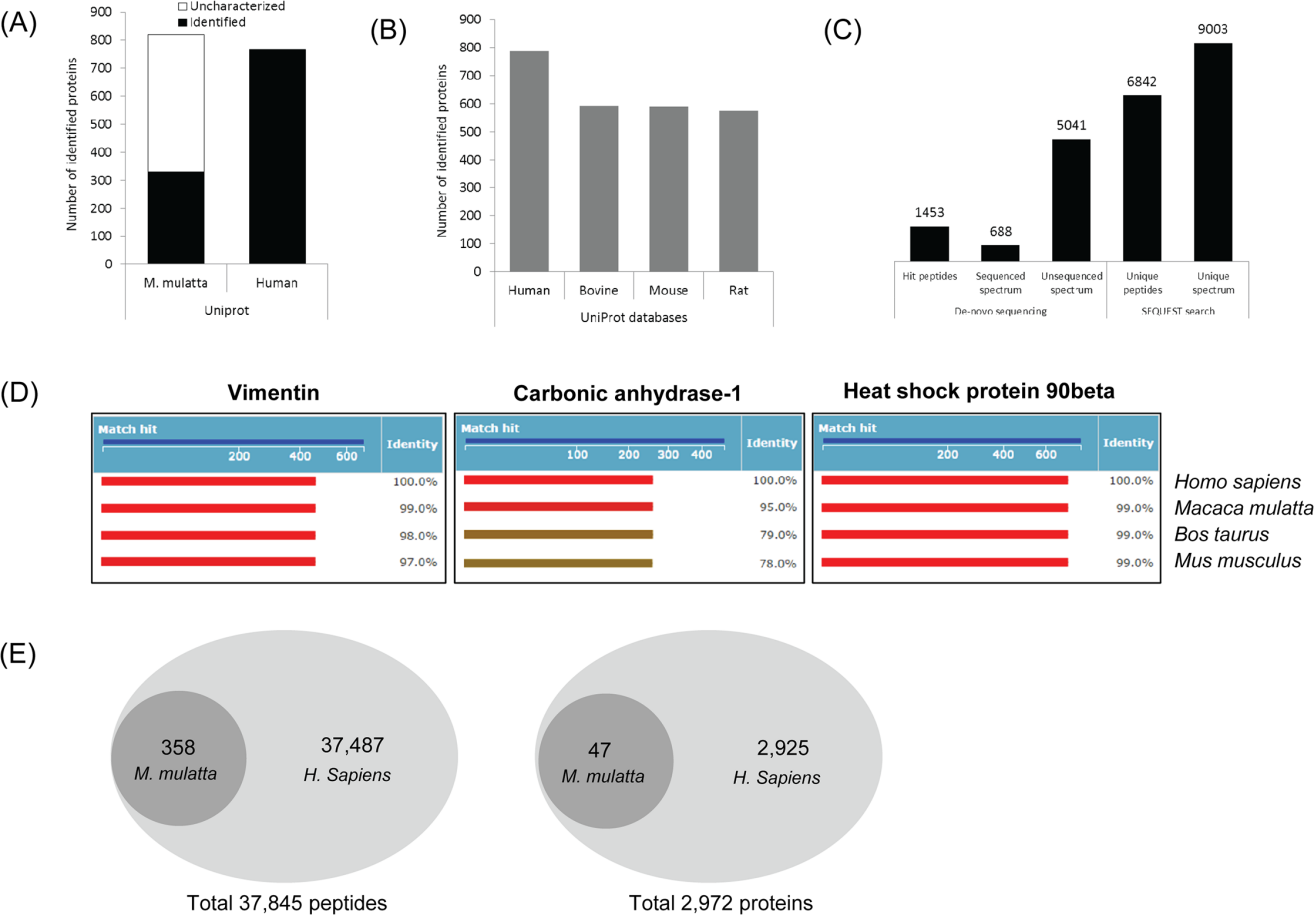
Evaluation of analytical strategies for monkey proteomics. (A) Comparative graph showing the number of identified proteins acquired from SEQUEST search employing UniProt databases of *Macaca mulatta* and *Homo sapiens* (B) Database comparison for employing the databases of four mammals, human, bovine, mouse and rat (C) Comparison of *de novo* sequencing and SEQUEST algorithm. The SEQUEST search of the UniProt human database provided a higher yield of peptides compared to PEAKS (D) Graphs exhibiting homology (%) of representative proteins identified from four mammals. (E) The total number of identified peptides and proteins from 9 organs of the male subject (EL30) by database search using UniProt (Swiss-Prot) databases of *Macaca mulatta* and *Homo sapiens*.

A complete set of MS raw data files of the male liver tissue was used for comparison of the protein numbers identified. The UniProt database of human and *Macaca mulatta* were tested using the SEQUEST search algorithm. (Fig 2A) Since the annotated FASTA database of *Macaca mulatta* has only 358 entries, TrEMBL database was used for monkey. Although the TrEMBL UniProt database of *Macaca mulatta* contains over 70,000 entries, the SEQUEST search with the UniProt monkey database returned matches to 819 proteins, of which 488 were “uncharacterized proteins” due to the fact that most of the entries have not yet been annotated. S2 Table presents the top 20 proteins identified from the search with the tested UniProt databases and demonstrates that most of the listed proteins are indicating the same proteins. Also top proteins from International Protein Index (IPI) human database (v3.72) have been presented for comparison.

Among the tested databases, the integrated UniProt database containing protein sequences of all available species would represent an alternative for the deficient monkey protein database. However, it requires a tremendous amount of time for the processing of the MS data files, which is not practical compared to the other databases tested (Data not shown). The SEQUEST search using the NCBI human database, IPI human database and NCBI *Macaca mulatta* database were revealed to be time effective for monkey proteomics, however, the NCBI *Macaca mulatta* database has a barrier due to its limited protein sequence entries, similar to the *Macaca mulatta* UniProt database, thus NCBI databases were excluded from the evaluation. Also, the UniProt databases of three non-human mammals (bovine, mouse and rat) were tested comparatively. As shown in Fig 2B, the human database provided the largest number of protein identifications compared to the other databases. The human database identified 786 proteins from male liver tissue, while bovine, mouse and rat databases only identified 593, 590 and 574 proteins, respectively. Though the human database provided the largest number of proteins, the databases from the three mammals still covered around 70% of the identified proteins. To evaluate the protein sequence homology, alignment analysis was performed using representative proteins, vimentin, carbonic anhydrase-1 and heat shock protein 90-beta. Human protein sequences were compared to the corresponding sequences from other species directly by alignment (http://www.uniprot.org/align), which demonstrates that common proteins identified from four mammals, human, monkey, bovine and mouse, showed high homology of their amino acid sequences (Fig 2D). Especially, among other species, human exhibited the highest homology to a large portion of the entire rhesus monkey proteome.

The proteomic data from the human database search was subjected to further comparison to the data from the UniProt *Macaca mulatta* (annotated, 358 entries) database at the peptide level. In total 307 peptides (47 proteins) were identified from the male subject (9 organs from EL30) employing the *Macaca mulatta* database search, while a total of 37,845 peptides (2,972 proteins) were identified from the human database search. The Venn-diagram in Fig 2E presents the intersection of common and unique peptide numbers. It was revealed that all of the peptides identified from the *Macaca mulatta* database were completely included in the peptides listed from the human database. It is a reasonable result judging from the high homology of protein sequences presented in Fig 2D and the small numbers of total entries in the *Macaca mulatta* database.

Additionally, MS raw data files were processed for *de novo* sequencing using PEAKS Studio v5.1 software for comparison with the SEQUEST algorithm. The *de novo* peptide sequencing approach was introduced as a promising methodology for the interpretation of LC-MS/MS data from species for which only partial or no protein databases are available. This approach has recognized limitations due to the lack of software which support a fully automated search. Fig 2C is showing comparative peptides and numbers of hits generated by de novo sequencing software (PEAKS studio v5.1) *versus* SEQUEST search engine identifications. The SEQUEST algorithm generated 6,842 sequenced peptides, which was 4.7 fold higher than the number of peptide hits from the PEAKS software. Judging by the results shown in Fig 2, the SEQUEST algorithm utilizing the UniProt human database would be an acceptable and effective alternative for the proteomic profiling and analysis of rhesus monkey tissues.

### A draft tissue proteome map of female and male rhesus monkey

Comprehensive and integrated proteomic analysis employing human databases (UniProt, Swiss-Prot) was performed with the tissues from twelve organs of one male and one female rhesus monkey. The SEQUEST search employing the UniProt human FASTA protein database identified a total of 3,481 proteins at 0.5% of false discovery rate (FDR) from nine tissues (frontal cortex, cerebellum, right ventricle, mesenteric lymph node, pancreas, liver, proximal bile duct, prostate (apex) and penis) from a male subject and ten tissues (frontal cortex, cerebellum, right ventricle, mesenteric lymph node, pancreas, liver, proximal bile duct, breast, ovary and clitoris) from a female subject.

Since the whole monkey tissue proteome datasets were acquired from an early generation (LTQ-XL, Thermo Scientific) mass spectrometer, the number of protein identifications may seem to lower than expected. However, most of the proteins identified employing this well-established methodology have been observed to be confident and reliable. To investigate the effect of instrumentation on the variation of protein numbers and the quality of data, male prostate and pancreas samples were analyzed with a high-resolution ion trap mass spectrometer (LTQ/Orbitrap-XL, Thermo Scientific) in combination with nano-UPLC (NanoAcquity equipped with a reversed-phase capillary column, 250mm x 75μm, Waters).

S1 Fig presents the results from the comparative analysis of monkey prostate and pancreas using LTQ-XL (LTQ) and LTQ/Orbitrap-XL. Filter criteria were 95% peptide probabilities including single peptide hits. Orbitrap analysis identified 1,960 proteins from pancreas and 1,868 proteins from prostate, both at false discovery rates of 0.5%. This represents an increase of 95% and 70%, respectively (S1A Fig). In order to assess the quality of those proteomic datasets, the newly identified proteins acquired by the Orbitrap runs were subjected to further analysis using Scaffold v4.01. As a result, more than 80% of those unique proteins identified by Orbitrap were revealed to have lower scoring matches, for which spectral counts were less than 5 for both tissues, and most of those lower scoring proteins have lower protein sequence coverage (less than 10%) (S1B Fig). Of course portions of those proteins with lower scores are still valuable since they have reasonable protein probabilities and good quality of observed peptide sequences. But, in terms of the basic information about monkey organ tissues, the presented proteome datasets in the original data are still useful as a draft proteome map of multiple monkey organs since the datasets were obtained from an optimized and consistent analytical system with adequate quality controls between biological samples, and since the datasets exhibit characteristic expression profiles of monkey organ proteins—although they have smaller numbers of identified proteins.

**Fig 3:**
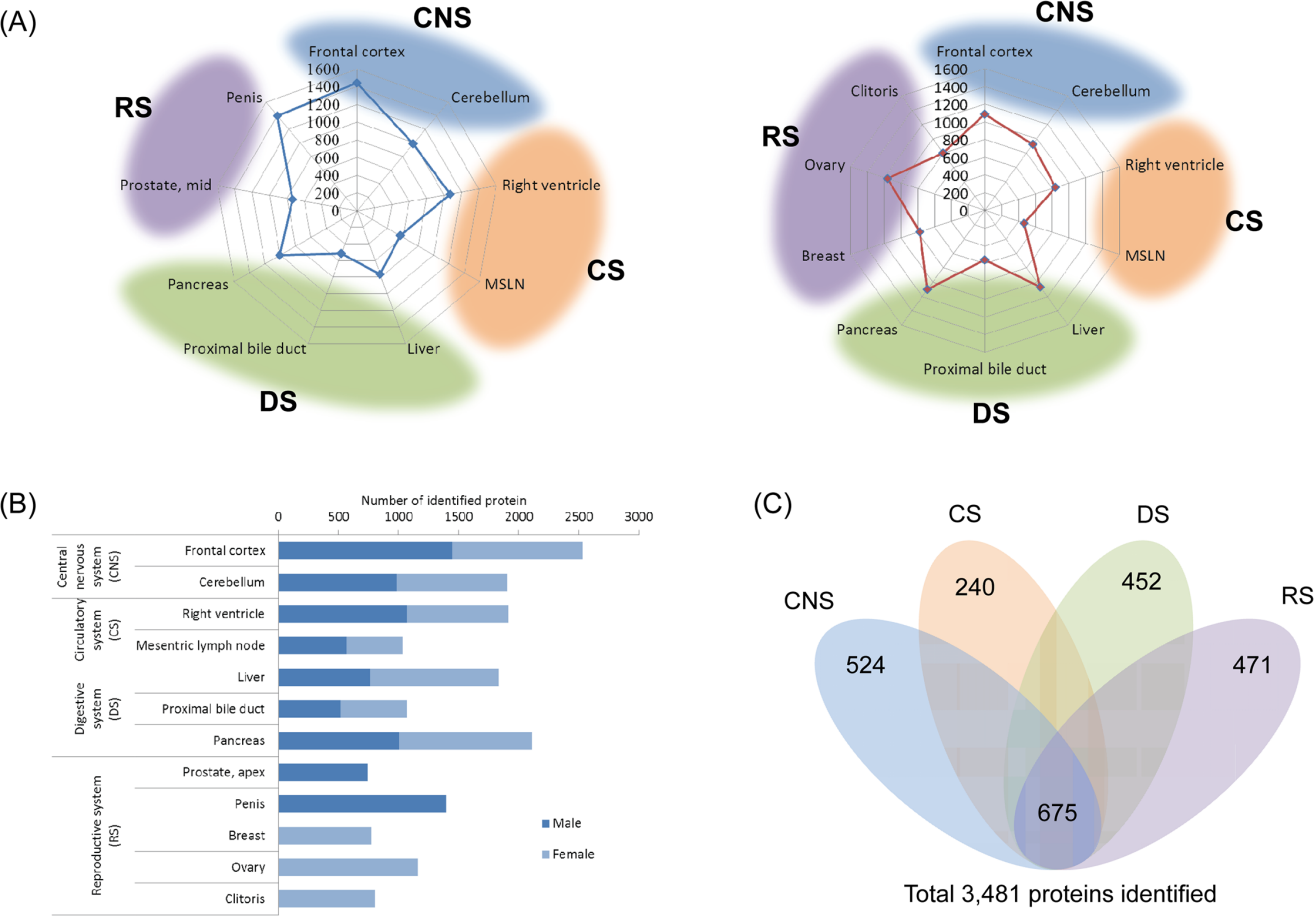
The comprehensive results monkey multi-organ proteomics. (A) Radar charts presenting the number of identified proteins from the tissues of rhesus monkey organs. Nine organ tissues were from the male subject (left) and ten organ tissues were from the female subject. A total of twelve tissues were clustered by their physiological function to give four groups, central nervous system (CNS), circulatory system (CS), digestive system (DS) and reproductive system (RS). (B) Bar chart comparing the number of identified proteins from male and female with a similar number of protein identifications overall. (C) SEQUEST search with annotated human UniProt database generated a total of 3,481 identified proteins from thirteen tissues of rhesus monkey. Intersect is showing common and unique proteins between four functional clusters.

The intersection of the lists of identified proteins from the individual organs generated by Scaffold software provided the top three most unique proteins from each tissue (Table 1) and the top thirty proteins identified commonly from all of the tissues (Table 2). Recently, it has been reported that 4,842 proteins were identified from 48 human tissues and 45 human cell lines employing tissue microarrays and immunohistochemical staining [25]. This study also provided a lists of tissue specific and cell type specific proteins. Surprisingly, a very low fraction (less than 2%) of proteins were reportedly expressed in a single or only few distinct types of cells, while the percentage of unique proteins was more than 34% in our current monkey multi-organ proteomics research. The difference in analytical approach is likely the main reason for such dramatic differences in the unique protein identification profiles. The human tissue and cell line article begins with a finite number of protein identifications possible (4,842), as the antibodies applied are a limiting factor for the total number of possible identifications. Using a global proteomics approach, which we present here, the limitation of possible identifications is dependent upon the number of entries in the database used to search the MS/MS spectra, which in this case was 20,162. The unique proteins identified from the current proteomics strategy correlated well with the characteristic function of each organ and their physiological roles, which is also supported by the knowledge-based pathway analysis. The lists of total identified proteins are available in supporting informations (S3 Table and S4 Table). For further data analysis, datasets from several organs were clustered by their physiological function; frontal cortex and cerebellum as central nervous system (CNS), right ventricle, mesenteric lymph node as circulatory system (CS), liver, proximal bile duct and pancreas as digestive system (DS), penis, prostate, clitoris, ovary and breast as reproductive system (RS), respectively. Fig 3A shows the number of protein identifications from each tissue in radar charts. We identified a similar number of proteins from female versus male tissues (Fig 3B), among which 675 proteins were common and 524, 240, 452 and 471 proteins were unique to CNS, CS, DS and RS, respectively (Fig 3C).

**Table 1:** Top 3 unique proteins identified from each tissue.

Organs	Description^1^	Accession	MW	Raw spectral counts
*Frontal cortex*	Serine/threonine-protein phosphatase 2B catalytic subunit beta isoform	PP2BB_HUMAN	59 kDa	29
	CaM kinase-like vesicle-associated protein	CAMKV_HUMAN	54 kDa	25
	Guanine deaminase	GUAD_HUMAN	51 kDa	22
*Cerebellum*	Excitatory amino acid transporter 4	EAA4_HUMAN	62 kDa	43
	Carbonic anhydrase-related protein	CAH8_HUMAN	33 kDa	17
	Probable ATP-dependent RNA helicase DDX5	DDX5_HUMAN	69 kDa	6
*Right ventricle*	Myosin-binding protein C, cardiac-type	MYPC3_HUMAN	141 kDa	28
	Myosin-1	MYH1_HUMAN	223 kDa	227
	Myosin-3	MYH3_HUMAN	224 kDa	18
*Mesentric lymph node*	Acetyl-CoA carboxylase 1	ACACA_HUMAN	266 kDa	6
	Immunoglobulin lambda-like polypeptide 1	IGLL1_HUMAN	23 kDa	5
	Aspartyl/asparaginyl beta-hydroxylase	ASPH_HUMAN	86 kDa	3
*Liver*	Carbamoyl-phosphate synthase [ammonia], mitochondrial	CPSM_HUMAN	165 kDa	992
	Alcohol dehydrogenase 4	ADH4_HUMAN	4 kDa	148
	Betaine—homocysteine S-methyltransferase 1	BHMT1_HUMAN	45 kDa	112
*Pancreas*	Alpha-amylase 1	AMY1_HUMAN	58 kDa	264
	Carboxypeptidase A1	CBPA1_HUMAN	47 kDa	218
	Pancreatic triacylglycerol lipase	LIPP_HUMAN	51 kDa	142
*Proximal bile duct*	Ras-related protein Rap-1A	RAP1A_HUMAN	21 kDa	8
	Galectin-4	LEG4_HUMAN	36 kDa	7
	Galectin-2	LEG2_HUMAN	15 kDa	3
*Prostate*	Prostatic acid phosphatase	PPAP_HUMAN	45 kDa	56
	Protein-glutamine gamma-glutamyltransferase 4	TGM4_HUMAN	77 kDa	56
	Prostate-specific antigen	KLK3_HUMAN	29 kDa	29
*Penis*	Envoplakin	EVPL_HUMAN	232 kDa	2
	Serine protease inhibitor Kazal-type 5	ISK5_HUMAN	121 kDa	18
	Filaggrin	FILA_HUMAN	435 kDa	16
*Breast*	Beta-casein	CASB_HUMAN	25 kDa	1
	Lactadherin	MFGM_HUMAN	43 kDa	9
	Signal transducer and activator of transcription 5B	STA5B_HUMAN	9 kDa	3
*Ovary*	LIM and cysteine-rich domains protein 1	LMCD1_HUMAN	41 kDa	16
	Zona pellucida sperm-binding protein 4	ZP4_HUMAN	59 kDa	7
	Core histone macro-H2A.2	H2AW_HUMAN	4 kDa	6
*Clitoris*	Transcription termination factor 3, mitochondrial	MTEF3_HUMAN	48 kDa	2
	Myosin light chain 1/3, skeletal muscle isoform	MYL1_HUMAN	21 kDa	2
	L-lactate dehydrogenase C chain	LDHC_HUMAN	36 kDa	2

^1^Keratins were excluded from the list.

**Table 2:** Top 30 common proteins identified from all of the tissue samples.

Description	Accession	MW	Raw spectral counts											
			Frontal cortex	Cerebellum	Right ventricle	Mesentric lymph node	Liver	Pancreas	Proximal bile duct	Prostate	Penis	Breast	Ovary	Clitoris
Serum albumin	ALBU_HUMAN	69 kDa	33	54	172	489	103	101	662	103	106	155	149	241
Actin, cytoplasmic 1	ACTB_HUMAN	42 kDa	137	253	164	362	114	165	509	149	86	67	167	150
Actin, alpha cardiac muscle 1	ACTC_HUMAN	42 kDa	94	183	229	387	80	116	548	175	68	63	145	145
Spectrin alpha chain	SPTN1_HUMAN	285 kDa	423	555	84	93	88	155	93	55	106	48	72	98
Filamin-A	FLNA_HUMAN	281 kDa	6	3	17	303	6	41	567	315	91	78	111	140
Tubulin beta chain	TBB5_HUMAN	50 kDa	284	435	45	134	53	104	239	23	67	63	91	106
Vimentin	VIME_HUMAN	54 kDa	55	70	43	420	18	32	362	37	140	82	188	185
ATP synthase subunit beta	ATPB_HUMAN	57 kDa	146	281	420	93	140	208	119	39	35	65	41	44
Tubulin alpha-1B chain	TBA1B_HUMAN	50 kDa	410	392	37	124	55	126	168	28	45	57	54	53
Tubulin alpha-1A chain	TBA1A_HUMAN	50 kDa	382	392	25	116	31	83	174	27	43	49	48	52
Tubulin beta-2B chain	TBB2B_HUMAN	50 kDa	277	385	32	104	41	84	193	20	46	44	60	77
Tubulin beta-4A chain	TBB4A_HUMAN	50 kDa	264	509	27	93	32	65	163	13	46	33	51	67
Hemoglobin subunit beta	HBB_HUMAN	16 kDa	46	42	67	317	103	20	319	13	55	35	37	51
Hemoglobin subunit alpha	HBA_HUMAN	15 kDa	49	35	110	230	155	27	265	9	75	41	37	44
Spectrin beta chain	SPTB2_HUMAN	275 kDa	242	342	20	71	65	88	71	4	28	33	46	58
Clathrin heavy chain 1	CLH1_HUMAN	192 kDa	369	191	48	35	51	132	35	49	53	50	17	33
Annexin A2	ANXA2_HUMAN	39 kDa	15	8	45	186	16	69	293	44	99	46	117	121
Hemoglobin subunit delta	HBD_HUMAN	16 kDa	40	32	60	297	105	20	280	12	47	33	26	49
ATP synthase subunit alpha	ATPA_HUMAN	60 kDa	94	162	307	42	61	121	47	48	26	29	20	23
Putative elongation factor 1α-like 3	EF1A3_HUMAN	50 kDa	34	31	26	88	120	218	85	55	83	48	26	55
Heat shock cognate 71 kDa protein	HSP7C_HUMAN	71 kDa	90	140	59	68	77	79	41	33	44	23	49	30
78 kDa glucose-regulated protein	GRP78_HUMAN	72 kDa	37	32	32	40	106	294	19	59	26	20	34	15
Alpha-enolase	ENOA_HUMAN	47 kDa	63	153	33	55	49	61	63	25	61	38	34	48
POTE ankyrin domain family	POTEI_HUMAN	121 kDa	54	67	29	128	34	30	181	31	22	22	39	45
Myosin-9	MYH9_HUMAN	227 kDa	32	6	7	45	70	177	26	3	97	3	79	120
Heat shock protein HSP 90-beta	HS90B_HUMAN	83 kDa	61	88	44	54	67	109	54	33	33	34	51	22
Malate dehydrogenase	MDHM_HUMAN	36 kDa	77	117	115	76	44	37	62	25	18	30	25	23
Alpha-actinin-1	ACTN1_HUMAN	103 kDa	69	15	55	67	20	60	120	84	31	44	34	50
GAPDH	G3P_HUMAN	36 kDa	75	164	59	45	60	26	64	27	31	15	35	42
Vinculin	VINC_HUMAN	124 kDa	7	1	74	140	25	67	125	63	30	22	35	47

**Fig 4:**
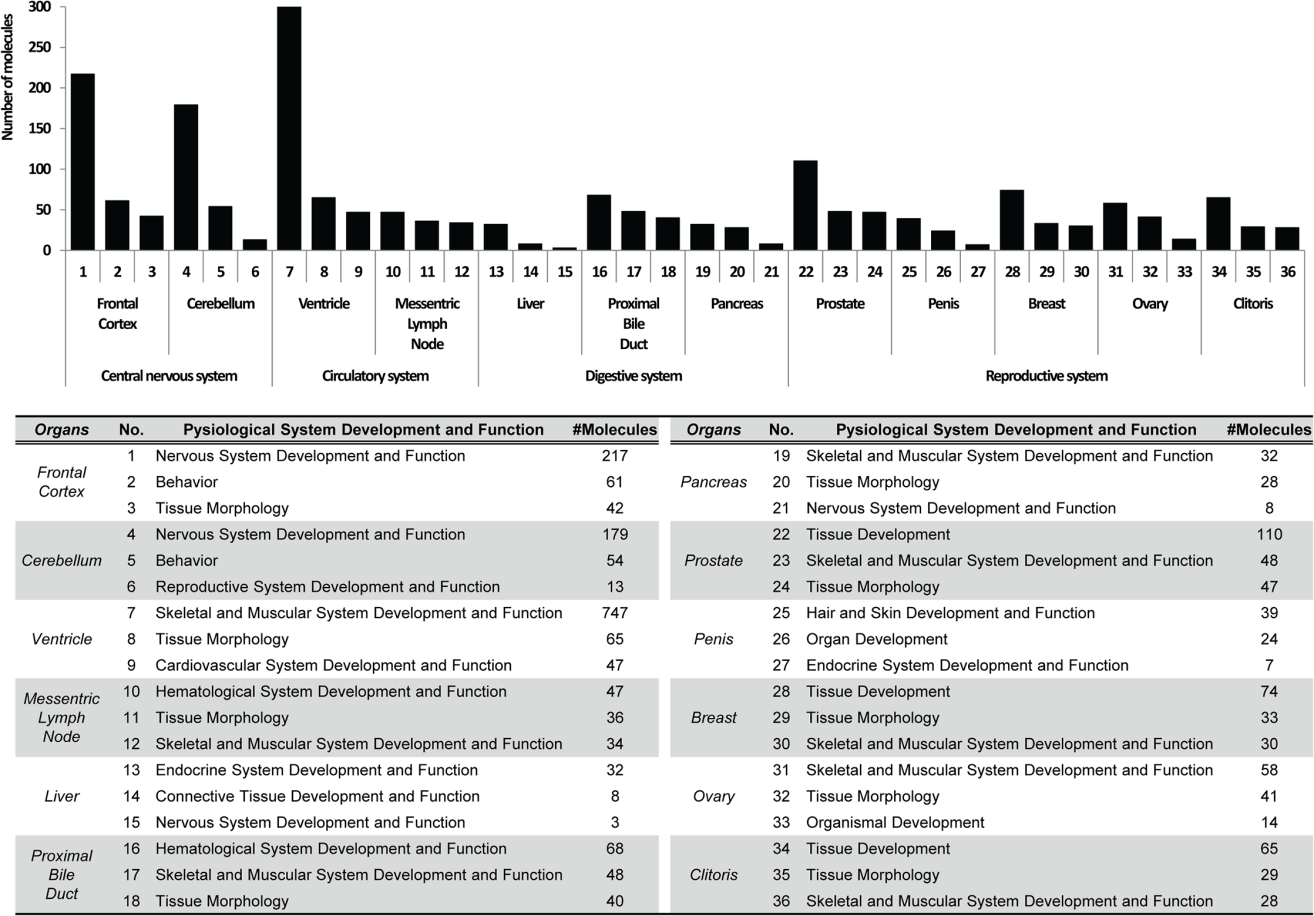
Top significant physiological functions of monkey tissues. Bar charts showing the most significant physiological function of each tissue provided by Ingenuity Pathway Analysis (IPA). The lists of identified proteins from twelve tissues were subjected to pathway analysis. Gene ontology analysis was performed by uploading the compiled list of protein spectral counts generated by Scaffold software.

The lists of proteins acquired from compilation using the Scaffold program were subjected to the knowledge-based Ingenuity Pathway Analysis (IPA) system for gene ontology analysis. Fig 4 presents the most relevant physiological functions of proteins identified from various tissue samples. From the data analysis of male and female monkey proteins, significant correlation was identified between organs and their physiological roles and functions. For example, GO analysis of proteins from CS tissues correctly clustered them within their ontological category as proteins having roles in “Cardiovascular System Development and Function” and “Hematological System Development and Function”. Additionally, protein classification analysis was performed using the Panther Classification System v8.1 (http://www.pantherdb.org), for which results are shown in supporting information (S2 Fig).

Recently, polyadenylated RNA sequencing from six organs of ten mammal species was carried out to investigate the dynamics of mammalian transcriptome evolution [26]. According to Brawand et al., the rates of expression divergence vary across tissues and chromosomes. Gene expression changes in six organs including brain (prefrontal cortex and brain without cerebellum), cerebellum, heart, kidney, liver and testes from several species were reported. The level of divergence from the common ancestor of all species were very similar. Judging from the total length of the expression tree, neural tissues such as brain and cerebellum were reported to evolve significantly more slowly than other tissues such as testes, for which the evolutionary rate was remarkably fast. Interestingly, the evolution of expression showed differing rates by species. Notably, the primates, monkey and human, showed similarity on total tree lengths of all tested organs as well as similar ratios of the X chromosome and autosome regions. These observations strongly support the fact that the human proteome database may demonstrate similar homology to a larger portion of the entire rhesus monkey proteome, and thus, the current suggested draft map of the monkey proteome acquired from the human database search would be an acceptable and effective alternative strategy for application in global monkey proteomics.

### Validation of proteomic dataset

**Fig 5:**
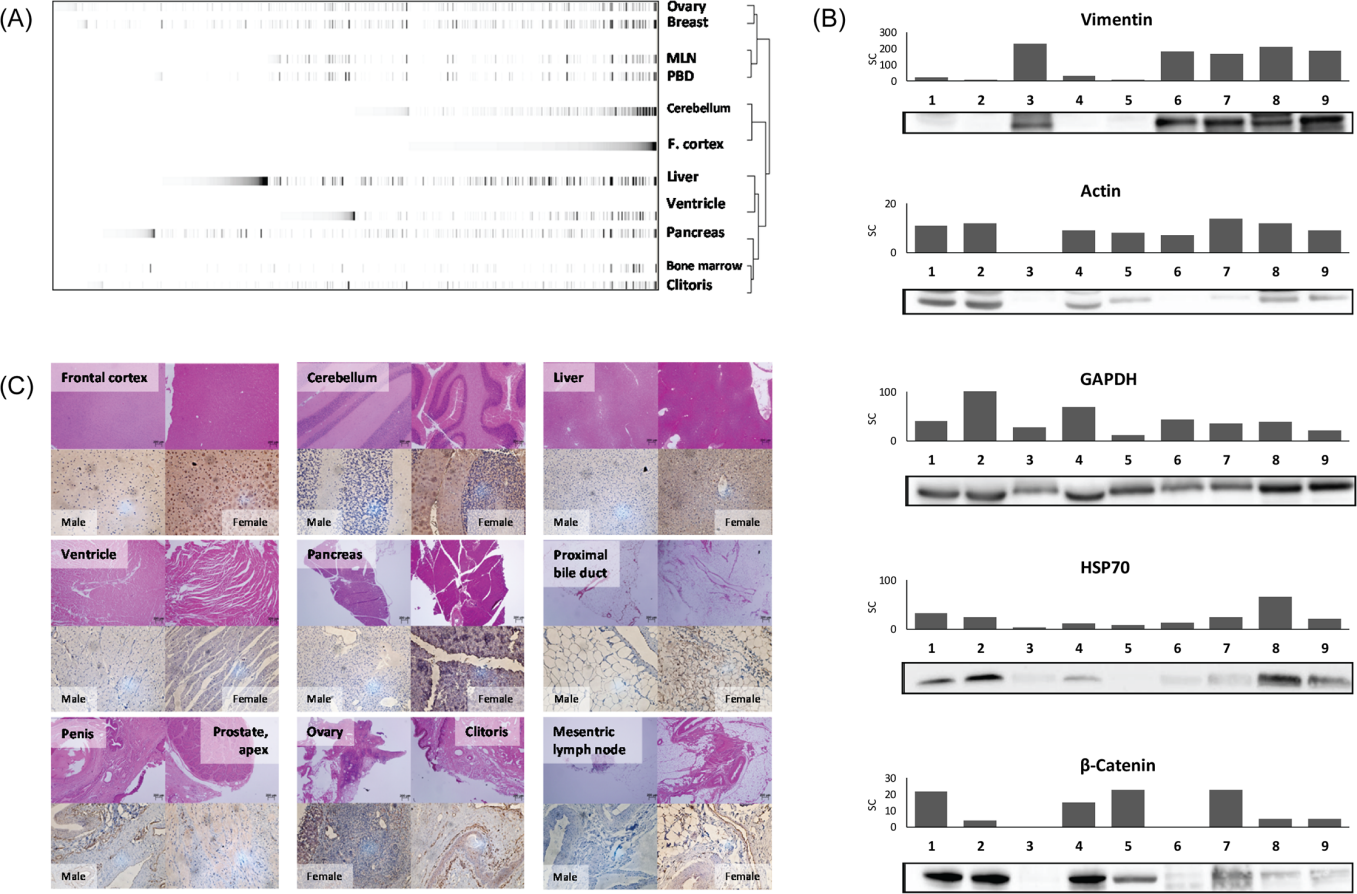
Validation of proteomic datasets. (A) Heat map provided by cluster analysis of proteomic dataset from female monkey indicating the distribution and relative abundances of identified proteins. Also, the clustered organs are presented as a tree view (B) Representative western blot images of common proteins identified from female organs (1, Frontal cortex; 2, cerebellum; 3, mesenteric lymph node; 4, liver; 5, pancreas; 6, proximal bile duct; 7, breast; 8, ovary; 9, clitoris). Band intensities corresponding to their raw spectral counts provided by Scaffold software (C) Immunohistochemistry images (lower) with H&E staining (upper) of monkey organ tissues presenting different expression levels of vimentin. Mesenteric lymph node tissue from male subject was used as a negative control (lower, left).

The validity of the current global rhesus monkey tissue proteomics data, identified by the human alternative database method, was evaluated by cluster analysis, western blot analysis and immunohistochemistry. As shown in Fig 5A, hierarchical analysis provided a clustering tree view informing physiological relevance between monkey tissues. Gene and hierarchical analysis were performed using TreeView (v1.60) software provided by Eisen Lab (http://rana.lbl.gov/EisenSoftware.htm). Briefly, a combined list of protein accession numbers and corresponding spectral counts (as identified from multiple organs) was loaded into the Gene Cluster Software (Eisen et al., Stanford University, USA) to generate a TreeView data file for further analysis[27]. From the cluster analysis of female monkey tissues, ovary, cerebellum and liver showed high relevance with breast, frontal cortex and pancreas respectively, which are considered to have similar physiological functions. Two-dimensional hierarchical analysis also demonstrated that tissues with high relevance showed similar distributions and intensities of protein components. MSLN (mesenteric lymph node) and PBD (proximal bile duct) were displayed as tissues with high similarity, which was due to high intensities of structural proteins (e.g. cytokeratins, actin filaments), which are known to be representative proteins in smooth muscle tissues.

Additionally, differential protein expression exhibited as spectral counts were confirmed by western blot analysis. Target proteins were chosen on the basis of differential expression when comparing their raw spectral counts given by Scaffold analysis of MS data. Fig 5B shows the altered expression of vimentin, β-actin, GAPDH, β-catenin and HSP-70 in female tissue samples. This data corresponds well with the spectral count data in most cases.

The proteomic data was also confirmed by visualizing the expression level of vimentin from the organ tissues using immunohistochemistry (IHC). Fig 5C is showing the images of vimentin expression from various organ tissues from male and female subjects. Male mesenteric lymph node tissue was used as a control since it provided high spectral counts (5C, lower right). IHC images are in good agreement with proteomic dataset, thus confirming higher expression for vimentin in proximal bile duct, mesenteric lymph node, and in the reproductive organ tissues.

## Conclusions

We conclude that the alternative human database search of LC-MS/MS data is a simple and powerful strategy to study large-scale, global proteomics of non-human primate animal models having currently incomplete protein databases. The 3,481 proteins identified with high confidence from twelve organs of male and female rhesus monkeys were established for future rhesus monkey proteome reference data for biomedical research.

These raw data files will be available in the public data repository (http://www.proteomexchange.org/) for further data comparison and analysis. Also these data could be re-searched with a more comprehensive monkey protein database in the future. We will continue to investigate neuronal disease, immunodeficiency virus, and aging using the non-human primate animal model.

## Supporting Information

S1 Fig The comparison of protein identification numbers acquired from LTQ-XL (LTQ) and from LTQ/Orbitrap-XL (Orbitrap). The tissue lysates of pancreas and prostate from the male subject (EL30) were used for the analysis. (A) Venn-diagrams showing protein numbers from each instrument. The advanced mass spectrometer (Orbitrap) has provided more protein identifications than LTQ. (B) The unique proteins given by Orbitrap analysis were examined to evaluate their confidence of identification. More than 80% showed lower spectral counts (<5) and most were revealed to have lower sequence coverage (< 10%).
(TIF)

S2 Fig Biological functions of proteins identified from monkey organs. Bar graphs presenting physiological functions of proteins identified from (A) frontal cortex, cerebellum, (B) right ventricle, mesenteric lymph node, (C) liver, pancreas, proximal bile duct and (D) penis, prostate, breast, ovary and clitoris. Classification analysis was performed using Panther Classification System v8.1 (http://www.pantherdatabase.org).
(TIF)

S1 Table The ARRIVE guidelines checklist. (PDF)

S2 Table Top 20 proteins identified from the search with three databases. (PDF)

S3 Table The raw spectral counts acquired from proteomic analysis of multi-organ tissues of male rhesus monkey. (PDF)

S4 Table The raw spectral counts acquired from proteomic analysis of multi-organ tissues of female rhesus monkey. (PDF)
