# Supplementary figures and images for: A Draft Map of Rhesus Monkey Tissue Proteome for Biomedical Research
Source: PLoS One. 2015 May 14;10(5):e0126243. doi: 10.1371/journal.pone.0126243 (PMC4431823; doi:10.1371/journal.pone.0126243)

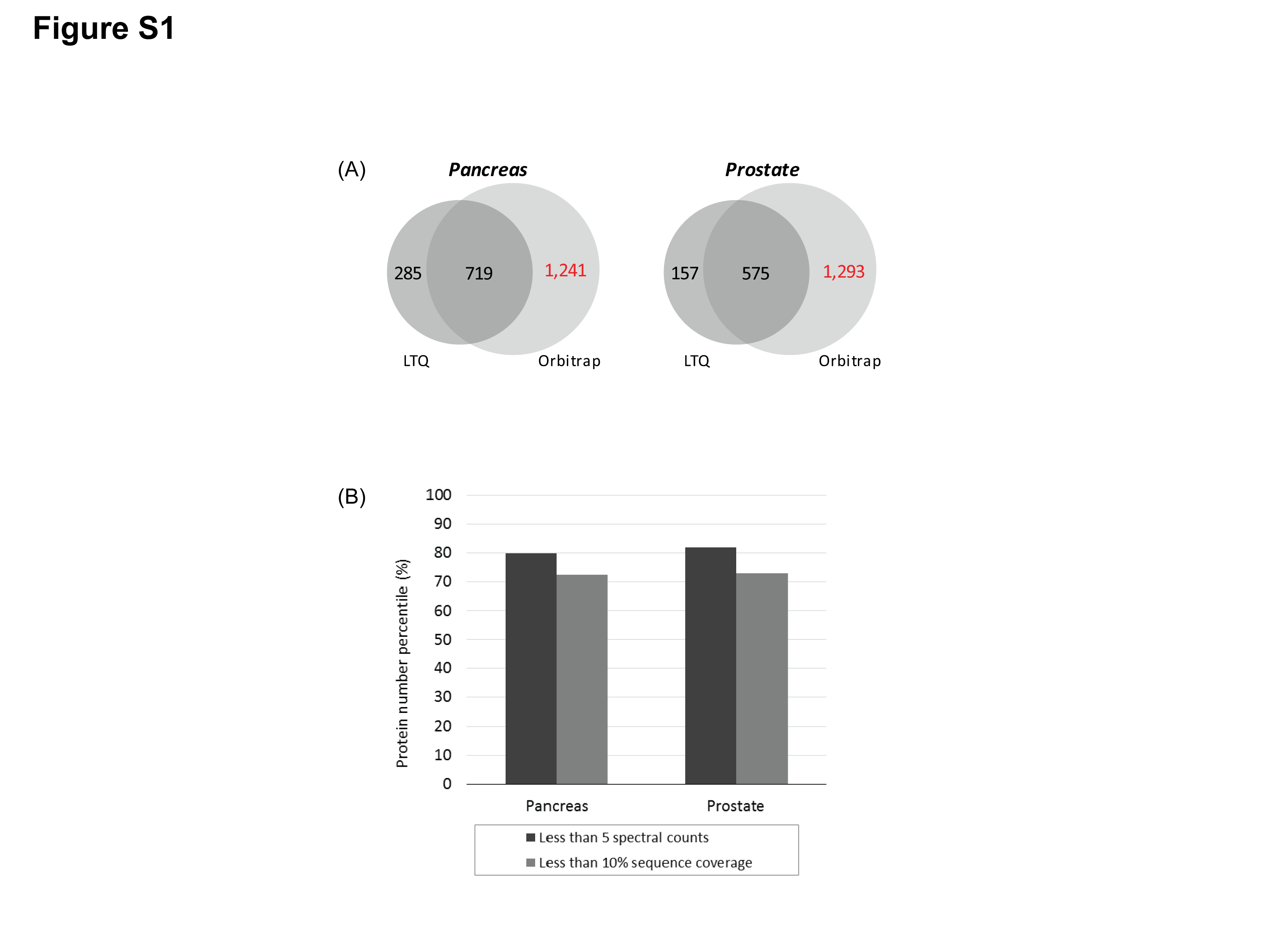

Supplement: S1 Fig — The tissue lysates of pancreas and prostate from the male subject (EL30) were used for the analysis. (A) Venn-diagrams showing protein numbers from each instrument. The advanced mass spectrometer (Orbitrap) has provided more protein identifications than LTQ. (B) The unique proteins given by Orbitrap analysis were examined to evaluate their confidence of identification. More than 80% showed lower spectral counts (<5) and most were revealed to have lower sequence coverage (< 10%). (TIF) [file pone.0126243.s001.tif]

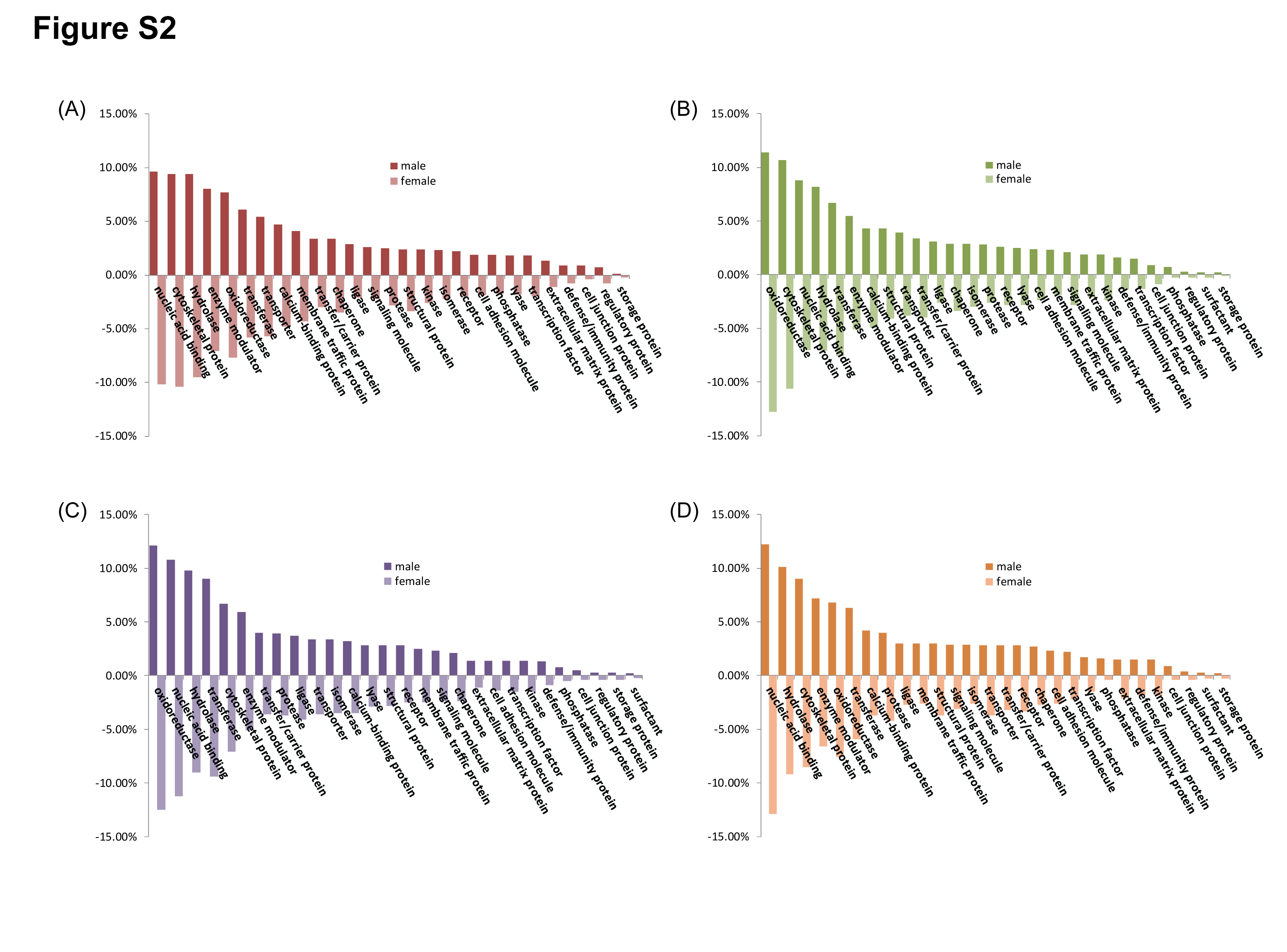

Supplement: S2 Fig — Bar graphs presenting physiological functions of proteins identified from (A) frontal cortex, cerebellum, (B) right ventricle, mesenteric lymph node, (C) liver, pancreas, proximal bile duct and (D) penis, prostate, breast, ovary and clitoris. Classification analysis was performed using Panther Classification System v8.1 (http://www.pantherdatabase.org). (TIF) [file pone.0126243.s002.tif]
