# Supplementary material for: A Draft Map of Rhesus Monkey Tissue Proteome for Biomedical Research
Source: PLoS One. 2015 May 14;10(5):e0126243. doi: 10.1371/journal.pone.0126243 (PMC4431823; doi:10.1371/journal.pone.0126243)
Supplement: S2 Table — (PDF) [file pone.0126243.s004.pdf]

S2_Table Top 20 proteins identified from the search with the following three databases; Uniprot database of *Macaca mulatta* and human, IPI database of human (v3.72). 20 raw data files from the single run of male liver tissue sample were used for comparative analysis.

**Uniprot DB of *Macaca mulatta***				**Uniprot DB of human**				**IPI DB of human v3.72**			
**Description**	**Accession number**	**MW**	**SC[1]**	**Description**	**Accession number**	**MW**	**SC[1]**	**Description**	**Accession number**	**MW**	**SC[1]**
Uncharacterized protein	F7B8E3_MACMU	165 kDa	627	Carbamoyl-phosphate synthase [ammonia], mitochondrial	CPSM_HUMAN	165 kDa	515	Isoform 1 of Carbamoyl-phosphate synthase [ammonia],	IPI00011062	165 kDa	505
Alcohol dehydrogenase 1B	F7HK90_MACMU	40 kDa	312	Alcohol dehydrogenase 1B	ADH1B_HUMAN	40 kDa	261	Alcohol dehydrogenase 1B	IPI00473031	40 kDa	261
Uncharacterized protein	F7HK88_MACMU	40 kDa	285	Keratin, type II cytoskeletal 1	K2C1_HUMAN	66 kDa	253	Keratin, type II cytoskeletal 1	IPI00220327	66 kDa	249
Catalase	F6PLF0_MACMU	60 kDa	247	Catalase	CATA_HUMAN	60 kDa	215	Catalase	IPI00465436	60 kDa	212
Cytokeratin-1	F7B786_MACMU	65 kDa	229	Alcohol dehydrogenase 1C	ADH1G_HUMAN	40 kDa	189	Alcohol dehydrogenase 1C	IPI00465343	40 kDa	190
Uncharacterized protein	F6Y3X8_MACMU	40 kDa	215	Keratin, type II cytoskeletal 2 epidermal	K22E_HUMAN	65 kDa	120	Keratin, type II cytoskeletal 2 epidermal	IPI00021304	66 kDa	115
Uncharacterized protein	F6Y3U8_MACMU	40 kDa	191	Hemoglobin subunit alpha	HBA_HUMAN	15 kDa	104	Hemoglobin subunit alpha	IPI00410714	15 kDa	101
Hemoglobin beta chain	F7AV81_MACMU	16 kDa	176	Keratin, type I cytoskeletal 10	K1C10_HUMAN	59 kDa	91	Keratin, type I cytoskeletal 10	IPI00009865	59 kDa	91
Hemoglobin alpha chain	G7NQC3_MACMU	15 kDa	144	Keratin, type I cytoskeletal 9	K1C9_HUMAN	62 kDa	85	Keratin, type I cytoskeletal 9	IPI00019359	62 kDa	79
Uncharacterized protein (Fragment)	F6PYE7_MACMU	273 kDa	133	Hemoglobin subunit delta	HBD_HUMAN	16 kDa	74	Hemoglobin subunit delta	IPI00473011	16 kDa	73
Serum albumin (Fragment)	ALBU_MACMU	68 kDa	107	Hemoglobin subunit beta	HBB_HUMAN	16 kDa	73	Hemoglobin subunit beta	IPI00654755	16 kDa	72
Uncharacterized protein OS=Macaca mulatta	F6T457_MACMU	148 kDa	101	Putative elongation factor 1-alpha-like 3	EF1A3_HUMAN	50 kDa	68	Argininosuccinate synthase	IPI00020632	51 kDa	63
Uncharacterized protein	F7B5E6_MACMU	57 kDa	86	ATP synthase subunit beta, mitochondrial	ATPB_HUMAN	57 kDa	67	Elongation factor 1-alpha 1	IPI00396485	50 kDa	63
Uncharacterized protein	F7BGW5_MACMU	104 kDa	84	Argininosuccinate synthase	ASSY_HUMAN	47 kDa	64	Betaine--homocysteine S-methyltransferase 1	IPI00004101	45 kDa	63
Uncharacterized protein	F7GWT6_MACMU	57 kDa	72	Betaine--homocysteine S-methyltransferase 1	BHMT1_HUMAN	45 kDa	63	ATP synthase subunit beta, mitochondrial	IPI00303476	57 kDa	63
Cytokeratin-2e	F7AEK9_MACMU	66 kDa	65	Endoplasmin	ENPL_HUMAN	92 kDa	58	Glutathione S-transferase A1	IPI00657682	26 kDa	57
Uncharacterized protein	F6YK88_MACMU	45 kDa	64	Aldehyde oxidase	AOXA_HUMAN	148 kDa	57	C-1-tetrahydrofolate synthase, cytoplasmic	IPI00218342	102 kDa	57
ATP synthase subunit beta	F6SIE0_MACMU	57 kDa	64	C-1-tetrahydrofolate synthase, cytoplasmic	C1TC_HUMAN	102 kDa	57	Endoplasmin	IPI00027230	92 kDa	57
ATP synthase subunit beta	F7F3I6_MACMU	56 kDa	63	Glutathione S-transferase A1	GSTA1_HUMAN	26 kDa	56	Aldehyde oxidase	IPI00029715	148 kDa	56
Uncharacterized protein	F6YGC3_MACMU	37 kDa	63	Glutathione S-transferase A3	GSTA3_HUMAN	25 kDa	56	Glutathione S-transferase A3	IPI00003929	25 kDa	56

[1]SC; spectral counts
