# Supplementary material for: A Draft Map of Rhesus Monkey Tissue Proteome for Biomedical Research
Source: PLoS One. 2015 May 14;10(5):e0126243. doi: 10.1371/journal.pone.0126243 (PMC4431823; doi:10.1371/journal.pone.0126243)
Supplement: S3 Table — (PDF) [file pone.0126243.s005.pdf]

S3_Table The raw spectral counts acquired from proteomic analysis of multi-organ tissues of male rhesus monkey. List of proteins were acquired from nine tissues, frontal cortex, cerebellum, right ventricle, mesentric lymph node, liver, pancreas, proxima

Description	Accession	MW	Raw spectral counts								
			Frontal Cortex	Cerebellum	Right Ventricle	Mesentric lymph node	Liver	Pancreas	Proximal bile duct	Penis	Prostate
Keratin, type II cytoskeletal 1	K2C1_HUMAN	66 kDa	291	176	418	563	253	496	384	377	532
Keratin, type I cytoskeletal 10	K1C10_HUMAN	59 kDa	206	32	314	261	91	285	133	193	313
Keratin, type I cytoskeletal 9	K1C9_HUMAN	62 kDa	112	84	202	192	85	243	129	99	222
Keratin, type II cytoskeletal 2 epidermal	K22E_HUMAN	65 kDa	83	41	165	245	120	142	145	112	259
Serum albumin	ALBU_HUMAN	69 kDa	18	37	74	251	52	52	426	106	103
Spectrin alpha chain, non-erythrocytic 1	SPTN1_HUMAN	285 kDa	190	284	51	84	16	83	73	106	55
Actin, cytoplasmic 1	ACTB_HUMAN	42 kDa	63	119	94	124	47	88	166	86	149
Myosin-7	MYH7_HUMAN	223 kDa	2	0	873	0	0	9	0	0	0
Actin, alpha cardiac muscle 1	ACTC_HUMAN	42 kDa	45	83	129	114	33	60	158	68	175
ATP synthase subunit beta	ATPB_HUMAN	57 kDa	62	133	233	53	67	68	47	35	39
Vimentin	VIME_HUMAN	54 kDa	35	54	28	219	1	14	189	140	37
Neuroblast differentiation-associated protein AHNAK	AHNK_HUMAN	629 kDa	8	12	47	198	0	20	167	256	9
Myosin-6	MYH6_HUMAN	224 kDa	0	0	693	0	0	5	0	0	0
Tubulin alpha-1B chain	TBA1B_HUMAN	50 kDa	181	190	21	68	19	53	60	45	28
Tubulin beta chain	TBB5_HUMAN	50 kDa	120	207	28	76	19	48	75	67	23
Filamin-A	FLNA_HUMAN	281 kDa	5	2	9	52	3	15	132	91	315
Tubulin alpha-1A chain	TBA1A_HUMAN	50 kDa	165	186	15	61	10	37	55	43	27
Clathrin heavy chain 1	CLH1_HUMAN	192 kDa	222	90	37	23	9	76	23	53	49
Tubulin beta-4A chain	TBB4A_HUMAN	50 kDa	112	241	17	54	11	28	52	46	13
Tubulin beta-4B chain	TBB4B_HUMAN	50 kDa	115	222	25	68	18	0	64	56	0
Tubulin beta-2B chain	TBB2B_HUMAN	50 kDa	117	183	20	57	13	37	61	46	20
Keratin, type II cytoskeletal 6B	K2C6B_HUMAN	60 kDa	0	0	63	89	0	117	78	85	101
Carbamoyl-phosphate synthase [ammonia]	CPSM_HUMAN	165 kDa	0	0	0	0	515	0	0	0	0
Keratin, type II cytoskeletal 5	K2C5_HUMAN	62 kDa	20	0	53	60	30	100	38	110	94
Spectrin beta chain, non-erythrocytic 1	SPTB2_HUMAN	275 kDa	114	173	8	58	11	40	57	28	4
ATP synthase subunit alpha	ATPA_HUMAN	60 kDa	47	66	148	25	21	54	18	26	48
Putative elongation factor 1-alpha-like 3	EF1A3_HUMAN	50 kDa	14	14	10	42	68	122	43	83	55
Actin, aortic smooth muscle	ACTA_HUMAN	42 kDa	0	0	0	109	0	0	153	0	171
78 kDa glucose-regulated protein	GRP78_HUMAN	72 kDa	21	14	20	26	45	201	14	26	59
Sodium/potassium-transporting ATPase subunit alpha-1	AT1A1_HUMAN	113 kDa	181	133	39	0	1	11	3	34	5
Tubulin beta-2A chain	TBB2A_HUMAN	50 kDa	117	184	0	58	0	0	0	47	0
Annexin A2	ANXA2_HUMAN	39 kDa	12	8	22	50	9	36	120	99	44
Titin	TITIN_HUMAN	3816 kDa	1	0	391	1	0	0	0	2	0
Keratin, type I cytoskeletal 14	K1C14_HUMAN	52 kDa	18	3	37	35	5	76	34	111	70
Heat shock cognate 71 kDa protein	HSP7C_HUMAN	71 kDa	42	65	35	41	42	52	25	44	33
Hemoglobin subunit alpha	HBA_HUMAN	15 kDa	37	19	51	36	104	13	30	75	9
Sodium/potassium-transporting ATPase subunit alpha-3	AT1A3_HUMAN	112 kDa	181	120	45	0	0	0	0	21	0
Tubulin beta-3 chain	TBB3_HUMAN	50 kDa	102	160	0	49	0	0	48	0	0
Vinculin	VINC_HUMAN	124 kDa	4	1	45	90	10	39	61	30	63
Alpha-enolase	ENOA_HUMAN	47 kDa	26	76	20	38	24	40	25	61	25
Tubulin alpha-4A chain	TBA4A_HUMAN	50 kDa	124	130	0	0	0	40	0	31	0
Annexin A1	ANXA1_HUMAN	39 kDa	8	50	8	78	0	23	85	62	11
Pyruvate kinase PKM	KPYM_HUMAN	58 kDa	67	39	87	8	0	17	6	65	36
Hemoglobin subunit beta	HBB_HUMAN	16 kDa	29	25	30	43	73	11	45	55	13
Fatty acid synthase	FAS_HUMAN	273 kDa	4	0	0	182	49	0	73	14	0
Keratin, type II cytoskeletal 6A	K2C6A_HUMAN	60 kDa	0	0	48	47	0	105	56	64	0
Elongation factor 1-alpha 2	EF1A2_HUMAN	50 kDa	16	19	17	28	44	74	30	54	34
Cytoplasmic dynein 1 heavy chain 1	DYHC1_HUMAN	532 kDa	120	62	50	2	1	6	4	65	1

Description	Accession	MW	Raw spectral counts								
			Frontal Cortex	Cerebellum	Right Ventricle	Mesentric lymph node	Liver	Pancreas	Proximal bile duct	Penis	Prostate
Keratin, type II cytoskeletal 8	K2C8_HUMAN	54 kDa	0	0	0	25	29	163	18	0	75
Endoplasmin	ENPL_HUMAN	92 kDa	11	12	12	25	58	121	17	14	37
Glyceraldehyde-3-phosphate dehydrogenase	G3P_HUMAN	36 kDa	40	72	32	22	37	15	25	31	27
Plectin	PLEC_HUMAN	532 kDa	65	46	40	9	0	11	10	99	21
Alcohol dehydrogenase 1B	ADH1B_HUMAN	40 kDa	0	0	0	14	261	10	16	0	0
ADP/ATP translocase 3	ADT3_HUMAN	33 kDa	65	36	90	18	19	28	15	13	11
Heat shock protein HSP 90-beta	HS90B_HUMAN	83 kDa	21	44	25	32	25	50	31	33	33
14-3-3 protein epsilon	1433E_HUMAN	29 kDa	49	57	29	31	18	17	24	43	21
Heat shock protein HSP 90-alpha	HS90A_HUMAN	85 kDa	27	47	18	28	25	47	32	26	36
Beta-actin-like protein 2	ACTBL_HUMAN	42 kDa	18	37	41	42	0	0	63	23	59
Hemoglobin subunit delta	HBD_HUMAN	16 kDa	24	19	25	29	74	12	39	47	12
Catalase	CATA_HUMAN	60 kDa	3	0	12	14	215	17	0	10	9
Malate dehydrogenase	MDHM_HUMAN	36 kDa	41	52	48	28	24	17	23	18	25
Actin, alpha skeletal muscle	ACTS_HUMAN	42 kDa	0	0	121	0	0	0	155	0	0
Alpha-actinin-1	ACTN1_HUMAN	103 kDa	36	10	17	25	0	35	35	31	84
Annexin A6	ANXA6_HUMAN	76 kDa	28	20	27	31	27	44	23	13	55
ADP/ATP translocase 1	ADT1_HUMAN	33 kDa	45	19	129	11	15	15	12	0	14
Myosin-9	MYH9_HUMAN	227 kDa	22	2	0	26	5	97	8	97	3
Alpha-actinin-4	ACTN4_HUMAN	105 kDa	27	8	14	31	20	38	32	42	43
Prelamin-A/C	LMNA_HUMAN	74 kDa	5	3	22	36	0	49	26	84	27
POTE ankyrin domain family member I	POTEI_HUMAN	121 kDa	26	33	29	48	0	0	60	22	31
Sodium/potassium-transporting ATPase subunit alpha-2	AT1A2_HUMAN	112 kDa	134	92	21	0	0	0	0	0	0
L-lactate dehydrogenase B chain	LDHB_HUMAN	37 kDa	35	59	36	36	0	14	36	6	24
Protein disulfide-isomerase	PDIA1_HUMAN	57 kDa	6	0	4	15	23	147	6	18	25
Pancreatic alpha-amylase	AMYP_HUMAN	58 kDa	0	0	4	0	0	234	0	0	0
Phosphoglycerate kinase 1	PGK1_HUMAN	45 kDa	27	33	21	32	18	32	24	25	25
60 kDa heat shock protein	CH60_HUMAN	61 kDa	36	29	53	23	49	12	8	12	14
14-3-3 protein zeta/delta	1433Z_HUMAN	28 kDa	34	41	13	25	9	15	26	40	24
Transgelin	TAGL_HUMAN	23 kDa	5	1	4	35	4	7	57	22	92
Annexin A5	ANXA5_HUMAN	36 kDa	21	13	19	23	13	33	45	24	33
Tubulin alpha-8 chain	TBA8_HUMAN	50 kDa	104	101	17	0	0	0	0	0	0
Syntaxin-binding protein 1	STXB1_HUMAN	68 kDa	97	124	0	0	0	0	0	0	0
Filamin-C	FLNC_HUMAN	291 kDa	0	0	87	0	0	2	17	0	115
Creatine kinase B-type	KCRB_HUMAN	43 kDa	48	98	9	0	0	0	4	0	58
Alcohol dehydrogenase 1C	ADH1G_HUMAN	40 kDa	0	0	0	9	189	7	11	0	0
ADP/ATP translocase 2	ADT2_HUMAN	33 kDa	38	23	74	10	28	18	0	15	8
Keratin, type I cytoskeletal 16	K1C16_HUMAN	51 kDa	0	0	34	0	0	76	41	61	0
Protein disulfide-isomerase A3	PDIA3_HUMAN	57 kDa	13	6	12	15	19	82	11	21	31
Aconitate hydratase	ACON_HUMAN	85 kDa	23	34	65	12	4	26	15	12	17
Fructose-bisphosphate aldolase C	ALDOC_HUMAN	39 kDa	34	90	22	27	0	1	22	5	6
NAD(P) transhydrogenase	NNTM_HUMAN	114 kDa	31	7	97	8	22	30	6	4	0
Dihydropyrimidinase-related protein 2	DPYL2_HUMAN	62 kDa	76	102	5	2	0	3	1	8	7
Filamin-B	FLNB_HUMAN	278 kDa	3	0	0	21	20	31	13	40	71
Sarcoplasmic/endoplasmic reticulum calcium ATPase 2	AT2A2_HUMAN	115 kDa	37	9	131	0	0	10	0	0	10
Trifunctional enzyme subunit alpha	ECHA_HUMAN	83 kDa	8	8	78	19	24	29	11	16	2
Desmoplakin	DESP_HUMAN	332 kDa	2	0	32	0	0	14	0	145	1
Polymerase I and transcript release factor	PTRF_HUMAN	43 kDa	0	0	22	83	0	2	56	21	7
Ubiquitin-40S ribosomal protein S27a	RS27A_HUMAN	18 kDa	26	13	25	20	8	17	20	46	15
Talin-1	TLN1_HUMAN	270 kDa	5	1	2	53	15	8	47	29	27
Elongation factor 2	EF2_HUMAN	95 kDa	11	7	20	3	12	91	4	27	11

Description	Accession	MW	Raw spectral counts								
			Frontal Cortex	Cerebellum	Right Ventricle	Mesentric lymph node	Liver	Pancreas	Proximal bile duct	Penis	Prostate
Histone H2A type 2-A	H2A2A_HUMAN	14 kDa	15	24	16	29	0	15	47	37	2
Alpha-amylase 1	AMY1_HUMAN	58 kDa	0	0	0	0	0	185	0	0	0
Transitional endoplasmic reticulum ATPase	TERA_HUMAN	89 kDa	18	14	21	15	16	47	10	19	23
Histone H1.2	H12_HUMAN	21 kDa	13	36	17	15	10	23	18	50	0
Desmin	DESM_HUMAN	54 kDa	0	0	32	34	0	0	41	0	75
Tubulin beta-8 chain	TBB8_HUMAN	50 kDa	43	101	13	0	0	0	0	22	0
Lumican	LUM_HUMAN	38 kDa	0	0	27	40	0	4	74	29	5
Phosphate carrier protein	MPCP_HUMAN	40 kDa	25	16	74	7	13	21	7	5	10
Serotransferrin	TRFE_HUMAN	77 kDa	6	4	13	31	12	8	61	18	20
ADP-ribosylation factor 3	ARF3_HUMAN	21 kDa	20	29	10	9	23	36	7	15	21
Heat shock 70 kDa protein 1A/1B	HSP71_HUMAN	70 kDa	22	25	25	11	5	13	11	38	19
Stress-70 protein	GRP75_HUMAN	74 kDa	27	20	38	12	21	24	6	10	10
Collagen alpha-1(XIV) chain	COEA1_HUMAN	194 kDa	0	0	9	57	0	0	82	2	17
14-3-3 protein gamma	1433G_HUMAN	28 kDa	20	31	9	22	7	9	22	25	16
14-3-3 protein beta/alpha	1433B_HUMAN	28 kDa	21	27	8	18	6	12	19	28	18
Fatty acid-binding protein, adipocyte	FABP4_HUMAN	15 kDa	0	0	4	69	0	0	84	0	0
Isocitrate dehydrogenase [NADP]	IDHP_HUMAN	51 kDa	17	19	58	18	11	16	8	8	1
Voltage-dependent anion-selective channel protein 2	VDAC2_HUMAN	32 kDa	26	37	43	7	5	10	6	7	12
Keratin, type I cytoskeletal 13	K1C13_HUMAN	50 kDa	14	0	38	0	0	69	0	29	0
Myosin-binding protein C, cardiac-type	MYPC3_HUMAN	141 kDa	0	0	149	0	0	0	0	0	0
Decorin	PGS2_HUMAN	40 kDa	0	0	8	25	0	0	70	39	4
Triosephosphate isomerase	TPIS_HUMAN	31 kDa	23	30	16	11	10	6	8	21	18
Phosphatidylethanolamine-binding protein 1	PEBP1_HUMAN	21 kDa	19	27	6	10	40	11	13	11	6
Ribosome-binding protein 1	RRBP1_HUMAN	152 kDa	0	0	0	0	16	118	0	0	9
Heterogeneous nuclear ribonucleoproteins A2/B1	ROA2_HUMAN	37 kDa	16	35	10	7	7	14	8	34	11
Aldehyde dehydrogenase	ALDH2_HUMAN	56 kDa	5	7	26	18	34	10	19	16	6
40S ribosomal protein S3	RS3_HUMAN	27 kDa	8	4	7	11	12	57	6	28	6
Microtubule-associated protein 1B	MAP1B_HUMAN	271 kDa	80	45	12	0	0	0	0	0	1
Peroxiredoxin-2	PRDX2_HUMAN	22 kDa	19	29	11	11	14	15	14	16	8
Apolipoprotein A-I	APOA1_HUMAN	31 kDa	11	11	15	27	10	7	31	13	12
Alpha-1-antitrypsin	A1AT_HUMAN	47 kDa	3	1	9	21	2	7	54	21	19
Tropomyosin alpha-1 chain	TPM1_HUMAN	33 kDa	13	8	55	9	0	3	11	11	26
Peroxiredoxin-1	PRDX1_HUMAN	22 kDa	14	20	4	15	8	9	19	28	18
Carboxypeptidase A1	CBPA1_HUMAN	47 kDa	0	0	0	0	0	135	0	0	0
Collagen alpha-3(VI) chain	CO6A3_HUMAN	344 kDa	0	0	2	12	0	0	14	98	8
L-lactate dehydrogenase A chain	LDHA_HUMAN	37 kDa	10	16	8	27	21	8	16	16	11
Prolargin	PRELP_HUMAN	44 kDa	3	1	13	21	0	0	67	28	0
Myosin-1	MYH1_HUMAN	223 kDa	0	0	133	0	0	0	0	0	0
Spectrin beta chain, non-erythrocytic 2	SPTN2_HUMAN	271 kDa	30	79	0	0	1	0	0	22	0
14-3-3 protein theta	1433T_HUMAN	28 kDa	18	25	7	14	4	8	16	28	11
Tropomyosin alpha-4 chain	TPM4_HUMAN	29 kDa	13	13	14	20	3	3	18	21	26
Keratin, type I cytoskeletal 18	K1C18_HUMAN	48 kDa	0	0	0	0	24	69	0	0	38
Cytochrome b-c1 complex subunit 2	QCR2_HUMAN	48 kDa	23	27	34	7	10	11	7	10	1
Keratin, type I cytoskeletal 19	K1C19_HUMAN	44 kDa	0	0	9	24	0	71	19	0	7
Syntaxin-1B	STX1B_HUMAN	33 kDa	58	71	0	0	0	0	0	0	0
Protein disulfide-isomerase A4	PDIA4_HUMAN	73 kDa	0	0	0	1	30	66	0	4	27
Glutamate dehydrogenase 1	DHE3_HUMAN	61 kDa	22	14	15	3	33	8	3	10	19
Complement C3	CO3_HUMAN	187 kDa	1	0	13	16	10	1	62	15	9
Dolichyl-diphosphooligosaccharide--protein glycosyltransferase subunit 2	RPN2_HUMAN	69 kDa	3	0	1	0	9	105	0	0	6
Myelin basic protein	MBP_HUMAN	33 kDa	69	53	0	0	0	0	0	1	0

Description	Accession	MW	Raw spectral counts								
			Frontal Cortex	Cerebellum	Right Ventricle	Mesentric lymph node	Liver	Pancreas	Proximal bile duct	Penis	Prostate
Tropomyosin alpha-3 chain	TPM3_HUMAN	33 kDa	19	12	33	12	0	5	10	16	16
Protein disulfide-isomerase A6	PDIA6_HUMAN	48 kDa	2	2	5	10	13	56	3	8	24
Perilipin-4	PLIN4_HUMAN	134 kDa	0	0	18	87	0	0	17	0	0
Prohibitin	PHB_HUMAN	30 kDa	20	21	19	10	19	7	8	11	6
Ankyrin-2	ANK2_HUMAN	434 kDa	61	56	2	0	0	0	0	1	0
Malate dehydrogenase, cytoplasmic	MDHC_HUMAN	36 kDa	19	27	15	13	7	6	16	6	11
Voltage-dependent anion-selective channel protein 1	VDAC1_HUMAN	31 kDa	38	23	25	3	4	6	4	4	13
Fructose-bisphosphate aldolase A	ALDOA_HUMAN	39 kDa	28	20	33	8	0	3	6	10	12
Synapsin-1	SYN1_HUMAN	74 kDa	68	50	0	0	0	0	0	0	0
Dolichyl-diphosphooligosaccharide--protein glycosyltransferase subunit 1	RPN1_HUMAN	69 kDa	1	0	0	0	14	93	0	2	8
Hexokinase-1	HXK1_HUMAN	102 kDa	26	47	18	4	0	0	4	8	9
Aldo-keto reductase family 1 member C1	AK1C1_HUMAN	37 kDa	6	2	5	25	31	18	21	5	3
EH domain-containing protein 2	EHD2_HUMAN	61 kDa	1	0	9	58	0	0	29	11	8
Glial fibrillary acidic protein	GFAP_HUMAN	50 kDa	29	86	0	0	0	0	0	0	0
ATP synthase subunit O	ATPO_HUMAN	23 kDa	18	23	23	9	17	6	11	6	2
Rab GDP dissociation inhibitor beta	GDIB_HUMAN	51 kDa	21	19	10	7	9	9	6	15	19
Trifunctional enzyme subunit beta	ECHB_HUMAN	51 kDa	1	3	46	14	8	20	7	7	8
3-hydroxyacyl-CoA dehydrogenase type-2	HCD2_HUMAN	27 kDa	16	12	18	4	35	12	1	13	1
Histone H2B type 1-J	H2B1J_HUMAN	14 kDa	8	16	11	12	0	8	22	33	1
Selenium-binding protein 1	SBP1_HUMAN	52 kDa	0	0	16	25	19	8	13	5	24
Glycogen phosphorylase, brain form	PYGB_HUMAN	97 kDa	12	29	33	10	10	0	5	8	2
Myosin-3	MYH3_HUMAN	224 kDa	0	0	109	0	0	0	0	0	0
NADH-ubiquinone oxidoreductase 75 kDa subunit	NDUS1_HUMAN	79 kDa	22	27	28	3	5	12	1	10	0
ADP-ribosylation factor 4	ARF4_HUMAN	21 kDa	16	17	0	6	12	31	4	8	14
Glycogen phosphorylase, muscle form	PYGM_HUMAN	97 kDa	16	23	44	8	9	1	5	1	0
Bile salt-activated lipase	CEL_HUMAN	79 kDa	0	0	0	0	0	107	0	0	0
Plasma membrane calcium-transporting ATPase 1	AT2B1_HUMAN	139 kDa	64	37	2	0	0	0	0	3	0
Prohibitin-2	PHB2_HUMAN	33 kDa	12	22	18	7	9	14	9	11	4
Voltage-dependent anion-selective channel protein 3	VDAC3_HUMAN	31 kDa	25	21	28	3	6	3	6	9	5
Heterogeneous nuclear ribonucleoprotein K	HNRPK_HUMAN	51 kDa	16	12	9	0	4	19	1	34	11
2-oxoglutarate dehydrogenase	ODO1_HUMAN	116 kDa	11	8	55	6	5	8	1	7	5
Aspartate aminotransferase	AATM_HUMAN	48 kDa	14	25	28	0	11	9	2	10	6
Guanine nucleotide-binding protein G(o) subunit alpha	GNAO_HUMAN	40 kDa	40	59	2	0	0	0	0	0	2
Plasma membrane calcium-transporting ATPase 2	AT2B2_HUMAN	137 kDa	48	50	4	0	0	0	0	0	1
Rab GDP dissociation inhibitor alpha	GDIA_HUMAN	51 kDa	34	36	5	0	0	0	0	12	16
14-3-3 protein eta	1433F_HUMAN	28 kDa	19	21	6	13	4	6	0	23	11
Glucose-6-phosphate isomerase	G6PI_HUMAN	63 kDa	12	8	18	16	2	10	14	7	15
Heat shock protein beta-1	HSPB1_HUMAN	23 kDa	5	5	23	13	0	3	13	30	10
Glycerol-3-phosphate dehydrogenase [NAD(+)], cytoplasmic	GPDA_HUMAN	38 kDa	0	1	1	51	11	0	38	0	0
Myosin-4	MYH4_HUMAN	223 kDa	0	0	101	0	0	0	0	0	0
40S ribosomal protein SA	RSSA_HUMAN	33 kDa	7	3	6	5	8	44	4	15	8
Calnexin	CALX_HUMAN	68 kDa	8	9	9	8	13	20	4	8	20
Electron transfer flavoprotein subunit beta	ETFB_HUMAN	28 kDa	8	7	28	10	20	6	6	11	3
Cofilin-1	COF1_HUMAN	19 kDa	13	22	4	4	11	9	8	17	10
Terminal uridylyltransferase 4	TUT4_HUMAN	185 kDa	8	15	9	24	0	8	0	21	12
Ras GTPase-activating-like protein IQGAP1	IQGA1_HUMAN	189 kDa	0	0	0	6	0	15	8	54	14
Myosin-11	MYH11_HUMAN	227 kDa	11	1	0	15	1	16	7	24	21
Thioredoxin-dependent peroxide reductase	PRDX3_HUMAN	28 kDa	14	15	12	13	13	5	9	6	8
Peroxiredoxin-5	PRDX5_HUMAN	22 kDa	25	17	15	1	10	13	1	8	4
Glutathione S-transferase Mu 2	GSTM2_HUMAN	26 kDa	7	13	9	7	15	20	9	8	6

Description	Accession	MW	Raw spectral counts								
			Frontal Cortex	Cerebellum	Right Ventricle	Mesentric lymph node	Liver	Pancreas	Proximal bile duct	Penis	Prostate
Perilipin-1	PLIN1_HUMAN	56 kDa	0	0	0	66	0	0	28	0	0
Coiled-coil domain-containing protein 180	CC180_HUMAN	191 kDa	6	11	9	15	9	10	11	9	13
Dynamin-1	DYN1_HUMAN	97 kDa	42	50	0	0	0	0	0	0	0
Pancreatic triacylglycerol lipase	LIPP_HUMAN	51 kDa	0	0	0	0	0	91	0	0	0
Mitochondrial 2-oxoglutarate/malate carrier protein	M2OM_HUMAN	34 kDa	26	16	29	0	9	0	1	5	3
Adenosylhomocysteinase	SAHH_HUMAN	48 kDa	0	5	1	11	18	24	8	13	9
Staphylococcal nuclease domain-containing protein 1	SND1_HUMAN	102 kDa	0	0	0	3	9	57	0	9	11
Vesicle-fusing ATPase	NSF_HUMAN	83 kDa	46	38	0	0	0	1	0	2	0
Peroxiredoxin-6	PRDX6_HUMAN	25 kDa	8	14	12	14	6	3	7	12	11
Moesin	MOES_HUMAN	68 kDa	4	4	8	17	3	9	15	15	12
Coatomer subunit alpha	COPA_HUMAN	138 kDa	0	0	0	0	4	66	0	9	8
Neprilysin	NEP_HUMAN	86 kDa	0	0	0	0	0	0	0	0	87
Histone H4	H4_HUMAN	11 kDa	16	0	10	8	0	9	18	23	3
3-ketoacyl-CoA thiolase	THIM_HUMAN	42 kDa	3	0	15	17	29	10	3	8	2
Cytosol aminopeptidase	AMPL_HUMAN	56 kDa	1	0	17	3	10	4	2	15	35
Excitatory amino acid transporter 1	EAA1_HUMAN	60 kDa	30	55	1	0	0	0	0	0	0
Dihydrolipoyl dehydrogenase	DLDH_HUMAN	54 kDa	5	12	39	9	4	5	2	3	7
Excitatory amino acid transporter 2	EAA2_HUMAN	62 kDa	75	6	5	0	0	0	0	0	0
Histone H2B type 1-C/E/F/G/I	H2B1C_HUMAN	14 kDa	7	0	10	12	0	0	23	34	0
Putative tubulin-like protein alpha-4B	TBA4B_HUMAN	28 kDa	0	53	0	15	0	0	17	0	0
Calcium-binding mitochondrial carrier protein Aralar1	CMC1_HUMAN	75 kDa	27	22	30	0	4	2	0	0	0
Citrate synthase	CISY_HUMAN	52 kDa	6	10	24	15	0	7	4	10	9
Hemoglobin subunit gamma-1	HBG1_HUMAN	16 kDa	0	0	11	0	40	0	0	30	4
Microtubule-associated protein 2	MTAP2_HUMAN	200 kDa	77	7	0	0	0	0	0	0	0
Peptidyl-prolyl cis-trans isomerase B	PPIB_HUMAN	24 kDa	6	6	4	4	19	21	10	7	7
Creatine kinase M-type	KCRM_HUMAN	43 kDa	1	0	83	0	0	0	0	0	0
Cullin-associated NEDD8-dissociated protein 1	CAND1_HUMAN	136 kDa	21	19	1	1	5	2	3	26	5
Myosin-10	MYH10_HUMAN	229 kDa	39	6	0	0	0	25	0	13	0
Synapsin-2	SYN2_HUMAN	63 kDa	45	36	0	0	0	0	0	0	1
AP-2 complex subunit beta	AP2B1_HUMAN	105 kDa	45	20	4	0	3	3	2	5	0
Phosphoglucomutase-1	PGM1_HUMAN	61 kDa	10	2	10	3	18	19	0	7	13
Very long-chain specific acyl-CoA dehydrogenase	ACADV_HUMAN	70 kDa	2	2	43	1	12	15	0	4	3
Membrane primary amine oxidase	AOC3_HUMAN	85 kDa	0	0	1	43	0	0	32	0	6
Ras-related protein Rab-7a	RAB7A_HUMAN	23 kDa	15	13	0	6	5	7	5	17	13
Ubiquitin-like modifier-activating enzyme 1	UBA1_HUMAN	118 kDa	14	11	10	4	4	12	5	9	12
Gelsolin	GELS_HUMAN	86 kDa	4	1	6	12	0	2	26	25	5
Isocitrate dehydrogenase [NAD] subunit alpha	IDH3A_HUMAN	40 kDa	17	26	19	6	0	1	2	4	5
UTP--glucose-1-phosphate uridylyltransferase	UGPA_HUMAN	57 kDa	4	2	5	10	40	3	8	8	0
Long-chain-fatty-acid--CoA ligase 1	ACSL1_HUMAN	78 kDa	0	0	12	25	35	0	5	3	0
Gamma-enolase	ENOG_HUMAN	47 kDa	19	59	0	0	0	0	0	0	0
ADP-ribosylation factor 5	ARF5_HUMAN	21 kDa	18	23	0	0	0	26	0	11	0
Unconventional myosin-Ic	MYO1C_HUMAN	122 kDa	0	0	0	38	0	0	27	13	0
Profilin-1	PROF1_HUMAN	15 kDa	10	0	8	5	12	8	8	10	17
Tenascin-R	TENR_HUMAN	150 kDa	47	27	3	0	0	0	0	0	0
Plasma membrane calcium-transporting ATPase 4	AT2B4_HUMAN	138 kDa	45	30	0	0	0	0	0	0	0
Puromycin-sensitive aminopeptidase	PSA_HUMAN	103 kDa	19	16	16	4	1	2	2	7	8
Heat shock 70 kDa protein 6	HSP76_HUMAN	71 kDa	0	0	9	13	11	22	10	0	10
Glutathione S-transferase P	GSTP1_HUMAN	23 kDa	6	10	5	10	8	7	9	11	8
Transketolase	TKT_HUMAN	68 kDa	12	12	0	8	8	16	1	8	8
Tubulin beta-6 chain	TBB6_HUMAN	50 kDa	62	0	11	0	0	0	0	0	0

Description	Accession	MW	Raw spectral counts								
			Frontal Cortex	Cerebellum	Right Ventricle	Mesentric lymph node	Liver	Pancreas	Proximal bile duct	Penis	Prostate
Myosin-13	MYH13_HUMAN	224 kDa	0	0	73	0	0	0	0	0	0
Phosphoglycerate mutase 1	PGAM1_HUMAN	29 kDa	8	15	7	8	3	6	7	10	8
Superoxide dismutase [Mn]	SODM_HUMAN	25 kDa	8	14	16	9	8	5	5	4	3
Eukaryotic translation initiation factor 5A-1	IF5A1_HUMAN	17 kDa	6	9	7	3	9	14	9	12	3
40S ribosomal protein S18	RS18_HUMAN	18 kDa	7	5	6	1	8	18	9	12	6
Myomesin-2	MYOM2_HUMAN	165 kDa	0	0	72	0	0	0	0	0	0
Nucleoside diphosphate kinase A	NDKA_HUMAN	17 kDa	16	11	12	2	8	6	0	12	4
GTP:AMP phosphotransferase AK3	KAD3_HUMAN	26 kDa	3	1	28	10	11	2	2	9	5
Protein disulfide-isomerase A2	PDIA2_HUMAN	58 kDa	0	0	0	0	0	71	0	0	0
Mimecan	MIME_HUMAN	34 kDa	3	0	4	16	0	0	40	8	0
Aspartate aminotransferase, cytoplasmic	AATC_HUMAN	46 kDa	11	23	14	0	6	6	0	6	4
ATP synthase subunit gamma	ATPG_HUMAN	33 kDa	16	12	18	3	7	5	3	6	0
Succinate dehydrogenase [ubiquinone] flavoprotein subunit	SDHA_HUMAN	73 kDa	7	6	32	0	11	10	0	2	2
Argininosuccinate synthase	ASSY_HUMAN	47 kDa	0	2	0	0	64	1	0	3	0
Visinin-like protein 1	VISL1_HUMAN	22 kDa	31	38	0	0	0	0	0	0	0
Syntaxin-1A	STX1A_HUMAN	33 kDa	56	13	0	0	0	0	0	0	0
Carbonic anhydrase 1	CAH1_HUMAN	29 kDa	8	13	10	6	10	2	4	12	4
Pyruvate carboxylase	PYC_HUMAN	130 kDa	3	5	1	20	29	0	11	0	0
Epoxide hydrolase 1	HYEP_HUMAN	53 kDa	0	0	0	12	31	17	2	0	7
Alpha-actinin-2	ACTN2_HUMAN	104 kDa	22	0	47	0	0	0	0	0	0
Synaptosomal-associated protein 25	SNP25_HUMAN	23 kDa	40	28	0	0	0	0	0	0	0
Putative heat shock protein HSP 90-beta-3	H90B3_HUMAN	68 kDa	12	16	10	0	0	18	12	0	0
Nucleoside diphosphate kinase B	NDKB_HUMAN	17 kDa	10	12	9	3	6	7	4	12	5
ATP synthase F(0) complex subunit B1	AT5F1_HUMAN	29 kDa	14	9	22	2	5	8	2	3	3
Electron transfer flavoprotein subunit alpha	ETFA_HUMAN	35 kDa	8	8	15	2	14	5	6	8	2
Keratin, type II cytoskeletal 1b	K2C1B_HUMAN	62 kDa	0	0	0	0	0	22	0	0	46
Calreticulin	CALR_HUMAN	48 kDa	3	0	5	3	10	26	2	8	11
Keratin, type II cytoskeletal 4	K2C4_HUMAN	57 kDa	4	0	18	0	0	32	0	11	3
Synaptotagmin-1	SYT1_HUMAN	48 kDa	40	27	0	0	0	0	0	0	0
Peptidyl-prolyl cis-trans isomerase A	PPIA_HUMAN	18 kDa	7	12	3	5	8	3	7	15	7
Transgelin-2	TAGL2_HUMAN	22 kDa	3	2	4	8	3	8	6	32	1
Plakophilin-1	PKP1_HUMAN	83 kDa	0	0	0	0	0	0	0	67	0
LIM domain-binding protein 3	LDB3_HUMAN	77 kDa	0	0	67	0	0	0	0	0	0
Beta-soluble NSF attachment protein	SNAB_HUMAN	34 kDa	28	35	0	0	0	0	0	3	0
Contactin-1	CNTN1_HUMAN	113 kDa	34	29	1	1	0	1	0	0	0
Neutral alpha-glucosidase AB	GANAB_HUMAN	107 kDa	5	4	2	2	15	23	2	8	5
60S acidic ribosomal protein P0	RLA0_HUMAN	34 kDa	7	3	6	0	6	35	2	6	1
Acetyl-CoA acetyltransferase	THIL_HUMAN	45 kDa	10	3	13	6	10	14	1	9	0
Glutathione S-transferase A1	GSTA1_HUMAN	26 kDa	1	0	0	0	56	6	0	2	1
Aldehyde oxidase	AOXA_HUMAN	148 kDa	0	0	1	4	57	2	1	1	0
Amine oxidase [flavin-containing] A	AOFA_HUMAN	60 kDa	3	0	7	8	9	23	3	0	13
Tropomyosin beta chain	TPM2_HUMAN	33 kDa	10	0	21	0	0	0	0	0	35
Neural cell adhesion molecule 1	NCAM1_HUMAN	95 kDa	35	27	2	0	0	0	0	0	1
Histone H2AX	H2AX_HUMAN	15 kDa	14	9	8	0	0	6	14	14	0
40S ribosomal protein S2	RS2_HUMAN	31 kDa	6	4	3	1	8	28	2	8	5
C-1-tetrahydrofolate synthase, cytoplasmic	C1TC_HUMAN	102 kDa	0	2	0	0	57	0	1	5	0
Haptoglobin	HPT_HUMAN	45 kDa	3	2	8	8	2	3	20	13	6
60S ribosomal protein L6	RL6_HUMAN	33 kDa	2	1	2	0	6	40	0	9	5
Isocitrate dehydrogenase [NADP] cytoplasmic	IDHC_HUMAN	47 kDa	0	0	0	16	19	7	11	9	2
Sarcoplasmic/endoplasmic reticulum calcium ATPase 1	AT2A1_HUMAN	110 kDa	0	0	57	0	0	0	0	0	7

Description	Accession	MW	Raw spectral counts								
			Frontal Cortex	Cerebellum	Right Ventricle	Mesentric lymph node	Liver	Pancreas	Proximal bile duct	Penis	Prostate
Guanine nucleotide-binding protein G(I)/G(S)/G(T) subunit beta-2	GBB2_HUMAN	37 kDa	17	29	1	1	2	3	4	2	4
Pyruvate dehydrogenase E1 component subunit beta	ODPB_HUMAN	39 kDa	11	18	26	0	2	0	1	5	0
Heterogeneous nuclear ribonucleoprotein A1	ROA1_HUMAN	39 kDa	4	17	3	2	1	6	6	16	8
Cytochrome b-c1 complex subunit 1	QCR1_HUMAN	53 kDa	5	14	19	2	4	12	2	4	1
Carbonyl reductase [NADPH] 1	CBR1_HUMAN	30 kDa	9	11	5	2	14	3	0	16	3
Ras-related protein Rab-1A	RAB1A_HUMAN	23 kDa	14	9	2	4	5	17	3	6	3
Cytoplasmic aconitate hydratase	ACOC_HUMAN	98 kDa	5	1	7	3	35	5	1	5	1
Betaine--homocysteine S-methyltransferase 1	BHMT1_HUMAN	45 kDa	0	0	0	0	63	0	0	0	0
AP-1 complex subunit beta-1	AP1B1_HUMAN	105 kDa	25	15	4	0	0	10	0	8	0
Cathepsin D	CATD_HUMAN	45 kDa	4	7	6	6	2	1	3	3	30
Flavin reductase (NADPH)	BLVRB_HUMAN	22 kDa	6	6	12	4	9	2	5	13	5
Alpha-soluble NSF attachment protein	SNAA_HUMAN	33 kDa	17	21	1	1	5	0	1	15	0
Nucleolin	NUCL_HUMAN	77 kDa	6	7	4	3	0	20	1	15	5
14-3-3 protein sigma	1433S_HUMAN	28 kDa	0	0	0	0	0	0	0	61	0
Hydroxyacyl-coenzyme A dehydrogenase	HCDH_HUMAN	34 kDa	2	0	25	8	10	4	6	3	3
Ras-related protein Rap-1b	RAP1B_HUMAN	21 kDa	5	9	4	9	5	8	7	6	7
V-type proton ATPase catalytic subunit A	VATA_HUMAN	68 kDa	29	22	0	0	1	3	0	3	1
Mitochondrial inner membrane protein	IMMT_HUMAN	84 kDa	8	12	25	2	2	5	1	1	3
Glutathione S-transferase A3	GSTA3_HUMAN	25 kDa	0	0	0	0	56	3	0	0	0
Ras-related protein Rab-2A	RAB2A_HUMAN	24 kDa	18	13	1	5	3	5	1	6	6
60S ribosomal protein L7a	RL7A_HUMAN	30 kDa	5	4	3	0	4	23	4	11	4
Cytochrome c	CYC_HUMAN	12 kDa	11	2	24	2	4	6	2	3	4
Potassium-transporting ATPase alpha chain 1	ATP4A_HUMAN	114 kDa	27	30	0	0	0	0	0	0	0
X-ray repair cross-complementing protein 5	XRCC5_HUMAN	83 kDa	4	13	1	1	2	13	1	19	3
ATP synthase subunit d	ATP5H_HUMAN	18 kDa	11	12	19	1	7	4	0	3	0
X-ray repair cross-complementing protein 6	XRCC6_HUMAN	70 kDa	4	9	1	0	2	10	2	21	8
Endoplasmic reticulum resident protein 29	ERP29_HUMAN	29 kDa	6	5	2	9	12	8	8	5	2
UDP-glucose:glycoprotein glucosyltransferase 1	UGGG1_HUMAN	177 kDa	0	0	0	1	1	45	1	4	5
Chymotrypsin-like elastase family member 3B	CEL3B_HUMAN	29 kDa	0	0	1	0	0	56	0	0	0
Extended synaptotagmin-1	ESYT1_HUMAN	123 kDa	4	0	4	22	6	2	9	7	3
Caveolin-1	CAV1_HUMAN	20 kDa	0	0	16	16	0	3	15	2	5
ATP-dependent 6-phosphofructokinase, muscle type	PFKAM_HUMAN	85 kDa	13	9	33	0	0	0	0	1	0
Protein DJ-1	PARK7_HUMAN	20 kDa	16	8	9	3	2	7	2	5	4
Reticulon-3	RTN3_HUMAN	113 kDa	21	5	6	6	3	6	4	2	3
Methylmalonate-semialdehyde dehydrogenase [acylating]	MMSA_HUMAN	58 kDa	3	1	12	1	16	16	2	0	5
Arginase-1	ARGI1_HUMAN	35 kDa	0	0	0	0	38	0	0	18	0
Prostatic acid phosphatase	PPAP_HUMAN	45 kDa	0	0	0	0	0	0	0	0	56
Protein-glutamine gamma-glutamyltransferase 4	TGM4_HUMAN	77 kDa	0	0	0	0	0	0	0	0	56
Ryanodine receptor 2	RYR2_HUMAN	565 kDa	1	0	55	0	0	0	0	0	0
Vacuolar protein sorting-associated protein 35	VPS35_HUMAN	92 kDa	18	11	3	4	0	4	3	7	5
Cysteine and glycine-rich protein 1	CSRP1_HUMAN	21 kDa	7	5	0	3	1	2	6	4	27
Rho GDP-dissociation inhibitor 1	GDIR1_HUMAN	23 kDa	8	5	2	8	3	6	7	7	9
40S ribosomal protein S9	RS9_HUMAN	23 kDa	4	3	2	2	4	26	0	9	5
Carboxypeptidase B	CBPB1_HUMAN	47 kDa	0	0	0	0	0	55	0	0	0
60S ribosomal protein L3	RL3_HUMAN	46 kDa	1	0	0	2	0	39	0	13	0
Plasma membrane calcium-transporting ATPase 3	AT2B3_HUMAN	134 kDa	30	24	0	0	0	0	0	0	0
Guanine nucleotide-binding protein subunit beta-2-like 1	GBLP_HUMAN	35 kDa	3	4	2	0	4	20	0	17	4
Adenylate kinase isoenzyme 1	KAD1_HUMAN	22 kDa	18	9	18	0	0	1	0	5	2
Enoyl-CoA hydratase	ECHM_HUMAN	31 kDa	7	4	9	5	16	2	1	3	6
Alcohol dehydrogenase 4	ADH4_HUMAN	40 kDa	0	0	0	0	53	0	0	0	0

Description	Accession	MW	Raw spectral counts								
			Frontal Cortex	Cerebellum	Right Ventricle	Mesentric lymph node	Liver	Pancreas	Proximal bile duct	Penis	Prostate
Annexin A4	ANXA4_HUMAN	36 kDa	4	0	0	3	3	9	9	16	9
Myomesin-1	MYOM1_HUMAN	188 kDa	0	0	53	0	0	0	0	0	0
AP-2 complex subunit alpha-2	AP2A2_HUMAN	104 kDa	25	21	1	0	1	0	1	3	0
Glycine amidinotransferase	GATM_HUMAN	48 kDa	0	0	0	0	12	39	0	1	0
Retinal dehydrogenase 1	AL1A1_HUMAN	55 kDa	1	0	2	4	26	11	5	0	3
Troponin T, cardiac muscle	TNNT2_HUMAN	36 kDa	1	0	51	0	0	0	0	0	0
Inositol 1,4,5-trisphosphate receptor type 1	ITPR1_HUMAN	314 kDa	1	50	0	0	0	0	0	0	0
Chymotrypsin-like elastase family member 2A	CEL2A_HUMAN	29 kDa	0	0	0	0	0	51	0	0	0
Vigilin	VIGLN_HUMAN	141 kDa	1	0	0	1	2	39	0	8	0
Dolichyl-diphosphooligosaccharide-protein glycosyltransferase 48 kDa subunit	OST48_HUMAN	51 kDa	0	0	0	4	6	28	2	3	8
Apoptosis-inducing factor 1	AIFM1_HUMAN	67 kDa	4	0	22	0	10	14	0	0	1
2',3'-cyclic-nucleotide 3'-phosphodiesterase	CN37_HUMAN	48 kDa	25	20	2	0	0	0	0	3	0
Heterogeneous nuclear ribonucleoprotein A3	ROA3_HUMAN	40 kDa	6	11	0	0	0	3	1	20	9
V-type proton ATPase 116 kDa subunit a isoform 1	VPP1_HUMAN	96 kDa	40	10	0	0	0	0	0	0	0
Ferritin heavy chain	FRIH_HUMAN	21 kDa	7	8	2	5	11	3	7	2	5
Trypsin-2	TRY2_HUMAN	26 kDa	0	0	0	0	0	50	0	0	0
Potassium-transporting ATPase alpha chain 2	AT12A_HUMAN	116 kDa	25	24	0	0	0	0	0	0	0
Glutathione S-transferase Mu 3	GSTM3_HUMAN	27 kDa	10	23	6	0	0	4	0	3	3
Transgelin-3	TAGL3_HUMAN	22 kDa	28	21	0	0	0	0	0	0	0
Dihydropyrimidinase-related protein 1	DPYL1_HUMAN	62 kDa	29	20	0	0	0	0	0	0	0
Alpha-internexin	AINX_HUMAN	55 kDa	30	19	0	0	0	0	0	0	0
Neurofilament light polypeptide	NFL_HUMAN	62 kDa	29	17	0	0	0	0	0	0	3
V-type proton ATPase subunit B, brain isoform	VATB2_HUMAN	57 kDa	29	15	0	0	0	0	0	3	2
Guanine nucleotide-binding protein G(i) subunit alpha-2	GNAI2_HUMAN	40 kDa	22	12	3	6	0	0	6	0	0
Microtubule-associated protein 1A	MAP1A_HUMAN	305 kDa	34	10	5	0	0	0	0	0	0
Ras-related protein Rab-11B	RB11B_HUMAN	24 kDa	8	8	3	6	0	2	3	14	5
Heat shock protein 75 kDa	TRAP1_HUMAN	80 kDa	6	6	5	5	4	8	3	4	8
Elongation factor 1-gamma	EF1G_HUMAN	50 kDa	3	4	5	2	2	24	0	7	2
Trypsin-3	TRY3_HUMAN	33 kDa	0	2	0	10	0	37	0	0	0
2,4-dienoyl-CoA reductase	DECR_HUMAN	36 kDa	5	2	11	2	16	7	2	1	3
Epiplakin	EPIPL_HUMAN	556 kDa	0	0	0	0	0	0	0	49	0
Myosin regulatory light chain 2, ventricular/cardiac muscle isoform	MLRV_HUMAN	19 kDa	0	0	40	0	0	9	0	0	0
Myosin light chain 3	MYL3_HUMAN	22 kDa	0	0	49	0	0	0	0	0	0
Lamin-B2	LMNB2_HUMAN	68 kDa	5	19	1	3	0	6	0	7	7
Serine/threonine-protein phosphatase 2B catalytic subunit alpha isoform	PP2BA_HUMAN	59 kDa	28	17	0	0	0	0	0	1	2
High mobility group protein B1	HMGB1_HUMAN	25 kDa	3	8	2	6	3	9	5	7	5
40S ribosomal protein S4, X isoform	RS4X_HUMAN	30 kDa	7	6	3	3	4	15	2	6	2
Reticulon-4	RTN4_HUMAN	130 kDa	17	4	0	5	13	0	3	6	0
Cytochrome P450 2A13	CP2AD_HUMAN	57 kDa	0	0	0	0	48	0	0	0	0
Fructose-bisphosphate aldolase B	ALDOB_HUMAN	39 kDa	0	0	0	0	39	9	0	0	0
Antithrombin-III	ANT3_HUMAN	53 kDa	0	0	7	9	0	1	18	11	2
Heat shock 70 kDa protein 12A	HS12A_HUMAN	75 kDa	23	9	0	9	0	0	5	0	1
NADH dehydrogenase [ubiquinone] iron-sulfur protein 3	NDUS3_HUMAN	30 kDa	12	8	18	0	2	2	0	5	0
Carbonic anhydrase 2	CAH2_HUMAN	29 kDa	10	7	8	0	3	7	3	8	1
Serine/threonine-protein phosphatase 2A 65 kDa regulatory subunit A	2AAA_HUMAN	65 kDa	18	6	13	1	0	2	0	6	1
40S ribosomal protein S8	RS8_HUMAN	24 kDa	2	3	0	0	3	25	1	9	4
40S ribosomal protein S7	RS7_HUMAN	22 kDa	4	1	3	1	9	20	2	7	0
Aldo-keto reductase family 1 member B10	AK1BA_HUMAN	36 kDa	3	0	6	21	0	2	5	6	4
Junction plakoglobin	PLAK_HUMAN	82 kDa	0	0	13	0	0	5	0	29	0
Neuronal membrane glycoprotein M6-a	GPM6A_HUMAN	31 kDa	22	24	0	0	0	0	0	0	0

Description	Accession	MW	Raw spectral counts								
			Frontal Cortex	Cerebellum	Right Ventricle	Mesentric lymph node	Liver	Pancreas	Proximal bile duct	Penis	Prostate
Myelin proteolipid protein	MYPR_HUMAN	30 kDa	28	14	4	0	0	0	0	0	0
Dynactin subunit 1	DCTN1_HUMAN	142 kDa	16	6	9	0	0	5	1	9	0
Cytochrome b-c1 complex subunit Rieske	UCRI_HUMAN	30 kDa	7	6	22	3	3	2	0	3	0
60S ribosomal protein L18	RL18_HUMAN	22 kDa	4	3	4	0	6	17	0	8	4
Hypoxia up-regulated protein 1	HYOU1_HUMAN	111 kDa	6	1	0	0	6	23	2	7	1
Transmembrane emp24 domain-containing protein 10	TMEDA_HUMAN	25 kDa	2	1	2	3	9	14	1	5	9
Fructose-1,6-bisphosphatase 1	F16P1_HUMAN	37 kDa	0	0	0	0	43	3	0	0	0
Tenascin-X	TENX_HUMAN	464 kDa	0	0	0	3	0	0	3	40	0
Dihydropyrimidinase-related protein 3	DPYL3_HUMAN	62 kDa	16	0	0	0	0	0	0	14	16
Ig lambda-3 chain C regions	LAC3_HUMAN	11 kDa	0	0	7	14	1	0	18	5	1
Alpha-adducin	ADDA_HUMAN	81 kDa	13	29	1	1	0	0	1	0	0
Elongation factor Tu	EFTU_HUMAN	50 kDa	5	16	11	4	0	4	0	5	0
40S ribosomal protein S13	RS13_HUMAN	17 kDa	6	5	3	0	3	7	7	11	3
60S ribosomal protein L9	RL9_HUMAN	22 kDa	5	2	4	0	3	22	1	8	0
Periplakin	PEPL_HUMAN	205 kDa	0	0	0	0	0	0	1	44	0
Collagen alpha-1(VI) chain	CO6A1_HUMAN	109 kDa	0	0	6	3	0	0	5	29	2
Calbindin	CALB1_HUMAN	30 kDa	8	36	0	0	0	0	0	0	0
Pyruvate dehydrogenase E1 component subunit alpha, somatic form	ODPA_HUMAN	43 kDa	12	13	15	2	1	1	0	0	0
Eukaryotic initiation factor 4A-II	IF4A2_HUMAN	46 kDa	5	5	2	2	2	12	0	11	5
Inactive pancreatic lipase-related protein 1	LIPR1_HUMAN	52 kDa	0	0	0	0	0	44	0	0	0
Sarcalumenin	SRCA_HUMAN	101 kDa	0	0	44	0	0	0	0	0	0
Neurofilament medium polypeptide	NFM_HUMAN	102 kDa	24	19	0	0	0	0	0	0	0
Vesicle-associated membrane protein 2	VAMP2_HUMAN	13 kDa	23	15	3	0	0	0	0	2	0
Fumarate hydratase	FUMH_HUMAN	55 kDa	8	5	16	1	7	1	0	2	3
40S ribosomal protein S10	RS10_HUMAN	19 kDa	2	4	1	3	7	12	3	9	2
Glycogen phosphorylase, liver form	PYGL_HUMAN	97 kDa	0	0	0	0	43	0	0	0	0
Keratin, type I cytoskeletal 17	K1C17_HUMAN	48 kDa	0	0	0	0	0	29	14	0	0
Ezrin	EZRI_HUMAN	69 kDa	4	0	7	0	0	9	0	14	9
Dystrophin	DMD_HUMAN	427 kDa	0	0	39	1	0	0	0	0	3
60S ribosomal protein L7	RL7_HUMAN	29 kDa	3	2	3	2	2	12	3	11	4
Alcohol dehydrogenase [NADP(+)]	AK1A1_HUMAN	37 kDa	3	1	2	0	6	16	2	5	7
4-hydroxyphenylpyruvate dioxygenase	HPPD_HUMAN	45 kDa	0	0	0	0	42	0	0	0	0
Cytochrome P450 2A6	CP2A6_HUMAN	57 kDa	0	0	0	0	42	0	0	0	0
Bifunctional glutamate/proline--tRNA ligase	SYEP_HUMAN	171 kDa	0	0	0	0	0	31	0	11	0
Dynamin-3	DYN3_HUMAN	98 kDa	17	23	0	0	0	0	0	1	0
Band 4.1-like protein 3	E41L3_HUMAN	121 kDa	20	21	0	0	0	0	0	0	0
Calmodulin	CALM_HUMAN	17 kDa	12	8	3	3	3	5	5	1	1
Interleukin enhancer-binding factor 2	ILF2_HUMAN	43 kDa	6	7	2	3	1	8	2	7	5
Poly(rC)-binding protein 1	PCBP1_HUMAN	37 kDa	8	4	4	1	0	10	1	11	2
40S ribosomal protein S14	RS14_HUMAN	16 kDa	7	3	7	2	3	11	4	3	1
Glycogen debranching enzyme	GDE_HUMAN	175 kDa	8	3	14	0	13	1	0	2	0
Amine oxidase [flavin-containing] B	AOFB_HUMAN	59 kDa	7	2	2	0	5	17	2	2	4
Myosin regulatory light chain 12B	ML12B_HUMAN	20 kDa	8	0	0	5	2	9	3	14	0
Calcium-binding mitochondrial carrier protein Aralar2	CMC2_HUMAN	74 kDa	5	0	6	0	27	2	0	1	0
Catenin alpha-1	CTNA1_HUMAN	100 kDa	1	0	7	0	1	10	0	19	3
V-type proton ATPase subunit E 1	VATE1_HUMAN	26 kDa	16	20	0	0	0	0	0	4	0
AP-2 complex subunit alpha-1	AP2A1_HUMAN	108 kDa	23	17	0	0	0	0	0	0	0
Synaptic vesicle glycoprotein 2A	SV2A_HUMAN	83 kDa	26	14	0	0	0	0	0	0	0
Cell division control protein 42 homolog	CDC42_HUMAN	21 kDa	7	5	2	1	5	7	4	4	5
Alpha-crystallin B chain	CRYAB_HUMAN	20 kDa	6	4	19	7	0	0	0	4	0

Description	Accession	MW	Raw spectral counts								
			Frontal Cortex	Cerebellum	Right Ventricle	Mesentric lymph node	Liver	Pancreas	Proximal bile duct	Penis	Prostate
60S ribosomal protein L26	RL26_HUMAN	17 kDa	4	3	0	3	1	21	1	6	1
40S ribosomal protein S3a	RS3A_HUMAN	30 kDa	3	3	4	0	1	17	1	9	2
Omega-amidase NIT2	NIT2_HUMAN	31 kDa	3	0	4	3	13	2	1	9	5
Neurofilament heavy polypeptide	NFH_HUMAN	112 kDa	21	18	0	0	0	0	0	0	0
Heterogeneous nuclear ribonucleoprotein U	HNRPU_HUMAN	91 kDa	7	12	4	0	0	5	0	11	0
Heterogeneous nuclear ribonucleoprotein D0	HNRPD_HUMAN	38 kDa	4	10	1	1	0	5	1	10	7
NADH dehydrogenase [ubiquinone] iron-sulfur protein 2	NDUS2_HUMAN	53 kDa	8	7	21	0	0	3	0	0	0
Calcium/calmodulin-dependent protein kinase type II subunit alpha	KCC2A_HUMAN	54 kDa	27	6	3	0	0	0	0	1	2
Heterogeneous nuclear ribonucleoproteins C1/C2	HNRPC_HUMAN	34 kDa	13	5	2	0	0	3	1	12	3
Myosin regulatory light polypeptide 9	MYL9_HUMAN	20 kDa	6	1	0	6	0	6	6	14	0
40S ribosomal protein S16	RS16_HUMAN	16 kDa	3	1	0	2	6	13	3	10	1
Myosin light polypeptide 6	MYL6_HUMAN	17 kDa	5	1	3	3	0	5	8	11	3
Redox-regulatory protein FAM213A	F213A_HUMAN	26 kDa	6	0	1	12	3	4	8	3	2
Alpha-2-macroglobulin	A2MG_HUMAN	163 kDa	2	0	3	10	0	1	17	6	0
Elongation factor 1-delta	EF1D_HUMAN	31 kDa	0	0	3	3	4	9	4	13	3
Integrin beta-1	ITB1_HUMAN	88 kDa	0	0	3	14	1	4	6	3	8
Septin-7	SEPT7_HUMAN	51 kDa	14	16	0	3	0	0	0	2	3
Transaldolase	TALDO_HUMAN	38 kDa	4	9	1	4	5	4	4	4	3
Vesicle-trafficking protein SEC22b	SC22B_HUMAN	25 kDa	5	9	2	8	2	4	2	3	3
Actin-related protein 2/3 complex subunit 2	ARPC2_HUMAN	34 kDa	13	8	1	2	1	4	0	9	0
Importin subunit beta-1	IMB1_HUMAN	97 kDa	8	6	6	4	3	1	1	7	2
Peroxiredoxin-4	PRDX4_HUMAN	31 kDa	0	3	0	5	5	15	0	7	3
3-hydroxyisobutyrate dehydrogenase	3HIDH_HUMAN	35 kDa	3	3	14	0	8	8	0	0	2
Dolichyl-diphosphooligosaccharide--protein glycosyltransferase subunit STT3A	STT3A_HUMAN	81 kDa	0	0	0	0	1	37	0	0	0
Coatomer subunit gamma-1	COPG1_HUMAN	98 kDa	1	0	0	1	5	17	1	5	8
Major vault protein	MVP_HUMAN	99 kDa	0	0	0	9	2	13	1	3	10
Protein bassoon	BSN_HUMAN	416 kDa	17	20	0	0	0	0	0	0	0
Cofilin-2	COF2_HUMAN	19 kDa	6	12	7	0	8	0	0	0	4
Guanine nucleotide-binding protein G(i) subunit alpha-1	GNAI1_HUMAN	40 kDa	27	10	0	0	0	0	0	0	0
40S ribosomal protein S6	RS6_HUMAN	29 kDa	1	4	2	0	4	20	0	4	2
Proteasome subunit alpha type-6	PSA6_HUMAN	27 kDa	8	4	5	2	0	3	2	9	4
Proteasome subunit alpha type-7	PSA7_HUMAN	28 kDa	8	3	6	3	2	5	0	7	3
T-complex protein 1 subunit beta	TCPB_HUMAN	57 kDa	14	3	7	2	0	0	1	10	0
Glutathione S-transferase omega-1	GSTO1_HUMAN	28 kDa	5	2	2	2	19	0	1	3	3
17-beta-hydroxysteroid dehydrogenase type 6	H17B6_HUMAN	36 kDa	1	0	0	0	34	0	0	0	2
Coatomer subunit beta'	COPB2_HUMAN	102 kDa	0	0	0	0	0	28	0	3	6
Keratin, type II cytoskeletal 71	K2C71_HUMAN	57 kDa	3	0	4	6	3	0	0	7	14
Medium-chain specific acyl-CoA dehydrogenase	ACADM_HUMAN	47 kDa	0	0	9	8	9	8	1	2	0
Adenylate kinase 2	KAD2_HUMAN	26 kDa	0	0	10	5	7	6	3	6	0
Microtubule-associated protein tau	TAU_HUMAN	79 kDa	17	19	0	0	0	0	0	0	0
Dynamin-1-like protein	DNM1L_HUMAN	82 kDa	15	13	4	0	0	0	0	4	0
Heterogeneous nuclear ribonucleoprotein M	HNRPM_HUMAN	78 kDa	6	11	0	1	0	1	1	16	0
B-cell receptor-associated protein 31	BAP31_HUMAN	28 kDa	7	2	3	8	1	4	2	5	4
D-3-phosphoglycerate dehydrogenase	SERA_HUMAN	57 kDa	4	1	0	1	4	18	1	7	0
Asporin	ASPN_HUMAN	43 kDa	0	0	0	19	0	0	14	3	0
Caldesmon	CALD1_HUMAN	93 kDa	0	0	0	1	0	0	0	0	35
Troponin I, cardiac muscle	TNNI3_HUMAN	24 kDa	0	0	36	0	0	0	0	0	0
Matrin-3	MATR3_HUMAN	95 kDa	10	16	2	0	0	0	0	6	1
Protein NDRG2	NDRG2_HUMAN	41 kDa	8	12	3	0	6	3	0	2	1
Vesicle-associated membrane protein-associated protein A	VAPA_HUMAN	28 kDa	8	11	4	3	1	1	1	4	2

Description	Accession	MW	Raw spectral counts								
			Frontal Cortex	Cerebellum	Right Ventricle	Mesentric lymph node	Liver	Pancreas	Proximal bile duct	Penis	Prostate
Cytoplasmic FMR1-interacting protein 2	CYFP2_HUMAN	148 kDa	18	10	0	0	0	0	0	7	0
Alpha-synuclein	SYUA_HUMAN	14 kDa	26	9	0	0	0	0	0	0	0
Calcium/calmodulin-dependent protein kinase type II subunit delta	KCC2D_HUMAN	56 kDa	12	9	7	0	0	0	0	2	5
Protein-L-isoaspartate(D-aspartate) O-methyltransferase	PIMT_HUMAN	25 kDa	14	6	9	0	0	0	0	3	3
UMP-CMP kinase	KCY_HUMAN	22 kDa	5	4	1	2	4	8	1	8	2
Hormone-sensitive lipase	LIPS_HUMAN	117 kDa	0	0	0	24	0	0	11	0	0
Galectin-7	LEG7_HUMAN	15 kDa	0	0	0	0	0	1	0	34	0
Eukaryotic translation initiation factor 3 subunit A	EIF3A_HUMAN	167 kDa	4	0	1	0	0	10	0	20	0
Ubiquitin thioesterase OTUB1	OTUB1_HUMAN	31 kDa	14	8	0	0	0	1	0	11	0
Nucleophosmin	NPM_HUMAN	33 kDa	4	6	0	0	0	16	0	7	1
ATP-citrate synthase	ACLY_HUMAN	121 kDa	8	4	0	6	0	9	2	4	1
GTP-binding protein SAR1a	SAR1A_HUMAN	22 kDa	4	4	2	3	7	6	3	4	1
Threonine--tRNA ligase, cytoplasmic	SYTC_HUMAN	83 kDa	2	3	0	0	0	17	0	2	10
40S ribosomal protein S19	RS19_HUMAN	16 kDa	4	3	4	0	4	9	0	8	2
6-phosphogluconate dehydrogenase, decarboxylating	6PGD_HUMAN	53 kDa	0	1	0	9	1	2	6	11	4
Creatine kinase S-type	KCRS_HUMAN	48 kDa	2	1	31	0	0	0	0	0	0
Zymogen granule membrane protein 16	ZG16_HUMAN	18 kDa	0	0	0	0	0	34	0	0	0
Protein transport protein Sec61 subunit alpha isoform 1	S61A1_HUMAN	52 kDa	0	0	0	0	0	34	0	0	0
Myosin light chain kinase, smooth muscle	MYLK_HUMAN	211 kDa	0	0	0	0	0	0	2	2	30
Synaptic vesicle membrane protein VAT-1 homolog	VAT1_HUMAN	42 kDa	0	0	1	5	0	7	5	5	11
40S ribosomal protein S25	RS25_HUMAN	14 kDa	5	0	2	0	6	8	1	8	4
ATP synthase subunit g	ATP5L_HUMAN	11 kDa	8	0	16	0	3	5	1	1	0
Calretinin	CALB2_HUMAN	32 kDa	9	24	0	0	0	0	0	0	0
Endophilin-A1	SH3G2_HUMAN	40 kDa	16	16	1	0	0	0	0	0	0
Hypoxanthine-guanine phosphoribosyltransferase	HPRT_HUMAN	25 kDa	10	9	3	0	0	4	0	6	1
Dynamin-like 120 kDa protein	OPA1_HUMAN	112 kDa	14	7	12	0	0	0	0	0	0
Actin-related protein 2/3 complex subunit 4	ARPC4_HUMAN	20 kDa	6	6	0	1	5	5	2	5	3
Ras-related protein Rab-5A	RAB5A_HUMAN	24 kDa	11	5	2	2	0	2	1	6	4
Obg-like ATPase 1	OLA1_HUMAN	45 kDa	6	2	7	0	0	7	0	8	3
Electron transfer flavoprotein-ubiquinone oxidoreductase	ETFD_HUMAN	68 kDa	5	2	11	0	6	9	0	0	0
Ornithine carbamoyltransferase	OTC_HUMAN	40 kDa	0	0	0	0	33	0	0	0	0
Serpin I2	SPI2_HUMAN	46 kDa	0	0	0	0	0	31	0	2	0
Serpin B5	SPB5_HUMAN	42 kDa	0	0	0	0	0	0	0	33	0
Plastin-2	PLSL_HUMAN	70 kDa	0	0	0	1	2	23	1	5	1
Proteasome activator complex subunit 1	PSME1_HUMAN	29 kDa	1	0	3	1	15	3	0	9	1
Creatine kinase U-type	KCRU_HUMAN	47 kDa	9	10	3	0	0	1	0	7	2
ES1 protein homolog	ES1_HUMAN	28 kDa	7	8	8	4	1	1	0	3	0
Succinyl-CoA ligase [ADP/GDP-forming] subunit alpha	SUCA_HUMAN	36 kDa	6	6	9	1	4	2	1	1	2
NADH dehydrogenase [ubiquinone] flavoprotein 2	NDUV2_HUMAN	27 kDa	6	6	10	0	3	3	0	4	0
Ras-related protein Rab-14	RAB14_HUMAN	24 kDa	10	5	0	2	1	2	2	6	4
Dihydrolipoyllysine	ODO2_HUMAN	49 kDa	4	5	13	3	0	2	1	1	3
Ras-related protein Rab-10	RAB10_HUMAN	23 kDa	7	4	3	3	0	3	1	8	3
Quinone oxidoreductase	QOR_HUMAN	35 kDa	3	3	3	0	12	5	0	3	3
Polyadenylate-binding protein 1	PABP1_HUMAN	71 kDa	1	2	2	0	1	11	0	11	4
60S ribosomal protein L11	RL11_HUMAN	20 kDa	2	2	3	2	5	7	4	4	3
Aldehyde dehydrogenase X	AL1B1_HUMAN	57 kDa	0	0	0	6	24	0	2	0	0
Eukaryotic initiation factor 4A-I	IF4A1_HUMAN	46 kDa	0	0	0	0	0	15	0	17	0
Clathrin coat assembly protein AP180	AP180_HUMAN	93 kDa	15	16	0	0	0	0	0	0	0
Ras-related protein Rab-5C	RAB5C_HUMAN	23 kDa	9	7	0	4	1	0	0	5	5
Adipocyte plasma membrane-associated protein	APMAP_HUMAN	46 kDa	2	3	0	9	3	2	7	3	2

Description	Accession	MW	Raw spectral counts								
			Frontal Cortex	Cerebellum	Right Ventricle	Mesentric lymph node	Liver	Pancreas	Proximal bile duct	Penis	Prostate
T-complex protein 1 subunit theta	TCPQ_HUMAN	60 kDa	15	3	1	1	0	0	0	11	0
Leucine-rich PPR motif-containing protein	LPPRC_HUMAN	158 kDa	6	2	14	0	0	2	0	7	0
60S ribosomal protein L5	RL5_HUMAN	34 kDa	0	0	0	0	2	22	0	7	0
Myelin P2 protein	MYP2_HUMAN	15 kDa	0	0	4	10	0	0	14	3	0
Propionyl-CoA carboxylase alpha chain	PCCA_HUMAN	80 kDa	0	0	5	1	21	4	0	0	0
Succinyl-CoA ligase [GDP-forming] subunit beta	SUCB2_HUMAN	47 kDa	0	0	9	1	11	2	2	6	0
Sodium/potassium-transporting ATPase subunit beta-1	AT1B1_HUMAN	35 kDa	16	12	2	0	0	0	0	0	0
Calcium/calmodulin-dependent protein kinase type II subunit beta	KCC2B_HUMAN	73 kDa	16	10	4	0	0	0	0	0	0
Vesicle-associated membrane protein-associated protein B/C	VAPB_HUMAN	27 kDa	7	7	4	4	4	0	0	4	0
Ubiquitin-conjugating enzyme E2 N	UBE2N_HUMAN	17 kDa	8	6	4	2	1	4	0	4	1
Dihydropteridine reductase	DHPR_HUMAN	26 kDa	6	5	3	3	6	1	2	2	2
Elongation factor 1-beta	EF1B_HUMAN	25 kDa	4	3	1	2	3	8	1	6	2
Proteasome subunit alpha type-5	PSA5_HUMAN	26 kDa	3	3	4	0	4	2	0	11	3
Programmed cell death 6-interacting protein	PDC6I_HUMAN	96 kDa	6	2	5	1	0	1	2	11	2
Proteasome subunit beta type-1	PSB1_HUMAN	26 kDa	6	1	2	2	2	6	1	8	2
Nebulette	NEBL_HUMAN	116 kDa	1	1	28	0	0	0	0	0	0
Xanthine dehydrogenase/oxidase	XDH_HUMAN	146 kDa	0	0	0	0	10	20	0	0	0
Sulfotransferase 1A1	ST1A1_HUMAN	34 kDa	0	0	0	1	23	0	3	3	0
Translocon-associated protein subunit alpha	SSRA_HUMAN	32 kDa	0	0	1	1	3	19	0	3	3
Polypyrimidine tract-binding protein 1	PTBP1_HUMAN	57 kDa	1	0	1	1	0	16	0	7	4
Phosphoglucomutase-like protein 5	PGM5_HUMAN	62 kDa	0	0	10	0	0	0	0	0	20
Immunoglobulin superfamily member 8	IGSF8_HUMAN	65 kDa	10	18	1	0	0	0	0	0	0
Calcium-dependent secretion activator 1	CAPS1_HUMAN	153 kDa	17	12	0	0	0	0	0	0	0
Splicing factor, proline- and glutamine-rich	SFPQ_HUMAN	76 kDa	4	11	2	0	2	2	0	4	4
Solute carrier family 12 member 5	S12A5_HUMAN	126 kDa	19	9	1	0	0	0	0	0	0
Ras-related protein Rab-3A	RAB3A_HUMAN	25 kDa	23	6	0	0	0	0	0	0	0
Actin-related protein 3	ARP3_HUMAN	47 kDa	2	5	0	2	0	6	2	8	4
Thy-1 membrane glycoprotein	THY1_HUMAN	18 kDa	11	4	0	0	0	1	6	3	4
26S proteasome non-ATPase regulatory subunit 2	PSMD2_HUMAN	100 kDa	2	4	5	2	4	3	0	7	2
Proteasome subunit beta type-2	PSB2_HUMAN	23 kDa	3	3	1	3	6	3	2	5	3
T-complex protein 1 subunit delta	TCPD_HUMAN	58 kDa	11	3	2	1	0	0	0	10	2
Proteasome subunit beta type-3	PSB3_HUMAN	23 kDa	5	3	3	1	5	4	0	6	2
Lon protease homolog	LONM_HUMAN	106 kDa	5	3	10	0	7	1	0	3	0
Extracellular superoxide dismutase [Cu-Zn]	SODE_HUMAN	26 kDa	0	2	0	8	0	1	14	3	1
60S ribosomal protein L8	RL8_HUMAN	28 kDa	1	2	3	1	1	13	2	5	1
Proliferation-associated protein 2G4	PA2G4_HUMAN	44 kDa	0	1	0	0	0	13	2	13	0
60S ribosomal protein L13	RL13_HUMAN	24 kDa	2	1	3	1	3	13	0	3	3
Fumarylacetoacetase	FAAA_HUMAN	46 kDa	0	0	0	5	19	0	5	0	0
Calponin-1	CNN1_HUMAN	33 kDa	0	0	0	0	0	0	6	2	21
Prostate-specific antigen	KLK3_HUMAN	29 kDa	0	0	0	0	0	0	0	0	29
Ras-related protein Rab-1B	RAB1B_HUMAN	22 kDa	11	0	3	5	0	0	6	0	4
Four and a half LIM domains protein 1	FHL1_HUMAN	36 kDa	2	0	3	4	0	2	6	4	8
Neurofascin	NFASC_HUMAN	150 kDa	16	12	0	0	0	0	0	0	0
Sideroflexin-3	SFXN3_HUMAN	36 kDa	12	10	0	0	1	0	0	5	0
BTB/POZ domain-containing protein KCTD12	KCD12_HUMAN	36 kDa	6	10	0	3	0	0	2	7	0
ATP-dependent 6-phosphofructokinase, platelet type	PFKAP_HUMAN	86 kDa	16	6	5	0	0	0	1	0	0
Microtubule-actin cross-linking factor 1, isoforms 1/2/3/5	MACF1_HUMAN	838 kDa	5	5	0	0	0	0	0	18	0
Ras-related C3 botulinum toxin substrate 1	RAC1_HUMAN	21 kDa	6	5	1	2	3	3	3	5	0
Alpha-centractin	ACTZ_HUMAN	43 kDa	10	3	3	3	0	1	2	6	0
Calpain-1 catalytic subunit	CAN1_HUMAN	82 kDa	2	3	3	0	1	6	3	10	0

Description	Accession	MW	Raw spectral counts								
			Frontal Cortex	Cerebellum	Right Ventricle	Mesentric lymph node	Liver	Pancreas	Proximal bile duct	Penis	Prostate
Destrin	DEST_HUMAN	19 kDa	6	2	2	0	3	5	2	3	5
Alanine--tRNA ligase, cytoplasmic	SYAC_HUMAN	107 kDa	9	1	0	0	1	13	0	2	2
Peroxisomal multifunctional enzyme type 2	DHB4_HUMAN	80 kDa	0	1	1	2	19	0	0	2	3
Mitochondrial 10-formyltetrahydrofolate dehydrogenase	AL1L2_HUMAN	102 kDa	0	0	0	0	9	19	0	0	0
Phosphoserine aminotransferase	SERC_HUMAN	40 kDa	4	0	0	0	8	7	0	9	0
Septin-2	SEPT2_HUMAN	41 kDa	9	0	2	4	0	1	2	7	3
Ceruloplasmin	CERU_HUMAN	122 kDa	0	0	7	2	2	0	12	4	1
GTP-binding nuclear protein Ran	RAN_HUMAN	24 kDa	3	12	2	1	0	2	1	5	1
4-aminobutyrate aminotransferase	GABT_HUMAN	56 kDa	4	10	0	0	12	1	0	0	0
Ankyrin-3	ANK3_HUMAN	480 kDa	16	10	0	0	0	0	0	1	0
Heterogeneous nuclear ribonucleoprotein R	HNRPR_HUMAN	71 kDa	2	9	0	1	0	7	0	6	2
Ubiquitin carboxyl-terminal hydrolase isozyme L1	UCHL1_HUMAN	25 kDa	8	9	2	3	0	0	0	2	3
Peripherin	PERI_HUMAN	54 kDa	0	7	0	0	0	0	0	0	20
NADH dehydrogenase [ubiquinone] 1 alpha subcomplex subunit 10	NDUAA_HUMAN	41 kDa	5	6	9	0	2	2	0	3	0
Ras-related protein Rab-35	RAB35_HUMAN	23 kDa	12	5	0	0	3	0	0	4	3
Serine/threonine-protein phosphatase PP1-beta catalytic subunit	PP1B_HUMAN	37 kDa	8	4	3	0	0	2	0	4	6
T-complex protein 1 subunit zeta	TCPZ_HUMAN	58 kDa	7	3	2	2	0	5	0	8	0
60S ribosomal protein L12	RL12_HUMAN	18 kDa	2	2	0	0	6	9	3	2	3
ATP-dependent RNA helicase A	DHX9_HUMAN	141 kDa	1	2	2	0	1	9	0	11	1
Dimethylglycine dehydrogenase	M2GD_HUMAN	97 kDa	0	0	0	0	27	0	0	0	0
Cytosolic 10-formyltetrahydrofolate dehydrogenase	AL1L1_HUMAN	99 kDa	0	0	0	0	27	0	0	0	0
Radixin	RADI_HUMAN	69 kDa	0	0	0	0	4	7	0	8	8
Collagen alpha-2(VI) chain	CO6A2_HUMAN	109 kDa	0	0	1	3	0	1	2	18	2
Ras-related protein Rab-3D	RAB3D_HUMAN	24 kDa	12	0	1	0	0	8	0	1	5
Delta-aminolevulinic acid dehydratase	HEM2_HUMAN	36 kDa	0	0	2	2	14	4	1	2	2
Cytochrome b5	CYB5_HUMAN	15 kDa	1	0	3	5	11	2	2	3	0
Synaptotagmin-2	SYT2_HUMAN	47 kDa	10	16	0	0	0	0	0	0	0
Calcineurin subunit B type 1	CANB1_HUMAN	19 kDa	12	14	0	0	0	0	0	0	0
Mitogen-activated protein kinase 1	MK01_HUMAN	41 kDa	8	11	0	0	0	0	0	4	3
Brain acid soluble protein 1	BASP1_HUMAN	23 kDa	12	8	0	0	0	0	0	0	6
Apolipoprotein E	APOE_HUMAN	36 kDa	4	7	1	5	1	0	6	2	0
Isocitrate dehydrogenase [NAD] subunit beta	IDH3B_HUMAN	42 kDa	11	7	8	0	0	0	0	0	0
Ras-related protein Rab-5B	RAB5B_HUMAN	24 kDa	10	5	0	0	0	0	0	6	5
Stress-induced-phosphoprotein 1	STIP1_HUMAN	63 kDa	5	5	4	0	1	6	0	3	2
Dihydrolipoyllysine-residue acetyltransferase component of pyruvate dehydrogenase	ODP2_HUMAN	69 kDa	6	5	13	1	0	1	0	0	0
Succinyl-CoA:3-ketoacid coenzyme A transferase 1	SCOT1_HUMAN	56 kDa	9	4	7	1	0	0	0	5	0
40S ribosomal protein S20	RS20_HUMAN	13 kDa	3	3	2	2	2	6	2	4	2
T-complex protein 1 subunit epsilon	TCPE_HUMAN	60 kDa	12	2	0	0	1	6	0	5	0
Proteasome subunit beta type-5	PSB5_HUMAN	28 kDa	5	2	5	0	4	4	0	6	0
Aldose reductase	ALDR_HUMAN	36 kDa	4	2	8	1	0	0	4	2	5
40S ribosomal protein S24	RS24_HUMAN	15 kDa	2	1	2	0	0	14	0	5	2
Ras GTPase-activating-like protein IQGAP2	IQGA2_HUMAN	181 kDa	0	0	0	0	17	9	0	0	0
Glutathione S-transferase A5	GSTA5_HUMAN	26 kDa	0	0	0	0	24	0	0	2	0
Arylacetamide deacetylase	AAAD_HUMAN	46 kDa	0	0	0	0	21	0	0	5	0
Heterogeneous nuclear ribonucleoprotein Q	HNRPQ_HUMAN	70 kDa	0	0	0	0	0	13	0	10	3
Chloride intracellular channel protein 4	CLIC4_HUMAN	29 kDa	2	0	9	3	0	2	1	4	5
Short-chain specific acyl-CoA dehydrogenase	ACADS_HUMAN	44 kDa	0	0	15	2	5	4	0	0	0
Myosin-7B	MYH7B_HUMAN	221 kDa	0	0	26	0	0	0	0	0	0
Reticulon-1	RTN1_HUMAN	84 kDa	12	13	0	0	0	0	0	0	0
Beta-synuclein	SYUB_HUMAN	14 kDa	16	9	0	0	0	0	0	0	0

Description	Accession	MW	Raw spectral counts								
			Frontal Cortex	Cerebellum	Right Ventricle	Mesentric lymph node	Liver	Pancreas	Proximal bile duct	Penis	Prostate
Histone H1.0	H10_HUMAN	21 kDa	3	6	2	3	1	4	2	4	0
Kinesin-1 heavy chain	KINH_HUMAN	110 kDa	8	4	2	0	0	5	0	5	1
Dual specificity protein phosphatase 3	DUS3_HUMAN	20 kDa	9	4	6	1	0	1	0	4	0
NADH dehydrogenase [ubiquinone] 1 alpha subcomplex subunit 13	NDUAD_HUMAN	17 kDa	7	4	12	0	0	1	0	1	0
Inorganic pyrophosphatase	IPYR_HUMAN	33 kDa	8	3	2	5	0	4	0	3	0
Ubiquitin-conjugating enzyme E2 L3	UB2L3_HUMAN	18 kDa	5	3	5	0	4	3	1	2	2
Enoyl-CoA delta isomerase 1	ECI1_HUMAN	33 kDa	4	3	6	1	7	1	0	3	0
Proteasome subunit alpha type-1	PSA1_HUMAN	30 kDa	6	2	4	1	3	1	0	6	2
60S ribosomal protein L15	RL15_HUMAN	24 kDa	1	1	1	0	1	16	0	4	1
Prenylcysteine oxidase 1	PCYOX_HUMAN	57 kDa	3	1	2	5	2	2	4	2	4
Chymotrypsin-like elastase family member 2B	CEL2B_HUMAN	29 kDa	0	0	0	0	0	25	0	0	0
Translocon-associated protein subunit delta	SSRD_HUMAN	19 kDa	1	0	0	0	0	22	0	2	0
Leukocyte elastase inhibitor	ILEU_HUMAN	43 kDa	0	0	0	0	0	19	0	6	0
Ethylmalonyl-CoA decarboxylase	ECHD1_HUMAN	34 kDa	0	0	0	13	1	0	10	0	1
Gamma-glutamyl hydrolase	GGH_HUMAN	36 kDa	0	0	0	0	0	0	0	12	13
Thrombospondin-1	TSP1_HUMAN	129 kDa	0	0	0	0	0	0	0	0	25
N-acylethanolamine-hydrolyzing acid amidase	NAAA_HUMAN	40 kDa	0	0	0	0	0	0	0	0	25
Propionyl-CoA carboxylase beta chain	PCCB_HUMAN	58 kDa	1	0	11	0	6	6	0	0	1
Myristoylated alanine-rich C-kinase substrate	MARCS_HUMAN	32 kDa	6	17	0	0	0	1	0	0	0
Phosphoglycerate mutase 2	PGAM2_HUMAN	29 kDa	6	8	10	0	0	0	0	0	0
Mitochondrial carrier homolog 2	MTCH2_HUMAN	33 kDa	7	7	2	0	7	0	0	1	0
NADH dehydrogenase [ubiquinone] 1 alpha subcomplex subunit 9	NDUA9_HUMAN	43 kDa	4	6	8	0	1	3	0	2	0
NADH dehydrogenase [ubiquinone] flavoprotein 1	NDUV1_HUMAN	51 kDa	2	4	12	2	0	0	0	1	3
Poly(rC)-binding protein 2	PCBP2_HUMAN	39 kDa	6	3	0	0	0	5	0	10	0
Succinate dehydrogenase [ubiquinone] iron-sulfur subunit	SDHB_HUMAN	32 kDa	3	3	7	2	4	3	0	2	0
D-beta-hydroxybutyrate dehydrogenase	BDH_HUMAN	38 kDa	3	2	11	0	8	0	0	0	0
Prostaglandin E synthase 2	PGES2_HUMAN	42 kDa	5	2	13	0	1	0	0	2	1
60S ribosomal protein L27	RL27_HUMAN	16 kDa	3	1	1	0	3	10	1	4	1
Translationally-controlled tumor protein	TCTP_HUMAN	20 kDa	1	1	2	2	2	8	1	7	0
Phosphoenolpyruvate carboxykinase [GTP]	PCKGM_HUMAN	71 kDa	0	0	0	0	23	1	0	0	0
Serine--tRNA ligase, cytoplasmic	SYSC_HUMAN	59 kDa	3	0	0	0	0	13	0	8	0
Protein transport protein Sec31A	SC31A_HUMAN	133 kDa	1	0	0	0	1	18	0	2	2
NADPH--cytochrome P450 reductase	NCPR_HUMAN	77 kDa	0	0	1	3	12	1	0	6	1
4-trimethylaminobutyraldehyde dehydrogenase	AL9A1_HUMAN	54 kDa	2	0	3	5	5	2	0	2	5
Platelet glycoprotein 4	CD36_HUMAN	53 kDa	0	0	4	13	2	1	4	0	0
40S ribosomal protein S17-like	RS17L_HUMAN	16 kDa	3	0	4	0	2	7	0	8	0
Myoglobin	MYG_HUMAN	17 kDa	0	0	21	0	0	3	0	0	0
Troponin C, slow skeletal and cardiac muscles	TNNC1_HUMAN	18 kDa	0	0	24	0	0	0	0	0	0
AFG3-like protein 2	AFG32_HUMAN	89 kDa	5	7	11	0	0	0	0	0	0
Glycerol-3-phosphate dehydrogenase	GPDM_HUMAN	81 kDa	6	6	5	0	0	3	0	1	2
Membrane-associated progesterone receptor component 1	PGRC1_HUMAN	22 kDa	6	5	0	0	11	0	0	0	1
Serine/threonine-protein phosphatase 2A catalytic subunit alpha isoform	PP2AA_HUMAN	36 kDa	8	5	2	0	0	1	1	4	2
EH domain-containing protein 3	EHD3_HUMAN	61 kDa	16	3	2	2	0	0	0	0	0
60S ribosomal protein L10	RL10_HUMAN	25 kDa	2	2	2	0	1	10	0	6	0
NADH-cytochrome b5 reductase 3	NB5R3_HUMAN	34 kDa	2	2	2	3	2	2	3	6	1
S-formylglutathione hydrolase	ESTD_HUMAN	31 kDa	5	1	5	0	6	3	0	3	0
cAMP-dependent protein kinase type II-alpha regulatory subunit	KAP2_HUMAN	46 kDa	5	1	5	2	0	3	0	3	4
Proteasome subunit alpha type-2	PSA2_HUMAN	26 kDa	5	1	7	2	2	2	0	3	1
Aldo-keto reductase family 1 member C3	AK1C3_HUMAN	37 kDa	0	0	0	0	23	0	0	0	0
Glycine N-methyltransferase	GNMT_HUMAN	33 kDa	0	0	0	0	22	1	0	0	0

Description	Accession	MW	Raw spectral counts								
			Frontal Cortex	Cerebellum	Right Ventricle	Mesentric lymph node	Liver	Pancreas	Proximal bile duct	Penis	Prostate
Trypsin-1	TRY1_HUMAN	27 kDa	0	0	0	0	0	23	0	0	0
Plastin-3	PLST_HUMAN	71 kDa	0	0	0	0	8	3	0	12	0
Liver carboxylesterase 1	EST1_HUMAN	63 kDa	0	0	0	0	19	2	0	0	2
Protein kinase C delta-binding protein	PRDBP_HUMAN	28 kDa	0	0	1	10	0	0	6	6	0
Cathepsin B	CATB_HUMAN	38 kDa	3	0	1	0	2	0	0	3	14
60S ribosomal protein L13a	RL13A_HUMAN	24 kDa	2	0	2	2	3	11	0	1	2
Beta-adducin	ADDB_HUMAN	81 kDa	11	11	0	0	0	0	0	0	0
V-type proton ATPase subunit D	VATD_HUMAN	28 kDa	11	10	0	0	0	0	0	1	0
N(G),N(G)-dimethylarginine dimethylaminohydrolase 1	DDAH1_HUMAN	31 kDa	10	8	0	0	1	0	0	0	3
Septin-11	SEP11_HUMAN	49 kDa	10	7	0	2	0	2	0	0	1
Coiled-coil-helix-coiled-coil-helix domain-containing protein 3	CHCH3_HUMAN	26 kDa	5	4	10	1	0	2	0	0	0
Heat shock 70 kDa protein 4	HSP74_HUMAN	94 kDa	6	3	2	0	1	1	1	5	3
Microsomal glutathione S-transferase 3	MGST3_HUMAN	17 kDa	9	3	10	0	0	0	0	0	0
Heterogeneous nuclear ribonucleoprotein L	HNRPL_HUMAN	64 kDa	3	2	1	0	1	4	0	5	6
Keratin, type II cytoskeletal 3	K2C3_HUMAN	64 kDa	0	0	0	0	0	22	0	0	0
60S ribosomal protein L10a	RL10A_HUMAN	25 kDa	2	0	0	0	0	11	0	5	4
Eukaryotic translation initiation factor 5B	IF2P_HUMAN	139 kDa	0	0	0	0	0	12	0	5	5
Chloride intracellular channel protein 1	CLIC1_HUMAN	27 kDa	0	0	1	1	3	4	3	8	2
Talin-2	TLN2_HUMAN	272 kDa	12	0	2	8	0	0	0	0	0
Ketimine reductase mu-crystallin	CRYM_HUMAN	34 kDa	17	0	2	0	0	0	0	3	0
Thioredoxin	THIO_HUMAN	12 kDa	3	0	3	0	2	3	0	7	4
Cystatin-B	CYTB_HUMAN	11 kDa	4	0	4	0	0	5	0	7	2
Fatty acid-binding protein, heart	FABPH_HUMAN	15 kDa	8	0	13	1	0	0	0	0	0
Myomesin-3	MYOM3_HUMAN	162 kDa	0	0	22	0	0	0	0	0	0
Excitatory amino acid transporter 4	EAA4_HUMAN	62 kDa	0	21	0	0	0	0	0	0	0
Phosphatidylinositol-binding clathrin assembly protein	PICAL_HUMAN	71 kDa	8	11	0	0	0	0	1	1	0
Synaptophysin	SYPH_HUMAN	34 kDa	12	9	0	0	0	0	0	0	0
ATP-dependent RNA helicase DDX1	DDX1_HUMAN	82 kDa	3	8	2	0	1	2	0	3	2
Heterogeneous nuclear ribonucleoprotein D-like	HNRDL_HUMAN	46 kDa	6	7	0	0	0	0	0	8	0
Serine/arginine-rich splicing factor 1	SRSF1_HUMAN	28 kDa	3	7	0	0	0	2	0	7	2
Kinesin heavy chain isoform 5C	KIF5C_HUMAN	109 kDa	15	6	0	0	0	0	0	0	0
Neural cell adhesion molecule L1	L1CAM_HUMAN	140 kDa	10	6	0	0	0	0	0	0	5
F-actin-capping protein subunit beta	CAPZB_HUMAN	31 kDa	6	6	1	0	0	1	0	6	1
Neurochondrin	NCDN_HUMAN	79 kDa	16	5	0	0	0	0	0	0	0
Very-long-chain (3R)-3-hydroxyacyl-CoA dehydratase 3	HACD3_HUMAN	43 kDa	7	5	1	0	2	6	0	0	0
Biliverdin reductase A	BIEA_HUMAN	33 kDa	2	4	2	1	0	1	3	8	0
F-actin-capping protein subunit alpha-2	CAZA2_HUMAN	33 kDa	6	3	0	2	0	2	4	4	0
Aflatoxin B1 aldehyde reductase member 2	ARK72_HUMAN	40 kDa	1	3	1	5	4	2	3	2	0
60S ribosomal protein L22	RL22_HUMAN	15 kDa	4	3	2	0	3	4	0	3	2
60S ribosomal protein L27a	RL27A_HUMAN	17 kDa	2	2	0	0	3	9	0	3	2
Proteasome subunit alpha type-4	PSA4_HUMAN	29 kDa	5	2	4	0	3	1	0	5	1
Lactoylglutathione lyase	LGUL_HUMAN	21 kDa	8	1	3	0	3	1	0	4	1
Adenine phosphoribosyltransferase	APT_HUMAN	20 kDa	0	1	4	2	6	5	0	3	0
Kinectin	KTN1_HUMAN	156 kDa	1	1	5	0	0	5	0	4	5
Bifunctional ATP-dependent dihydroxyacetone kinase/FAD-AMP lyase	DHAK_HUMAN	59 kDa	0	0	0	0	21	0	0	0	0
Hydroxymethylglutaryl-CoA synthase	HMCS2_HUMAN	57 kDa	0	0	0	0	21	0	0	0	0
Serpin H1	SERPH_HUMAN	46 kDa	0	0	0	16	0	0	5	0	0
Histone H2A.Z	H2AZ_HUMAN	14 kDa	6	0	0	0	0	0	0	15	0
Ribonuclease UK114	UK114_HUMAN	14 kDa	7	0	0	0	10	3	0	0	1
Plasma protease C1 inhibitor	IC1_HUMAN	55 kDa	0	0	0	4	0	3	9	3	2

Description	Accession	MW	Raw spectral counts								
			Frontal Cortex	Cerebellum	Right Ventricle	Mesentric lymph node	Liver	Pancreas	Proximal bile duct	Penis	Prostate
Filaggrin-2	FILA2_HUMAN	248 kDa	0	0	0	0	0	0	0	19	2
1-phosphatidylinositol 4,5-bisphosphate phosphodiesterase beta-1	PLCB1_HUMAN	139 kDa	20	0	1	0	0	0	0	0	0
Insulin-degrading enzyme	IDE_HUMAN	118 kDa	0	0	1	0	0	2	0	18	0
D-dopachrome decarboxylase	DOPD_HUMAN	13 kDa	2	0	2	0	6	6	0	2	3
GTP-binding protein SAR1b	SAR1B_HUMAN	22 kDa	0	0	3	0	8	6	0	4	0
10 kDa heat shock protein	CH10_HUMAN	11 kDa	4	0	8	0	2	4	1	0	2
Spectrin beta chain, erythrocytic	SPTB1_HUMAN	246 kDa	0	16	0	0	0	0	0	4	0
EH domain-containing protein 1	EHD1_HUMAN	61 kDa	7	7	2	3	0	0	0	0	1
Nck-associated protein 1	NCKP1_HUMAN	129 kDa	11	6	0	0	0	0	0	3	0
Phospholipid hydroperoxide glutathione peroxidase	GPX4_HUMAN	22 kDa	5	5	3	1	3	1	1	0	1
Heterogeneous nuclear ribonucleoprotein H2	HNRH2_HUMAN	49 kDa	1	4	2	0	1	5	0	3	4
UPF0568 protein C14orf166	CN166_HUMAN	28 kDa	6	4	3	0	0	4	0	3	0
NADH dehydrogenase [ubiquinone] 1 beta subcomplex subunit 10	NDUBA_HUMAN	21 kDa	3	4	7	0	1	2	0	3	0
Long-chain-fatty-acid--CoA ligase 6	ACSL6_HUMAN	78 kDa	4	3	1	5	6	0	1	0	0
Proteasome subunit alpha type-3	PSA3_HUMAN	28 kDa	4	3	4	1	1	3	0	3	1
Erlin-2	ERLN2_HUMAN	38 kDa	1	2	0	1	0	7	2	7	0
L-xylulose reductase	DCXR_HUMAN	26 kDa	2	2	0	0	15	0	0	0	1
60S ribosomal protein L23a	RL23A_HUMAN	18 kDa	3	1	1	0	1	2	1	9	2
Mitochondrial amidoxime reducing component 2	MARC2_HUMAN	38 kDa	4	1	3	0	9	3	0	0	0
Peroxisomal bifunctional enzyme	ECHP_HUMAN	79 kDa	0	0	0	0	20	0	0	0	0
Sulfotransferase 1A2	ST1A2_HUMAN	34 kDa	0	0	0	0	20	0	0	0	0
Short/branched chain specific acyl-CoA dehydrogenase	ACDSB_HUMAN	47 kDa	0	0	0	0	20	0	0	0	0
Laminin subunit alpha-4	LAMA4_HUMAN	203 kDa	0	0	0	17	0	0	3	0	0
Regucalcin	RGN_HUMAN	33 kDa	2	0	0	0	7	11	0	0	0
Serpin B3	SPB3_HUMAN	45 kDa	0	0	0	0	0	4	0	16	0
Envoplakin	EVPL_HUMAN	232 kDa	0	0	0	0	0	0	0	20	0
Caspase-14	CASPE_HUMAN	28 kDa	0	0	0	0	0	0	0	19	1
Cell surface glycoprotein MUC18	MUC18_HUMAN	72 kDa	0	0	0	10	0	0	6	0	4
Beta-hexosaminidase subunit beta	HEXB_HUMAN	63 kDa	0	0	1	0	0	0	0	3	16
Peptidyl-prolyl cis-trans isomerase FKBP2	FKBP2_HUMAN	16 kDa	4	0	2	0	3	8	1	0	2
Vesicle-associated membrane protein 3	VAMP3_HUMAN	11 kDa	16	0	4	0	0	0	0	0	0
Myozenin-2	MYOZ2_HUMAN	30 kDa	0	0	20	0	0	0	0	0	0
Calsequestrin-2	CASQ2_HUMAN	46 kDa	0	0	20	0	0	0	0	0	0
Probable ATP-dependent RNA helicase DDX17	DDX17_HUMAN	80 kDa	3	11	0	0	0	2	0	3	0
Calcium/calmodulin-dependent protein kinase type II subunit gamma	KCC2G_HUMAN	63 kDa	11	8	0	0	0	0	0	0	0
Ras-related protein Rab-6A	RAB6A_HUMAN	24 kDa	7	7	0	0	0	0	0	5	0
Microtubule-associated protein RP/EB family member 3	MARE3_HUMAN	32 kDa	11	7	0	0	0	0	0	0	1
Sideroflexin-1	SFXN1_HUMAN	36 kDa	7	7	1	0	3	1	0	0	0
Hippocalcin-like protein 4	HPCL4_HUMAN	22 kDa	13	6	0	0	0	0	0	0	0
Transcriptional activator protein Pur-alpha	PURA_HUMAN	35 kDa	6	5	0	1	0	4	0	3	0
NADH dehydrogenase [ubiquinone] iron-sulfur protein 8	NDUS8_HUMAN	24 kDa	3	4	5	0	2	1	0	4	0
Actin-related protein 2	ARP2_HUMAN	45 kDa	5	3	0	2	0	2	1	5	1
Serine/threonine-protein phosphatase 2A activator	PTPA_HUMAN	41 kDa	6	3	0	0	2	3	0	3	2
Leukotriene A-4 hydrolase	LKHA4_HUMAN	69 kDa	2	3	1	0	0	9	0	2	2
Hsc70-interacting protein	F10A1_HUMAN	41 kDa	1	3	2	0	0	2	0	7	4
Acidic leucine-rich nuclear phosphoprotein 32 family member A	AN32A_HUMAN	29 kDa	2	3	3	0	2	2	0	4	3
Ras-related protein Rab-18	RAB18_HUMAN	23 kDa	4	3	4	2	3	0	0	3	0
F-actin-capping protein subunit alpha-1	CAZA1_HUMAN	33 kDa	5	2	0	2	0	3	3	4	0
Alpha-aminoadipic semialdehyde dehydrogenase	AL7A1_HUMAN	58 kDa	2	2	1	1	7	1	0	2	3
Protein transport protein Sec23B	SC23B_HUMAN	86 kDa	0	1	0	0	0	15	0	0	3

Description	Accession	MW	Raw spectral counts								
			Frontal Cortex	Cerebellum	Right Ventricle	Mesentric lymph node	Liver	Pancreas	Proximal bile duct	Penis	Prostate
Catenin beta-1	CTNB1_HUMAN	85 kDa	5	1	1	0	0	6	0	6	0
DNA-dependent protein kinase catalytic subunit	PRKDC_HUMAN	469 kDa	2	1	1	0	0	0	0	14	1
Glutathione S-transferase kappa 1	GSTK1_HUMAN	25 kDa	1	1	7	2	5	0	0	3	0
Serine hydroxymethyltransferase, cytosolic	GLYC_HUMAN	53 kDa	0	0	0	0	19	0	0	0	0
UDP-glucose 6-dehydrogenase	UGDH_HUMAN	55 kDa	0	0	0	0	7	0	0	12	0
Histone H1.5	H15_HUMAN	23 kDa	0	0	0	2	0	0	2	15	0
Sulfide:quinone oxidoreductase	SQRD_HUMAN	50 kDa	0	0	0	1	2	5	3	7	1
Signal peptidase complex subunit 2	SPCS2_HUMAN	25 kDa	0	0	0	0	2	14	0	1	2
Annexin A3	ANXA3_HUMAN	36 kDa	0	0	0	0	0	7	1	3	8
Gamma-glutamyltranspeptidase 1	GGT1_HUMAN	61 kDa	0	0	0	0	0	5	0	0	14
Isovaleryl-CoA dehydrogenase	IVD_HUMAN	46 kDa	2	0	3	0	9	5	0	0	0
Complement C4-A	CO4A_HUMAN	193 kDa	0	0	3	5	0	0	7	2	2
Ras-related protein R-Ras	RRAS_HUMAN	23 kDa	1	0	3	7	0	0	3	2	3
Serum deprivation-response protein	SDPR_HUMAN	47 kDa	0	0	4	12	0	0	2	0	1
Ras suppressor protein 1	RSU1_HUMAN	32 kDa	0	0	4	2	1	2	3	2	5
Heat shock protein beta-6	HSPB6_HUMAN	17 kDa	0	0	5	5	0	0	7	0	2
Delta(3,5)-Delta(2,4)-dienoyl-CoA isomerase	ECH1_HUMAN	36 kDa	0	0	16	0	3	0	0	0	0
Receptor-type tyrosine-protein phosphatase zeta	PTPRZ_HUMAN	255 kDa	10	7	1	0	0	0	0	0	0
Tubulin polymerization-promoting protein	TPPP_HUMAN	24 kDa	12	6	0	0	0	0	0	0	0
Sodium/calcium exchanger 2	NAC2_HUMAN	100 kDa	11	6	1	0	0	0	0	0	0
Ras-related protein Rab-6B	RAB6B_HUMAN	23 kDa	9	5	0	0	0	1	0	2	1
tRNA-splicing ligase RtcB homolog	RTCB_HUMAN	55 kDa	1	4	4	0	0	1	0	5	3
Lamin-B1	LMNB1_HUMAN	66 kDa	0	3	0	0	0	8	0	7	0
V-type proton ATPase subunit C 1	VATC1_HUMAN	44 kDa	13	3	0	0	0	1	0	0	1
Sorcin	SORCN_HUMAN	22 kDa	5	3	1	1	0	1	2	5	0
ATP-dependent RNA helicase DDX3X	DDX3X_HUMAN	73 kDa	3	3	1	0	0	1	0	9	1
Protein SET	SET_HUMAN	33 kDa	3	3	1	0	0	3	0	6	2
Cytochrome c oxidase subunit 4 isoform 1	COX41_HUMAN	20 kDa	3	3	6	0	3	2	0	1	0
Glycine--tRNA ligase	SYG_HUMAN	83 kDa	2	2	0	0	0	9	0	5	0
Lupus La protein	LA_HUMAN	47 kDa	0	2	0	0	0	5	0	8	3
Calpain small subunit 1	CPNS1_HUMAN	28 kDa	2	2	1	2	0	3	2	4	2
CDP-diacylglycerol--inositol 3-phosphatidyltransferase	CDIPT_HUMAN	24 kDa	1	2	4	0	0	11	0	0	0
WD repeat-containing protein 1	WDR1_HUMAN	66 kDa	3	2	4	0	0	1	1	2	5
Vesicular integral-membrane protein VIP36	LMAN2_HUMAN	40 kDa	0	1	1	0	4	8	0	0	4
60S ribosomal protein L24	RL24_HUMAN	18 kDa	0	1	2	0	1	6	2	5	1
Transmembrane protein 43	TMM43_HUMAN	45 kDa	0	0	0	7	0	3	2	6	0
Cytoplasmic FMR1-interacting protein 1	CYFP1_HUMAN	145 kDa	10	0	0	0	0	0	0	8	0
Serine protease inhibitor Kazal-type 5	ISK5_HUMAN	121 kDa	0	0	0	0	0	0	0	18	0
Alcohol dehydrogenase class 4 mu/sigma chain	ADH7_HUMAN	41 kDa	0	0	0	0	0	0	0	18	0
Heat shock-related 70 kDa protein 2	HSP72_HUMAN	70 kDa	0	0	0	0	0	0	0	18	0
Malectin	MLEC_HUMAN	32 kDa	0	0	0	0	6	9	0	1	2
Rho GTPase-activating protein 1	RHG01_HUMAN	50 kDa	3	0	0	1	0	2	0	9	3
Sorbitol dehydrogenase	DHSO_HUMAN	38 kDa	0	0	0	0	12	2	0	0	4
Coatomer subunit beta	COPB_HUMAN	107 kDa	1	0	0	0	2	4	0	5	6
Tenascin	TENA_HUMAN	241 kDa	6	0	0	0	0	0	0	0	12
Aldo-keto reductase family 1 member C4	AK1C4_HUMAN	37 kDa	0	0	3	0	15	0	0	0	0
Sepiapterin reductase	SPRE_HUMAN	28 kDa	3	0	4	2	4	3	0	0	2
CDGSH iron-sulfur domain-containing protein 1	CISD1_HUMAN	12 kDa	10	0	6	0	0	2	0	0	0
Methylmalonyl-CoA mutase	MUTA_HUMAN	83 kDa	0	0	6	0	10	2	0	0	0
Guanine nucleotide-binding protein G(q) subunit alpha	GNAQ_HUMAN	42 kDa	9	8	0	0	0	0	0	0	0

Description	Accession	MW	Raw spectral counts								
			Frontal Cortex	Cerebellum	Right Ventricle	Mesentric lymph node	Liver	Pancreas	Proximal bile duct	Penis	Prostate
ELAV-like protein 1	ELAV1_HUMAN	36 kDa	0	8	0	0	0	0	0	7	2
2-oxoglutarate dehydrogenase-like	OGDHL_HUMAN	114 kDa	10	7	0	0	0	0	0	0	0
Protein kinase C and casein kinase substrate in neurons protein 1	PACN1_HUMAN	51 kDa	11	6	0	0	0	0	0	0	0
GTPase NRas	RASN_HUMAN	21 kDa	6	6	0	1	0	0	0	4	0
Protein FAM49B	FA49B_HUMAN	37 kDa	9	5	0	0	0	0	0	3	0
Heterogeneous nuclear ribonucleoprotein H	HNRH1_HUMAN	49 kDa	0	5	0	1	0	7	0	4	0
T-complex protein 1 subunit eta	TCPH_HUMAN	59 kDa	9	4	0	0	0	0	0	4	0
S-adenosylmethionine synthase isoform type-2	METK2_HUMAN	44 kDa	1	2	0	0	5	5	0	2	2
Fumarylacetoacetate hydrolase domain-containing protein 2A	FAH2A_HUMAN	35 kDa	3	2	1	2	3	3	1	2	0
LETM1 and EF-hand domain-containing protein 1	LETM1_HUMAN	83 kDa	8	2	4	0	0	0	0	3	0
Purine nucleoside phosphorylase	PNPH_HUMAN	32 kDa	2	1	0	0	8	0	0	6	0
Thiosulfate sulfurtransferase	THTR_HUMAN	33 kDa	1	1	1	1	9	1	0	2	1
Acetyl-CoA carboxylase 2	ACACB_HUMAN	277 kDa	0	0	0	11	5	0	1	0	0
Nodal modulator 1	NOMO1_HUMAN	134 kDa	0	0	0	0	1	16	0	0	0
Annexin A8	ANXA8_HUMAN	37 kDa	0	0	0	0	0	0	0	17	0
Annexin A8-like protein 2	AXA82_HUMAN	37 kDa	0	0	0	0	0	0	0	17	0
Early endosome antigen 1	EEA1_HUMAN	162 kDa	5	0	0	1	0	4	0	6	1
60S ribosomal protein L4	RL4_HUMAN	48 kDa	0	0	0	0	1	9	0	6	1
Tumor protein D52	TPD52_HUMAN	24 kDa	4	0	0	0	0	3	0	8	2
Adenylyl cyclase-associated protein 1	CAP1_HUMAN	52 kDa	2	0	0	2	0	2	2	6	3
Sialic acid synthase	SIAS_HUMAN	40 kDa	1	0	0	0	0	4	0	1	11
3-ketoacyl-CoA thiolase, peroxisomal	THIK_HUMAN	44 kDa	1	0	1	0	14	0	0	1	0
60S ribosomal protein L14	RL14_HUMAN	23 kDa	2	0	2	0	1	3	0	7	2
Dehydrogenase/reductase SDR family member 4	DHRS4_HUMAN	30 kDa	0	0	4	1	11	0	0	1	0
Fibrinogen beta chain	FIBB_HUMAN	56 kDa	0	0	5	3	0	1	2	5	1
Cadherin-13	CAD13_HUMAN	78 kDa	2	0	12	0	0	0	0	3	0
PDZ and LIM domain protein 5	PDLI5_HUMAN	64 kDa	0	0	17	0	0	0	0	0	0
Beta-enolase	ENOB_HUMAN	47 kDa	0	0	17	0	0	0	0	0	0
Far upstream element-binding protein 2	FUBP2_HUMAN	73 kDa	2	8	0	1	1	0	2	2	0
Mitochondrial import receptor subunit TOM70	TOM70_HUMAN	67 kDa	9	6	1	0	0	0	0	0	0
Cold-inducible RNA-binding protein	CIRBP_HUMAN	19 kDa	4	6	1	0	0	3	0	2	0
Carbonyl reductase [NADPH] 3	CBR3_HUMAN	31 kDa	5	5	0	0	0	0	0	6	0
Translin	TSN_HUMAN	26 kDa	4	5	1	0	0	2	0	4	0
Apolipoprotein O	APOO_HUMAN	22 kDa	6	5	5	0	0	0	0	0	0
Neuromodulin	NEUM_HUMAN	25 kDa	12	4	0	0	0	0	0	0	0
Glutaminase kidney isoform	GLSK_HUMAN	73 kDa	12	4	0	0	0	0	0	0	0
NADH dehydrogenase [ubiquinone] iron-sulfur protein 7	NDUS7_HUMAN	24 kDa	5	4	5	0	0	0	0	2	0
60S ribosomal protein L21	RL21_HUMAN	19 kDa	1	3	0	0	1	8	1	2	0
4F2 cell-surface antigen heavy chain	4F2_HUMAN	68 kDa	10	2	0	0	0	0	0	4	0
Actin-related protein 2/3 complex subunit 3	ARPC3_HUMAN	21 kDa	5	2	1	0	3	2	0	2	1
Nuclear mitotic apparatus protein 1	NUMA1_HUMAN	238 kDa	0	2	1	1	0	2	0	7	3
Mycophenolic acid acyl-glucuronide esterase	ABHDA_HUMAN	34 kDa	6	2	2	0	5	1	0	0	0
Nascent polypeptide-associated complex subunit alpha, muscle-specific form	NACAM_HUMAN	205 kDa	0	2	2	1	1	1	1	7	1
Low molecular weight phosphotyrosine protein phosphatase	PPAC_HUMAN	18 kDa	3	2	3	0	2	2	0	3	1
General vesicular transport factor p115	USO1_HUMAN	108 kDa	0	1	1	0	0	10	0	1	3
Thioredoxin-like protein 1	TXNL1_HUMAN	32 kDa	1	1	1	0	0	3	0	6	4
Serine/threonine-protein phosphatase 2B catalytic subunit beta isoform	PP2BB_HUMAN	59 kDa	16	0	0	0	0	0	0	0	0
CaM kinase-like vesicle-associated protein	CAMKV_HUMAN	54 kDa	16	0	0	0	0	0	0	0	0
Peroxisomal acyl-coenzyme A oxidase 1	ACOX1_HUMAN	74 kDa	0	0	0	0	16	0	0	0	0
Glutamine--fructose-6-phosphate aminotransferase [isomerizing] 1	GFPT1_HUMAN	79 kDa	0	0	0	0	1	15	0	0	0

Description	Accession	MW	Raw spectral counts								
			Frontal Cortex	Cerebellum	Right Ventricle	Mesentric lymph node	Liver	Pancreas	Proximal bile duct	Penis	Prostate
5-oxoprolinase	OPLA_HUMAN	137 kDa	0	0	0	0	8	0	0	8	0
Filaggrin	FILA_HUMAN	435 kDa	0	0	0	0	0	0	0	16	0
Plakophilin-3	PKP3_HUMAN	87 kDa	0	0	0	0	0	0	0	16	0
40S ribosomal protein S15a	RS15A_HUMAN	15 kDa	2	0	0	0	2	7	1	3	1
Alkaline phosphatase, tissue-nonspecific isozyme	PPBT_HUMAN	57 kDa	0	0	0	0	0	0	0	0	16
Histidine-rich glycoprotein	HRG_HUMAN	60 kDa	0	0	1	2	0	0	5	4	4
Transmembrane emp24 domain-containing protein 2	TMED2_HUMAN	23 kDa	0	0	1	0	1	7	0	0	7
SPRY domain-containing protein 4	SPRY4_HUMAN	23 kDa	3	0	2	0	8	2	0	0	1
26S proteasome non-ATPase regulatory subunit 1	PSMD1_HUMAN	106 kDa	1	0	4	1	2	4	0	3	1
Glycerol-3-phosphate dehydrogenase 1-like protein	GPD1L_HUMAN	38 kDa	2	0	10	1	0	2	1	0	0
Pyruvate dehydrogenase protein X component	ODPX_HUMAN	54 kDa	3	0	13	0	0	0	0	0	0
Carnitine O-palmitoyltransferase 1, muscle isoform	CPT1B_HUMAN	88 kDa	0	0	16	0	0	0	0	0	0
Sodium/potassium-transporting ATPase subunit beta-2	AT1B2_HUMAN	33 kDa	2	13	0	0	0	0	0	0	0
Guanine nucleotide-binding protein G(z) subunit alpha	GNAZ_HUMAN	41 kDa	7	8	0	0	0	0	0	0	0
Septin-6	SEPT6_HUMAN	50 kDa	8	7	0	0	0	0	0	0	0
Tropomodulin-2	TMOD2_HUMAN	40 kDa	9	6	0	0	0	0	0	0	0
Septin-8	SEPT8_HUMAN	56 kDa	10	5	0	0	0	0	0	0	0
V-type proton ATPase subunit d 1	VA0D1_HUMAN	40 kDa	8	5	0	0	0	0	0	2	0
Glutamine synthetase	GLNA_HUMAN	42 kDa	7	5	1	0	1	0	0	0	1
Splicing factor 3B subunit 1	SF3B1_HUMAN	146 kDa	0	3	0	0	1	0	0	10	1
Ubiquitin carboxyl-terminal hydrolase 5	UBP5_HUMAN	96 kDa	6	3	2	0	0	0	0	4	0
EF-hand domain-containing protein D2	EFHD2_HUMAN	27 kDa	9	2	2	0	0	0	0	2	0
Dynactin subunit 2	DCTN2_HUMAN	44 kDa	1	2	2	2	0	0	0	7	1
Eukaryotic translation initiation factor 4H	IF4H_HUMAN	27 kDa	3	2	3	0	0	3	0	4	0
Unconventional myosin-Va	MYO5A_HUMAN	215 kDa	14	1	0	0	0	0	0	0	0
Ras-related protein Rab-3C	RAB3C_HUMAN	26 kDa	13	1	0	0	0	0	0	0	1
Leucine-rich repeat-containing protein 59	LRC59_HUMAN	35 kDa	0	1	0	0	1	11	0	1	1
Prolow-density lipoprotein receptor-related protein 1	LRP1_HUMAN	505 kDa	8	1	0	0	1	0	0	4	1
Amiloride-sensitive amine oxidase [copper-containing]	AOC1_HUMAN	85 kDa	0	0	0	0	15	0	0	0	0
S-adenosylmethionine synthase isoform type-1	METK1_HUMAN	44 kDa	0	0	0	0	7	8	0	0	0
Aromatic-L-amino-acid decarboxylase	DDC_HUMAN	54 kDa	0	0	0	0	1	14	0	0	0
Endoplasmic reticulum resident protein 27	ERP27_HUMAN	30 kDa	0	0	0	0	0	15	0	0	0
Cytoskeleton-associated protein 4	CKAP4_HUMAN	66 kDa	0	0	0	0	0	10	0	5	0
Keratin, type I cuticular Ha1	K1H1_HUMAN	47 kDa	0	0	0	5	0	0	1	9	0
CAP-Gly domain-containing linker protein 1	CLIP1_HUMAN	162 kDa	1	0	0	0	0	0	0	14	0
60S acidic ribosomal protein P2	RLA2_HUMAN	12 kDa	1	0	1	0	0	8	0	5	0
Mitochondrial fission 1 protein	FIS1_HUMAN	17 kDa	4	0	2	0	2	3	0	4	0
Ig kappa chain V-I region Gal	KV107_HUMAN	12 kDa	0	0	2	5	1	0	4	2	1
Lysosome membrane protein 2	SCRB2_HUMAN	54 kDa	0	0	2	1	1	2	0	1	8
Carnitine O-acetyltransferase	CACP_HUMAN	71 kDa	1	0	11	0	3	0	0	0	0
Synaptogyrin-1	SNG1_HUMAN	25 kDa	5	9	0	0	0	0	0	0	0
Guanine nucleotide-binding protein G(s) subunit alpha isoforms XLas	GNAS1_HUMAN	111 kDa	0	8	0	0	0	0	0	6	0
Synaptic vesicle glycoprotein 2B	SV2B_HUMAN	77 kDa	8	6	0	0	0	0	0	0	0
Dual specificity mitogen-activated protein kinase kinase 1	MP2K1_HUMAN	43 kDa	8	6	0	0	0	0	0	0	0
Signal-regulatory protein beta-1	SIRB1_HUMAN	43 kDa	8	6	0	0	0	0	0	0	0
Growth factor receptor-bound protein 2	GRB2_HUMAN	25 kDa	7	6	0	0	0	0	0	1	0
Tyrosine-protein phosphatase non-receptor type substrate 1	SHPS1_HUMAN	55 kDa	9	5	0	0	0	0	0	0	0
Gamma-soluble NSF attachment protein	SNAG_HUMAN	35 kDa	7	5	0	0	0	0	0	2	0
Serine/arginine-rich splicing factor 7	SRSF7_HUMAN	27 kDa	4	5	1	0	0	1	0	3	0
Amphiphysin	AMPH_HUMAN	76 kDa	10	4	0	0	0	0	0	0	0

Description	Accession	MW	Raw spectral counts								
			Frontal Cortex	Cerebellum	Right Ventricle	Mesentric lymph node	Liver	Pancreas	Proximal bile duct	Penis	Prostate
LIM and SH3 domain protein 1	LASP1_HUMAN	30 kDa	7	4	1	0	0	1	0	1	0
Methylglutaconyl-CoA hydratase	AUHM_HUMAN	36 kDa	5	4	5	0	0	0	0	0	0
Ras-related protein Rap-2a	RAP2A_HUMAN	21 kDa	11	3	0	0	0	0	0	0	0
Monoglyceride lipase	MGLL_HUMAN	33 kDa	7	3	0	1	1	0	1	1	0
ADP-ribosylation factor 6	ARF6_HUMAN	20 kDa	2	3	0	0	4	0	0	4	1
Protein NipSnap homolog 2	NIPS2_HUMAN	34 kDa	2	3	5	0	0	1	1	2	0
Acidic leucine-rich nuclear phosphoprotein 32 family member B	AN32B_HUMAN	29 kDa	2	2	1	0	2	1	1	5	0
Histone H1x	H1X_HUMAN	22 kDa	2	2	1	0	0	1	0	8	0
Receptor expression-enhancing protein 5	REEP5_HUMAN	21 kDa	4	2	2	0	2	0	0	2	2
Interleukin enhancer-binding factor 3	ILF3_HUMAN	95 kDa	1	2	2	0	0	3	0	4	2
3-hydroxyisobutyryl-CoA hydrolase	HIBCH_HUMAN	43 kDa	2	2	4	0	3	2	0	1	0
NADH dehydrogenase [ubiquinone] iron-sulfur protein 4	NDUS4_HUMAN	20 kDa	3	2	6	0	1	1	0	1	0
60S ribosomal protein L23	RL23_HUMAN	15 kDa	4	1	0	0	1	5	0	3	0
Valine--tRNA ligase	SYVC_HUMAN	140 kDa	3	1	0	0	0	4	0	6	0
Ras-related protein Ral-B	RALB_HUMAN	23 kDa	4	1	1	0	0	0	0	5	3
Adenylate kinase 4	KAD4_HUMAN	25 kDa	3	1	9	0	1	0	0	0	0
Microsomal triglyceride transfer protein large subunit	MTP_HUMAN	99 kDa	0	0	0	0	14	0	0	0	0
Cytochrome P450 2D6	CP2D6_HUMAN	56 kDa	0	0	0	0	14	0	0	0	0
Retinol dehydrogenase 16	RDH16_HUMAN	36 kDa	0	0	0	0	14	0	0	0	0
Urocanate hydratase	HUTU_HUMAN	75 kDa	0	0	0	0	14	0	0	0	0
Chymotrypsin-like elastase family member 1	CELA1_HUMAN	28 kDa	0	0	0	0	0	14	0	0	0
Tricarboxylate transport protein	TXTP_HUMAN	34 kDa	2	0	0	3	6	0	2	1	0
Coatomer subunit zeta-1	COPZ1_HUMAN	20 kDa	1	0	0	0	4	5	0	4	0
UDP-glucuronosyltransferase 1-6	UD16_HUMAN	61 kDa	0	0	0	0	8	0	0	6	0
Macrophage-capping protein	CAPG_HUMAN	38 kDa	0	0	0	3	0	0	4	7	0
Alcohol dehydrogenase class-3	ADHX_HUMAN	40 kDa	2	0	0	0	6	1	0	0	5
Beta-mannosidase	MANBA_HUMAN	101 kDa	0	0	0	0	0	0	0	0	14
Tyrosine 3-monooxygenase	TY3H_HUMAN	59 kDa	0	0	0	0	0	0	0	0	14
Proteasome activator complex subunit 2	PSME2_HUMAN	27 kDa	1	0	1	0	4	3	0	5	0
Myosin-14	MYH14_HUMAN	228 kDa	0	0	1	0	0	0	0	13	0
Laminin subunit gamma-1	LAMC1_HUMAN	178 kDa	0	0	2	9	0	1	2	0	0
Protein ERGIC-53	LMAN1_HUMAN	58 kDa	0	0	2	0	3	8	0	0	1
Isochorismatase domain-containing protein 1	ISOC1_HUMAN	32 kDa	0	0	4	2	2	0	0	2	4
Cytochrome c oxidase subunit 5A	COX5A_HUMAN	17 kDa	3	0	5	0	0	4	0	2	0
Mitochondrial pyruvate carrier 2	MPC2_HUMAN	14 kDa	3	0	10	1	0	0	0	0	0
Neuroplastin	NPTN_HUMAN	44 kDa	3	9	0	0	0	0	0	0	1
Myc box-dependent-interacting protein 1	BIN1_HUMAN	65 kDa	9	4	0	0	0	0	0	0	0
T-complex protein 1 subunit gamma	TCPG_HUMAN	61 kDa	4	4	0	0	1	0	0	4	0
Cleavage and polyadenylation specificity factor subunit 5	CPSF5_HUMAN	26 kDa	2	4	0	0	0	1	0	6	0
Hydroxyacylglutathione hydrolase	GLO2_HUMAN	34 kDa	3	4	3	0	1	2	0	0	0
Neuronal-specific septin-3	SEPT3_HUMAN	41 kDa	10	3	0	0	0	0	0	0	0
Spliceosome RNA helicase DDX39B	DX39B_HUMAN	49 kDa	0	3	0	0	0	0	0	8	2
Methionine adenosyltransferase 2 subunit beta	MAT2B_HUMAN	38 kDa	4	3	0	0	2	0	0	1	3
Exportin-1	XPO1_HUMAN	123 kDa	6	3	1	0	0	0	0	3	0
FXYD domain-containing ion transport regulator 6	FXYD6_HUMAN	11 kDa	11	2	0	0	0	0	0	0	0
Estradiol 17-beta-dehydrogenase 12	DHB12_HUMAN	34 kDa	5	2	1	2	1	0	0	2	0
Haloacid dehalogenase-like hydrolase domain-containing protein 2	HDHD2_HUMAN	29 kDa	7	2	1	0	0	0	0	2	1
NAD(P)H-hydrate epimerase	NNRE_HUMAN	32 kDa	5	2	2	0	1	1	0	1	1
Synaptojanin-1	SYNJ1_HUMAN	173 kDa	12	1	0	0	0	0	0	0	0
A-kinase anchor protein 12	AKA12_HUMAN	191 kDa	4	1	0	7	0	0	1	0	0

Description	Accession	MW	Raw spectral counts								
			Frontal Cortex	Cerebellum	Right Ventricle	Mesentric lymph node	Liver	Pancreas	Proximal bile duct	Penis	Prostate
40S ribosomal protein S26	RS26_HUMAN	13 kDa	1	1	2	0	1	6	0	2	0
Cytochrome b-c1 complex subunit 9	QCR9_HUMAN	7 kDa	6	1	5	0	0	1	0	0	0
Atlastin-1	ATLA1_HUMAN	64 kDa	13	0	0	0	0	0	0	0	0
S-methylmethionine--homocysteine S-methyltransferase BHMT2	BHMT2_HUMAN	40 kDa	0	0	0	0	13	0	0	0	0
Cocaine esterase	EST2_HUMAN	62 kDa	0	0	0	0	13	0	0	0	0
3-oxo-5-beta-steroid 4-dehydrogenase	AK1D1_HUMAN	37 kDa	0	0	0	0	13	0	0	0	0
Homogentisate 1,2-dioxygenase	HGD_HUMAN	50 kDa	0	0	0	0	11	2	0	0	0
Spermidine synthase	SPEE_HUMAN	34 kDa	3	0	0	0	0	9	0	1	0
40S ribosomal protein S23	RS23_HUMAN	16 kDa	2	0	0	0	0	9	1	1	0
Signal recognition particle receptor subunit beta	SRPRB_HUMAN	30 kDa	0	0	0	0	0	12	0	1	0
Peptidyl-prolyl cis-trans isomerase FKBP11	FKB11_HUMAN	22 kDa	0	0	0	0	1	10	0	2	0
Histidine--tRNA ligase, cytoplasmic	SYHC_HUMAN	57 kDa	0	0	0	0	0	11	0	2	0
60S ribosomal protein L17	RL17_HUMAN	21 kDa	1	0	0	0	0	4	0	8	0
Programmed cell death protein 4	PDCD4_HUMAN	52 kDa	0	0	0	0	0	0	0	10	3
60S ribosomal protein L35	RL35_HUMAN	15 kDa	1	0	1	0	2	5	0	2	2
Fatty acid-binding protein 9	FABP9_HUMAN	15 kDa	0	0	2	5	0	0	6	0	0
6-phosphogluconolactonase	6PGL_HUMAN	28 kDa	3	0	2	0	1	2	0	4	1
Prefoldin subunit 2	PFD2_HUMAN	17 kDa	5	0	3	0	0	0	0	5	0
Acid ceramidase	ASAH1_HUMAN	45 kDa	2	0	3	0	0	0	0	0	8
Serpin B6	SPB6_HUMAN	43 kDa	0	0	5	0	0	4	0	4	0
Annexin A11	ANX11_HUMAN	54 kDa	0	0	5	0	0	0	0	4	4
Cytochrome b-c1 complex subunit 7	QCR7_HUMAN	14 kDa	5	0	6	0	0	2	0	0	0
NAD-dependent malic enzyme	MAOM_HUMAN	65 kDa	0	0	13	0	0	0	0	0	0
Synaptic vesicle membrane protein VAT-1 homolog-like	VAT1L_HUMAN	46 kDa	1	9	0	0	0	0	0	0	2
Electrogenic sodium bicarbonate cotransporter 1	S4A4_HUMAN	121 kDa	3	8	0	0	0	1	0	0	0
CB1 cannabinoid receptor-interacting protein 1	CNRP1_HUMAN	19 kDa	5	7	0	0	0	0	0	0	0
Protein lin-7 homolog A	LIN7A_HUMAN	26 kDa	5	6	0	0	0	0	0	1	0
Signal recognition particle 14 kDa protein	SRP14_HUMAN	15 kDa	2	6	0	0	0	2	0	2	0
Proline-rich transmembrane protein 2	PRRT2_HUMAN	35 kDa	7	5	0	0	0	0	0	0	0
Vacuolar protein sorting-associated protein 29	VPS29_HUMAN	21 kDa	4	4	0	0	1	0	0	3	0
Serine/arginine-rich splicing factor 3	SRSF3_HUMAN	19 kDa	3	4	0	0	0	2	0	3	0
Succinyl-CoA ligase [ADP-forming] subunit beta	SUCB1_HUMAN	50 kDa	4	4	3	0	0	0	0	1	0
V-type proton ATPase subunit H	VATH_HUMAN	56 kDa	9	3	0	0	0	0	0	0	0
ATP synthase subunit f	ATPK_HUMAN	11 kDa	1	3	4	0	1	1	0	2	0
Neuronal cell adhesion molecule	NRCAM_HUMAN	144 kDa	10	2	0	0	0	0	0	0	0
Ornithine aminotransferase	OAT_HUMAN	49 kDa	0	2	0	0	6	0	2	2	0
Non-POU domain-containing octamer-binding protein	NONO_HUMAN	54 kDa	2	2	0	0	0	3	0	4	1
Proteasome subunit beta type-6	PSB6_HUMAN	25 kDa	2	2	0	1	1	2	1	1	2
Diablo homolog	DBLOH_HUMAN	27 kDa	2	2	1	2	0	2	0	3	0
Ubiquitin-conjugating enzyme E2 variant 1	UB2V1_HUMAN	16 kDa	3	2	2	0	0	2	0	3	0
Microtubule-associated protein 4	MAP4_HUMAN	121 kDa	0	2	2	1	0	0	0	7	0
Proteasome subunit beta type-4	PSB4_HUMAN	29 kDa	3	2	2	0	0	1	0	3	1
Oxidation resistance protein 1	OXR1_HUMAN	98 kDa	11	1	0	0	0	0	0	0	0
Protein canopy homolog 2	CNPY2_HUMAN	21 kDa	3	1	0	0	2	6	0	0	0
26S proteasome non-ATPase regulatory subunit 11	PSD11_HUMAN	47 kDa	0	1	1	0	0	1	0	9	0
LanC-like protein 1	LANC1_HUMAN	45 kDa	4	1	2	0	0	0	0	2	3
Bile salt sulfotransferase	ST2A1_HUMAN	34 kDa	0	0	0	0	12	0	0	0	0
Serpin B12	SPB12_HUMAN	46 kDa	0	0	0	0	0	0	0	12	0
Galectin-3	LEG3_HUMAN	26 kDa	0	0	0	0	0	0	0	12	0
Periostin	POSTN_HUMAN	93 kDa	0	0	0	0	0	0	0	12	0

Description	Accession	MW	Raw spectral counts								
			Frontal Cortex	Cerebellum	Right Ventricle	Mesentric lymph node	Liver	Pancreas	Proximal bile duct	Penis	Prostate
Poly [ADP-ribose] polymerase 1	PARP1_HUMAN	113 kDa	0	0	0	0	0	11	0	0	1
Atlastin-3	ATLA3_HUMAN	61 kDa	0	0	0	4	1	0	0	5	2
Coatomer subunit delta	COPD_HUMAN	57 kDa	0	0	0	0	0	5	0	4	3
Delta-1-pyrroline-5-carboxylate dehydrogenase	AL4A1_HUMAN	62 kDa	0	0	1	0	9	0	0	2	0
Dynamin-2	DYN2_HUMAN	98 kDa	7	0	2	0	0	0	0	3	0
40S ribosomal protein S28	RS28_HUMAN	8 kDa	3	0	2	0	0	4	0	3	0
Ig kappa chain V-II region RPMI 6410	KV206_HUMAN	15 kDa	0	0	2	3	0	0	3	3	1
Microtubule-associated protein RP/EB family member 1	MARE1_HUMAN	30 kDa	3	0	2	1	0	1	0	3	2
Prefoldin subunit 3	PFD3_HUMAN	23 kDa	3	0	4	0	0	0	1	4	0
Peptidyl-prolyl cis-trans isomerase FKBP3	FKBP3_HUMAN	25 kDa	3	0	4	0	0	2	0	2	1
Sorbin and SH3 domain-containing protein 1	SRBS1_HUMAN	143 kDa	0	0	8	3	0	0	0	0	1
Cadherin-2	CADH2_HUMAN	100 kDa	2	0	10	0	0	0	0	0	0
Sorting and assembly machinery component 50 homolog	SAM50_HUMAN	52 kDa	2	0	10	0	0	0	0	0	0
Histone-lysine N-methyltransferase SMYD1	SMYD1_HUMAN	57 kDa	0	0	12	0	0	0	0	0	0
Carbonic anhydrase-related protein	CAH8_HUMAN	33 kDa	0	11	0	0	0	0	0	0	0
Hippocalcin-like protein 1	HPCL1_HUMAN	22 kDa	5	6	0	0	0	0	0	0	0
Septin-5	SEPT5_HUMAN	43 kDa	6	5	0	0	0	0	0	0	0
Protein lin-7 homolog C	LIN7C_HUMAN	22 kDa	4	5	0	0	0	0	0	2	0
Actin-related protein 2/3 complex subunit 5-like protein	ARP5L_HUMAN	17 kDa	5	3	0	0	0	0	0	3	0
Clathrin light chain A	CLCA_HUMAN	27 kDa	6	3	1	0	0	1	0	0	0
Peptidyl-prolyl cis-trans isomerase NIMA-interacting 1	PIN1_HUMAN	18 kDa	4	3	1	0	0	0	0	2	1
Rap1 GTPase-GDP dissociation stimulator 1	GDS1_HUMAN	66 kDa	9	2	0	0	0	0	0	0	0
Gamma-synuclein	SYUG_HUMAN	13 kDa	9	2	0	0	0	0	0	0	0
3-hydroxybutyrate dehydrogenase type 2	BDH2_HUMAN	27 kDa	5	2	0	0	2	0	1	1	0
Toll-interacting protein	TOLIP_HUMAN	30 kDa	6	2	0	0	0	0	0	3	0
Ras-related protein Ral-A	RALA_HUMAN	24 kDa	6	2	0	0	0	0	0	3	0
Nuclear migration protein nudC	NUDC_HUMAN	38 kDa	4	2	0	0	0	0	0	5	0
Calcyclin-binding protein	CYBP_HUMAN	26 kDa	6	2	2	0	0	0	0	1	0
Protein phosphatase 1 regulatory subunit 7	PP1R7_HUMAN	42 kDa	3	1	0	0	0	2	0	4	1
Adenylyl cyclase-associated protein 2	CAP2_HUMAN	53 kDa	9	1	1	0	0	0	0	0	0
T-complex protein 1 subunit alpha	TCPA_HUMAN	60 kDa	4	1	1	0	2	0	0	3	0
Ubiquitin-conjugating enzyme E2 K	UBE2K_HUMAN	22 kDa	4	1	1	0	0	0	0	5	0
60S ribosomal protein L31	RL31_HUMAN	14 kDa	2	1	1	0	0	3	0	3	1
Annexin A7	ANXA7_HUMAN	53 kDa	1	1	1	1	0	1	1	2	3
26S proteasome non-ATPase regulatory subunit 13	PSD13_HUMAN	43 kDa	3	1	3	0	0	2	0	2	0
NADH-cytochrome b5 reductase 1	NB5R1_HUMAN	34 kDa	3	1	5	1	0	0	0	0	1
Fascin	FSCN1_HUMAN	55 kDa	11	0	0	0	0	0	0	0	0
Argininosuccinate lyase	ARLY_HUMAN	52 kDa	0	0	0	0	11	0	0	0	0
Hydroxyacid oxidase 1	HAOX1_HUMAN	41 kDa	0	0	0	0	11	0	0	0	0
Serine--pyruvate aminotransferase	SPYA_HUMAN	43 kDa	0	0	0	0	11	0	0	0	0
Sarcosine dehydrogenase	SARDH_HUMAN	101 kDa	0	0	0	0	9	2	0	0	0
Chymotrypsin-C	CTRC_HUMAN	29 kDa	0	0	0	0	0	11	0	0	0
Keratin, type II cytoskeletal 7	K2C7_HUMAN	51 kDa	0	0	0	0	0	11	0	0	0
Eukaryotic translation elongation factor 1 epsilon-1	MCA3_HUMAN	20 kDa	3	0	0	0	1	6	0	1	0
Phenylalanine--tRNA ligase beta subunit	SYFB_HUMAN	66 kDa	3	0	0	0	0	7	0	1	0
Mesencephalic astrocyte-derived neurotrophic factor	MANF_HUMAN	21 kDa	0	0	0	0	3	7	0	1	0
Glucosidase 2 subunit beta	GLU2B_HUMAN	59 kDa	2	0	0	0	1	5	0	3	0
Dipeptidyl peptidase 3	DPP3_HUMAN	83 kDa	0	0	0	0	0	8	0	3	0
Eukaryotic translation initiation factor 3 subunit E	EIF3E_HUMAN	52 kDa	0	0	0	0	0	7	0	4	0
Eukaryotic translation initiation factor 4 gamma 1	IF4G1_HUMAN	175 kDa	1	0	0	0	0	2	0	8	0

Description	Accession	MW	Raw spectral counts								
			Frontal Cortex	Cerebellum	Right Ventricle	Mesentric lymph node	Liver	Pancreas	Proximal bile duct	Penis	Prostate
Protein S100-A9	S10A9_HUMAN	13 kDa	0	0	0	0	0	0	0	11	0
Ig gamma-2 chain C region	IGHG2_HUMAN	36 kDa	0	0	0	5	0	0	5	0	1
Receptor expression-enhancing protein 6	REEP6_HUMAN	21 kDa	0	0	0	0	8	2	0	0	1
Heme-binding protein 1	HEBP1_HUMAN	21 kDa	4	0	0	0	0	1	0	5	1
Bleomycin hydrolase	BLMH_HUMAN	53 kDa	0	0	0	0	0	0	0	10	1
Alpha-parvin	PARVA_HUMAN	42 kDa	0	0	0	2	0	0	2	0	7
Nicastrin	NICA_HUMAN	78 kDa	0	0	0	0	0	0	0	0	11
CD63 antigen	CD63_HUMAN	26 kDa	0	0	0	0	0	0	0	0	11
Ig gamma-1 chain C region	IGHG1_HUMAN	36 kDa	0	0	1	4	0	0	6	0	0
Microtubule-associated protein RP/EB family member 2	MARE2_HUMAN	37 kDa	8	0	1	0	0	0	0	2	0
Transthyretin	TTHY_HUMAN	16 kDa	1	0	1	2	0	1	2	3	1
17-beta-hydroxysteroid dehydrogenase 13	DHB13_HUMAN	34 kDa	0	0	1	0	8	0	0	0	2
Arachidonate 15-lipoxygenase	LOX15_HUMAN	75 kDa	1	0	2	2	1	1	2	2	0
Aspartate--tRNA ligase, cytoplasmic	SYDC_HUMAN	57 kDa	2	0	2	0	0	3	0	4	0
Ubiquitin carboxyl-terminal hydrolase 14	UBP14_HUMAN	56 kDa	4	0	2	0	0	0	0	5	0
Fibrinogen gamma chain	FIBG_HUMAN	52 kDa	0	0	3	4	0	1	2	1	0
Calpastatin	ICAL_HUMAN	77 kDa	0	0	4	0	0	0	0	7	0
PDZ and LIM domain protein 1	PDLI1_HUMAN	36 kDa	0	0	5	0	0	0	0	6	0
Sorbin and SH3 domain-containing protein 2	SRBS2_HUMAN	124 kDa	0	0	6	0	0	1	0	0	4
Carnitine O-palmitoyltransferase 2	CPT2_HUMAN	74 kDa	0	0	7	0	2	2	0	0	0
Hydroxysteroid dehydrogenase-like protein 2	HSDL2_HUMAN	45 kDa	0	0	8	0	2	0	0	1	0
ATP synthase F(0) complex subunit C2	AT5G2_HUMAN	15 kDa	0	0	11	0	0	0	0	0	0
Tripartite motif-containing protein 72	TRI72_HUMAN	53 kDa	0	0	11	0	0	0	0	0	0
Secernin-1	SCRN1_HUMAN	46 kDa	3	7	0	0	0	0	0	0	0
Heterogeneous nuclear ribonucleoprotein H3	HNRH3_HUMAN	37 kDa	2	6	0	0	0	1	0	1	0
Ankyrin-1	ANK1_HUMAN	206 kDa	0	6	0	0	0	0	0	4	0
Platelet-activating factor acetylhydrolase IB subunit gamma	PA1B3_HUMAN	26 kDa	3	4	0	0	0	0	0	3	0
Programmed cell death protein 6	PDCD6_HUMAN	22 kDa	2	3	0	0	0	2	0	3	0
Platelet-activating factor acetylhydrolase IB subunit beta	PA1B2_HUMAN	26 kDa	1	3	0	0	0	0	1	2	3
V-type proton ATPase subunit G 2	VATG2_HUMAN	14 kDa	8	2	0	0	0	0	0	0	0
S-phase kinase-associated protein 1	SKP1_HUMAN	19 kDa	3	2	0	0	0	2	0	3	0
Sorting nexin-3	SNX3_HUMAN	19 kDa	3	2	1	1	1	0	0	2	0
Exportin-2	XPO2_HUMAN	110 kDa	4	2	1	0	0	0	0	3	0
Metaxin-2	MTX2_HUMAN	30 kDa	5	2	2	0	0	1	0	0	0
Isoleucine--tRNA ligase	SYIM_HUMAN	114 kDa	2	2	3	0	2	0	0	1	0
Microtubule-associated protein 6	MAP6_HUMAN	87 kDa	9	1	0	0	0	0	0	0	0
Guanine deaminase	GUAD_HUMAN	51 kDa	10	0	0	0	0	0	0	0	0
Fatty acid-binding protein, liver	FABPL_HUMAN	14 kDa	0	0	0	0	10	0	0	0	0
Acyl-coenzyme A synthetase ACSM2A	ACS2A_HUMAN	64 kDa	0	0	0	0	10	0	0	0	0
Nucleoside diphosphate kinase 3	NDK3_HUMAN	19 kDa	4	0	0	0	4	2	0	0	0
Pancreatic lipase-related protein 2	LIPR2_HUMAN	52 kDa	0	0	0	0	0	10	0	0	0
Chymotrypsin-like protease CTRL-1	CTRL_HUMAN	28 kDa	0	0	0	0	0	10	0	0	0
Signal recognition particle subunit SRP72	SRP72_HUMAN	75 kDa	1	0	0	0	0	8	0	1	0
Importin-5	IPO5_HUMAN	124 kDa	6	0	0	0	0	0	0	4	0
Suprabasin	SBSN_HUMAN	61 kDa	0	0	0	0	0	0	0	10	0
Tripartite motif-containing protein 29	TRI29_HUMAN	66 kDa	0	0	0	0	0	0	0	10	0
Dolichyl-diphosphooligosaccharide--protein glycosyltransferase subunit DAD1	DAD1_HUMAN	12 kDa	0	0	0	0	1	8	0	0	1
UDP-glucose 4-epimerase	GALE_HUMAN	38 kDa	1	0	0	0	2	5	0	1	1
Eukaryotic translation initiation factor 3 subunit C-like protein	EIFCL_HUMAN	105 kDa	1	0	0	0	1	3	0	4	1
Dehydrogenase/reductase SDR family member 7	DHRS7_HUMAN	38 kDa	0	0	0	0	5	3	0	0	2

Description	Accession	MW	Raw spectral counts								
			Frontal Cortex	Cerebellum	Right Ventricle	Mesentric lymph node	Liver	Pancreas	Proximal bile duct	Penis	Prostate
Alpha-1-acid glycoprotein 2	A1AG2_HUMAN	24 kDa	0	0	0	0	0	0	3	4	3
CD44 antigen	CD44_HUMAN	82 kDa	0	0	0	0	0	0	0	7	3
Basement membrane-specific heparan sulfate proteoglycan core protein	PGBM_HUMAN	469 kDa	0	0	0	2	0	0	0	0	8
Lysosomal alpha-mannosidase	MA2B1_HUMAN	114 kDa	0	0	0	0	0	1	0	1	8
Translocon-associated protein subunit gamma	SSRG_HUMAN	21 kDa	0	0	1	0	1	8	0	0	0
Tumor protein D54	TPD54_HUMAN	22 kDa	0	0	1	4	0	0	2	3	0
Ig alpha-1 chain C region	IGHA1_HUMAN	38 kDa	0	0	1	2	0	0	5	1	1
Lysine--tRNA ligase	SYK_HUMAN	68 kDa	2	0	2	0	0	3	0	3	0
Copine-3	CPNE3_HUMAN	60 kDa	0	0	2	0	0	5	0	3	0
Fermitin family homolog 2	FERM2_HUMAN	78 kDa	0	0	3	1	0	0	1	0	5
NADH dehydrogenase [ubiquinone] 1 alpha subcomplex subunit 4	NDUA4_HUMAN	9 kDa	2	0	4	0	0	4	0	0	0
Protein S100-A1	S10A1_HUMAN	11 kDa	2	0	4	0	0	1	0	0	3
All-trans-retinol 13,14-reductase	RETST_HUMAN	67 kDa	0	0	5	2	3	0	0	0	0
Methylcrotonoyl-CoA carboxylase beta chain	MCCB_HUMAN	61 kDa	0	0	5	0	0	5	0	0	0
Single-stranded DNA-binding protein	SSBP_HUMAN	17 kDa	3	0	5	0	0	0	0	2	0
Calpain-2 catalytic subunit	CAN2_HUMAN	80 kDa	0	0	5	0	0	2	0	3	0
Cysteine-rich protein 2	CRIP2_HUMAN	22 kDa	2	0	6	0	0	0	0	2	0
Protein-glutamine gamma-glutamyltransferase 2	TGM2_HUMAN	77 kDa	0	0	7	0	3	0	0	0	0
Methylcrotonoyl-CoA carboxylase subunit alpha	MCCA_HUMAN	80 kDa	0	0	7	0	2	1	0	0	0
Protein unc-45 homolog B	UN45B_HUMAN	104 kDa	0	0	10	0	0	0	0	0	0
Plakophilin-2	PKP2_HUMAN	97 kDa	0	0	10	0	0	0	0	0	0
Acetyl-coenzyme A synthetase 2-like	ACS2L_HUMAN	75 kDa	0	0	10	0	0	0	0	0	0
Aquaporin-4	AQP4_HUMAN	35 kDa	4	5	0	0	0	0	0	0	0
Mitochondrial import receptor subunit TOM22 homolog	TOM22_HUMAN	16 kDa	2	5	2	0	0	0	0	0	0
Palmitoyl-protein thioesterase 1	PPT1_HUMAN	34 kDa	3	4	0	0	0	0	0	0	2
Vesicular glutamate transporter 1	VGLU1_HUMAN	62 kDa	6	3	0	0	0	0	0	0	0
Transcriptional activator protein Pur-beta	PURB_HUMAN	33 kDa	4	3	0	0	0	0	0	2	0
Endophilin-B2	SHLB2_HUMAN	44 kDa	4	3	0	0	0	0	0	2	0
Neuronal calcium sensor 1	NCS1_HUMAN	22 kDa	7	2	0	0	0	0	0	0	0
Astrocytic phosphoprotein PEA-15	PEA15_HUMAN	15 kDa	7	2	0	0	0	0	0	0	0
Dipeptidyl aminopeptidase-like protein 6	DPP6_HUMAN	98 kDa	7	2	0	0	0	0	0	0	0
Unconventional myosin-VI	MYO6_HUMAN	150 kDa	0	2	0	0	0	6	0	1	0
Basigin	BASI_HUMAN	42 kDa	4	2	3	0	0	0	0	0	0
Succinate-semialdehyde dehydrogenase	SSDH_HUMAN	57 kDa	3	2	4	0	0	0	0	0	0
Actin-related protein 2/3 complex subunit 5	ARPC5_HUMAN	16 kDa	2	1	0	0	1	2	0	3	0
ADP-ribosylation factor-like protein 8B	ARL8B_HUMAN	22 kDa	5	1	1	0	0	0	0	2	0
Crk-like protein	CRKL_HUMAN	34 kDa	3	1	1	0	0	0	0	4	0
Acylpyruvase FAHD1	FAHD1_HUMAN	25 kDa	3	1	2	0	1	0	0	2	0
DmX-like protein 2	DMXL2_HUMAN	340 kDa	9	0	0	0	0	0	0	0	0
Serum paraoxonase/arylesterase 1	PON1_HUMAN	40 kDa	0	0	0	0	9	0	0	0	0
Glycine dehydrogenase (decarboxylating)	GCSP_HUMAN	113 kDa	0	0	0	0	9	0	0	0	0
Prostaglandin reductase 1	PTGR1_HUMAN	36 kDa	0	0	0	1	8	0	0	0	0
cAMP-dependent protein kinase type II-beta regulatory subunit	KAP3_HUMAN	46 kDa	4	0	0	4	0	0	1	0	0
Hemopexin	HEMO_HUMAN	52 kDa	0	0	0	3	2	1	3	0	0
Translocating chain-associated membrane protein 1	TRAM1_HUMAN	43 kDa	0	0	0	0	0	9	0	0	0
Pancreatic secretory granule membrane major glycoprotein GP2	GP2_HUMAN	59 kDa	0	0	0	0	0	9	0	0	0
Basal cell adhesion molecule	BCAM_HUMAN	67 kDa	0	0	0	0	0	7	0	2	0
Glutathione peroxidase 1	GPX1_HUMAN	22 kDa	0	0	0	0	6	0	0	3	0
Eukaryotic translation initiation factor 2 subunit 2	IF2B_HUMAN	38 kDa	0	0	0	0	0	6	0	3	0
Biglycan	PGS1_HUMAN	42 kDa	0	0	0	2	0	0	2	5	0

Description	Accession	MW	Raw spectral counts								
			Frontal Cortex	Cerebellum	Right Ventricle	Mesentric lymph node	Liver	Pancreas	Proximal bile duct	Penis	Prostate
Eukaryotic translation initiation factor 3 subunit I	EIF3I_HUMAN	37 kDa	0	0	0	0	0	4	0	5	0
Glycolipid transfer protein	GLTP_HUMAN	24 kDa	0	0	0	0	0	0	0	9	0
Myoferlin	MYOF_HUMAN	235 kDa	0	0	0	0	0	0	0	9	0
CD109 antigen	CD109_HUMAN	162 kDa	0	0	0	0	0	0	0	9	0
Aldehyde dehydrogenase, dimeric NADP-preferring	AL3A1_HUMAN	50 kDa	0	0	0	0	0	0	0	9	0
Signal peptidase complex catalytic subunit SEC11C	SC11C_HUMAN	22 kDa	0	0	0	0	0	7	0	0	2
Galectin-1	LEG1_HUMAN	15 kDa	3	0	0	1	0	0	2	1	2
Tripeptidyl-peptidase 1	TPP1_HUMAN	61 kDa	1	0	0	1	1	1	2	1	2
ER lumen protein-retaining receptor 2	ERD22_HUMAN	24 kDa	0	0	0	0	0	6	0	0	3
Endoplasmic reticulum resident protein 44	ERP44_HUMAN	47 kDa	0	0	0	0	0	5	0	1	3
CD166 antigen	CD166_HUMAN	65 kDa	2	0	0	0	0	0	0	0	7
Lipid phosphate phosphohydrolase 1	LPP1_HUMAN	32 kDa	0	0	0	0	0	0	0	0	9
Unconventional myosin-XVIIIa	MY18A_HUMAN	233 kDa	4	0	1	0	0	1	0	3	0
26S protease regulatory subunit 6A	PRS6A_HUMAN	49 kDa	0	0	1	0	0	2	0	6	0
Eukaryotic translation initiation factor 6	IF6_HUMAN	27 kDa	0	0	1	0	1	0	0	7	0
60S ribosomal protein L19	RL19_HUMAN	23 kDa	1	0	1	0	0	5	0	0	2
Importin-7	IPO7_HUMAN	120 kDa	4	0	2	1	0	0	0	2	0
Stomatin-like protein 2	STML2_HUMAN	39 kDa	3	0	3	0	0	2	0	1	0
Cell adhesion molecule 3	CADM3_HUMAN	43 kDa	3	5	0	0	0	0	0	0	0
Acylglycerol kinase	AGK_HUMAN	47 kDa	0	5	1	0	0	1	0	1	0
Band 4.1-like protein 1	E41L1_HUMAN	99 kDa	4	4	0	0	0	0	0	0	0
Dihydropyrimidinase-related protein 5	DPYL5_HUMAN	61 kDa	4	4	0	0	0	0	0	0	0
Putative adenosylhomocysteinase 2	SAHH2_HUMAN	59 kDa	3	4	0	0	0	1	0	0	0
Solute carrier family 2, facilitated glucose transporter member 1	GTR1_HUMAN	54 kDa	1	4	0	0	0	0	1	2	0
DnaJ homolog subfamily C member 5	DNJC5_HUMAN	22 kDa	5	3	0	0	0	0	0	0	0
Dihydropyrimidinase-related protein 4	DPYL4_HUMAN	62 kDa	4	3	0	0	0	0	0	1	0
Versican core protein	CSPG2_HUMAN	373 kDa	4	3	0	0	0	0	0	1	0
Calcium-binding protein 39	CAB39_HUMAN	40 kDa	0	3	0	0	0	3	0	1	1
Protein RUFY3	RUFY3_HUMAN	53 kDa	6	2	0	0	0	0	0	0	0
Very-long-chain enoyl-CoA reductase	TECR_HUMAN	36 kDa	0	2	0	2	4	0	0	0	0
Band 4.1-like protein 2	E41L2_HUMAN	113 kDa	4	2	0	1	0	0	1	0	0
DNA-(apurinic or apyrimidinic site) lyase	APEX1_HUMAN	36 kDa	1	2	0	0	0	2	0	2	1
SRC kinase signaling inhibitor 1	SRCN1_HUMAN	112 kDa	7	1	0	0	0	0	0	0	0
Cullin-3	CUL3_HUMAN	89 kDa	2	1	0	0	0	0	0	3	2
26S protease regulatory subunit 10B	PRS10_HUMAN	44 kDa	0	1	1	0	0	0	0	6	0
PRA1 family protein 3	PRAF3_HUMAN	22 kDa	1	1	2	0	0	1	2	0	1
Brevican core protein	PGCB_HUMAN	99 kDa	8	0	0	0	0	0	0	0	0
Adenylate kinase isoenzyme 5	KAD5_HUMAN	63 kDa	8	0	0	0	0	0	0	0	0
Profilin-2	PROF2_HUMAN	15 kDa	8	0	0	0	0	0	0	0	0
Ethanolamine-phosphate cytidylyltransferase	PCY2_HUMAN	44 kDa	1	0	0	0	7	0	0	0	0
Cytochrome P450 3A4	CP3A4_HUMAN	57 kDa	0	0	0	0	8	0	0	0	0
SEC14-like protein 2	S14L2_HUMAN	46 kDa	0	0	0	0	8	0	0	0	0
Aldehyde dehydrogenase family 8 member A1	AL8A1_HUMAN	53 kDa	0	0	0	0	8	0	0	0	0
Solute carrier family 2, facilitated glucose transporter member 2	GTR2_HUMAN	57 kDa	0	0	0	0	8	0	0	0	0
Cytochrome P450 2C18	CP2CI_HUMAN	56 kDa	0	0	0	0	8	0	0	0	0
Phenazine biosynthesis-like domain-containing protein	PBLD_HUMAN	32 kDa	0	0	0	0	8	0	0	0	0
Keratin, type II cuticular Hb5	KRT85_HUMAN	56 kDa	0	0	0	8	0	0	0	0	0
Microsomal glutathione S-transferase 1	MGST1_HUMAN	18 kDa	0	0	0	2	5	0	1	0	0
Rho GDP-dissociation inhibitor 2	GDIR2_HUMAN	23 kDa	0	0	0	2	2	0	4	0	0
Ig heavy chain V-III region TEI	HV316_HUMAN	13 kDa	0	0	0	4	0	0	4	0	0

Description	Accession	MW	Raw spectral counts								
			Frontal Cortex	Cerebellum	Right Ventricle	Mesentric lymph node	Liver	Pancreas	Proximal bile duct	Penis	Prostate
Ras-related protein Rap-1A	RAP1A_HUMAN	21 kDa	0	0	0	0	0	0	8	0	0
Guanine nucleotide-binding protein subunit alpha-11	GNA11_HUMAN	42 kDa	6	0	0	0	0	2	0	0	0
Guanidinoacetate N-methyltransferase	GAMT_HUMAN	26 kDa	0	0	0	0	2	6	0	0	0
Polyadenylate-binding protein 4	PABP4_HUMAN	71 kDa	0	0	0	0	0	8	0	0	0
Protein transport protein Sec23A	SC23A_HUMAN	86 kDa	0	0	0	0	6	0	1	1	0
Vitamin D-binding protein	VTDB_HUMAN	53 kDa	0	0	0	3	0	0	4	1	0
NAD kinase 2	NAKD2_HUMAN	49 kDa	1	0	0	0	5	1	0	1	0
Transmembrane protein 205	TM205_HUMAN	21 kDa	0	0	0	0	4	3	0	1	0
Keratin, type II cuticular Hb3	KRT83_HUMAN	54 kDa	0	0	0	5	0	0	1	2	0
Tyrosine--tRNA ligase, cytoplasmic	SYYC_HUMAN	59 kDa	2	0	0	0	0	4	0	2	0
Glutamine--tRNA ligase	SYQ_HUMAN	88 kDa	0	0	0	0	0	6	0	2	0
Eukaryotic translation initiation factor 3 subunit B	EIF3B_HUMAN	92 kDa	0	0	0	0	0	6	0	2	0
Erythrocyte band 7 integral membrane protein	STOM_HUMAN	32 kDa	0	0	0	3	2	0	0	3	0
Activated RNA polymerase II transcriptional coactivator p15	TCP4_HUMAN	14 kDa	3	0	0	0	1	1	0	3	0
3-mercaptopyruvate sulfurtransferase	THTM_HUMAN	33 kDa	0	0	0	0	3	2	0	3	0
60S ribosomal protein L32	RL32_HUMAN	16 kDa	0	0	0	0	1	4	0	3	0
Eukaryotic translation initiation factor 3 subunit F	EIF3F_HUMAN	38 kDa	2	0	0	0	0	2	0	4	0
N-acetyl-D-glucosamine kinase	NAGK_HUMAN	37 kDa	1	0	0	0	0	3	0	4	0
Core histone macro-H2A.1	H2AY_HUMAN	40 kDa	1	0	0	0	0	0	1	6	0
Niban-like protein 1	NIBL1_HUMAN	84 kDa	0	0	0	0	0	0	0	8	0
Integrin beta-4	ITB4_HUMAN	202 kDa	0	0	0	0	0	0	0	8	0
Target of Nesh-SH3	TARSH_HUMAN	119 kDa	0	0	0	0	0	0	0	8	0
Serine/threonine-protein phosphatase PP1-alpha catalytic subunit	PP1A_HUMAN	38 kDa	0	0	0	0	0	0	0	8	0
26S protease regulatory subunit 8	PRS8_HUMAN	46 kDa	0	0	0	0	0	0	0	8	0
Thioredoxin domain-containing protein 5	TXND5_HUMAN	48 kDa	0	0	0	0	0	7	0	0	1
HLA class I histocompatibility antigen, Cw-17 alpha chain	1C17_HUMAN	41 kDa	0	0	0	2	0	0	4	1	1
60S ribosomal protein L36	RL36_HUMAN	12 kDa	1	0	0	0	1	2	0	2	2
Alpha/beta hydrolase domain-containing protein 14B	ABHEB_HUMAN	22 kDa	0	0	0	0	2	2	0	2	2
Lysosome-associated membrane glycoprotein 1	LAMP1_HUMAN	45 kDa	0	0	0	0	0	3	0	1	4
NADP-dependent malic enzyme	MAON_HUMAN	67 kDa	7	0	1	0	0	0	0	0	0
Rho-related GTP-binding protein RhoB	RHOB_HUMAN	22 kDa	4	0	1	0	0	3	0	0	0
Protein transport protein Sec24D	SC24D_HUMAN	113 kDa	0	0	1	0	0	7	0	0	0
U8 snoRNA-decapping enzyme	NUD16_HUMAN	21 kDa	0	0	1	0	5	1	0	1	0
60S acidic ribosomal protein P1	RLA1_HUMAN	12 kDa	1	0	1	0	0	2	2	2	0
DNA damage-binding protein 1	DDB1_HUMAN	127 kDa	1	0	1	0	1	3	0	2	0
Bifunctional purine biosynthesis protein PURH	PUR9_HUMAN	65 kDa	3	0	1	0	1	2	0	0	1
Alpha-2-HS-glycoprotein	FETUA_HUMAN	39 kDa	0	0	1	1	0	1	3	1	1
Coatomer subunit epsilon	COPE_HUMAN	34 kDa	1	0	1	0	0	4	0	1	1
Aldose 1-epimerase	GALM_HUMAN	38 kDa	0	0	1	0	2	3	0	0	2
Protein NipSnap homolog 3A	NPS3A_HUMAN	28 kDa	2	0	2	0	0	1	0	3	0
26S protease regulatory subunit 4	PRS4_HUMAN	49 kDa	0	0	2	0	0	0	0	6	0
Myotrophin	MTPN_HUMAN	13 kDa	4	0	2	0	0	1	0	0	1
Voltage-dependent calcium channel subunit alpha-2/delta-1	CA2D1_HUMAN	125 kDa	4	0	4	0	0	0	0	0	0
Peptidyl-prolyl cis-trans isomerase F	PPIF_HUMAN	22 kDa	0	0	5	0	3	0	0	0	0
Regulator of microtubule dynamics protein 1	RMD1_HUMAN	36 kDa	0	0	6	0	0	0	0	2	0
NADH-ubiquinone oxidoreductase chain 4	NU4M_HUMAN	52 kDa	1	0	7	0	0	0	0	0	0
Catenin alpha-3	CTNA3_HUMAN	100 kDa	0	0	8	0	0	0	0	0	0
Fat storage-inducing transmembrane protein 2	FITM2_HUMAN	30 kDa	0	0	8	0	0	0	0	0	0
SH3 domain-binding glutamic acid-rich protein	SH3BG_HUMAN	26 kDa	0	0	8	0	0	0	0	0	0
LanC-like protein 2	LANC2_HUMAN	51 kDa	2	5	0	0	0	0	0	0	0

Description	Accession	MW	Raw spectral counts								
			Frontal Cortex	Cerebellum	Right Ventricle	Mesentric lymph node	Liver	Pancreas	Proximal bile duct	Penis	Prostate
Gamma-adducin	ADDG_HUMAN	79 kDa	2	5	0	0	0	0	0	0	0
Syntaxin-12	STX12_HUMAN	32 kDa	3	4	0	0	0	0	0	0	0
Protein NipSnap homolog 1	NIPS1_HUMAN	33 kDa	2	4	0	0	1	0	0	0	0
Ras-related protein Rab-4B	RAB4B_HUMAN	24 kDa	2	4	0	0	0	0	0	1	0
Protein kinase C beta type	KPCB_HUMAN	77 kDa	4	3	0	0	0	0	0	0	0
Adaptin ear-binding coat-associated protein 1	NECP1_HUMAN	30 kDa	4	3	0	0	0	0	0	0	0
Phosphatidylinositol 4-kinase alpha	PI4KA_HUMAN	231 kDa	4	3	0	0	0	0	0	0	0
Neurotrimin	NTRI_HUMAN	38 kDa	4	3	0	0	0	0	0	0	0
Leukocyte surface antigen CD47	CD47_HUMAN	35 kDa	4	3	0	0	0	0	0	0	0
Heterogeneous nuclear ribonucleoprotein U-like protein 2	HNRL2_HUMAN	85 kDa	0	3	0	0	0	1	0	3	0
Heat shock 70 kDa protein 4L	HS74L_HUMAN	95 kDa	0	3	0	0	0	0	0	4	0
Peptidyl-prolyl cis-trans isomerase FKBP4	FKBP4_HUMAN	52 kDa	0	3	0	0	0	0	0	4	0
Pyridoxal phosphate phosphatase	PLPP_HUMAN	32 kDa	5	2	0	0	0	0	0	0	0
AP-3 complex subunit beta-2	AP3B2_HUMAN	119 kDa	5	2	0	0	0	0	0	0	0
Ribose-phosphate pyrophosphokinase 1	PRPS1_HUMAN	35 kDa	3	2	0	0	0	0	0	2	0
Peptidyl-prolyl cis-trans isomerase-like 1	PPIL1_HUMAN	18 kDa	1	2	0	0	0	0	0	4	0
Clathrin light chain B	CLCB_HUMAN	25 kDa	4	2	1	0	0	0	0	0	0
Ganglioside-induced differentiation-associated protein 1-like 1	GD1L1_HUMAN	42 kDa	6	1	0	0	0	0	0	0	0
Contactin-associated protein 1	CNTP1_HUMAN	156 kDa	5	1	0	0	1	0	0	0	0
Mitochondrial glutamate carrier 1	GHC1_HUMAN	34 kDa	4	1	0	0	2	0	0	0	0
Coronin-1A	COR1A_HUMAN	51 kDa	5	1	0	0	0	0	1	0	0
N(G),N(G)-dimethylarginine dimethylaminohydrolase 2	DDAH2_HUMAN	30 kDa	2	1	0	2	0	0	0	2	0
RuvB-like 2	RUVB2_HUMAN	51 kDa	1	1	0	0	0	1	0	4	0
Putative pre-mRNA-splicing factor ATP-dependent RNA helicase DHX15	DHX15_HUMAN	91 kDa	0	1	0	0	0	2	0	4	0
1,5-anhydro-D-fructose reductase	AKCL2_HUMAN	37 kDa	0	1	0	0	2	2	0	1	1
Acyl-CoA dehydrogenase family member 9	ACAD9_HUMAN	69 kDa	1	1	5	0	0	0	0	0	0
Ras-related protein Rap-2b	RAP2B_HUMAN	21 kDa	7	0	0	0	0	0	0	0	0
Catenin alpha-2	CTNA2_HUMAN	105 kDa	7	0	0	0	0	0	0	0	0
Paralemmin-1	PALM_HUMAN	42 kDa	7	0	0	0	0	0	0	0	0
V-type proton ATPase subunit F	VATF_HUMAN	13 kDa	7	0	0	0	0	0	0	0	0
Neural cell adhesion molecule 2	NCAM2_HUMAN	93 kDa	7	0	0	0	0	0	0	0	0
Synaptopodin	SYNPO_HUMAN	99 kDa	7	0	0	0	0	0	0	0	0
Protein FAM49A	FA49A_HUMAN	37 kDa	7	0	0	0	0	0	0	0	0
Non-specific lipid-transfer protein	NLTP_HUMAN	59 kDa	2	0	0	0	5	0	0	0	0
UDP-glucuronosyltransferase 2B11	UDB11_HUMAN	61 kDa	0	0	0	0	7	0	0	0	0
Corticosteroid 11-beta-dehydrogenase isozyme 1	DHI1_HUMAN	32 kDa	0	0	0	0	7	0	0	0	0
Glycine N-acyltransferase	GLYAT_HUMAN	34 kDa	0	0	0	0	7	0	0	0	0
Solute carrier family 22 member 1	S22A1_HUMAN	61 kDa	0	0	0	0	7	0	0	0	0
Aflatoxin B1 aldehyde reductase member 3	ARK73_HUMAN	37 kDa	0	0	0	0	7	0	0	0	0
Transforming protein RhoA	RHOA_HUMAN	22 kDa	6	0	0	0	0	1	0	0	0
Cytochrome b5 type B	CYB5B_HUMAN	16 kDa	3	0	0	0	1	3	0	0	0
Eukaryotic translation initiation factor 3 subunit J	EIF3J_HUMAN	29 kDa	2	0	0	0	0	5	0	0	0
ADP-ribosylation factor-like protein 1	ARL1_HUMAN	20 kDa	1	0	0	0	1	5	0	0	0
Colipase	COL_HUMAN	12 kDa	0	0	0	0	0	7	0	0	0
Signal peptidase complex subunit 3	SPCS3_HUMAN	20 kDa	0	0	0	0	0	7	0	0	0
Protein sel-1 homolog 1	SE1L1_HUMAN	89 kDa	0	0	0	0	0	7	0	0	0
Casein kinase II subunit alpha	CSK21_HUMAN	45 kDa	5	0	0	0	0	0	0	2	0
Serine hydroxymethyltransferase	GLYM_HUMAN	56 kDa	0	0	0	0	2	3	0	2	0
Dual specificity mitogen-activated protein kinase kinase 2	MP2K2_HUMAN	44 kDa	4	0	0	0	0	0	0	3	0
Membrane-associated progesterone receptor component 2	PGRC2_HUMAN	24 kDa	0	0	0	0	3	1	0	3	0

Description	Accession	MW	Raw spectral counts								
			Frontal Cortex	Cerebellum	Right Ventricle	Mesentric lymph node	Liver	Pancreas	Proximal bile duct	Penis	Prostate
Phosphatidylinositol transfer protein beta isoform	PIPNB_HUMAN	32 kDa	2	0	0	0	0	2	0	3	0
Heterogeneous nuclear ribonucleoprotein F	HNRPF_HUMAN	46 kDa	0	0	0	0	0	4	0	3	0
Inter-alpha-trypsin inhibitor heavy chain H1	ITIH1_HUMAN	101 kDa	0	0	0	0	0	0	3	4	0
Copine-1	CPNE1_HUMAN	59 kDa	0	0	0	0	0	3	0	4	0
Glutathione S-transferase Mu 4	GSTM4_HUMAN	26 kDa	0	0	0	0	0	2	0	5	0
Protein AHNAK2	AHNK2_HUMAN	617 kDa	1	0	0	0	0	0	0	6	0
Zinc finger protein 185	ZN185_HUMAN	74 kDa	0	0	0	0	0	0	0	7	0
Eukaryotic translation initiation factor 5	IF5_HUMAN	49 kDa	0	0	0	0	0	0	0	7	0
26S protease regulatory subunit 7	PRS7_HUMAN	49 kDa	0	0	0	0	0	0	0	7	0
Zinc-alpha-2-glycoprotein	ZA2G_HUMAN	34 kDa	0	0	0	0	0	0	0	7	0
Transforming growth factor-beta-induced protein ig-h3	BGH3_HUMAN	75 kDa	0	0	0	0	0	0	0	7	0
Eukaryotic translation initiation factor 2 subunit 1	IF2A_HUMAN	36 kDa	0	0	0	0	0	0	0	7	0
Glyoxylate reductase/hydroxypyruvate reductase	GRHPR_HUMAN	36 kDa	0	0	0	1	4	1	0	0	1
Mannose-1-phosphate guanyltransferase alpha	GMPPA_HUMAN	46 kDa	0	0	0	0	1	5	0	0	1
Golgi apparatus protein 1	GSLG1_HUMAN	135 kDa	0	0	0	0	0	4	0	2	1
Structural maintenance of chromosomes protein 1A	SMC1A_HUMAN	143 kDa	0	0	0	0	0	2	0	4	1
26S proteasome non-ATPase regulatory subunit 6	PSMD6_HUMAN	46 kDa	0	0	0	0	0	1	0	4	2
Vacuolar protein sorting-associated protein 26A	VP26A_HUMAN	38 kDa	0	0	0	0	0	0	0	5	2
Maleylacetoacetate isomerase	MAAI_HUMAN	24 kDa	0	0	1	0	6	0	0	0	0
COP9 signalosome complex subunit 7a	CSN7A_HUMAN	30 kDa	4	0	1	0	0	0	0	2	0
Dehydrogenase/reductase SDR family member 1	DHRS1_HUMAN	34 kDa	0	0	1	0	3	1	0	2	0
Adenosine kinase	ADK_HUMAN	41 kDa	0	0	1	0	2	1	0	3	0
Leucine--tRNA ligase, cytoplasmic	SYLC_HUMAN	134 kDa	0	0	1	0	0	2	0	4	0
Laminin subunit beta-1	LAMB1_HUMAN	198 kDa	0	0	1	5	0	0	0	0	1
Integrin-linked protein kinase	ILK_HUMAN	51 kDa	0	0	1	2	0	0	2	1	1
Glyoxalase domain-containing protein 4	GLOD4_HUMAN	35 kDa	2	0	1	0	0	0	0	3	1
NEDD8	NEDD8_HUMAN	9 kDa	3	0	2	0	0	1	0	1	0
Cytospin-B	CYTSB_HUMAN	119 kDa	2	0	2	0	0	0	0	3	0
UV excision repair protein RAD23 homolog B	RD23B_HUMAN	43 kDa	2	0	2	0	0	0	0	3	0
Nucleosome assembly protein 1-like 4	NP1L4_HUMAN	43 kDa	0	0	3	0	0	0	0	4	0
Sodium/calcium exchanger 1	NAC1_HUMAN	109 kDa	3	0	4	0	0	0	0	0	0
Complement component 1 Q subcomponent-binding protein	C1QBP_HUMAN	31 kDa	2	0	5	0	0	0	0	0	0
2-methoxy-6-polyprenyl-1,4-benzoquinol methylase	COQ5_HUMAN	37 kDa	2	0	5	0	0	0	0	0	0
Acyl-protein thioesterase 1	LYPA1_HUMAN	25 kDa	1	0	5	0	1	0	0	0	0
cAMP-dependent protein kinase type I-alpha regulatory subunit	KAP0_HUMAN	43 kDa	0	0	5	0	0	0	0	0	2
Four and a half LIM domains protein 2	FHL2_HUMAN	32 kDa	0	0	7	0	0	0	0	0	0
Glycogenin-1	GLYG_HUMAN	39 kDa	0	0	7	0	0	0	0	0	0
NLR family member X1	NLRX1_HUMAN	108 kDa	0	0	7	0	0	0	0	0	0
Myosin light chain 5	MYL5_HUMAN	20 kDa	0	0	7	0	0	0	0	0	0
ELAV-like protein 4	ELAV4_HUMAN	42 kDa	2	4	0	0	0	0	0	0	0
Heterochromatin protein 1-binding protein 3	HP1B3_HUMAN	61 kDa	0	4	0	0	0	1	0	1	0
C-terminal-binding protein 1	CTBP1_HUMAN	48 kDa	3	3	0	0	0	0	0	0	0
PH and SEC7 domain-containing protein 3	PSD3_HUMAN	116 kDa	3	3	0	0	0	0	0	0	0
V-type proton ATPase subunit S1	VAS1_HUMAN	52 kDa	3	3	0	0	0	0	0	0	0
Guanine nucleotide-binding protein subunit beta-5	GBB5_HUMAN	44 kDa	3	3	0	0	0	0	0	0	0
Cell adhesion molecule 4	CADM4_HUMAN	43 kDa	3	3	0	0	0	0	0	0	0
Metallo-beta-lactamase domain-containing protein 2	MBLC2_HUMAN	31 kDa	3	3	0	0	0	0	0	0	0
SAP domain-containing ribonucleoprotein	SARNP_HUMAN	24 kDa	0	3	0	0	0	0	0	3	0
Spectrin alpha chain, erythrocytic 1	SPTA1_HUMAN	280 kDa	0	3	0	0	0	0	0	3	0
Hsp90 co-chaperone Cdc37	CDC37_HUMAN	44 kDa	0	3	1	0	0	0	0	2	0

Description	Accession	MW	Raw spectral counts								
			Frontal Cortex	Cerebellum	Right Ventricle	Mesentric lymph node	Liver	Pancreas	Proximal bile duct	Penis	Prostate
MAGUK p55 subfamily member 2	MPP2_HUMAN	65 kDa	4	2	0	0	0	0	0	0	0
Calcineurin B homologous protein 1	CHP1_HUMAN	22 kDa	3	2	0	1	0	0	0	0	0
TAR DNA-binding protein 43	TADBP_HUMAN	45 kDa	0	2	0	0	0	0	0	4	0
High mobility group protein B2	HMGB2_HUMAN	24 kDa	0	2	0	0	0	0	0	4	0
RNA-binding motif protein, X chromosome	RBMX_HUMAN	42 kDa	0	2	0	0	1	0	0	1	2
Transcription factor A	TFAM_HUMAN	29 kDa	2	2	1	0	0	0	0	1	0
Interferon-inducible double-stranded RNA-dependent protein kinase activator A	PRKRA_HUMAN	34 kDa	5	1	0	0	0	0	0	0	0
Endophilin-A3	SH3G3_HUMAN	39 kDa	5	1	0	0	0	0	0	0	0
Synaptogyrin-3	SNG3_HUMAN	25 kDa	5	1	0	0	0	0	0	0	0
Rabphilin-3A	RP3A_HUMAN	77 kDa	5	1	0	0	0	0	0	0	0
MAGUK p55 subfamily member 6	MPP6_HUMAN	61 kDa	5	1	0	0	0	0	0	0	0
Heat shock protein 105 kDa	HS105_HUMAN	97 kDa	5	1	0	0	0	0	0	0	0
AP-2 complex subunit sigma	AP2S1_HUMAN	17 kDa	5	1	0	0	0	0	0	0	0
Ganglioside-induced differentiation-associated protein 1	GDAP1_HUMAN	41 kDa	5	1	0	0	0	0	0	0	0
Myelin-oligodendrocyte glycoprotein	MOG_HUMAN	28 kDa	5	1	0	0	0	0	0	0	0
Large proline-rich protein BAG6	BAG6_HUMAN	119 kDa	2	1	0	0	0	0	0	3	0
Far upstream element-binding protein 1	FUBP1_HUMAN	68 kDa	0	1	0	0	0	0	0	5	0
Sorting nexin-12	SNX12_HUMAN	20 kDa	3	1	0	0	0	0	0	1	1
Mitogen-activated protein kinase 3	MK03_HUMAN	43 kDa	6	0	0	0	0	0	0	0	0
Neuron-specific calcium-binding protein hippocalcin	HPCA_HUMAN	22 kDa	6	0	0	0	0	0	0	0	0
Neural cell adhesion molecule L1-like protein	CHL1_HUMAN	135 kDa	6	0	0	0	0	0	0	0	0
Glutamate receptor 2	GRIA2_HUMAN	99 kDa	6	0	0	0	0	0	0	0	0
Protein SCAI	SCAI_HUMAN	70 kDa	6	0	0	0	0	0	0	0	0
Caskin-1	CSKI1_HUMAN	150 kDa	6	0	0	0	0	0	0	0	0
NAD-dependent protein deacetylase sirtuin-2	SIR2_HUMAN	43 kDa	6	0	0	0	0	0	0	0	0
Cytochrome P450 3A7	CP3A7_HUMAN	58 kDa	0	0	0	0	6	0	0	0	0
UDP-glucuronosyltransferase 2B4	UD2B4_HUMAN	61 kDa	0	0	0	0	6	0	0	0	0
Serum paraoxonase/lactonase 3	PON3_HUMAN	40 kDa	0	0	0	0	6	0	0	0	0
GDH/6PGL endoplasmic bifunctional protein	G6PE_HUMAN	89 kDa	0	0	0	0	6	0	0	0	0
Peroxisomal membrane protein 2	PXMP2_HUMAN	22 kDa	0	0	0	0	6	0	0	0	0
Alpha-aminoadipic semialdehyde synthase	AASS_HUMAN	102 kDa	0	0	0	0	6	0	0	0	0
Ferritin light chain	FRIL_HUMAN	20 kDa	1	0	0	1	4	0	0	0	0
Keratin, type II cuticular Hb2	KRT82_HUMAN	57 kDa	0	0	0	6	0	0	0	0	0
Nidogen-1	NID1_HUMAN	136 kDa	0	0	0	6	0	0	0	0	0
Ras-related protein Rab-8B	RAB8B_HUMAN	24 kDa	3	0	0	2	0	0	1	0	0
Parathymosin	PTMS_HUMAN	12 kDa	2	0	0	0	3	1	0	0	0
Keratinocyte-associated protein 2	KTAP2_HUMAN	15 kDa	0	0	0	0	0	6	0	0	0
Signal recognition particle receptor subunit alpha	SRPR_HUMAN	70 kDa	0	0	0	0	0	6	0	0	0
Galactose-1-phosphate uridylyltransferase	GALT_HUMAN	43 kDa	0	0	0	0	0	6	0	0	0
Signal recognition particle subunit SRP68	SRP68_HUMAN	71 kDa	0	0	0	0	0	6	0	0	0
40S ribosomal protein S5	RS5_HUMAN	23 kDa	0	0	0	0	0	6	0	0	0
Tubulin-folding cofactor B	TBCB_HUMAN	27 kDa	5	0	0	0	0	0	0	1	0
Farnesyl pyrophosphate synthase	FPPS_HUMAN	48 kDa	1	0	0	0	4	0	0	1	0
60S ribosomal protein L38	RL38_HUMAN	8 kDa	2	0	0	0	1	2	0	1	0
Isoleucine--tRNA ligase, cytoplasmic	SYIC_HUMAN	145 kDa	2	0	0	0	0	3	0	1	0
40S ribosomal protein S21	RS21_HUMAN	9 kDa	2	0	0	0	0	3	0	1	0
Mannosyl-oligosaccharide glucosidase	MOGS_HUMAN	92 kDa	0	0	0	0	1	4	0	1	0
26S proteasome non-ATPase regulatory subunit 14	PSDE_HUMAN	35 kDa	4	0	0	0	0	0	0	2	0
Inositol monophosphatase 1	IMPA1_HUMAN	30 kDa	4	0	0	0	0	0	0	2	0
Phenylalanine--tRNA ligase alpha subunit	SYFA_HUMAN	58 kDa	3	0	0	0	0	1	0	2	0

Description	Accession	MW	Raw spectral counts								
			Frontal Cortex	Cerebellum	Right Ventricle	Mesentric lymph node	Liver	Pancreas	Proximal bile duct	Penis	Prostate
SH3 domain-binding glutamic acid-rich-like protein 3	SH3L3_HUMAN	10 kDa	2	0	0	0	0	2	0	2	0
Signal peptidase complex catalytic subunit SEC11A	SC11A_HUMAN	21 kDa	0	0	0	0	2	2	0	2	0
Translational activator GCN1	GCN1L_HUMAN	293 kDa	0	0	0	0	1	3	0	2	0
60S ribosomal protein L18a	RL18A_HUMAN	21 kDa	0	0	0	0	1	3	0	2	0
Thymidine phosphorylase	TYPH_HUMAN	50 kDa	0	0	0	0	3	0	0	3	0
Splicing factor 3B subunit 3	SF3B3_HUMAN	136 kDa	0	0	0	0	0	3	0	3	0
Protein NDRG1	NDRG1_HUMAN	43 kDa	2	0	0	0	0	0	0	4	0
Nuclease-sensitive element-binding protein 1	YBOX1_HUMAN	36 kDa	0	0	0	0	0	2	0	4	0
S-methyl-5'-thioadenosine phosphorylase	MTAP_HUMAN	31 kDa	0	0	0	0	0	2	0	4	0
NADP-dependent malic enzyme	MAOX_HUMAN	64 kDa	0	0	0	1	0	0	0	5	0
60 kDa SS-A/Ro ribonucleoprotein	RO60_HUMAN	61 kDa	0	0	0	0	0	0	0	6	0
Chromobox protein homolog 3	CBX3_HUMAN	21 kDa	0	0	0	0	0	0	0	6	0
DnaJ homolog subfamily B member 1	DNJB1_HUMAN	38 kDa	0	0	0	0	0	0	0	6	0
Catenin delta-1	CTND1_HUMAN	108 kDa	0	0	0	0	0	0	0	6	0
Calpain small subunit 2	CPNS2_HUMAN	28 kDa	0	0	0	0	0	0	0	6	0
UDP-glucuronosyltransferase 1-8	UD18_HUMAN	60 kDa	0	0	0	0	0	0	0	6	0
U2 small nuclear ribonucleoprotein A'	RU2A_HUMAN	28 kDa	0	0	0	0	0	0	0	6	0
Guanine nucleotide-binding protein G(k) subunit alpha	GNAI3_HUMAN	41 kDa	0	0	0	0	0	0	0	6	0
rRNA 2'-O-methyltransferase fibrillarin	FBRL_HUMAN	34 kDa	0	0	0	0	0	0	0	6	0
Quinone oxidoreductase PIG3	QORX_HUMAN	36 kDa	0	0	0	0	0	0	0	6	0
RuvB-like 1	RUVB1_HUMAN	50 kDa	0	0	0	0	0	0	0	6	0
Myelin protein P0	MYP0_HUMAN	28 kDa	0	0	0	0	0	0	0	6	0
Copine-6	CPNE6_HUMAN	62 kDa	5	0	0	0	0	0	0	0	1
Echinoderm microtubule-associated protein-like 2	EMAL2_HUMAN	71 kDa	0	0	0	0	0	5	0	0	1
SH3 domain-binding glutamic acid-rich-like protein	SH3L1_HUMAN	13 kDa	3	0	0	0	0	0	0	2	1
60S ribosomal protein L28	RL28_HUMAN	16 kDa	1	0	0	0	0	3	0	0	2
Protein disulfide-isomerase A5	PDIA5_HUMAN	60 kDa	0	0	0	0	0	4	0	0	2
Tetratricopeptide repeat protein 38	TTC38_HUMAN	53 kDa	0	0	0	0	1	2	0	1	2
Beta-galactosidase	BGAL_HUMAN	76 kDa	0	0	0	0	0	0	0	0	6
N-acetylglucosamine-6-sulfatase	GNS_HUMAN	62 kDa	0	0	0	0	0	0	0	0	6
Lipopolysaccharide-responsive and beige-like anchor protein	LRBA_HUMAN	319 kDa	0	0	0	0	0	0	0	0	6
Pterin-4-alpha-carbinolamine dehydratase	PHS_HUMAN	12 kDa	3	0	1	0	1	1	0	0	0
Charged multivesicular body protein 4b	CHM4B_HUMAN	25 kDa	4	0	1	0	0	0	0	1	0
Prefoldin subunit 5	PFD5_HUMAN	17 kDa	3	0	1	0	0	0	0	2	0
Ig mu chain C region	IGHM_HUMAN	49 kDa	0	0	1	1	0	0	2	2	0
Coiled-coil domain-containing protein 18	CCD18_HUMAN	169 kDa	2	0	1	0	0	0	0	3	0
26S proteasome non-ATPase regulatory subunit 7	PSMD7_HUMAN	37 kDa	1	0	1	0	0	0	0	4	0
COP9 signalosome complex subunit 6	CSN6_HUMAN	36 kDa	1	0	1	0	0	1	0	2	1
Alpha-1-antichymotrypsin	AACT_HUMAN	48 kDa	0	0	1	1	0	0	0	2	2
Acyl-CoA synthetase short-chain family member 3	ACSS3_HUMAN	75 kDa	0	0	2	0	4	0	0	0	0
Leucyl-cystinyl aminopeptidase	LCAP_HUMAN	117 kDa	0	0	2	4	0	0	0	0	0
Leucine-rich repeat-containing protein 47	LRC47_HUMAN	63 kDa	2	0	2	0	0	0	0	2	0
Glia-derived nexin	GDN_HUMAN	44 kDa	0	0	2	0	0	1	1	2	0
26S protease regulatory subunit 6B	PRS6B_HUMAN	47 kDa	0	0	2	0	0	1	0	3	0
Glutathione reductase	GSHR_HUMAN	56 kDa	1	0	2	0	0	2	0	0	1
Mitochondrial import inner membrane translocase subunit Tim13	TIM13_HUMAN	11 kDa	3	0	3	0	0	0	0	0	0
NADH dehydrogenase [ubiquinone] 1 alpha subcomplex subunit 2	NDUA2_HUMAN	11 kDa	3	0	3	0	0	0	0	0	0
Proteasome subunit beta type-7	PSB7_HUMAN	30 kDa	3	0	3	0	0	0	0	0	0
Isoamyl acetate-hydrolyzing esterase 1 homolog	IAH1_HUMAN	28 kDa	2	0	3	0	0	0	0	1	0
NADH dehydrogenase [ubiquinone] 1 alpha subcomplex subunit 6	NDUA6_HUMAN	18 kDa	2	0	3	0	0	0	0	1	0

Description	Accession	MW	Raw spectral counts								
			Frontal Cortex	Cerebellum	Right Ventricle	Mesentric lymph node	Liver	Pancreas	Proximal bile duct	Penis	Prostate
Glutaredoxin-1	GLRX1_HUMAN	12 kDa	1	0	3	0	0	0	0	2	0
1,4-alpha-glucan-branching enzyme	GLGB_HUMAN	80 kDa	0	0	3	0	2	0	0	0	1
28S ribosomal protein S36	RT36_HUMAN	11 kDa	2	0	4	0	0	0	0	0	0
Up-regulated during skeletal muscle growth protein 5	USMG5_HUMAN	6 kDa	1	0	4	0	0	1	0	0	0
Inorganic pyrophosphatase 2	IPYR2_HUMAN	38 kDa	0	0	4	0	0	2	0	0	0
Branched-chain-amino-acid aminotransferase	BCAT2_HUMAN	44 kDa	0	0	4	0	0	1	0	1	0
Cytochrome c oxidase subunit 1	COX1_HUMAN	57 kDa	0	0	6	0	0	0	0	0	0
Chaperone activity of bc1 complex-like	ADCK3_HUMAN	72 kDa	0	0	6	0	0	0	0	0	0
Protein Niban	NIBAN_HUMAN	103 kDa	0	0	6	0	0	0	0	0	0
Ras-related protein Rab-33B	RB33B_HUMAN	26 kDa	0	5	0	0	0	0	0	0	0
PC4 and SFRS1-interacting protein	PSIP1_HUMAN	60 kDa	0	4	0	0	0	0	0	1	0
Protein kinase C gamma type	KPCG_HUMAN	78 kDa	2	3	0	0	0	0	0	0	0
GTP-binding protein Di-Ras2	DIRA2_HUMAN	22 kDa	2	3	0	0	0	0	0	0	0
Septin-4	SEPT4_HUMAN	55 kDa	2	3	0	0	0	0	0	0	0
Transcription elongation factor A protein-like 3	TCAL3_HUMAN	23 kDa	2	3	0	0	0	0	0	0	0
Mitochondrial carrier homolog 1	MTCH1_HUMAN	42 kDa	3	2	0	0	0	0	0	0	0
Glia maturation factor beta	GMFB_HUMAN	17 kDa	2	2	0	0	0	0	0	1	0
Peptidyl-prolyl cis-trans isomerase H	PPIH_HUMAN	19 kDa	0	2	0	0	0	0	0	3	0
Secretory carrier-associated membrane protein 1	SCAM1_HUMAN	38 kDa	4	1	0	0	0	0	0	0	0
Cytosolic acyl coenzyme A thioester hydrolase	BACH_HUMAN	42 kDa	4	1	0	0	0	0	0	0	0
Neurocan core protein	NCAN_HUMAN	143 kDa	4	1	0	0	0	0	0	0	0
Armadillo repeat-containing protein 1	ARMC1_HUMAN	31 kDa	4	1	0	0	0	0	0	0	0
Putative tyrosine-protein phosphatase auxilin	AUXI_HUMAN	100 kDa	4	1	0	0	0	0	0	0	0
Phytanoyl-CoA hydroxylase-interacting protein	PHYIP_HUMAN	38 kDa	4	1	0	0	0	0	0	0	0
BRI3-binding protein	BRI3B_HUMAN	28 kDa	3	1	0	0	1	0	0	0	0
E3 ubiquitin-protein ligase CHIP	CHIP_HUMAN	35 kDa	3	1	0	0	0	0	0	1	0
Ras-related protein Rab-21	RAB21_HUMAN	24 kDa	1	1	0	2	0	0	0	1	0
Coronin-1C	COR1C_HUMAN	53 kDa	0	1	0	0	0	0	0	4	0
NSFL1 cofactor p47	NSF1C_HUMAN	41 kDa	0	1	0	0	0	0	0	4	0
SUMO-conjugating enzyme UBC9	UBC9_HUMAN	18 kDa	0	1	0	0	0	0	0	4	0
Platelet-activating factor acetylhydrolase IB subunit alpha	LIS1_HUMAN	47 kDa	1	1	0	0	0	0	0	1	2
Activator of 90 kDa heat shock protein ATPase homolog 1	AHSA1_HUMAN	38 kDa	3	1	1	0	0	0	0	0	0
Deoxynucleoside triphosphate triphosphohydrolase SAMHD1	SAMH1_HUMAN	72 kDa	0	1	1	1	0	0	1	1	0
Endothelial differentiation-related factor 1	EDF1_HUMAN	16 kDa	1	1	1	0	0	1	0	1	0
Mitochondrial import inner membrane translocase subunit TIM50	TIM50_HUMAN	40 kDa	0	1	4	0	0	0	0	0	0
Cell adhesion molecule 2	CADM2_HUMAN	48 kDa	5	0	0	0	0	0	0	0	0
Drebrin	DREB_HUMAN	71 kDa	5	0	0	0	0	0	0	0	0
Plexin-A1	PLXA1_HUMAN	211 kDa	5	0	0	0	0	0	0	0	0
Heme oxygenase 2	HMOX2_HUMAN	36 kDa	5	0	0	0	0	0	0	0	0
Dematin	DEMA_HUMAN	46 kDa	5	0	0	0	0	0	0	0	0
Neurocalcin-delta	NCALD_HUMAN	22 kDa	5	0	0	0	0	0	0	0	0
Calcium-dependent secretion activator 2	CAPS2_HUMAN	148 kDa	5	0	0	0	0	0	0	0	0
Probable phosphoglycerate mutase 4	PGAM4_HUMAN	29 kDa	5	0	0	0	0	0	0	0	0
SH3-containing GRB2-like protein 3-interacting protein 1	SGIP1_HUMAN	89 kDa	5	0	0	0	0	0	0	0	0
cAMP-dependent protein kinase catalytic subunit beta	KAPCB_HUMAN	41 kDa	5	0	0	0	0	0	0	0	0
Nicotinate-nucleotide pyrophosphorylase [carboxylating]	NADC_HUMAN	31 kDa	1	0	0	0	4	0	0	0	0
3-hydroxyanthranilate 3,4-dioxygenase	3HAO_HUMAN	33 kDa	0	0	0	0	5	0	0	0	0
Acyl-CoA dehydrogenase family member 11	ACD11_HUMAN	87 kDa	0	0	0	0	5	0	0	0	0
Peroxisomal acyl-coenzyme A oxidase 2	ACOX2_HUMAN	77 kDa	0	0	0	0	5	0	0	0	0
Dimethylaniline monooxygenase [N-oxide-forming] 3	FMO3_HUMAN	60 kDa	0	0	0	0	5	0	0	0	0

Description	Accession	MW	Raw spectral counts								
			Frontal Cortex	Cerebellum	Right Ventricle	Mesentric lymph node	Liver	Pancreas	Proximal bile duct	Penis	Prostate
Peroxisomal 2,4-dienoyl-CoA reductase	DECR2_HUMAN	31 kDa	0	0	0	0	5	0	0	0	0
Cytochrome P450 2C19	CP2CJ_HUMAN	56 kDa	0	0	0	0	5	0	0	0	0
Cytosolic beta-glucosidase	GBA3_HUMAN	54 kDa	0	0	0	0	5	0	0	0	0
Phosphoenolpyruvate carboxykinase, cytosolic [GTP]	PCKGC_HUMAN	69 kDa	0	0	0	0	5	0	0	0	0
Acetyl-CoA carboxylase 1	ACACA_HUMAN	266 kDa	0	0	0	5	0	0	0	0	0
Prostacyclin synthase	PTGIS_HUMAN	57 kDa	0	0	0	2	0	0	3	0	0
Isochorismatase domain-containing protein 2	ISOC2_HUMAN	22 kDa	1	0	0	0	3	1	0	0	0
Bifunctional epoxide hydrolase 2	HYES_HUMAN	63 kDa	0	0	0	0	4	1	0	0	0
Cystathionine gamma-lyase	CGL_HUMAN	45 kDa	0	0	0	0	4	1	0	0	0
CUB and zona pellucida-like domain-containing protein 1	CUZD1_HUMAN	68 kDa	0	0	0	0	0	5	0	0	0
Putative nucleoside diphosphate kinase	NDK8_HUMAN	16 kDa	0	0	0	0	0	5	0	0	0
Transducin beta-like protein 2	TBL2_HUMAN	50 kDa	0	0	0	0	0	5	0	0	0
Protein PRRC1	PRRC1_HUMAN	47 kDa	0	0	0	0	0	5	0	0	0
Glucagon	GLUC_HUMAN	21 kDa	0	0	0	0	0	5	0	0	0
Brain-specific angiogenesis inhibitor 1-associated protein 2	BAIP2_HUMAN	61 kDa	4	0	0	0	0	0	0	1	0
Gephyrin	GEPH_HUMAN	80 kDa	3	0	0	0	1	0	0	1	0
ADP-ribosylation factor-like protein 3	ARL3_HUMAN	20 kDa	3	0	0	0	1	0	0	1	0
Glutathione S-transferase theta-1	GSTT1_HUMAN	27 kDa	0	0	0	0	4	0	0	1	0
Lambda-crystallin homolog	CRYL1_HUMAN	35 kDa	0	0	0	0	4	0	0	1	0
Acetyl-CoA acetyltransferase, cytosolic	THIC_HUMAN	41 kDa	0	0	0	0	4	0	0	1	0
Transmembrane and coiled-coil domain-containing protein 1	TMCO1_HUMAN	21 kDa	0	0	0	0	3	1	0	1	0
UPF0556 protein C19orf10	CS010_HUMAN	19 kDa	0	0	0	0	0	4	0	1	0
E3 ubiquitin-protein ligase HUWE1	HUWE1_HUMAN	482 kDa	3	0	0	0	0	0	0	2	0
Apolipoprotein B-100	APOB_HUMAN	516 kDa	0	0	0	0	2	0	1	2	0
Transportin-1	TNPO1_HUMAN	102 kDa	1	0	0	0	0	1	1	2	0
WD repeat-containing protein 61	WDR61_HUMAN	34 kDa	1	0	0	0	0	2	0	2	0
Small nuclear ribonucleoprotein Sm D2	SMD2_HUMAN	14 kDa	1	0	0	0	0	2	0	2	0
40S ribosomal protein S11	RS11_HUMAN	18 kDa	0	0	0	0	0	3	0	2	0
Pyridoxine-5'-phosphate oxidase	PNPO_HUMAN	30 kDa	1	0	0	0	1	0	0	3	0
Glutaredoxin-3	GLRX3_HUMAN	37 kDa	0	0	0	0	0	2	0	3	0
Dermcidin	DCD_HUMAN	11 kDa	0	0	0	0	0	2	0	3	0
Eukaryotic translation initiation factor 3 subunit H	EIF3H_HUMAN	40 kDa	0	0	0	0	0	2	0	3	0
28 kDa heat- and acid-stable phosphoprotein	HAP28_HUMAN	21 kDa	1	0	0	0	0	0	0	4	0
Calponin-2	CNN2_HUMAN	34 kDa	1	0	0	0	0	0	0	4	0
Tripeptidyl-peptidase 2	TPP2_HUMAN	138 kDa	0	0	0	0	0	1	0	4	0
Protein S100-A16	S10AG_HUMAN	12 kDa	0	0	0	0	0	1	0	4	0
Ribonuclease inhibitor	RINI_HUMAN	50 kDa	0	0	0	0	0	1	0	4	0
Integrin alpha-6	ITA6_HUMAN	127 kDa	0	0	0	0	0	1	0	4	0
Tubulin--tyrosine ligase-like protein 12	TTL12_HUMAN	74 kDa	0	0	0	0	0	0	0	5	0
Clustered mitochondria protein homolog	CLU_HUMAN	147 kDa	0	0	0	0	0	0	0	5	0
Thyroid hormone receptor-associated protein 3	TR150_HUMAN	109 kDa	0	0	0	0	0	0	0	5	0
Collagen alpha-1(XVII) chain	COHA1_HUMAN	150 kDa	0	0	0	0	0	0	0	5	0
ADP-sugar pyrophosphatase	NUDT5_HUMAN	24 kDa	0	0	0	0	0	0	0	5	0
Transcription intermediary factor 1-beta	TIF1B_HUMAN	89 kDa	0	0	0	0	0	0	0	5	0
Desmocollin-3	DSC3_HUMAN	100 kDa	0	0	0	0	0	0	0	5	0
Rho-associated protein kinase 2	ROCK2_HUMAN	161 kDa	4	0	0	0	0	0	0	0	1
Eukaryotic translation initiation factor 2 subunit 3	IF2G_HUMAN	51 kDa	0	0	0	0	0	4	0	0	1
Small nuclear ribonucleoprotein Sm D1	SMD1_HUMAN	13 kDa	1	0	0	0	0	1	0	2	1
26S proteasome non-ATPase regulatory subunit 12	PSD12_HUMAN	53 kDa	0	0	0	0	0	1	0	2	2
Phospholipase D3	PLD3_HUMAN	55 kDa	2	0	0	0	0	0	0	0	3

Description	Accession	MW	Raw spectral counts								
			Frontal Cortex	Cerebellum	Right Ventricle	Mesentric lymph node	Liver	Pancreas	Proximal bile duct	Penis	Prostate
Tissue alpha-L-fucosidase	FUCO_HUMAN	54 kDa	0	0	0	0	0	0	0	1	4
Transmembrane emp24 domain-containing protein 9	TMED9_HUMAN	27 kDa	0	0	0	0	0	0	0	1	4
Alpha-N-acetylgalactosaminidase	NAGAB_HUMAN	47 kDa	0	0	0	0	0	0	0	0	5
N-acetylgalactosamine-6-sulfatase	GALNS_HUMAN	58 kDa	0	0	0	0	0	0	0	0	5
Alpha-N-acetylglucosaminidase	ANAG_HUMAN	82 kDa	0	0	0	0	0	0	0	0	5
Syntaxin-binding protein 5	STXB5_HUMAN	128 kDa	4	0	1	0	0	0	0	0	0
Transmembrane protein 65	TMM65_HUMAN	25 kDa	4	0	1	0	0	0	0	0	0
Proline synthase co-transcribed bacterial homolog protein	PROSC_HUMAN	30 kDa	1	0	1	0	1	2	0	0	0
Surfeit locus protein 4	SURF4_HUMAN	30 kDa	0	0	1	0	0	4	0	0	0
Suppressor of G2 allele of SKP1 homolog	SUGT1_HUMAN	41 kDa	2	0	1	0	0	0	0	2	0
26S proteasome non-ATPase regulatory subunit 8	PSMD8_HUMAN	40 kDa	2	0	1	0	0	0	0	2	0
Calcium-binding mitochondrial carrier protein SCaMC-1	SCMC1_HUMAN	53 kDa	0	0	1	0	0	1	0	3	0
DnaJ homolog subfamily A member 2	DNJA2_HUMAN	46 kDa	2	0	1	0	0	1	0	0	1
Iron-sulfur cluster assembly 2 homolog	ISCA2_HUMAN	16 kDa	3	0	2	0	0	0	0	0	0
Coiled-coil domain-containing protein 90B	CC90B_HUMAN	30 kDa	3	0	2	0	0	0	0	0	0
Acylphosphatase-2	ACYP2_HUMAN	11 kDa	3	0	2	0	0	0	0	0	0
Neutral cholesterol ester hydrolase 1	NCEH1_HUMAN	46 kDa	3	0	2	0	0	0	0	0	0
cAMP-dependent protein kinase catalytic subunit gamma	KAPCG_HUMAN	40 kDa	2	0	2	0	0	1	0	0	0
Fibromodulin	FMOD_HUMAN	43 kDa	2	0	2	0	0	0	0	1	0
Protein S100-A11	S10AB_HUMAN	12 kDa	0	0	2	0	0	1	0	2	0
Protein QIL1	QIL1_HUMAN	13 kDa	2	0	3	0	0	0	0	0	0
GrpE protein homolog 1	GRPE1_HUMAN	24 kDa	2	0	3	0	0	0	0	0	0
Transmembrane protein 126A	T126A_HUMAN	22 kDa	1	0	3	0	1	0	0	0	0
Acyl-coenzyme A thioesterase 13	ACO13_HUMAN	15 kDa	0	0	3	0	2	0	0	0	0
Isobutyryl-CoA dehydrogenase	ACAD8_HUMAN	45 kDa	0	0	3	0	2	0	0	0	0
HLA class II histocompatibility antigen, DR alpha chain	DRA_HUMAN	29 kDa	0	0	3	2	0	0	0	0	0
Transcription factor BTF3	BTF3_HUMAN	22 kDa	0	0	3	0	0	0	0	2	0
Extended synaptotagmin-2	ESYT2_HUMAN	102 kDa	0	0	3	0	0	0	0	2	0
Sarcolemmal membrane-associated protein	SLMAP_HUMAN	95 kDa	0	0	3	0	0	0	0	0	2
Glutaredoxin-related protein 5	GLRX5_HUMAN	17 kDa	1	0	4	0	0	0	0	0	0
FUN14 domain-containing protein 2	FUND2_HUMAN	21 kDa	0	0	4	0	0	1	0	0	0
Protein kinase C and casein kinase substrate in neurons protein 3	PACN3_HUMAN	48 kDa	0	0	4	0	0	0	0	1	0
Synaptopodin 2-like protein	SYP2L_HUMAN	102 kDa	0	0	5	0	0	0	0	0	0
Cardiac phospholamban	PPLA_HUMAN	6 kDa	0	0	5	0	0	0	0	0	0
Probable C->U-editing enzyme APOBEC-2	ABEC2_HUMAN	26 kDa	0	0	5	0	0	0	0	0	0
ELKS/Rab6-interacting/CAST family member 1	RB6I2_HUMAN	128 kDa	1	3	0	0	0	0	0	0	0
F-box only protein 2	FBX2_HUMAN	33 kDa	1	3	0	0	0	0	0	0	0
Nesprin-1	SYNE1_HUMAN	1011 kDa	1	3	0	0	0	0	0	0	0
Hepatocyte cell adhesion molecule	HECAM_HUMAN	46 kDa	1	3	0	0	0	0	0	0	0
High mobility group protein HMG-I/HMG-Y	HMGA1_HUMAN	12 kDa	0	3	1	0	0	0	0	0	0
SLIT-ROBO Rho GTPase-activating protein 2	SRGP2_HUMAN	121 kDa	2	2	0	0	0	0	0	0	0
Peripheral plasma membrane protein CASK	CSKP_HUMAN	105 kDa	2	2	0	0	0	0	0	0	0
Na(+)/H(+) exchange regulatory cofactor NHE-RF1	NHRF1_HUMAN	39 kDa	2	2	0	0	0	0	0	0	0
Heterogeneous nuclear ribonucleoprotein A0	ROA0_HUMAN	31 kDa	0	2	0	0	0	0	0	2	0
Sodium/potassium-transporting ATPase subunit beta-3	AT1B3_HUMAN	32 kDa	0	2	0	0	0	0	0	2	0
Disks large homolog 2	DLG2_HUMAN	98 kDa	3	1	0	0	0	0	0	0	0
Sodium channel subunit beta-2	SCN2B_HUMAN	24 kDa	3	1	0	0	0	0	0	0	0
Glutathione S-transferase A4	GSTA4_HUMAN	26 kDa	3	1	0	0	0	0	0	0	0
WD repeat-containing protein 7	WDR7_HUMAN	164 kDa	3	1	0	0	0	0	0	0	0
Regulator of G-protein signaling 7	RGS7_HUMAN	58 kDa	3	1	0	0	0	0	0	0	0

Description	Accession	MW	Raw spectral counts								
			Frontal Cortex	Cerebellum	Right Ventricle	Mesentric lymph node	Liver	Pancreas	Proximal bile duct	Penis	Prostate
Myelin-associated glycoprotein	MAG_HUMAN	69 kDa	3	1	0	0	0	0	0	0	0
Phosphatidylinositol transfer protein alpha isoform	PIPNA_HUMAN	32 kDa	3	1	0	0	0	0	0	0	0
Peflin	PEF1_HUMAN	30 kDa	2	1	0	0	1	0	0	0	0
Saccharopine dehydrogenase-like oxidoreductase	SCPDL_HUMAN	47 kDa	2	1	0	1	0	0	0	0	0
ATPase ASNA1	ASNA_HUMAN	39 kDa	1	1	0	0	0	0	0	2	0
NEDD8-conjugating enzyme Ubc12	UBC12_HUMAN	21 kDa	1	1	0	0	0	0	0	2	0
Trafficking protein particle complex subunit 3	TPPC3_HUMAN	20 kDa	1	1	0	0	0	0	0	2	0
U1 small nuclear ribonucleoprotein A	SNRPA_HUMAN	31 kDa	1	1	0	0	0	0	0	2	0
Small nuclear ribonucleoprotein-associated proteins B and B'	RSMB_HUMAN	25 kDa	0	1	0	0	0	0	0	3	0
N-acylneuraminate cytidylyltransferase	NEUA_HUMAN	48 kDa	0	1	0	0	1	0	0	0	2
COP9 signalosome complex subunit 8	CSN8_HUMAN	23 kDa	2	1	1	0	0	0	0	0	0
ATP-dependent Clp protease proteolytic subunit	CLPP_HUMAN	30 kDa	1	1	1	1	0	0	0	0	0
Vacuolar protein sorting-associated protein 13C	VP13C_HUMAN	422 kDa	1	1	1	1	0	0	0	0	0
KH domain-containing, RNA-binding, signal transduction-associated protein 1	KHDR1_HUMAN	48 kDa	1	1	1	0	0	0	0	1	0
Peptidyl-tRNA hydrolase ICT1	ICT1_HUMAN	24 kDa	0	1	2	0	0	0	0	1	0
OCIA domain-containing protein 1	OCAD1_HUMAN	28 kDa	0	1	3	0	0	0	0	0	0
Kinesin light chain 1	KLC1_HUMAN	65 kDa	4	0	0	0	0	0	0	0	0
Serine/threonine-protein kinase DCLK1	DCLK1_HUMAN	82 kDa	4	0	0	0	0	0	0	0	0
Rho guanine nucleotide exchange factor 2	ARHG2_HUMAN	112 kDa	4	0	0	0	0	0	0	0	0
Neurogranin	NEUG_HUMAN	8 kDa	4	0	0	0	0	0	0	0	0
Sodium-driven chloride bicarbonate exchanger	S4A10_HUMAN	126 kDa	4	0	0	0	0	0	0	0	0
Wiskott-Aldrich syndrome protein family member 1	WASF1_HUMAN	62 kDa	4	0	0	0	0	0	0	0	0
Glucose 1,6-bisphosphate synthase	PGM2L_HUMAN	70 kDa	4	0	0	0	0	0	0	0	0
Epsin-1	EPN1_HUMAN	60 kDa	4	0	0	0	0	0	0	0	0
Guanine nucleotide-binding protein G(I)/G(S)/G(O) subunit gamma-3	GBG3_HUMAN	8 kDa	4	0	0	0	0	0	0	0	0
ADP-ribosylation factor-like protein 8A	ARL8A_HUMAN	21 kDa	4	0	0	0	0	0	0	0	0
ERC protein 2	ERC2_HUMAN	111 kDa	4	0	0	0	0	0	0	0	0
Guanylate cyclase soluble subunit beta-1	GCYB1_HUMAN	71 kDa	4	0	0	0	0	0	0	0	0
CD81 antigen	CD81_HUMAN	26 kDa	4	0	0	0	0	0	0	0	0
Stathmin	STMN1_HUMAN	17 kDa	4	0	0	0	0	0	0	0	0
Voltage-gated potassium channel subunit beta-2	KCAB2_HUMAN	41 kDa	4	0	0	0	0	0	0	0	0
Serine/threonine-protein kinase PAK 1	PAK1_HUMAN	61 kDa	4	0	0	0	0	0	0	0	0
AP-2 complex subunit mu	AP2M1_HUMAN	50 kDa	4	0	0	0	0	0	0	0	0
Protein S100-B	S100B_HUMAN	11 kDa	4	0	0	0	0	0	0	0	0
Beta-centractin	ACTY_HUMAN	42 kDa	3	0	0	0	1	0	0	0	0
Peroxisomal trans-2-enoyl-CoA reductase	PECR_HUMAN	33 kDa	0	0	0	0	4	0	0	0	0
UDP-glucuronosyltransferase 2B7	UD2B7_HUMAN	61 kDa	0	0	0	0	4	0	0	0	0
Bile acyl-CoA synthetase	S27A5_HUMAN	75 kDa	0	0	0	0	4	0	0	0	0
Bile acid-CoA:amino acid N-acyltransferase	BAAT_HUMAN	46 kDa	0	0	0	0	4	0	0	0	0
Cytochrome P450 4A11	CP4AB_HUMAN	59 kDa	0	0	0	0	4	0	0	0	0
Transferrin receptor protein 2	TFR2_HUMAN	89 kDa	0	0	0	0	4	0	0	0	0
Putative L-aspartate dehydrogenase	ASPD_HUMAN	30 kDa	0	0	0	0	4	0	0	0	0
Keratin, type I cuticular Ha3-II	KT33B_HUMAN	46 kDa	0	0	0	4	0	0	0	0	0
Olfactomedin-like protein 1	OLFL1_HUMAN	46 kDa	0	0	0	4	0	0	0	0	0
Complement component C8 gamma chain	CO8G_HUMAN	22 kDa	0	0	0	0	2	0	2	0	0
1-acylglycerol-3-phosphate O-acyltransferase ABHD5	ABHD5_HUMAN	39 kDa	0	0	0	2	0	0	2	0	0
Eukaryotic translation initiation factor 1A, Y-chromosomal	IF1AY_HUMAN	16 kDa	2	0	0	0	0	2	0	0	0
Selenocysteine lyase	SCLY_HUMAN	48 kDa	0	0	0	0	2	2	0	0	0
Protein transport protein Sec24C	SC24C_HUMAN	118 kDa	0	0	0	2	0	2	0	0	0
Acyl-coenzyme A synthetase ACSM3	ACSM3_HUMAN	66 kDa	0	0	0	0	1	3	0	0	0

Description	Accession	MW	Raw spectral counts								
			Frontal Cortex	Cerebellum	Right Ventricle	Mesentric lymph node	Liver	Pancreas	Proximal bile duct	Penis	Prostate
Lysozyme C	LYSC_HUMAN	17 kDa	0	0	0	0	0	3	1	0	0
Cleft lip and palate transmembrane protein 1-like protein	CLP1L_HUMAN	62 kDa	0	0	0	0	0	4	0	0	0
Signal recognition particle 54 kDa protein	SRP54_HUMAN	56 kDa	0	0	0	0	0	4	0	0	0
Asparagine synthetase [glutamine-hydrolyzing]	ASNS_HUMAN	64 kDa	0	0	0	0	0	4	0	0	0
Transmembrane emp24 domain-containing protein 6	TMED6_HUMAN	28 kDa	0	0	0	0	0	4	0	0	0
40S ribosomal protein S27	RS27_HUMAN	9 kDa	0	0	0	0	0	4	0	0	0
BTB/POZ domain-containing protein KCTD14	KCD14_HUMAN	30 kDa	0	0	0	0	0	4	0	0	0
DnaJ homolog subfamily B member 11	DJB11_HUMAN	41 kDa	0	0	0	0	0	4	0	0	0
Calcyphosin	CAYP1_HUMAN	21 kDa	0	0	0	0	0	4	0	0	0
Reticulocalbin-1	RCN1_HUMAN	39 kDa	3	0	0	0	0	0	0	1	0
Endophilin-A2	SH3G1_HUMAN	41 kDa	3	0	0	0	0	0	0	1	0
Galactokinase	GALK1_HUMAN	42 kDa	0	0	0	0	3	0	0	1	0
Cytochrome P450 4F11	CP4FB_HUMAN	60 kDa	0	0	0	0	3	0	0	1	0
Prothrombin	THRB_HUMAN	70 kDa	0	0	0	1	0	0	2	1	0
Fibrillin-1	FBN1_HUMAN	312 kDa	0	0	0	1	0	0	2	1	0
ATP-binding cassette sub-family A member 13	ABCAD_HUMAN	576 kDa	1	0	0	0	0	2	0	1	0
Methionine--tRNA ligase, cytoplasmic	SYMC_HUMAN	101 kDa	0	0	0	0	0	3	0	1	0
Ran-specific GTPase-activating protein	RANG_HUMAN	23 kDa	2	0	0	0	0	0	0	2	0
Prolyl endopeptidase	PPCE_HUMAN	81 kDa	2	0	0	0	0	0	0	2	0
Retinol-binding protein 4	RET4_HUMAN	23 kDa	0	0	0	0	2	0	0	2	0
Lysosomal protective protein	PPGB_HUMAN	54 kDa	0	0	0	1	1	0	0	2	0
Nucleoprotein TPR	TPR_HUMAN	267 kDa	1	0	0	0	0	0	1	2	0
Proteolipid protein 2	PLP2_HUMAN	17 kDa	0	0	0	1	0	0	1	2	0
Cell cycle and apoptosis regulator protein 2	CCAR2_HUMAN	103 kDa	1	0	0	0	0	1	0	2	0
Dolichol-phosphate mannosyltransferase subunit 1	DPM1_HUMAN	30 kDa	1	0	0	0	0	1	0	2	0
ATP-dependent (S)-NAD(P)H-hydrate dehydratase	NNRD_HUMAN	37 kDa	1	0	0	0	0	1	0	2	0
Osteoclast-stimulating factor 1	OSTF1_HUMAN	24 kDa	1	0	0	0	0	0	0	3	0
Thioredoxin-related transmembrane protein 4	TMX4_HUMAN	39 kDa	1	0	0	0	0	0	0	3	0
Cytoplasmic dynein 1 intermediate chain 2	DC1I2_HUMAN	71 kDa	0	0	0	0	1	0	0	3	0
N-alpha-acetyltransferase 50	NAA50_HUMAN	19 kDa	0	0	0	0	0	1	0	3	0
Pre-mRNA-processing-splicing factor 8	PRP8_HUMAN	274 kDa	0	0	0	0	0	1	0	3	0
Glutathione S-transferase Mu 1	GSTM1_HUMAN	26 kDa	0	0	0	0	0	0	0	4	0
Histidine ammonia-lyase	HUTH_HUMAN	73 kDa	0	0	0	0	0	0	0	4	0
Protein diaphanous homolog 1	DIAP1_HUMAN	141 kDa	0	0	0	0	0	0	0	4	0
Gasdermin-A	GSDMA_HUMAN	49 kDa	0	0	0	0	0	0	0	4	0
Desmoglein-3	DSG3_HUMAN	108 kDa	0	0	0	0	0	0	0	4	0
Splicing factor 3A subunit 3	SF3A3_HUMAN	59 kDa	0	0	0	0	0	0	0	4	0
Casein kinase I isoform alpha	KC1A_HUMAN	39 kDa	0	0	0	0	0	0	0	4	0
Protein S100-A8	S10A8_HUMAN	11 kDa	0	0	0	0	0	0	0	4	0
Ras GTPase-activating protein-binding protein 1	G3BP1_HUMAN	52 kDa	0	0	0	0	0	0	0	4	0
Protein DDI1 homolog 2	DDI2_HUMAN	45 kDa	0	0	0	0	0	0	0	4	0
Ubiquitin carboxyl-terminal hydrolase isozyme L5	UCHL5_HUMAN	38 kDa	0	0	0	0	0	0	0	4	0
Peptidyl-prolyl cis-trans isomerase-like 3	PPIL3_HUMAN	18 kDa	0	0	0	0	0	0	0	4	0
Chloride intracellular channel protein 3	CLIC3_HUMAN	27 kDa	0	0	0	0	0	0	0	4	0
Calmodulin-like protein 3	CALL3_HUMAN	17 kDa	0	0	0	0	0	0	0	4	0
Kallikrein-14	KLK14_HUMAN	29 kDa	0	0	0	0	0	0	0	4	0
Keratinocyte differentiation-associated protein	KTDAP_HUMAN	11 kDa	0	0	0	0	0	0	0	4	0
Heterogeneous nuclear ribonucleoprotein A/B	ROAA_HUMAN	36 kDa	0	0	0	0	0	0	0	4	0
Y-box-binding protein 3	YBOX3_HUMAN	40 kDa	0	0	0	0	0	0	0	4	0
Desmoglein-1	DSG1_HUMAN	114 kDa	0	0	0	0	0	0	0	4	0

Description	Accession	MW	Raw spectral counts								
			Frontal Cortex	Cerebellum	Right Ventricle	Mesentric lymph node	Liver	Pancreas	Proximal bile duct	Penis	Prostate
Probable ATP-dependent RNA helicase DDX6	DDX6_HUMAN	54 kDa	0	0	0	0	0	0	0	4	0
Aldehyde dehydrogenase family 3 member B1	AL3B1_HUMAN	52 kDa	0	0	0	0	0	0	0	4	0
Putative RNA-binding protein 3	RBM3_HUMAN	17 kDa	0	0	0	0	0	0	0	4	0
Translin-associated protein X	TSNAX_HUMAN	33 kDa	0	0	0	0	0	0	0	4	0
RNA-binding protein Raly	RALY_HUMAN	32 kDa	0	0	0	0	0	0	0	4	0
Glutathione peroxidase 3	GPX3_HUMAN	26 kDa	0	0	0	1	0	0	2	0	1
Protein SEC13 homolog	SEC13_HUMAN	36 kDa	0	0	0	0	1	2	0	0	1
LIM and senescent cell antigen-like-containing domain protein 1	LIMS1_HUMAN	37 kDa	0	0	0	1	0	0	1	1	1
60S ribosomal protein L35a	RL35A_HUMAN	13 kDa	1	0	0	0	0	1	0	1	1
Flotillin-1	FLOT1_HUMAN	47 kDa	2	0	0	0	0	0	0	0	2
Aspartyl aminopeptidase	DNPEP_HUMAN	52 kDa	0	0	0	0	2	0	0	0	2
Nuclear protein localization protein 4 homolog	NPL4_HUMAN	68 kDa	1	0	0	0	0	1	0	0	2
DnaJ homolog subfamily C member 3	DNJC3_HUMAN	58 kDa	0	0	0	0	0	2	0	0	2
Septin-9	SEPT9_HUMAN	65 kDa	1	0	0	0	0	0	0	1	2
Keratin, type I cuticular Ha6	KRT36_HUMAN	52 kDa	0	0	0	1	0	0	0	1	2
Estradiol 17-beta-dehydrogenase 8	DHB8_HUMAN	27 kDa	0	0	1	0	3	0	0	0	0
Importin subunit alpha-3	IMA3_HUMAN	58 kDa	1	0	1	0	0	2	0	0	0
Peptidyl-prolyl cis-trans isomerase FKBP1A	FKB1A_HUMAN	12 kDa	1	0	1	0	0	1	0	1	0
Small nuclear ribonucleoprotein E	RUXE_HUMAN	11 kDa	1	0	1	0	0	1	0	1	0
Golgi-associated plant pathogenesis-related protein 1	GAPR1_HUMAN	17 kDa	1	0	1	0	0	0	0	2	0
Serine/threonine-protein phosphatase 2A 55 kDa regulatory subunit B	2ABA_HUMAN	52 kDa	1	0	1	0	0	0	0	2	0
Heme-binding protein 2	HEBP2_HUMAN	23 kDa	0	0	1	0	0	0	0	3	0
Thioredoxin domain-containing protein 17	TXD17_HUMAN	14 kDa	1	0	1	0	1	0	0	0	1
Prostaglandin F2 receptor negative regulator	FPRP_HUMAN	99 kDa	0	0	1	0	0	1	0	1	1
Cytochrome c oxidase subunit 7A-related protein	COX7R_HUMAN	13 kDa	2	0	2	0	0	0	0	0	0
Cytochrome c oxidase subunit 6C	COX6C_HUMAN	9 kDa	2	0	2	0	0	0	0	0	0
NADH dehydrogenase [ubiquinone] 1 beta subcomplex subunit 9	NDUB9_HUMAN	22 kDa	2	0	2	0	0	0	0	0	0
Mitochondrial carnitine/acylcarnitine carrier protein	MCAT_HUMAN	33 kDa	0	0	2	0	2	0	0	0	0
Cytochrome c oxidase subunit 6B1	CX6B1_HUMAN	10 kDa	1	0	2	0	0	1	0	0	0
Isocitrate dehydrogenase [NAD] subunit gamma	IDH3G_HUMAN	43 kDa	1	0	3	0	0	0	0	0	0
NADH dehydrogenase [ubiquinone] iron-sulfur protein 6	NDUS6_HUMAN	14 kDa	1	0	3	0	0	0	0	0	0
NADH dehydrogenase [ubiquinone] 1 alpha subcomplex subunit 5	NDUA5_HUMAN	13 kDa	1	0	3	0	0	0	0	0	0
[Pyruvate dehydrogenase [acetyl-transferring]]-phosphatase 1	PDP1_HUMAN	61 kDa	0	0	3	0	0	0	0	1	0
39S ribosomal protein L17	RM17_HUMAN	20 kDa	0	0	3	0	0	0	0	1	0
ATPase family AAA domain-containing protein 1	ATAD1_HUMAN	41 kDa	0	0	4	0	0	0	0	0	0
Desmoglein-2	DSG2_HUMAN	122 kDa	0	0	4	0	0	0	0	0	0
[Protein ADP-ribosylarginine] hydrolase-like protein 1	ARHL1_HUMAN	40 kDa	0	0	4	0	0	0	0	0	0
Enoyl-CoA hydratase domain-containing protein 2	ECHD2_HUMAN	31 kDa	0	0	4	0	0	0	0	0	0
Heat shock protein beta-7	HSPB7_HUMAN	19 kDa	0	0	4	0	0	0	0	0	0
Ubiquinone biosynthesis protein COQ7 homolog	COQ7_HUMAN	24 kDa	0	0	4	0	0	0	0	0	0
Junctophilin-2	JPH2_HUMAN	74 kDa	0	0	4	0	0	0	0	0	0
Delta-sarcoglycan	SGCD_HUMAN	32 kDa	0	0	4	0	0	0	0	0	0
Probable D-lactate dehydrogenase	LDHD_HUMAN	55 kDa	0	0	4	0	0	0	0	0	0
Ubiquinone biosynthesis protein COQ9	COQ9_HUMAN	36 kDa	0	0	4	0	0	0	0	0	0
Heat shock protein beta-2	HSPB2_HUMAN	20 kDa	0	0	4	0	0	0	0	0	0
Hexokinase-3	HXK3_HUMAN	99 kDa	0	3	0	0	0	0	0	0	0
Rho GTPase-activating protein 26	RHG26_HUMAN	92 kDa	0	3	0	0	0	0	0	0	0
Homer protein homolog 3	HOME3_HUMAN	40 kDa	0	3	0	0	0	0	0	0	0
PI-PLC X domain-containing protein 3	PLCX3_HUMAN	36 kDa	0	3	0	0	0	0	0	0	0
Dystonin	DYST_HUMAN	861 kDa	1	2	0	0	0	0	0	0	0

Description	Accession	MW	Raw spectral counts								
			Frontal Cortex	Cerebellum	Right Ventricle	Mesentric lymph node	Liver	Pancreas	Proximal bile duct	Penis	Prostate
Regulating synaptic membrane exocytosis protein 1	RIMS1_HUMAN	189 kDa	1	2	0	0	0	0	0	0	0
Glucokinase	HXK4_HUMAN	52 kDa	1	2	0	0	0	0	0	0	0
Rab3 GTPase-activating protein catalytic subunit	RB3GP_HUMAN	111 kDa	1	2	0	0	0	0	0	0	0
G-protein coupled receptor family C group 5 member B	GPC5B_HUMAN	45 kDa	1	2	0	0	0	0	0	0	0
V-type proton ATPase subunit d 2	VA0D2_HUMAN	40 kDa	1	2	0	0	0	0	0	0	0
Diphosphoinositol polyphosphate phosphohydrolase 1	NUDT3_HUMAN	19 kDa	1	2	0	0	0	0	0	0	0
Lamina-associated polypeptide 2, isoforms beta/gamma	LAP2B_HUMAN	51 kDa	0	2	0	0	0	0	0	1	0
Disks large homolog 4	DLG4_HUMAN	80 kDa	2	1	0	0	0	0	0	0	0
Histidine triad nucleotide-binding protein 3	HINT3_HUMAN	20 kDa	2	1	0	0	0	0	0	0	0
MAP kinase-activating death domain protein	MADD_HUMAN	183 kDa	2	1	0	0	0	0	0	0	0
Protein phosphatase 1H	PPM1H_HUMAN	56 kDa	2	1	0	0	0	0	0	0	0
Sodium/calcium exchanger 3	NAC3_HUMAN	103 kDa	2	1	0	0	0	0	0	0	0
Cyclin-dependent kinase 5	CDK5_HUMAN	33 kDa	2	1	0	0	0	0	0	0	0
Phospholipid-transporting ATPase IA	AT8A1_HUMAN	131 kDa	2	1	0	0	0	0	0	0	0
Complexin-1	CPLX1_HUMAN	15 kDa	2	1	0	0	0	0	0	0	0
Ras GTPase-activating protein-binding protein 2	G3BP2_HUMAN	54 kDa	2	1	0	0	0	0	0	0	0
1-phosphatidylinositol 4,5-bisphosphate phosphodiesterase delta-1	PLCD1_HUMAN	86 kDa	2	1	0	0	0	0	0	0	0
Ribosome-recycling factor	RRFM_HUMAN	29 kDa	1	1	0	0	0	1	0	0	0
Epithelial cell adhesion molecule	EPCAM_HUMAN	35 kDa	0	1	0	0	0	2	0	0	0
Dynactin subunit 3	DCTN3_HUMAN	21 kDa	1	1	0	0	0	0	0	1	0
Casein kinase II subunit beta	CSK2B_HUMAN	25 kDa	1	1	0	0	0	0	0	1	0
Eukaryotic translation initiation factor 4 gamma 3	IF4G3_HUMAN	177 kDa	1	1	0	0	0	0	0	1	0
3'(2'),5'-bisphosphate nucleotidase 1	BPNT1_HUMAN	33 kDa	0	1	0	0	1	0	0	1	0
U5 small nuclear ribonucleoprotein 200 kDa helicase	U520_HUMAN	245 kDa	0	1	0	0	0	1	0	1	0
Signal transducer and activator of transcription 1-alpha/beta	STAT1_HUMAN	87 kDa	0	1	0	0	0	0	0	2	0
Acyl-protein thioesterase 2	LYPA2_HUMAN	25 kDa	0	1	0	0	0	0	0	2	0
Serine/arginine-rich splicing factor 2	SRSF2_HUMAN	25 kDa	0	1	0	0	0	0	0	1	1
Phosphofurin acidic cluster sorting protein 1	PACS1_HUMAN	105 kDa	1	1	1	0	0	0	0	0	0
39S ribosomal protein L4	RM04_HUMAN	35 kDa	0	1	1	0	0	0	0	1	0
DENN domain-containing protein 5A	DEN5A_HUMAN	147 kDa	0	1	2	0	0	0	0	0	0
LisH domain and HEAT repeat-containing protein KIAA1468	K1468_HUMAN	135 kDa	3	0	0	0	0	0	0	0	0
Stathmin-2	STMN2_HUMAN	21 kDa	3	0	0	0	0	0	0	0	0
Protein KIAA1045	K1045_HUMAN	45 kDa	3	0	0	0	0	0	0	0	0
Guanine nucleotide-binding protein G(I)/G(S)/G(O) subunit gamma-2	GBG2_HUMAN	8 kDa	3	0	0	0	0	0	0	0	0
AP2-associated protein kinase 1	AAK1_HUMAN	104 kDa	3	0	0	0	0	0	0	0	0
Probable E3 ubiquitin-protein ligase HERC4	HERC4_HUMAN	119 kDa	3	0	0	0	0	0	0	0	0
E2/E3 hybrid ubiquitin-protein ligase UBE2O	UBE2O_HUMAN	141 kDa	3	0	0	0	0	0	0	0	0
Serine/threonine-protein phosphatase 5	PPP5_HUMAN	57 kDa	3	0	0	0	0	0	0	0	0
Dual specificity mitogen-activated protein kinase kinase 4	MP2K4_HUMAN	44 kDa	3	0	0	0	0	0	0	0	0
Reticulocalbin-2	RCN2_HUMAN	37 kDa	3	0	0	0	0	0	0	0	0
Mammalian ependymin-related protein 1	EPDR1_HUMAN	25 kDa	3	0	0	0	0	0	0	0	0
Kinesin-like protein KIF21A	KI21A_HUMAN	187 kDa	3	0	0	0	0	0	0	0	0
Actin-related protein 2/3 complex subunit 1A	ARC1A_HUMAN	42 kDa	3	0	0	0	0	0	0	0	0
Neurexin-3	NRX3A_HUMAN	181 kDa	3	0	0	0	0	0	0	0	0
Phosphatidylinositol 4-phosphate 5-kinase type-1 gamma	PI51C_HUMAN	73 kDa	3	0	0	0	0	0	0	0	0
Serine/threonine-protein phosphatase 2A 56 kDa regulatory subunit	2A5D_HUMAN	70 kDa	3	0	0	0	0	0	0	0	0
COP9 signalosome complex subunit 5	CSN5_HUMAN	38 kDa	3	0	0	0	0	0	0	0	0
Neurexin-1	NRX1A_HUMAN	162 kDa	3	0	0	0	0	0	0	0	0
Galectin-related protein	LEGL_HUMAN	19 kDa	3	0	0	0	0	0	0	0	0
Glucosamine-6-phosphate isomerase 2	GNPI2_HUMAN	31 kDa	3	0	0	0	0	0	0	0	0

Description	Accession	MW	Raw spectral counts								
			Frontal Cortex	Cerebellum	Right Ventricle	Mesentric lymph node	Liver	Pancreas	Proximal bile duct	Penis	Prostate
Glycerophosphodiester phosphodiesterase domain-containing protein 1	GDPD1_HUMAN	36 kDa	3	0	0	0	0	0	0	0	0
SH3 domain-binding glutamic acid-rich-like protein 2	SH3L2_HUMAN	12 kDa	3	0	0	0	0	0	0	0	0
Lymphocyte antigen 6H	LY6H_HUMAN	15 kDa	3	0	0	0	0	0	0	0	0
Protein stum homolog	STUM_HUMAN	15 kDa	3	0	0	0	0	0	0	0	0
Coactosin-like protein	COTL1_HUMAN	16 kDa	3	0	0	0	0	0	0	0	0
Kelch repeat and BTB domain-containing protein 11	KBTBB_HUMAN	66 kDa	3	0	0	0	0	0	0	0	0
Gamma-aminobutyric acid receptor-associated protein-like 2	GBRL2_HUMAN	14 kDa	3	0	0	0	0	0	0	0	0
Neutral amino acid transporter A	SATT_HUMAN	56 kDa	3	0	0	0	0	0	0	0	0
Enolase-phosphatase E1	ENOPH_HUMAN	29 kDa	3	0	0	0	0	0	0	0	0
Limbic system-associated membrane protein	LSAMP_HUMAN	37 kDa	3	0	0	0	0	0	0	0	0
Tubulin alpha chain-like 3	TBAL3_HUMAN	50 kDa	3	0	0	0	0	0	0	0	0
Fatty acid-binding protein, brain	FABP7_HUMAN	15 kDa	3	0	0	0	0	0	0	0	0
Serine/threonine-protein phosphatase PGAM5	PGAM5_HUMAN	32 kDa	2	0	0	0	1	0	0	0	0
4-hydroxy-2-oxoglutarate aldolase	HOGA1_HUMAN	35 kDa	0	0	0	0	3	0	0	0	0
Sterol 26-hydroxylase	CP27A_HUMAN	60 kDa	0	0	0	0	3	0	0	0	0
Beta-lactamase-like protein 2	LACB2_HUMAN	33 kDa	0	0	0	0	3	0	0	0	0
Dimethylaniline monooxygenase [N-oxide-forming] 5	FMO5_HUMAN	60 kDa	0	0	0	0	3	0	0	0	0
Cytochrome P450 2B6	CP2B6_HUMAN	56 kDa	0	0	0	0	3	0	0	0	0
UDP-glucuronosyltransferase 3A1	UD3A1_HUMAN	59 kDa	0	0	0	0	3	0	0	0	0
Bifunctional UDP-N-acetylglucosamine 2-epimerase/N-acetylmannosamine kinase	GLCNE_HUMAN	79 kDa	0	0	0	0	3	0	0	0	0
Enoyl-CoA hydratase domain-containing protein 3	ECHD3_HUMAN	33 kDa	0	0	0	0	3	0	0	0	0
Nidogen-2	NID2_HUMAN	151 kDa	0	0	0	3	0	0	0	0	0
Ubiquitin carboxyl-terminal hydrolase 7	UBP7_HUMAN	128 kDa	2	0	0	0	0	1	0	0	0
Guanine nucleotide-binding protein G(I)/G(S)/G(O) subunit gamma-12	GBG12_HUMAN	8 kDa	2	0	0	0	0	1	0	0	0
Acylamino-acid-releasing enzyme	ACPH_HUMAN	81 kDa	0	0	0	0	2	1	0	0	0
Estradiol 17-beta-dehydrogenase 11	DHB11_HUMAN	33 kDa	0	0	0	0	2	1	0	0	0
Transmembrane protein 33	TMM33_HUMAN	28 kDa	0	0	0	0	2	1	0	0	0
Peptidyl-tRNA hydrolase 2	PTH2_HUMAN	19 kDa	0	0	0	0	2	1	0	0	0
Choline dehydrogenase	CHDH_HUMAN	65 kDa	0	0	0	0	1	2	0	0	0
Thioredoxin-related transmembrane protein 1	TMX1_HUMAN	32 kDa	0	0	0	0	0	2	1	0	0
Ectonucleotide pyrophosphatase/phosphodiesterase family member 1	ENPP1_HUMAN	105 kDa	0	0	0	0	0	3	0	0	0
Transmembrane 9 superfamily member 2	TM9S2_HUMAN	76 kDa	0	0	0	0	0	3	0	0	0
Protein TFG	TFG_HUMAN	43 kDa	0	0	0	0	0	3	0	0	0
Acetyl-coenzyme A transporter 1	ACATN_HUMAN	61 kDa	0	0	0	0	0	3	0	0	0
ER lumen protein-retaining receptor 1	ERD21_HUMAN	25 kDa	0	0	0	0	0	3	0	0	0
28S ribosomal protein S15	RT15_HUMAN	30 kDa	0	0	0	0	0	3	0	0	0
Cystathionine beta-synthase	CBS_HUMAN	61 kDa	0	0	0	0	0	3	0	0	0
Fatty-acid amide hydrolase 1	FAAH1_HUMAN	63 kDa	0	0	0	0	0	3	0	0	0
Prolactin regulatory element-binding protein	PREB_HUMAN	45 kDa	0	0	0	0	0	3	0	0	0
Mannose-1-phosphate guanyltransferase beta	GMPPB_HUMAN	40 kDa	0	0	0	0	0	3	0	0	0
40S ribosomal protein S15	RS15_HUMAN	17 kDa	0	0	0	0	0	3	0	0	0
Vesicle transport protein GOT1B	GOT1B_HUMAN	15 kDa	0	0	0	0	0	3	0	0	0
ERO1-like protein beta	ERO1B_HUMAN	54 kDa	0	0	0	0	0	3	0	0	0
116 kDa U5 small nuclear ribonucleoprotein component	U5S1_HUMAN	109 kDa	0	0	0	0	0	3	0	0	0
Leucine-rich repeat-containing protein 57	LRC57_HUMAN	27 kDa	2	0	0	0	0	0	0	1	0
Tyrosine-protein phosphatase non-receptor type 11	PTN11_HUMAN	68 kDa	2	0	0	0	0	0	0	1	0
ERO1-like protein alpha	ERO1A_HUMAN	54 kDa	2	0	0	0	0	0	0	1	0
PRA1 family protein 2	PRAF2_HUMAN	19 kDa	2	0	0	0	0	0	0	1	0
HLA class I histocompatibility antigen, alpha chain G	HLAG_HUMAN	38 kDa	0	0	0	1	0	0	1	1	0
Insulin-like growth factor-binding protein complex acid labile subunit	ALS_HUMAN	66 kDa	0	0	0	0	0	0	2	1	0

Description	Accession	MW	Raw spectral counts								
			Frontal Cortex	Cerebellum	Right Ventricle	Mesentric lymph node	Liver	Pancreas	Proximal bile duct	Penis	Prostate
40S ribosomal protein S12	RS12_HUMAN	15 kDa	1	0	0	0	0	1	0	1	0
H/ACA ribonucleoprotein complex subunit 4	DKC1_HUMAN	58 kDa	0	0	0	0	0	2	0	1	0
60S ribosomal protein L30	RL30_HUMAN	13 kDa	0	0	0	0	0	2	0	1	0
Eukaryotic translation initiation factor 3 subunit D	EIF3D_HUMAN	64 kDa	0	0	0	0	0	2	0	1	0
Carboxymethylenebutenolidase homolog	CMBL_HUMAN	28 kDa	0	0	0	0	0	2	0	1	0
ER membrane protein complex subunit 2	EMC2_HUMAN	35 kDa	0	0	0	0	0	2	0	1	0
Prostaglandin E synthase 3	TEBP_HUMAN	19 kDa	1	0	0	0	0	0	0	2	0
Receptor-type tyrosine-protein phosphatase F	PTPRF_HUMAN	213 kDa	1	0	0	0	0	0	0	2	0
Golgi to ER traffic protein 4 homolog	GET4_HUMAN	37 kDa	1	0	0	0	0	0	0	2	0
Aminoacyl tRNA synthase complex-interacting multifunctional protein 1	AIMP1_HUMAN	34 kDa	1	0	0	0	0	0	0	2	0
Peptidyl-prolyl cis-trans isomerase D	PPID_HUMAN	41 kDa	1	0	0	0	0	0	0	2	0
26S proteasome non-ATPase regulatory subunit 3	PSMD3_HUMAN	61 kDa	0	0	0	1	0	0	0	2	0
Complement factor B	CFAB_HUMAN	86 kDa	0	0	0	0	0	0	1	2	0
Charged multivesicular body protein 2b	CHM2B_HUMAN	24 kDa	0	0	0	0	0	1	0	2	0
Eukaryotic peptide chain release factor GTP-binding subunit ERF3A	ERF3A_HUMAN	56 kDa	0	0	0	0	0	1	0	2	0
Serine/arginine repetitive matrix protein 1	SRRM1_HUMAN	102 kDa	0	0	0	0	0	1	0	2	0
Cytosolic purine 5'-nucleotidase	5NTC_HUMAN	65 kDa	0	0	0	0	0	1	0	2	0
Poly(ADP-ribose) glycohydrolase ARH3	ARHL2_HUMAN	39 kDa	0	0	0	0	0	0	0	3	0
SUMO-activating enzyme subunit 2	SAE2_HUMAN	71 kDa	0	0	0	0	0	0	0	3	0
Regulator of chromosome condensation	RCC1_HUMAN	45 kDa	0	0	0	0	0	0	0	3	0
Developmentally-regulated GTP-binding protein 1	DRG1_HUMAN	41 kDa	0	0	0	0	0	0	0	3	0
E3 SUMO-protein ligase RanBP2	RBP2_HUMAN	358 kDa	0	0	0	0	0	0	0	3	0
SWI/SNF complex subunit SMARCC2	SMRC2_HUMAN	133 kDa	0	0	0	0	0	0	0	3	0
Dual specificity protein phosphatase 23	DUS23_HUMAN	17 kDa	0	0	0	0	0	0	0	3	0
Serpin A12	SPA12_HUMAN	47 kDa	0	0	0	0	0	0	0	3	0
Gamma-glutamylcyclotransferase	GGCT_HUMAN	21 kDa	0	0	0	0	0	0	0	3	0
Basic leucine zipper and W2 domain-containing protein 1	BZW1_HUMAN	48 kDa	0	0	0	0	0	0	0	3	0
Desmocollin-2	DSC2_HUMAN	100 kDa	0	0	0	0	0	0	0	3	0
Chromobox protein homolog 1	CBX1_HUMAN	21 kDa	0	0	0	0	0	0	0	3	0
SH3 domain-binding protein 1	3BP1_HUMAN	76 kDa	0	0	0	0	0	0	0	3	0
Phospholipase B-like 1	PLBL1_HUMAN	63 kDa	0	0	0	0	0	0	0	3	0
Nucleolar transcription factor 1	UBF1_HUMAN	89 kDa	0	0	0	0	0	0	0	3	0
Nuclear pore complex protein Nup155	NU155_HUMAN	155 kDa	0	0	0	0	0	0	0	3	0
Adseverin	ADSV_HUMAN	80 kDa	0	0	0	0	0	0	0	3	0
Hydroxymethylglutaryl-CoA synthase, cytoplasmic	HMCS1_HUMAN	57 kDa	0	0	0	0	0	0	0	3	0
Cytochrome P450 4F22	CP4FN_HUMAN	62 kDa	0	0	0	0	0	0	0	3	0
Sulfotransferase family cytosolic 2B member 1	ST2B1_HUMAN	41 kDa	0	0	0	0	0	0	0	3	0
Ras-related protein Rab-25	RAB25_HUMAN	23 kDa	0	0	0	0	0	0	0	3	0
Glutaminyl-peptide cyclotransferase	QPCT_HUMAN	41 kDa	0	0	0	0	0	0	0	3	0
Catechol O-methyltransferase	COMT_HUMAN	30 kDa	0	0	0	0	0	0	0	3	0
Tight junction protein ZO-2	ZO2_HUMAN	134 kDa	0	0	0	0	0	0	0	3	0
Acidic leucine-rich nuclear phosphoprotein 32 family member E	AN32E_HUMAN	31 kDa	0	0	0	0	0	0	0	3	0
Eukaryotic translation initiation factor 3 subunit G	EIF3G_HUMAN	36 kDa	0	0	0	0	0	0	0	3	0
Splicing factor 3A subunit 1	SF3A1_HUMAN	89 kDa	0	0	0	0	0	0	0	3	0
Src substrate cortactin	SRC8_HUMAN	62 kDa	0	0	0	0	0	0	0	3	0
Programmed cell death protein 10	PDC10_HUMAN	25 kDa	0	0	0	0	0	0	0	3	0
Inter-alpha-trypsin inhibitor heavy chain H3	ITIH3_HUMAN	100 kDa	0	0	0	0	0	0	0	3	0
Cystatin-A	CYTA_HUMAN	11 kDa	0	0	0	0	0	0	0	3	0
Thioredoxin reductase 1, cytoplasmic	TRXR1_HUMAN	71 kDa	2	0	0	0	0	0	0	0	1
Zinc finger protein 510	ZN510_HUMAN	79 kDa	0	0	0	2	0	0	0	0	1

Description	Accession	MW	Raw spectral counts								
			Frontal Cortex	Cerebellum	Right Ventricle	Mesentric lymph node	Liver	Pancreas	Proximal bile duct	Penis	Prostate
Glucosamine 6-phosphate N-acetyltransferase	GNA1_HUMAN	21 kDa	0	0	0	0	1	1	0	0	1
Golgin subfamily A member 3	GOGA3_HUMAN	167 kDa	0	0	0	0	0	2	0	0	1
GDP-L-fucose synthase	FCL_HUMAN	36 kDa	0	0	0	0	0	1	0	1	1
NmrA-like family domain-containing protein 1	NMRL1_HUMAN	33 kDa	0	0	0	0	0	0	0	2	1
Golgin subfamily B member 1	GOGB1_HUMAN	376 kDa	0	0	0	0	0	0	0	2	1
Integrin alpha-1	ITA1_HUMAN	131 kDa	0	0	0	0	1	0	0	0	2
N-sulphoglucosamine sulphohydrolase	SPHM_HUMAN	57 kDa	0	0	0	0	0	0	0	0	3
Cation-independent mannose-6-phosphate receptor	MPRI_HUMAN	274 kDa	0	0	0	0	0	0	0	0	3
Synemin	SYNEM_HUMAN	173 kDa	0	0	0	0	0	0	0	0	3
Lysosome-associated membrane glycoprotein 2	LAMP2_HUMAN	45 kDa	0	0	0	0	0	0	0	0	3
Lysosomal acid phosphatase	PPAL_HUMAN	48 kDa	0	0	0	0	0	0	0	0	3
Aldehyde dehydrogenase family 1 member A3	AL1A3_HUMAN	56 kDa	0	0	0	0	0	0	0	0	3
Ras-related protein Rab-27B	RB27B_HUMAN	25 kDa	0	0	0	0	0	0	0	0	3
Putative phospholipase B-like 2	PLBL2_HUMAN	65 kDa	0	0	0	0	0	0	0	0	3
E3 ubiquitin-protein ligase RNF13	RNF13_HUMAN	43 kDa	0	0	0	0	0	0	0	0	3
Interferon-induced transmembrane protein 3	IFM3_HUMAN	15 kDa	0	0	0	0	0	0	0	0	3
Retinal dehydrogenase 2	AL1A2_HUMAN	57 kDa	0	0	0	0	0	0	0	0	3
Guanine nucleotide-binding protein G(I)/G(S)/G(O) subunit gamma-7	GBG7_HUMAN	8 kDa	2	0	1	0	0	0	0	0	0
Mitochondrial import receptor subunit TOM20 homolog	TOM20_HUMAN	16 kDa	2	0	1	0	0	0	0	0	0
2-oxoisovalerate dehydrogenase subunit beta	ODBB_HUMAN	43 kDa	2	0	1	0	0	0	0	0	0
Epimerase family protein SDR39U1	D39U1_HUMAN	35 kDa	2	0	1	0	0	0	0	0	0
5'-AMP-activated protein kinase catalytic subunit alpha-1	AAPK1_HUMAN	64 kDa	1	0	1	0	1	0	0	0	0
Fatty aldehyde dehydrogenase	AL3A2_HUMAN	55 kDa	0	0	1	0	2	0	0	0	0
NAD(P)H dehydrogenase [quinone] 1	NQO1_HUMAN	31 kDa	1	0	1	1	0	0	0	0	0
DnaJ homolog subfamily A member 3	DNJA3_HUMAN	52 kDa	1	0	1	0	0	1	0	0	0
Arginine--tRNA ligase, cytoplasmic	SYRC_HUMAN	75 kDa	1	0	1	0	0	0	0	1	0
Transformer-2 protein homolog beta	TRA2B_HUMAN	34 kDa	1	0	1	0	0	0	0	1	0
Cytosolic non-specific dipeptidase	CNDP2_HUMAN	53 kDa	0	0	1	0	1	0	0	1	0
StAR-related lipid transfer protein 9	STAR9_HUMAN	516 kDa	0	0	1	1	0	0	0	1	0
BAG family molecular chaperone regulator 3	BAG3_HUMAN	62 kDa	0	0	1	0	0	0	0	2	0
28S ribosomal protein S17	RT17_HUMAN	15 kDa	0	0	1	0	0	0	0	2	0
Plasminogen activator inhibitor 1 RNA-binding protein	PAIRB_HUMAN	45 kDa	0	0	1	0	0	0	0	2	0
Persulfide dioxygenase ETHE1	ETHE1_HUMAN	28 kDa	0	0	1	0	0	0	0	2	0
Palladin	PALLD_HUMAN	151 kDa	0	0	1	0	0	0	0	0	2
Serologically defined colon cancer antigen 8	SDCG8_HUMAN	83 kDa	1	0	2	0	0	0	0	0	0
Nucleosome-remodeling factor subunit BPTF	BPTF_HUMAN	338 kDa	1	0	2	0	0	0	0	0	0
Mitochondrial import inner membrane translocase subunit Tim8 A	TIM8A_HUMAN	11 kDa	1	0	2	0	0	0	0	0	0
Mitochondrial import inner membrane translocase subunit TIM16	TIM16_HUMAN	14 kDa	1	0	2	0	0	0	0	0	0
NADH dehydrogenase [ubiquinone] 1 alpha subcomplex subunit 12	NDUAC_HUMAN	17 kDa	1	0	2	0	0	0	0	0	0
Citrate lyase subunit beta-like protein	CLYBL_HUMAN	37 kDa	0	0	2	0	1	0	0	0	0
3-oxoacyl-[acyl-carrier-protein] synthase	OXSM_HUMAN	49 kDa	0	0	2	0	0	1	0	0	0
Endonuclease G	NUCG_HUMAN	33 kDa	0	0	2	0	0	0	0	1	0
39S ribosomal protein L19	RM19_HUMAN	34 kDa	0	0	3	0	0	0	0	0	0
Mitochondrial import inner membrane translocase subunit Tim21	TIM21_HUMAN	28 kDa	0	0	3	0	0	0	0	0	0
NADH dehydrogenase [ubiquinone] 1 alpha subcomplex assembly factor 3	NDUF3_HUMAN	20 kDa	0	0	3	0	0	0	0	0	0
Catechol O-methyltransferase domain-containing protein 1	CMTD1_HUMAN	29 kDa	0	0	3	0	0	0	0	0	0
Malonyl-CoA decarboxylase	DCMC_HUMAN	55 kDa	0	0	3	0	0	0	0	0	0
Mitochondrial pyruvate carrier 1	MPC1_HUMAN	12 kDa	0	0	3	0	0	0	0	0	0
Protein FAM162A	F162A_HUMAN	17 kDa	0	0	3	0	0	0	0	0	0
39S ribosomal protein L11	RM11_HUMAN	21 kDa	0	0	3	0	0	0	0	0	0

Description	Accession	MW	Raw spectral counts								
			Frontal Cortex	Cerebellum	Right Ventricle	Mesentric lymph node	Liver	Pancreas	Proximal bile duct	Penis	Prostate
Nexilin	NEXN_HUMAN	81 kDa	0	0	3	0	0	0	0	0	0
39S ribosomal protein L49	RM49_HUMAN	19 kDa	0	0	3	0	0	0	0	0	0
Alpha-1-syntrophin	SNTA1_HUMAN	54 kDa	0	0	3	0	0	0	0	0	0
Histidine triad nucleotide-binding protein 2	HINT2_HUMAN	17 kDa	0	0	3	0	0	0	0	0	0
NADH dehydrogenase [ubiquinone] 1 alpha subcomplex subunit 7	NDUA7_HUMAN	13 kDa	0	0	3	0	0	0	0	0	0
Protein DEK	DEK_HUMAN	43 kDa	0	2	0	0	0	0	0	0	0
AP-3 complex subunit sigma-1	AP3S1_HUMAN	22 kDa	0	2	0	0	0	0	0	0	0
MTSS1-like protein	MTSSL_HUMAN	80 kDa	0	2	0	0	0	0	0	0	0
MAP6 domain-containing protein 1	MA6D1_HUMAN	21 kDa	0	2	0	0	0	0	0	0	0
Growth arrest-specific protein 7	GAS7_HUMAN	54 kDa	0	2	0	0	0	0	0	0	0
Monofunctional C1-tetrahydrofolate synthase	C1TM_HUMAN	106 kDa	0	2	0	0	0	0	0	0	0
Protein rogdi homolog	ROGDI_HUMAN	32 kDa	0	2	0	0	0	0	0	0	0
Na(+)/H(+) exchange regulatory cofactor NHE-RF3	NHRF3_HUMAN	57 kDa	0	2	0	0	0	0	0	0	0
Phosphatidate cytidylyltransferase 2	CDS2_HUMAN	51 kDa	0	2	0	0	0	0	0	0	0
Synaptotagmin-12	SYT12_HUMAN	47 kDa	0	2	0	0	0	0	0	0	0
Protein tweety homolog 1	TTYH1_HUMAN	49 kDa	0	2	0	0	0	0	0	0	0
Neuronal growth regulator 1	NEGR1_HUMAN	39 kDa	1	1	0	0	0	0	0	0	0
Replication protein A 32 kDa subunit	RFA2_HUMAN	29 kDa	1	1	0	0	0	0	0	0	0
Charged multivesicular body protein 2a	CHM2A_HUMAN	25 kDa	1	1	0	0	0	0	0	0	0
Sodium channel protein type 2 subunit alpha	SCN2A_HUMAN	228 kDa	1	1	0	0	0	0	0	0	0
Type I inositol 1,4,5-trisphosphate 5-phosphatase	I5P1_HUMAN	48 kDa	1	1	0	0	0	0	0	0	0
Microtubule-associated proteins 1A/1B light chain 3A	MLP3A_HUMAN	14 kDa	1	1	0	0	0	0	0	0	0
Ras-related C3 botulinum toxin substrate 3	RAC3_HUMAN	21 kDa	1	1	0	0	0	0	0	0	0
Tryptophan--tRNA ligase, cytoplasmic	SYWC_HUMAN	53 kDa	1	1	0	0	0	0	0	0	0
Hepatoma-derived growth factor-related protein 2	HDGR2_HUMAN	74 kDa	1	1	0	0	0	0	0	0	0
Sodium- and chloride-dependent GABA transporter 1	SC6A1_HUMAN	67 kDa	1	1	0	0	0	0	0	0	0
Cytochrome c oxidase subunit 2	COX2_HUMAN	26 kDa	1	1	0	0	0	0	0	0	0
Cullin-5	CUL5_HUMAN	91 kDa	1	1	0	0	0	0	0	0	0
U1 small nuclear ribonucleoprotein 70 kDa	RU17_HUMAN	52 kDa	0	1	0	0	0	1	0	0	0
RNA-binding protein FUS	FUS_HUMAN	53 kDa	0	1	0	0	0	1	0	0	0
Small ubiquitin-related modifier 2	SUMO2_HUMAN	11 kDa	0	1	0	0	0	1	0	0	0
DnaJ homolog subfamily C member 8	DNJC8_HUMAN	30 kDa	0	1	0	0	0	0	0	1	0
Splicing factor 3B subunit 2	SF3B2_HUMAN	100 kDa	0	1	0	0	0	0	0	1	0
Cytoskeleton-associated protein 5	CKAP5_HUMAN	226 kDa	0	1	0	0	0	0	0	1	0
G2/mitotic-specific cyclin-B3	CCNB3_HUMAN	158 kDa	0	1	0	0	0	0	0	0	1
Histone-lysine N-methyltransferase 2D	KMT2D_HUMAN	593 kDa	0	1	1	0	0	0	0	0	0
Ral GTPase-activating protein subunit beta	RLGPB_HUMAN	167 kDa	0	1	1	0	0	0	0	0	0
CCA tRNA nucleotidyltransferase 1	TRNT1_HUMAN	50 kDa	0	1	1	0	0	0	0	0	0
Flotillin-2	FLOT2_HUMAN	47 kDa	2	0	0	0	0	0	0	0	0
Acylphosphatase-1	ACYP1_HUMAN	11 kDa	2	0	0	0	0	0	0	0	0
CLIP-associating protein 2	CLAP2_HUMAN	141 kDa	2	0	0	0	0	0	0	0	0
TIP41-like protein	TIPRL_HUMAN	31 kDa	2	0	0	0	0	0	0	0	0
Sorting nexin-1	SNX1_HUMAN	59 kDa	2	0	0	0	0	0	0	0	0
Liprin-alpha-3	LIPA3_HUMAN	134 kDa	2	0	0	0	0	0	0	0	0
Catenin delta-2	CTND2_HUMAN	133 kDa	2	0	0	0	0	0	0	0	0
Pseudouridine-5'-monophosphatase	HDHD1_HUMAN	25 kDa	2	0	0	0	0	0	0	0	0
Glutamate receptor 3	GRIA3_HUMAN	101 kDa	2	0	0	0	0	0	0	0	0
Ubiquilin-2	UBQL2_HUMAN	66 kDa	2	0	0	0	0	0	0	0	0
Target of Myb protein 1	TOM1_HUMAN	54 kDa	2	0	0	0	0	0	0	0	0
Protein unc-13 homolog A	UN13A_HUMAN	193 kDa	2	0	0	0	0	0	0	0	0

Description	Accession	MW	Raw spectral counts								
			Frontal Cortex	Cerebellum	Right Ventricle	Mesentric lymph node	Liver	Pancreas	Proximal bile duct	Penis	Prostate
UDP-N-acetylglucosamine--peptide N-acetylglucosaminyltransferase 110 kDa subunit	OGT1_HUMAN	117 kDa	2	0	0	0	0	0	0	0	0
Glutamate decarboxylase 2	DCE2_HUMAN	65 kDa	2	0	0	0	0	0	0	0	0
Syntaxin-6	STX6_HUMAN	29 kDa	2	0	0	0	0	0	0	0	0
Mitochondrial uncoupling protein 4	UCP4_HUMAN	36 kDa	2	0	0	0	0	0	0	0	0
Zinc finger protein 292	ZN292_HUMAN	305 kDa	2	0	0	0	0	0	0	0	0
Transcription elongation factor B polypeptide 1	ELOC_HUMAN	12 kDa	2	0	0	0	0	0	0	0	0
Myotubularin-related protein 5	MTMR5_HUMAN	208 kDa	2	0	0	0	0	0	0	0	0
Glutamate receptor ionotropic, kainate 4	GRIK4_HUMAN	107 kDa	2	0	0	0	0	0	0	0	0
AP-3 complex subunit delta-1	AP3D1_HUMAN	130 kDa	2	0	0	0	0	0	0	0	0
Fas-binding factor 1	FBF1_HUMAN	125 kDa	2	0	0	0	0	0	0	0	0
NACHT, LRR and PYD domains-containing protein 14	NAL14_HUMAN	125 kDa	2	0	0	0	0	0	0	0	0
5'-3' exoribonuclease 1	XRN1_HUMAN	194 kDa	2	0	0	0	0	0	0	0	0
Synaptonemal complex protein 2-like	SYC2L_HUMAN	94 kDa	2	0	0	0	0	0	0	0	0
Paraspeckle component 1	PSPC1_HUMAN	59 kDa	2	0	0	0	0	0	0	0	0
ATPase family AAA domain-containing protein 3A	ATD3A_HUMAN	71 kDa	2	0	0	0	0	0	0	0	0
Ras-specific guanine nucleotide-releasing factor 2	RGRF2_HUMAN	141 kDa	2	0	0	0	0	0	0	0	0
Coiled-coil-helix-coiled-coil-helix domain-containing protein 6	CHCH6_HUMAN	26 kDa	2	0	0	0	0	0	0	0	0
Contactin-associated protein-like 2	CNTP2_HUMAN	148 kDa	2	0	0	0	0	0	0	0	0
Peroxisomal membrane protein 11B	PX11B_HUMAN	28 kDa	2	0	0	0	0	0	0	0	0
Disintegrin and metalloproteinase domain-containing protein 22	ADA22_HUMAN	100 kDa	2	0	0	0	0	0	0	0	0
Ubiquitin-like protein 4A	UBL4A_HUMAN	18 kDa	2	0	0	0	0	0	0	0	0
Epidermal growth factor receptor substrate 15-like 1	EP15R_HUMAN	94 kDa	2	0	0	0	0	0	0	0	0
Metabotropic glutamate receptor 3	GRM3_HUMAN	99 kDa	2	0	0	0	0	0	0	0	0
Voltage-gated potassium channel subunit beta-1	KCAB1_HUMAN	47 kDa	2	0	0	0	0	0	0	0	0
28S ribosomal protein S16	RT16_HUMAN	15 kDa	2	0	0	0	0	0	0	0	0
Ras-related protein Rab-22A	RB22A_HUMAN	22 kDa	2	0	0	0	0	0	0	0	0
GTP-binding protein Rheb	RHEB_HUMAN	20 kDa	2	0	0	0	0	0	0	0	0
DnaJ homolog subfamily B member 6	DNJB6_HUMAN	36 kDa	2	0	0	0	0	0	0	0	0
Ras-related protein Rab-23	RAB23_HUMAN	27 kDa	2	0	0	0	0	0	0	0	0
Hyaluronan and proteoglycan link protein 4	HPLN4_HUMAN	43 kDa	2	0	0	0	0	0	0	0	0
Small glutamine-rich tetratricopeptide repeat-containing protein beta	SGTB_HUMAN	33 kDa	2	0	0	0	0	0	0	0	0
D-tyrosyl-tRNA(Tyr) deacylase 1	DTD1_HUMAN	23 kDa	2	0	0	0	0	0	0	0	0
A-kinase anchor protein 5	AKAP5_HUMAN	47 kDa	2	0	0	0	0	0	0	0	0
Dynactin subunit 5	DCTN5_HUMAN	20 kDa	2	0	0	0	0	0	0	0	0
V-type proton ATPase subunit G 1	VATG1_HUMAN	14 kDa	2	0	0	0	0	0	0	0	0
ADP-ribosylation factor-like protein 15	ARL15_HUMAN	23 kDa	2	0	0	0	0	0	0	0	0
Stromal membrane-associated protein 1	SMAP1_HUMAN	50 kDa	2	0	0	0	0	0	0	0	0
N-terminal EF-hand calcium-binding protein 1	NECA1_HUMAN	41 kDa	2	0	0	0	0	0	0	0	0
Dynein light chain roadblock-type 1	DLRB1_HUMAN	11 kDa	2	0	0	0	0	0	0	0	0
Prosaposin	SAP_HUMAN	58 kDa	2	0	0	0	0	0	0	0	0
Ubiquilin-1	UBQL1_HUMAN	63 kDa	2	0	0	0	0	0	0	0	0
U6 snRNA-associated Sm-like protein LSm8	LSM8_HUMAN	10 kDa	2	0	0	0	0	0	0	0	0
LYR motif-containing protein 4	LYRM4_HUMAN	11 kDa	2	0	0	0	0	0	0	0	0
Copper transport protein ATOX1	ATOX1_HUMAN	7 kDa	2	0	0	0	0	0	0	0	0
Huntingtin	HD_HUMAN	348 kDa	2	0	0	0	0	0	0	0	0
Guanine nucleotide-binding protein-like 1	GNL1_HUMAN	69 kDa	2	0	0	0	0	0	0	0	0
Intersectin-1	ITSN1_HUMAN	195 kDa	2	0	0	0	0	0	0	0	0
Parvalbumin alpha	PRVA_HUMAN	12 kDa	2	0	0	0	0	0	0	0	0
Glucosamine-6-phosphate isomerase 1	GNPI1_HUMAN	33 kDa	2	0	0	0	0	0	0	0	0
AMP deaminase 2	AMPD2_HUMAN	101 kDa	1	0	0	0	1	0	0	0	0

Description	Accession	MW	Raw spectral counts								
			Frontal Cortex	Cerebellum	Right Ventricle	Mesentric lymph node	Liver	Pancreas	Proximal bile duct	Penis	Prostate
Phenylalanine-4-hydroxylase	PH4H_HUMAN	52 kDa	0	0	0	0	2	0	0	0	0
Cytochrome P450 3A5	CP3A5_HUMAN	57 kDa	0	0	0	0	2	0	0	0	0
Peroxisomal sarcosine oxidase	SOX_HUMAN	44 kDa	0	0	0	0	2	0	0	0	0
Pyruvate kinase PKLR	KPYR_HUMAN	62 kDa	0	0	0	0	2	0	0	0	0
Ectonucleoside triphosphate diphosphohydrolase 5	ENTP5_HUMAN	48 kDa	0	0	0	0	2	0	0	0	0
Acyl-coenzyme A synthetase ACSM5	ACSM5_HUMAN	65 kDa	0	0	0	0	2	0	0	0	0
UDP-glucuronosyltransferase 2B15	UDB15_HUMAN	61 kDa	0	0	0	0	2	0	0	0	0
Very long-chain acyl-CoA synthetase	S27A2_HUMAN	70 kDa	0	0	0	0	2	0	0	0	0
Methyltransferase-like protein 7B	MET7B_HUMAN	28 kDa	0	0	0	0	2	0	0	0	0
Tetratricopeptide repeat protein 36	TTC36_HUMAN	21 kDa	0	0	0	0	2	0	0	0	0
SEC14-like protein 4	S14L4_HUMAN	47 kDa	0	0	0	0	2	0	0	0	0
Glutaryl-CoA dehydrogenase	GCDH_HUMAN	48 kDa	0	0	0	0	2	0	0	0	0
1,2-dihydroxy-3-keto-5-methylthiopentene dioxygenase	MTND_HUMAN	21 kDa	0	0	0	0	2	0	0	0	0
Calcium-regulated heat stable protein 1	CHSP1_HUMAN	16 kDa	0	0	0	0	2	0	0	0	0
Mitochondrial dicarboxylate carrier	DIC_HUMAN	31 kDa	0	0	0	0	2	0	0	0	0
UDP-glucuronosyltransferase 2A3	UD2A3_HUMAN	60 kDa	0	0	0	0	2	0	0	0	0
Serine dehydratase-like	SDSL_HUMAN	35 kDa	0	0	0	0	2	0	0	0	0
Ubiquitin conjugation factor E4 A	UBE4A_HUMAN	123 kDa	0	0	0	0	2	0	0	0	0
Extracellular glycoprotein lacritin	LACRT_HUMAN	14 kDa	0	0	0	0	2	0	0	0	0
Aminoacylase-1	ACY1_HUMAN	46 kDa	0	0	0	0	2	0	0	0	0
Ubiquitin-like modifier-activating enzyme 6	UBA6_HUMAN	118 kDa	1	0	0	1	0	0	0	0	0
Protein tyrosine phosphatase type IVA 2	TP4A2_HUMAN	19 kDa	1	0	0	1	0	0	0	0	0
Aspartyl/asparaginyl beta-hydroxylase	ASPH_HUMAN	86 kDa	0	0	0	2	0	0	0	0	0
M-phase phosphoprotein 9	MPP9_HUMAN	133 kDa	0	0	0	2	0	0	0	0	0
Janus kinase and microtubule-interacting protein 2	JKIP2_HUMAN	95 kDa	0	0	0	2	0	0	0	0	0
Glycogen [starch] synthase, muscle	GYS1_HUMAN	84 kDa	0	0	0	2	0	0	0	0	0
Solute carrier family 2, facilitated glucose transporter member 4	GTR4_HUMAN	55 kDa	0	0	0	2	0	0	0	0	0
TBC1 domain family member 10B	TB10B_HUMAN	87 kDa	1	0	0	0	0	0	1	0	0
Zyxin	ZYX_HUMAN	61 kDa	0	0	0	1	0	0	1	0	0
Coagulation factor XIII A chain	F13A_HUMAN	83 kDa	0	0	0	1	0	0	1	0	0
Microfibril-associated glycoprotein 4	MFAP4_HUMAN	29 kDa	0	0	0	1	0	0	1	0	0
Diacylglycerol kinase theta	DGKQ_HUMAN	101 kDa	0	0	0	0	0	0	2	0	0
Fibulin-1	FBLN1_HUMAN	77 kDa	0	0	0	0	0	0	2	0	0
Inter-alpha-trypsin inhibitor heavy chain H2	ITIH2_HUMAN	106 kDa	0	0	0	0	0	0	2	0	0
Tryptase delta	TRYD_HUMAN	27 kDa	0	0	0	0	0	0	2	0	0
Protocadherin Fat 2	FAT2_HUMAN	479 kDa	1	0	0	0	0	1	0	0	0
Small nuclear ribonucleoprotein Sm D3	SMD3_HUMAN	14 kDa	1	0	0	0	0	1	0	0	0
Bifunctional 3'-phosphoadenosine 5'-phosphosulfate synthase 2	PAPS2_HUMAN	70 kDa	0	0	0	0	1	1	0	0	0
2-amino-3-ketobutyrate coenzyme A ligase	KBL_HUMAN	45 kDa	0	0	0	0	1	1	0	0	0
Transmembrane 9 superfamily member 4	TM9S4_HUMAN	75 kDa	0	0	0	0	0	2	0	0	0
Receptor-type tyrosine-protein phosphatase U	PTPRU_HUMAN	162 kDa	0	0	0	0	0	2	0	0	0
Cyclin-dependent kinase 17	CDK17_HUMAN	60 kDa	0	0	0	0	0	2	0	0	0
Fibronectin type-III domain-containing protein 3A	FND3A_HUMAN	132 kDa	0	0	0	0	0	2	0	0	0
Heat shock 70 kDa protein 13	HSP13_HUMAN	52 kDa	0	0	0	0	0	2	0	0	0
Cornulin	CRNN_HUMAN	54 kDa	0	0	0	0	0	2	0	0	0
GDP-mannose 4,6 dehydratase	GMDS_HUMAN	42 kDa	0	0	0	0	0	2	0	0	0
Inosine-5'-monophosphate dehydrogenase 2	IMDH2_HUMAN	56 kDa	0	0	0	0	0	2	0	0	0
Probable D-tyrosyl-tRNA(Tyr) deacylase 2	DTD2_HUMAN	19 kDa	0	0	0	0	0	2	0	0	0
Cysteine--tRNA ligase, cytoplasmic	SYCC_HUMAN	85 kDa	0	0	0	0	0	2	0	0	0
Protein transport protein Sec61 subunit beta	SC61B_HUMAN	10 kDa	0	0	0	0	0	2	0	0	0

Description	Accession	MW	Raw spectral counts								
			Frontal Cortex	Cerebellum	Right Ventricle	Mesentric lymph node	Liver	Pancreas	Proximal bile duct	Penis	Prostate
PCTP-like protein	PCTL_HUMAN	33 kDa	0	0	0	0	0	2	0	0	0
Chymotrypsinogen B	CTRB1_HUMAN	28 kDa	0	0	0	0	0	2	0	0	0
Multiple coagulation factor deficiency protein 2	MCFD2_HUMAN	16 kDa	0	0	0	0	0	2	0	0	0
UDP-N-acetylhexosamine pyrophosphorylase	UAP1_HUMAN	59 kDa	0	0	0	0	0	2	0	0	0
Carboxypeptidase A2	CBPA2_HUMAN	47 kDa	0	0	0	0	0	2	0	0	0
DDRGK domain-containing protein 1	DDRGK_HUMAN	36 kDa	0	0	0	0	0	2	0	0	0
Secretagogin	SEGN_HUMAN	32 kDa	0	0	0	0	0	2	0	0	0
Dolichyl-diphosphooligosaccharide--protein glycosyltransferase subunit STT3B	STT3B_HUMAN	94 kDa	0	0	0	0	0	2	0	0	0
Clusterin	CLUS_HUMAN	52 kDa	1	0	0	0	0	0	0	1	0
Adapter molecule crk	CRK_HUMAN	34 kDa	1	0	0	0	0	0	0	1	0
Exonuclease 1	EXO1_HUMAN	94 kDa	1	0	0	0	0	0	0	1	0
Fatty acid-binding protein, epidermal	FABP5_HUMAN	15 kDa	1	0	0	0	0	0	0	1	0
COP9 signalosome complex subunit 1	CSN1_HUMAN	56 kDa	1	0	0	0	0	0	0	1	0
Phosphoglycolate phosphatase	PGP_HUMAN	34 kDa	1	0	0	0	0	0	0	1	0
Cystatin-C	CYTC_HUMAN	16 kDa	1	0	0	0	0	0	0	1	0
Xaa-Pro aminopeptidase 1	XPP1_HUMAN	70 kDa	1	0	0	0	0	0	0	1	0
Ferrochelatase	HEMH_HUMAN	48 kDa	1	0	0	0	0	0	0	1	0
Bifunctional coenzyme A synthase	COASY_HUMAN	62 kDa	0	0	0	0	1	0	0	1	0
Serum amyloid P-component	SAMP_HUMAN	25 kDa	0	0	0	0	1	0	0	1	0
Actin-related protein 2/3 complex subunit 1B	ARC1B_HUMAN	41 kDa	0	0	0	1	0	0	0	1	0
Colorectal mutant cancer protein	CRCM_HUMAN	93 kDa	0	0	0	1	0	0	0	1	0
Ig kappa chain V-I region DEE	KV105_HUMAN	12 kDa	0	0	0	1	0	0	0	1	0
Transcriptional repressor p66-beta	P66B_HUMAN	65 kDa	0	0	0	0	0	0	1	1	0
Tetranectin	TETN_HUMAN	23 kDa	0	0	0	0	0	0	1	1	0
Latexin	LXN_HUMAN	26 kDa	0	0	0	0	0	1	0	1	0
Aminopeptidase B	AMPB_HUMAN	73 kDa	0	0	0	0	0	1	0	1	0
Phosphoglucomutase-2	PGM2_HUMAN	68 kDa	0	0	0	0	0	1	0	1	0
Small proline-rich protein 3	SPRR3_HUMAN	18 kDa	0	0	0	0	0	1	0	1	0
Phosphomannomutase 2	PMM2_HUMAN	28 kDa	0	0	0	0	0	1	0	1	0
Protein CutA	CUTA_HUMAN	19 kDa	0	0	0	0	0	1	0	1	0
Regulation of nuclear pre-mRNA domain-containing protein 1B	RPR1B_HUMAN	37 kDa	0	0	0	0	0	0	0	2	0
Ubiquitin carboxyl-terminal hydrolase isozyme L3	UCHL3_HUMAN	26 kDa	0	0	0	0	0	0	0	2	0
Nucleosome assembly protein 1-like 1	NP1L1_HUMAN	45 kDa	0	0	0	0	0	0	0	2	0
Apoptosis regulator BAX	BAX_HUMAN	21 kDa	0	0	0	0	0	0	0	2	0
High mobility group protein B3	HMGB3_HUMAN	23 kDa	0	0	0	0	0	0	0	2	0
U2 small nuclear ribonucleoprotein B''	RU2B_HUMAN	25 kDa	0	0	0	0	0	0	0	2	0
ADP-ribosylation factor-like protein 2	ARL2_HUMAN	21 kDa	0	0	0	0	0	0	0	2	0
Collagen alpha-2(I) chain	CO1A2_HUMAN	129 kDa	0	0	0	0	0	0	0	2	0
H/ACA ribonucleoprotein complex subunit 2	NHP2_HUMAN	17 kDa	0	0	0	0	0	0	0	2	0
ATP-dependent 6-phosphofructokinase, liver type	PFKAL_HUMAN	85 kDa	0	0	0	0	0	0	0	2	0
E3 ubiquitin-protein ligase TRIP12	TRIPC_HUMAN	220 kDa	0	0	0	0	0	0	0	2	0
CAD protein	PYR1_HUMAN	243 kDa	0	0	0	0	0	0	0	2	0
Epidermal growth factor receptor	EGFR_HUMAN	134 kDa	0	0	0	0	0	0	0	2	0
RNA-binding protein 25	RBM25_HUMAN	100 kDa	0	0	0	0	0	0	0	2	0
Eukaryotic initiation factor 4A-III	IF4A3_HUMAN	47 kDa	0	0	0	0	0	0	0	2	0
Nicotinate phosphoribosyltransferase	PNCB_HUMAN	58 kDa	0	0	0	0	0	0	0	2	0
Regulator of nonsense transcripts 1	RENT1_HUMAN	124 kDa	0	0	0	0	0	0	0	2	0
Cellular retinoic acid-binding protein 2	RABP2_HUMAN	16 kDa	0	0	0	0	0	0	0	2	0
182 kDa tankyrase-1-binding protein	TB182_HUMAN	182 kDa	0	0	0	0	0	0	0	2	0
Density-regulated protein	DENR_HUMAN	22 kDa	0	0	0	0	0	0	0	2	0

Description	Accession	MW	Raw spectral counts								
			Frontal Cortex	Cerebellum	Right Ventricle	Mesentric lymph node	Liver	Pancreas	Proximal bile duct	Penis	Prostate
26S proteasome non-ATPase regulatory subunit 9	PSMD9_HUMAN	25 kDa	0	0	0	0	0	0	0	2	0
Tetratricopeptide repeat protein 37	TTC37_HUMAN	175 kDa	0	0	0	0	0	0	0	2	0
DNA-directed RNA polymerase II subunit RPB2	RPB2_HUMAN	134 kDa	0	0	0	0	0	0	0	2	0
Glucose-6-phosphate 1-dehydrogenase	G6PD_HUMAN	59 kDa	0	0	0	0	0	0	0	2	0
Glutamate receptor ionotropic, NMDA 2C	NMDE3_HUMAN	134 kDa	0	0	0	0	0	0	0	2	0
Glutamate--cysteine ligase catalytic subunit	GSH1_HUMAN	73 kDa	0	0	0	0	0	0	0	2	0
Cyclic GMP-AMP synthase	CGAS_HUMAN	59 kDa	0	0	0	0	0	0	0	2	0
Kinesin light chain 3	KLC3_HUMAN	55 kDa	0	0	0	0	0	0	0	2	0
Endophilin-B1	SHLB1_HUMAN	41 kDa	0	0	0	0	0	0	0	2	0
Polyadenylate-binding protein 2	PABP2_HUMAN	33 kDa	0	0	0	0	0	0	0	2	0
BRO1 domain-containing protein BROX	BROX_HUMAN	46 kDa	0	0	0	0	0	0	0	2	0
Peptidyl-prolyl cis-trans isomerase FKBP5	FKBP5_HUMAN	51 kDa	0	0	0	0	0	0	0	2	0
Vacuolar protein sorting-associated protein 4B	VPS4B_HUMAN	49 kDa	0	0	0	0	0	0	0	2	0
PDZ domain-containing protein GIPC1	GIPC1_HUMAN	36 kDa	0	0	0	0	0	0	0	2	0
Protein PBDC1	PBDC1_HUMAN	26 kDa	0	0	0	0	0	0	0	2	0
Serine-threonine kinase receptor-associated protein	STRAP_HUMAN	38 kDa	0	0	0	0	0	0	0	2	0
Nucleobindin-1	NUCB1_HUMAN	54 kDa	0	0	0	0	0	0	0	2	0
Rho guanine nucleotide exchange factor 10-like protein	ARGAL_HUMAN	140 kDa	0	0	0	0	0	0	0	2	0
Serpin B7	SPB7_HUMAN	43 kDa	0	0	0	0	0	0	0	2	0
Retroviral-like aspartic protease 1	APRV1_HUMAN	37 kDa	0	0	0	0	0	0	0	2	0
Ubiquitin fusion degradation protein 1 homolog	UFD1_HUMAN	35 kDa	0	0	0	0	0	0	0	2	0
Three prime repair exonuclease 2	TREX2_HUMAN	31 kDa	0	0	0	0	0	0	0	2	0
Emerin	EMD_HUMAN	29 kDa	0	0	0	0	0	0	0	2	0
Secreted frizzled-related protein 1	SFRP1_HUMAN	35 kDa	0	0	0	0	0	0	0	2	0
Protein phosphatase methylesterase 1	PPME1_HUMAN	42 kDa	0	0	0	0	0	0	0	2	0
F-box only protein 50	FBX50_HUMAN	31 kDa	0	0	0	0	0	0	0	2	0
Ragulator complex protein LAMTOR1	LTOR1_HUMAN	18 kDa	0	0	0	0	0	0	0	2	0
15-hydroxyprostaglandin dehydrogenase [NAD(+)]	PGDH_HUMAN	29 kDa	0	0	0	0	0	0	0	2	0
Transcription elongation factor A protein 1	TCEA1_HUMAN	34 kDa	0	0	0	0	0	0	0	2	0
Sterol-4-alpha-carboxylate 3-dehydrogenase, decarboxylating	NSDHL_HUMAN	42 kDa	0	0	0	0	0	0	0	2	0
Putative hydroxypyruvate isomerase	HYI_HUMAN	30 kDa	0	0	0	0	0	0	0	2	0
DCC-interacting protein 13-alpha	DP13A_HUMAN	80 kDa	0	0	0	0	0	0	0	2	0
Histone H3.1	H31_HUMAN	15 kDa	0	0	0	0	0	0	0	2	0
Sister chromatid cohesion protein PDS5 homolog B	PDS5B_HUMAN	165 kDa	0	0	0	0	0	0	0	2	0
Ribose-phosphate pyrophosphokinase 2	PRPS2_HUMAN	35 kDa	0	0	0	0	0	0	0	2	0
Protein S100-A14	S10AE_HUMAN	12 kDa	0	0	0	0	0	0	0	2	0
5'-nucleotidase	5NTD_HUMAN	63 kDa	0	0	0	0	0	0	0	2	0
Dermatopontin	DERM_HUMAN	24 kDa	0	0	0	0	0	0	0	2	0
Pigment epithelium-derived factor	PEDF_HUMAN	46 kDa	0	0	0	0	0	0	0	2	0
26S proteasome non-ATPase regulatory subunit 5	PSMD5_HUMAN	56 kDa	0	0	0	0	0	0	0	2	0
Periaxin	PRAX_HUMAN	155 kDa	0	0	0	0	0	0	0	2	0
Protein phosphatase 1A	PPM1A_HUMAN	42 kDa	1	0	0	0	0	0	0	0	1
Phosphatidylinositide phosphatase SAC1	SAC1_HUMAN	67 kDa	1	0	0	0	0	0	0	0	1
Alpha-mannosidase 2C1	MA2C1_HUMAN	116 kDa	0	0	0	0	1	0	0	0	1
Ester hydrolase C11orf54	CK054_HUMAN	35 kDa	0	0	0	0	1	0	0	0	1
Retinol dehydrogenase 11	RDH11_HUMAN	35 kDa	0	0	0	0	1	0	0	0	1
Zinc finger and BTB domain-containing protein 8A	ZBT8A_HUMAN	50 kDa	0	0	0	0	0	0	1	0	1
Translocation protein SEC63 homolog	SEC63_HUMAN	88 kDa	0	0	0	0	0	1	0	0	1
Rho-associated protein kinase 1	ROCK1_HUMAN	158 kDa	0	0	0	0	0	0	0	1	1
Cadherin-1	CADH1_HUMAN	97 kDa	0	0	0	0	0	0	0	1	1

Description	Accession	MW	Raw spectral counts								
			Frontal Cortex	Cerebellum	Right Ventricle	Mesentric lymph node	Liver	Pancreas	Proximal bile duct	Penis	Prostate
Eukaryotic translation initiation factor 3 subunit L	EIF3L_HUMAN	67 kDa	0	0	0	0	0	0	0	1	1
Integrin alpha-5	ITA5_HUMAN	115 kDa	0	0	0	0	0	0	0	0	2
Aspartoacylase	ACY2_HUMAN	36 kDa	0	0	0	0	0	0	0	0	2
Neutrophil gelatinase-associated lipocalin	NGAL_HUMAN	23 kDa	0	0	0	0	0	0	0	0	2
Multiple PDZ domain protein	MPDZ_HUMAN	222 kDa	0	0	0	0	0	0	0	0	2
Epididymis-specific alpha-mannosidase	MA2B2_HUMAN	114 kDa	0	0	0	0	0	0	0	0	2
PDZ and LIM domain protein 7	PDLI7_HUMAN	50 kDa	0	0	0	0	0	0	0	0	2
Solute carrier family 35 member F6	S35F6_HUMAN	40 kDa	0	0	0	0	0	0	0	0	2
NHL repeat-containing protein 3	NHLC3_HUMAN	38 kDa	0	0	0	0	0	0	0	0	2
Transmembrane emp24 domain-containing protein 7	TMED7_HUMAN	25 kDa	0	0	0	0	0	0	0	0	2
Protein CREG1	CREG1_HUMAN	24 kDa	0	0	0	0	0	0	0	0	2
Protein FAM84A	FA84A_HUMAN	32 kDa	0	0	0	0	0	0	0	0	2
Beta-hexosaminidase subunit alpha	HEXA_HUMAN	61 kDa	0	0	0	0	0	0	0	0	2
Sodium-dependent noradrenaline transporter	SC6A2_HUMAN	69 kDa	0	0	0	0	0	0	0	0	2
NAD-dependent protein deacylase sirtuin-5	SIR5_HUMAN	34 kDa	1	0	1	0	0	0	0	0	0
39S ribosomal protein L13	RM13_HUMAN	21 kDa	1	0	1	0	0	0	0	0	0
Protein NDRG4	NDRG4_HUMAN	38 kDa	1	0	1	0	0	0	0	0	0
NADH dehydrogenase [ubiquinone] 1 subunit C2	NDUC2_HUMAN	14 kDa	1	0	1	0	0	0	0	0	0
Mitochondrial import receptor subunit TOM5 homolog	TOM5_HUMAN	6 kDa	1	0	1	0	0	0	0	0	0
Small integral membrane protein 20	SMI20_HUMAN	18 kDa	1	0	1	0	0	0	0	0	0
Putative cytochrome c oxidase subunit 7A3	COX7S_HUMAN	12 kDa	1	0	1	0	0	0	0	0	0
Cytochrome c oxidase assembly factor 3 homolog	COA3_HUMAN	12 kDa	1	0	1	0	0	0	0	0	0
Cullin-1	CUL1_HUMAN	90 kDa	1	0	1	0	0	0	0	0	0
Cytochrome c oxidase subunit 7C	COX7C_HUMAN	7 kDa	1	0	1	0	0	0	0	0	0
Dynein light chain 2, cytoplasmic	DYL2_HUMAN	10 kDa	1	0	1	0	0	0	0	0	0
COP9 signalosome complex subunit 3	CSN3_HUMAN	48 kDa	1	0	1	0	0	0	0	0	0
Carbonyl reductase family member 4	CBR4_HUMAN	25 kDa	0	0	1	0	1	0	0	0	0
Hydroxymethylglutaryl-CoA lyase	HMGCL_HUMAN	34 kDa	0	0	1	0	1	0	0	0	0
Serpin B9	SPB9_HUMAN	42 kDa	0	0	1	1	0	0	0	0	0
Collagen alpha-1(XV) chain	COFA1_HUMAN	142 kDa	0	0	1	1	0	0	0	0	0
A-kinase anchor protein 9	AKAP9_HUMAN	454 kDa	0	0	1	0	0	0	1	0	0
Utrophin	UTRO_HUMAN	394 kDa	0	0	1	0	0	1	0	0	0
Transmembrane protein 14C	TM14C_HUMAN	12 kDa	0	0	1	0	0	1	0	0	0
Alpha-1B-glycoprotein	A1BG_HUMAN	54 kDa	0	0	1	0	0	1	0	0	0
Polyribonucleotide nucleotidyltransferase 1	PNPT1_HUMAN	86 kDa	0	0	1	0	0	1	0	0	0
COMM domain-containing protein 2	COMD2_HUMAN	23 kDa	0	0	1	0	0	0	0	1	0
UV excision repair protein RAD23 homolog A	RD23A_HUMAN	40 kDa	0	0	1	0	0	0	0	1	0
E3 ubiquitin-protein ligase UBR4	UBR4_HUMAN	574 kDa	0	0	1	0	0	0	0	1	0
Protein unc-13 homolog C	UN13C_HUMAN	251 kDa	0	0	1	0	0	0	0	1	0
Rab11 family-interacting protein 1	RFIP1_HUMAN	137 kDa	0	0	1	0	0	0	0	1	0
39S ribosomal protein L9	RM09_HUMAN	30 kDa	0	0	1	0	0	0	0	1	0
Heat shock protein beta-8	HSPB8_HUMAN	22 kDa	0	0	1	0	0	0	0	1	0
Protein MEMO1	MEMO1_HUMAN	34 kDa	0	0	1	0	0	0	0	1	0
Lipoamide acyltransferase component of branched-chain alpha-keto acid dehydrogenase	ODB2_HUMAN	53 kDa	0	0	1	0	0	0	0	1	0
Dysferlin	DYSF_HUMAN	237 kDa	0	0	1	0	0	0	0	0	1
Twinfilin-2	TWF2_HUMAN	40 kDa	0	0	2	0	0	0	0	0	0
Prostaglandin reductase 2	PTGR2_HUMAN	38 kDa	0	0	2	0	0	0	0	0	0
Acyl-coenzyme A thioesterase 1	ACOT1_HUMAN	46 kDa	0	0	2	0	0	0	0	0	0
Glycerol kinase	GLPK_HUMAN	61 kDa	0	0	2	0	0	0	0	0	0
Collagen alpha-6(VI) chain	CO6A6_HUMAN	247 kDa	0	0	2	0	0	0	0	0	0

Description	Accession	MW	Raw spectral counts								
			Frontal Cortex	Cerebellum	Right Ventricle	Mesentric lymph node	Liver	Pancreas	Proximal bile duct	Penis	Prostate
Thioredoxin	THIOM_HUMAN	18 kDa	0	0	2	0	0	0	0	0	0
Obscurin	OBSCN_HUMAN	868 kDa	0	0	2	0	0	0	0	0	0
Calcium/calmodulin-dependent 3',5'-cyclic nucleotide phosphodiesterase 1C	PDE1C_HUMAN	81 kDa	0	0	2	0	0	0	0	0	0
Smoothelin	SMTN_HUMAN	99 kDa	0	0	2	0	0	0	0	0	0
39S ribosomal protein L1	RM01_HUMAN	37 kDa	0	0	2	0	0	0	0	0	0
Alanine aminotransferase 2	ALAT2_HUMAN	58 kDa	0	0	2	0	0	0	0	0	0
Cytochrome c oxidase assembly factor 1 homolog	COA1_HUMAN	17 kDa	0	0	2	0	0	0	0	0	0
Apolipoprotein O-like	APOOL_HUMAN	29 kDa	0	0	2	0	0	0	0	0	0
39S ribosomal protein L2	RM02_HUMAN	33 kDa	0	0	2	0	0	0	0	0	0
Surfeit locus protein 1	SURF1_HUMAN	33 kDa	0	0	2	0	0	0	0	0	0
Caveolin-3	CAV3_HUMAN	17 kDa	0	0	2	0	0	0	0	0	0
Acetolactate synthase-like protein	ILVBL_HUMAN	68 kDa	0	0	2	0	0	0	0	0	0
Coenzyme Q-binding protein COQ10 homolog B	CQ10B_HUMAN	27 kDa	0	0	2	0	0	0	0	0	0
Caveolin-2	CAV2_HUMAN	18 kDa	0	0	2	0	0	0	0	0	0
39S ribosomal protein L39	RM39_HUMAN	39 kDa	0	0	2	0	0	0	0	0	0
Cysteine and glycine-rich protein 3	CSRP3_HUMAN	21 kDa	0	0	2	0	0	0	0	0	0
Iron-sulfur cluster co-chaperone protein HscB	HSC20_HUMAN	27 kDa	0	0	2	0	0	0	0	0	0
39S ribosomal protein L24	RM24_HUMAN	25 kDa	0	0	2	0	0	0	0	0	0
5'-AMP-activated protein kinase subunit beta-2	AAKB2_HUMAN	30 kDa	0	0	2	0	0	0	0	0	0
39S ribosomal protein L48	RM48_HUMAN	24 kDa	0	0	2	0	0	0	0	0	0
Ig kappa chain V-I region BAN	KV122_HUMAN	12 kDa	0	0	2	0	0	0	0	0	0
E3 UFM1-protein ligase 1	UFL1_HUMAN	90 kDa	0	0	2	0	0	0	0	0	0
Mitochondrial-processing peptidase subunit beta	MPPB_HUMAN	54 kDa	0	0	2	0	0	0	0	0	0
Integrin alpha-7	ITA7_HUMAN	129 kDa	0	0	2	0	0	0	0	0	0
NFU1 iron-sulfur cluster scaffold homolog	NFU1_HUMAN	28 kDa	0	0	2	0	0	0	0	0	0
NAD-dependent protein deacetylase sirtuin-3	SIR3_HUMAN	44 kDa	0	0	2	0	0	0	0	0	0
Gamma-sarcoglycan	SGCG_HUMAN	32 kDa	0	0	2	0	0	0	0	0	0
Reticulon-4-interacting protein 1	RT4I1_HUMAN	44 kDa	0	0	2	0	0	0	0	0	0
Ubiquinol-cytochrome-c reductase complex assembly factor 1	UQCC1_HUMAN	35 kDa	0	0	2	0	0	0	0	0	0
Protein NipSnap homolog 3B	NPS3B_HUMAN	28 kDa	0	0	2	0	0	0	0	0	0
Apolipoprotein C-II	APOC2_HUMAN	11 kDa	0	0	2	0	0	0	0	0	0
Delta-1-pyrroline-5-carboxylate synthase	P5CS_HUMAN	87 kDa	0	0	2	0	0	0	0	0	0
Presequence protease	PREP_HUMAN	117 kDa	0	0	2	0	0	0	0	0	0
Alpha-sarcoglycan	SGCA_HUMAN	43 kDa	0	0	2	0	0	0	0	0	0
39S ribosomal protein L43	RM43_HUMAN	23 kDa	0	0	2	0	0	0	0	0	0
Plasminogen receptor (KT)	PLRKT_HUMAN	17 kDa	0	0	2	0	0	0	0	0	0
Coenzyme Q-binding protein COQ10 homolog A	CQ10A_HUMAN	28 kDa	0	0	2	0	0	0	0	0	0
Guanine nucleotide-binding protein subunit alpha-13	GNA13_HUMAN	44 kDa	0	1	0	0	0	0	0	0	0
Protein FAM98B	FA98B_HUMAN	37 kDa	0	1	0	0	0	0	0	0	0
Inactive phospholipase C-like protein 2	PLCL2_HUMAN	126 kDa	0	1	0	0	0	0	0	0	0
Liprin-alpha-1	LIPA1_HUMAN	136 kDa	0	1	0	0	0	0	0	0	0
Exportin-7	XPO7_HUMAN	124 kDa	0	1	0	0	0	0	0	0	0
Protocadherin-12	PCD12_HUMAN	129 kDa	0	1	0	0	0	0	0	0	0
Latrophilin-3	LPHN3_HUMAN	162 kDa	0	1	0	0	0	0	0	0	0
Abnormal spindle-like microcephaly-associated protein	ASPM_HUMAN	410 kDa	0	1	0	0	0	0	0	0	0
Pericentrin	PCNT_HUMAN	378 kDa	0	1	0	0	0	0	0	0	0
Protein-tyrosine kinase 2-beta	FAK2_HUMAN	116 kDa	0	1	0	0	0	0	0	0	0
Synaptonemal complex protein 2	SYCP2_HUMAN	176 kDa	0	1	0	0	0	0	0	0	0
Nucleoporin NUP188 homolog	NU188_HUMAN	196 kDa	0	1	0	0	0	0	0	0	0
Zinc finger protein 432	ZN432_HUMAN	75 kDa	0	1	0	0	0	0	0	0	0

Description	Accession	MW	Raw spectral counts								
			Frontal Cortex	Cerebellum	Right Ventricle	Mesentric lymph node	Liver	Pancreas	Proximal bile duct	Penis	Prostate
Serine/threonine-protein kinase WNK2	WNK2_HUMAN	243 kDa	0	1	0	0	0	0	0	0	0
Spermatogenesis-associated protein 31D1	S31D1_HUMAN	176 kDa	0	1	0	0	0	0	0	0	0
Voltage-dependent calcium channel subunit alpha-2/delta-2	CA2D2_HUMAN	130 kDa	0	1	0	0	0	0	0	0	0
Pleckstrin homology-like domain family B member 2	PHLB2_HUMAN	142 kDa	0	1	0	0	0	0	0	0	0
Phosphatidylinositol 4-phosphate 3-kinase C2 domain-containing subunit alpha	P3C2A_HUMAN	191 kDa	0	1	0	0	0	0	0	0	0
FRAS1-related extracellular matrix protein 3	FREM3_HUMAN	238 kDa	0	1	0	0	0	0	0	0	0
Neuronal pentraxin-1	NPTX1_HUMAN	47 kDa	0	1	0	0	0	0	0	0	0
NEDD4-binding protein 2-like 2	N42L2_HUMAN	67 kDa	0	1	0	0	0	0	0	0	0
Unconventional myosin-VIIb	MYO7B_HUMAN	242 kDa	0	1	0	0	0	0	0	0	0
Calcium/calmodulin-dependent protein kinase type IV	KCC4_HUMAN	52 kDa	0	1	0	0	0	0	0	0	0
Putative spermatogenesis-associated protein 31D3	S31D3_HUMAN	102 kDa	0	1	0	0	0	0	0	0	0
ATPase family AAA domain-containing protein 2B	ATD2B_HUMAN	165 kDa	0	1	0	0	0	0	0	0	0
Growth/differentiation factor 8	GDF8_HUMAN	43 kDa	0	1	0	0	0	0	0	0	0
Rho guanine nucleotide exchange factor 33	ARG33_HUMAN	95 kDa	0	1	0	0	0	0	0	0	0
Zinc finger protein 217	ZN217_HUMAN	115 kDa	0	1	0	0	0	0	0	0	0
Ketosamine-3-kinase	KT3K_HUMAN	34 kDa	0	1	0	0	0	0	0	0	0
Putative ATP-dependent RNA helicase DHX30	DHX30_HUMAN	134 kDa	0	1	0	0	0	0	0	0	0
Ras-related protein Rab-37	RAB37_HUMAN	25 kDa	0	1	0	0	0	0	0	0	0
Transcription elongation factor SPT5	SPT5H_HUMAN	121 kDa	0	1	0	0	0	0	0	0	0
Plakophilin-4	PKP4_HUMAN	132 kDa	0	1	0	0	0	0	0	0	0
Epidermal growth factor receptor kinase substrate 8-like protein 2	ES8L2_HUMAN	81 kDa	0	1	0	0	0	0	0	0	0
Kv channel-interacting protein 4	KCIP4_HUMAN	29 kDa	0	1	0	0	0	0	0	0	0
Protein mago nashi homolog	MGN_HUMAN	17 kDa	0	1	0	0	0	0	0	0	0
Glutamate receptor ionotropic, delta-2	GRID2_HUMAN	113 kDa	0	1	0	0	0	0	0	0	0
Kinesin-like protein KIF2A	KIF2A_HUMAN	80 kDa	0	1	0	0	0	0	0	0	0
Centrosomal protein of 104 kDa	CE104_HUMAN	104 kDa	0	1	0	0	0	0	0	0	0
Ribosomal protein S6 kinase alpha-1	KS6A1_HUMAN	83 kDa	0	1	0	0	0	0	0	0	0
N-acetylserotonin O-methyltransferase-like protein	ASML_HUMAN	69 kDa	0	1	0	0	0	0	0	0	0
B-cell CLL/lymphoma 6 member B protein	BCL6B_HUMAN	52 kDa	0	1	0	0	0	0	0	0	0
Histone deacetylase complex subunit SAP18	SAP18_HUMAN	18 kDa	0	1	0	0	0	0	0	0	0
Fidgetin-like protein 1	FIGL1_HUMAN	74 kDa	0	1	0	0	0	0	0	0	0
Leucine carboxyl methyltransferase 1	LCMT1_HUMAN	38 kDa	0	1	0	0	0	0	0	0	0
Seizure 6-like protein 2	SE6L2_HUMAN	98 kDa	0	1	0	0	0	0	0	0	0
MAGUK p55 subfamily member 7	MPP7_HUMAN	66 kDa	0	1	0	0	0	0	0	0	0
Guanylate kinase	KGUA_HUMAN	22 kDa	0	1	0	0	0	0	0	0	0
Zinc finger Y-chromosomal protein	ZFY_HUMAN	91 kDa	0	1	0	0	0	0	0	0	0
Protein phosphatase 1 regulatory subunit 1B	PPR1B_HUMAN	23 kDa	0	1	0	0	0	0	0	0	0
Integrin alpha-V	ITAV_HUMAN	116 kDa	0	1	0	0	0	0	0	0	0
Sorting nexin-4	SNX4_HUMAN	52 kDa	0	1	0	0	0	0	0	0	0
Type I inositol 3,4-bisphosphate 4-phosphatase	INP4A_HUMAN	110 kDa	0	1	0	0	0	0	0	0	0
Protein piccolo	PCLO_HUMAN	553 kDa	0	1	0	0	0	0	0	0	0
Sn1-specific diacylglycerol lipase alpha	DGLA_HUMAN	115 kDa	0	1	0	0	0	0	0	0	0
Cullin-4A	CUL4A_HUMAN	88 kDa	0	1	0	0	0	0	0	0	0
Inositol 1,4,5-trisphosphate receptor type 3	ITPR3_HUMAN	304 kDa	0	1	0	0	0	0	0	0	0
Secretory carrier-associated membrane protein 5	SCAM5_HUMAN	26 kDa	0	1	0	0	0	0	0	0	0
MARCKS-related protein	MRP_HUMAN	20 kDa	1	0	0	0	0	0	0	0	0
Sorting nexin-2	SNX2_HUMAN	58 kDa	1	0	0	0	0	0	0	0	0
Malignant T-cell-amplified sequence 1	MCTS1_HUMAN	21 kDa	1	0	0	0	0	0	0	0	0
Regulator of microtubule dynamics protein 3	RMD3_HUMAN	52 kDa	1	0	0	0	0	0	0	0	0
Syntaxin-7	STX7_HUMAN	30 kDa	1	0	0	0	0	0	0	0	0

Description	Accession	MW	Raw spectral counts								
			Frontal Cortex	Cerebellum	Right Ventricle	Mesentric lymph node	Liver	Pancreas	Proximal bile duct	Penis	Prostate
Cytoplasmic dynein 1 light intermediate chain 1	DC1L1_HUMAN	57 kDa	1	0	0	0	0	0	0	0	0
TOM1-like protein 2	TM1L2_HUMAN	56 kDa	1	0	0	0	0	0	0	0	0
Microtubule cross-linking factor 1	MTCL1_HUMAN	210 kDa	1	0	0	0	0	0	0	0	0
Eukaryotic translation initiation factor 4 gamma 2	IF4G2_HUMAN	102 kDa	1	0	0	0	0	0	0	0	0
Contactin-2	CNTN2_HUMAN	113 kDa	1	0	0	0	0	0	0	0	0
WD repeat-containing protein 37	WDR37_HUMAN	55 kDa	1	0	0	0	0	0	0	0	0
Claudin-11	CLD11_HUMAN	22 kDa	1	0	0	0	0	0	0	0	0
Oligodendrocyte-myelin glycoprotein	OMGP_HUMAN	50 kDa	1	0	0	0	0	0	0	0	0
Uncharacterized protein C10orf35	CJ035_HUMAN	13 kDa	1	0	0	0	0	0	0	0	0
Mitogen-activated protein kinase kinase kinase 13	M3K13_HUMAN	108 kDa	1	0	0	0	0	0	0	0	0
Ras-related protein Rab-19	RAB19_HUMAN	24 kDa	1	0	0	0	0	0	0	0	0
Triple functional domain protein	TRIO_HUMAN	347 kDa	1	0	0	0	0	0	0	0	0
Trinucleotide repeat-containing gene 18 protein	TNC18_HUMAN	315 kDa	1	0	0	0	0	0	0	0	0
Ankyrin repeat domain-containing protein 26	ANR26_HUMAN	196 kDa	1	0	0	0	0	0	0	0	0
High affinity cAMP-specific and IBMX-insensitive 3',5'-cyclic phosphodiesterase 8B	PDE8B_HUMAN	99 kDa	1	0	0	0	0	0	0	0	0
Tonsoku-like protein	TONSL_HUMAN	151 kDa	1	0	0	0	0	0	0	0	0
Probable E3 ubiquitin-protein ligase HECTD4	HECD4_HUMAN	439 kDa	1	0	0	0	0	0	0	0	0
Protein VAC14 homolog	VAC14_HUMAN	88 kDa	1	0	0	0	0	0	0	0	0
Sodium channel protein type 10 subunit alpha	SCNAA_HUMAN	221 kDa	1	0	0	0	0	0	0	0	0
Disheveled-associated activator of morphogenesis 2	DAAM2_HUMAN	124 kDa	1	0	0	0	0	0	0	0	0
DNA-directed RNA polymerase II subunit RPB1	RPB1_HUMAN	217 kDa	1	0	0	0	0	0	0	0	0
Baculoviral IAP repeat-containing protein 6	BIRC6_HUMAN	530 kDa	1	0	0	0	0	0	0	0	0
Ubiquitin carboxyl-terminal hydrolase 34	UBP34_HUMAN	404 kDa	1	0	0	0	0	0	0	0	0
Zinc finger protein 33B	ZN33B_HUMAN	91 kDa	1	0	0	0	0	0	0	0	0
Polycystin-1	PKD1_HUMAN	463 kDa	1	0	0	0	0	0	0	0	0
Collagen alpha-3(V) chain	CO5A3_HUMAN	172 kDa	1	0	0	0	0	0	0	0	0
Afadin	AFAD_HUMAN	207 kDa	1	0	0	0	0	0	0	0	0
Phosphatidylinositol 4,5-bisphosphate 3-kinase catalytic subunit beta isoform	PK3CB_HUMAN	123 kDa	1	0	0	0	0	0	0	0	0
Ras/Rap GTPase-activating protein SynGAP	SYGP1_HUMAN	148 kDa	1	0	0	0	0	0	0	0	0
Histone H4-like protein type G	H4G_HUMAN	11 kDa	1	0	0	0	0	0	0	0	0
Secretogranin-2	SCG2_HUMAN	71 kDa	1	0	0	0	0	0	0	0	0
SURP and G-patch domain-containing protein 2	SUGP2_HUMAN	120 kDa	1	0	0	0	0	0	0	0	0
Transient receptor potential cation channel subfamily M member 5	TRPM5_HUMAN	131 kDa	1	0	0	0	0	0	0	0	0
Arf-GAP with GTPase, ANK repeat and PH domain-containing protein 2	AGAP2_HUMAN	125 kDa	1	0	0	0	0	0	0	0	0
Neurabin-2	NEB2_HUMAN	89 kDa	1	0	0	0	0	0	0	0	0
Nuclear receptor coactivator 6	NCOA6_HUMAN	219 kDa	1	0	0	0	0	0	0	0	0
Neogenin	NEO1_HUMAN	160 kDa	1	0	0	0	0	0	0	0	0
Abl interactor 1	ABI1_HUMAN	55 kDa	1	0	0	0	0	0	0	0	0
Protein arginine N-methyltransferase 5	ANM5_HUMAN	73 kDa	1	0	0	0	0	0	0	0	0
Nuclear receptor coactivator 2	NCOA2_HUMAN	159 kDa	1	0	0	0	0	0	0	0	0
F-box only protein 10	FBX10_HUMAN	105 kDa	1	0	0	0	0	0	0	0	0
Retinoblastoma-like protein 1	RBL1_HUMAN	121 kDa	1	0	0	0	0	0	0	0	0
Required for meiotic nuclear division protein 1 homolog	RMND1_HUMAN	52 kDa	1	0	0	0	0	0	0	0	0
Coiled-coil domain-containing protein 37	CCD37_HUMAN	71 kDa	1	0	0	0	0	0	0	0	0
Calcium/calmodulin-dependent protein kinase type 1D	KCC1D_HUMAN	43 kDa	1	0	0	0	0	0	0	0	0
Amyloid-like protein 1	APLP1_HUMAN	72 kDa	1	0	0	0	0	0	0	0	0
Arf-GAP with GTPase, ANK repeat and PH domain-containing protein 3	AGAP3_HUMAN	95 kDa	1	0	0	0	0	0	0	0	0
Integrin beta-8	ITB8_HUMAN	86 kDa	1	0	0	0	0	0	0	0	0
Protein KIBRA	KIBRA_HUMAN	125 kDa	1	0	0	0	0	0	0	0	0
Ubiquitin carboxyl-terminal hydrolase CYLD	CYLD_HUMAN	107 kDa	1	0	0	0	0	0	0	0	0

Description	Accession	MW	Raw spectral counts								
			Frontal Cortex	Cerebellum	Right Ventricle	Mesentric lymph node	Liver	Pancreas	Proximal bile duct	Penis	Prostate
Casein kinase II subunit alpha'	CSK22_HUMAN	41 kDa	1	0	0	0	0	0	0	0	0
Exocyst complex component 3	EXOC3_HUMAN	87 kDa	1	0	0	0	0	0	0	0	0
Inactive dipeptidyl peptidase 10	DPP10_HUMAN	91 kDa	1	0	0	0	0	0	0	0	0
Serine/threonine-protein kinase BRSK1	BRSK1_HUMAN	85 kDa	1	0	0	0	0	0	0	0	0
DnaJ homolog subfamily C member 7	DNJC7_HUMAN	56 kDa	1	0	0	0	0	0	0	0	0
Gamma-tubulin complex component 3	GCP3_HUMAN	104 kDa	1	0	0	0	0	0	0	0	0
Liprin-alpha-2	LIPA2_HUMAN	143 kDa	1	0	0	0	0	0	0	0	0
Disintegrin and metalloproteinase domain-containing protein 23	ADA23_HUMAN	92 kDa	1	0	0	0	0	0	0	0	0
Urotensin-2 receptor	UR2R_HUMAN	42 kDa	1	0	0	0	0	0	0	0	0
Protocadherin-1	PCDH1_HUMAN	115 kDa	1	0	0	0	0	0	0	0	0
cGMP-dependent 3',5'-cyclic phosphodiesterase	PDE2A_HUMAN	106 kDa	1	0	0	0	0	0	0	0	0
Actin-binding LIM protein 2	ABLM2_HUMAN	68 kDa	1	0	0	0	0	0	0	0	0
Syntaxin-4	STX4_HUMAN	34 kDa	1	0	0	0	0	0	0	0	0
Pre-mRNA-processing factor 19	PRP19_HUMAN	55 kDa	1	0	0	0	0	0	0	0	0
Armadillo repeat-containing X-linked protein 3	ARMX3_HUMAN	43 kDa	1	0	0	0	0	0	0	0	0
Proteasome inhibitor PI31 subunit	PSMF1_HUMAN	30 kDa	1	0	0	0	0	0	0	0	0
Transcription initiation factor TFIID subunit 3	TAF3_HUMAN	104 kDa	1	0	0	0	0	0	0	0	0
Protocadherin-9	PCDH9_HUMAN	136 kDa	1	0	0	0	0	0	0	0	0
Metabotropic glutamate receptor 7	GRM7_HUMAN	102 kDa	1	0	0	0	0	0	0	0	0
Leucine-rich glioma-inactivated protein 1	LGI1_HUMAN	64 kDa	1	0	0	0	0	0	0	0	0
Uncharacterized protein C2orf47	CB047_HUMAN	33 kDa	1	0	0	0	0	0	0	0	0
Solute carrier family 12 member 2	S12A2_HUMAN	131 kDa	1	0	0	0	0	0	0	0	0
Armadillo repeat-containing protein 8	ARMC8_HUMAN	76 kDa	1	0	0	0	0	0	0	0	0
Cytochrome c oxidase subunit 7B	COX7B_HUMAN	9 kDa	1	0	0	0	0	0	0	0	0
Nuclear distribution protein nudE-like 1	NDEL1_HUMAN	38 kDa	1	0	0	0	0	0	0	0	0
Sodium/nucleoside cotransporter 2	S28A2_HUMAN	72 kDa	1	0	0	0	0	0	0	0	0
Vacuolar protein sorting-associated protein 53 homolog	VPS53_HUMAN	80 kDa	1	0	0	0	0	0	0	0	0
Ciliary neurotrophic factor receptor subunit alpha	CNTFR_HUMAN	41 kDa	1	0	0	0	0	0	0	0	0
Tetratricopeptide repeat protein 19	TTC19_HUMAN	42 kDa	1	0	0	0	0	0	0	0	0
Homer protein homolog 1	HOME1_HUMAN	40 kDa	1	0	0	0	0	0	0	0	0
Neural Wiskott-Aldrich syndrome protein	WASL_HUMAN	55 kDa	1	0	0	0	0	0	0	0	0
Noelin	NOE1_HUMAN	55 kDa	1	0	0	0	0	0	0	0	0
Ubiquitin-like-conjugating enzyme ATG3	ATG3_HUMAN	36 kDa	1	0	0	0	0	0	0	0	0
Isoaspartyl peptidase/L-asparaginase	ASGL1_HUMAN	32 kDa	1	0	0	0	0	0	0	0	0
Fibroblast growth factor 12	FGF12_HUMAN	27 kDa	1	0	0	0	0	0	0	0	0
Protein syndesmos	SDOS_HUMAN	23 kDa	1	0	0	0	0	0	0	0	0
E3 ubiquitin-protein ligase HACE1	HACE1_HUMAN	102 kDa	1	0	0	0	0	0	0	0	0
Spermatogenesis- and oogenesis-specific basic helix-loop-helix-containing protein 2	SOLH2_HUMAN	47 kDa	1	0	0	0	0	0	0	0	0
Armadillo repeat-containing protein 10	ARM10_HUMAN	38 kDa	1	0	0	0	0	0	0	0	0
Cob(I)yrinic acid a,c-diamide adenosyltransferase	MMAB_HUMAN	27 kDa	1	0	0	0	0	0	0	0	0
Tubulin-specific chaperone A	TBCA_HUMAN	13 kDa	1	0	0	0	0	0	0	0	0
Protein C10	C10_HUMAN	13 kDa	1	0	0	0	0	0	0	0	0
Neuronal pentraxin receptor	NPTXR_HUMAN	53 kDa	1	0	0	0	0	0	0	0	0
Small VCP/p97-interacting protein	SVIP_HUMAN	8 kDa	1	0	0	0	0	0	0	0	0
Calcium signal-modulating cyclophilin ligand	CAMLG_HUMAN	33 kDa	1	0	0	0	0	0	0	0	0
Gap junction alpha-1 protein	CXA1_HUMAN	43 kDa	1	0	0	0	0	0	0	0	0
Synaptosomal-associated protein 29	SNP29_HUMAN	29 kDa	1	0	0	0	0	0	0	0	0
Ras-related protein Rab-31	RAB31_HUMAN	22 kDa	1	0	0	0	0	0	0	0	0
Calcium-activated potassium channel subunit beta-3	KCMB3_HUMAN	32 kDa	1	0	0	0	0	0	0	0	0
Receptor expression-enhancing protein 2	REEP2_HUMAN	28 kDa	1	0	0	0	0	0	0	0	0

Description	Accession	MW	Raw spectral counts								
			Frontal Cortex	Cerebellum	Right Ventricle	Mesentric lymph node	Liver	Pancreas	Proximal bile duct	Penis	Prostate
Protein S100-A13	S10AD_HUMAN	11 kDa	1	0	0	0	0	0	0	0	0
Sideroflexin-5	SFXN5_HUMAN	37 kDa	1	0	0	0	0	0	0	0	0
Cytochrome b-c1 complex subunit 8	QCR8_HUMAN	10 kDa	1	0	0	0	0	0	0	0	0
Mitochondrial fission factor	MFF_HUMAN	38 kDa	1	0	0	0	0	0	0	0	0
BolA-like protein 2	BOLA2_HUMAN	10 kDa	1	0	0	0	0	0	0	0	0
Protein shisa-4	SHSA4_HUMAN	22 kDa	1	0	0	0	0	0	0	0	0
Ras-related protein M-Ras	RASM_HUMAN	24 kDa	1	0	0	0	0	0	0	0	0
Protein NDRG3	NDRG3_HUMAN	41 kDa	1	0	0	0	0	0	0	0	0
Neuritin	NRN1_HUMAN	15 kDa	1	0	0	0	0	0	0	0	0
GPI transamidase component PIG-S	PIGS_HUMAN	62 kDa	1	0	0	0	0	0	0	0	0
Adipogenesis regulatory factor	ADIRF_HUMAN	8 kDa	1	0	0	0	0	0	0	0	0
Clathrin interactor 1	EPN4_HUMAN	68 kDa	1	0	0	0	0	0	0	0	0
Charged multivesicular body protein 5	CHMP5_HUMAN	25 kDa	1	0	0	0	0	0	0	0	0
Carboxypeptidase E	CBPE_HUMAN	53 kDa	1	0	0	0	0	0	0	0	0
L-aminoadipate-semialdehyde dehydrogenase-phosphopantetheinyl transferase	ADPPT_HUMAN	36 kDa	1	0	0	0	0	0	0	0	0
Serine/threonine-protein phosphatase 2A 56 kDa regulatory subunit gamma isoform	2A5G_HUMAN	61 kDa	1	0	0	0	0	0	0	0	0
Putative peptidyl-tRNA hydrolase PTRHD1	PTRD1_HUMAN	16 kDa	1	0	0	0	0	0	0	0	0
Leucine zipper transcription factor-like protein 1	LZTL1_HUMAN	35 kDa	1	0	0	0	0	0	0	0	0
Interferon-induced, double-stranded RNA-activated protein kinase	E2AK2_HUMAN	62 kDa	1	0	0	0	0	0	0	0	0
Trans-2-enoyl-CoA reductase	MECR_HUMAN	40 kDa	1	0	0	0	0	0	0	0	0
Uncharacterized protein KIAA1210	K1210_HUMAN	187 kDa	1	0	0	0	0	0	0	0	0
Pyridoxal kinase	PDXK_HUMAN	35 kDa	1	0	0	0	0	0	0	0	0
Kinesin-like protein KIF1B	KIF1B_HUMAN	204 kDa	1	0	0	0	0	0	0	0	0
Cytoplasmic dynein 1 intermediate chain 1	DC1I1_HUMAN	73 kDa	1	0	0	0	0	0	0	0	0
EPM2A-interacting protein 1	EPMIP_HUMAN	70 kDa	1	0	0	0	0	0	0	0	0
Calcium/calmodulin-dependent protein kinase kinase 2	KKCC2_HUMAN	65 kDa	1	0	0	0	0	0	0	0	0
Vesicle-associated membrane protein 1	VAMP1_HUMAN	13 kDa	1	0	0	0	0	0	0	0	0
Hyaluronan and proteoglycan link protein 2	HPLN2_HUMAN	38 kDa	1	0	0	0	0	0	0	0	0
Barrier-to-autointegration factor	BAF_HUMAN	10 kDa	1	0	0	0	0	0	0	0	0
Retinol-binding protein 1	RET1_HUMAN	16 kDa	0	0	0	0	1	0	0	0	0
Phosphoribosyl pyrophosphate synthase-associated protein 1	KPRA_HUMAN	39 kDa	0	0	0	0	1	0	0	0	0
Leukotriene-B(4) omega-hydroxylase 1	CP4F2_HUMAN	60 kDa	0	0	0	0	1	0	0	0	0
Oxygen-dependent coproporphyrinogen-III oxidase	HEM6_HUMAN	50 kDa	0	0	0	0	1	0	0	0	0
Formimidoyltransferase-cyclodeaminase	FTCD_HUMAN	59 kDa	0	0	0	0	1	0	0	0	0
Cytochrome P450 2E1	CP2E1_HUMAN	57 kDa	0	0	0	0	1	0	0	0	0
Alpha-tocopherol transfer protein	TTPA_HUMAN	32 kDa	0	0	0	0	1	0	0	0	0
Delta(14)-sterol reductase	ERG24_HUMAN	46 kDa	0	0	0	0	1	0	0	0	0
Dihydropyrimidinase	DPYS_HUMAN	57 kDa	0	0	0	0	1	0	0	0	0
7-alpha-hydroxycholest-4-en-3-one 12-alpha-hydroxylase	CP8B1_HUMAN	58 kDa	0	0	0	0	1	0	0	0	0
Probable imidazolonepropionase	HUTI_HUMAN	47 kDa	0	0	0	0	1	0	0	0	0
Diacylglycerol kinase epsilon	DGKE_HUMAN	64 kDa	0	0	0	0	1	0	0	0	0
Rho GTPase-activating protein 44	RHG44_HUMAN	89 kDa	0	0	0	0	1	0	0	0	0
Histone-lysine N-methyltransferase 2A	KMT2A_HUMAN	432 kDa	0	0	0	0	1	0	0	0	0
Hemicentin-2	HMCN2_HUMAN	543 kDa	0	0	0	0	1	0	0	0	0
F-box only protein 3	FBX3_HUMAN	55 kDa	0	0	0	0	1	0	0	0	0
Regulator of microtubule dynamics protein 2	RMD2_HUMAN	47 kDa	0	0	0	0	1	0	0	0	0
Ephrin type-A receptor 5	EPHA5_HUMAN	115 kDa	0	0	0	0	1	0	0	0	0
Dynein heavy chain 12, axonemal	DYH12_HUMAN	357 kDa	0	0	0	0	1	0	0	0	0
Protein sidekick-2	SDK2_HUMAN	239 kDa	0	0	0	0	1	0	0	0	0
Alpha-mannosidase 2	MA2A1_HUMAN	131 kDa	0	0	0	0	1	0	0	0	0

Description	Accession	MW	Raw spectral counts								
			Frontal Cortex	Cerebellum	Right Ventricle	Mesentric lymph node	Liver	Pancreas	Proximal bile duct	Penis	Prostate
Hepatocyte growth factor receptor	MET_HUMAN	156 kDa	0	0	0	0	1	0	0	0	0
Spermatogenesis-associated protein 13	SPT13_HUMAN	75 kDa	0	0	0	0	1	0	0	0	0
Dihydropyrimidine dehydrogenase [NADP(+)]	DPYD_HUMAN	111 kDa	0	0	0	0	1	0	0	0	0
EF-hand calcium-binding domain-containing protein 3	EFCB3_HUMAN	50 kDa	0	0	0	0	1	0	0	0	0
A disintegrin and metalloproteinase with thrombospondin motifs 13	ATS13_HUMAN	154 kDa	0	0	0	0	1	0	0	0	0
Glucokinase regulatory protein	GCKR_HUMAN	69 kDa	0	0	0	0	1	0	0	0	0
Carnitine O-palmitoyltransferase 1, liver isoform	CPT1A_HUMAN	88 kDa	0	0	0	0	1	0	0	0	0
Paraplegin	SPG7_HUMAN	88 kDa	0	0	0	0	1	0	0	0	0
Glucose-6-phosphate translocase	G6PT1_HUMAN	46 kDa	0	0	0	0	1	0	0	0	0
Solute carrier family 12 member 8	S12A8_HUMAN	78 kDa	0	0	0	0	1	0	0	0	0
GLIPR1-like protein 1	GPRL1_HUMAN	27 kDa	0	0	0	0	1	0	0	0	0
Beta,beta-carotene 9',10'-oxygenase	BCDO2_HUMAN	66 kDa	0	0	0	0	1	0	0	0	0
Phosphatidylglycerophosphatase and protein-tyrosine phosphatase 1	PTPM1_HUMAN	23 kDa	0	0	0	0	1	0	0	0	0
Putative 2-oxo-4-hydroxy-4-carboxy-5-ureidoimidazoline decarboxylase	URAD_HUMAN	19 kDa	0	0	0	0	1	0	0	0	0
Agmatinase	SPEB_HUMAN	38 kDa	0	0	0	0	1	0	0	0	0
Ubiquinone biosynthesis protein COQ4 homolog	COQ4_HUMAN	30 kDa	0	0	0	0	1	0	0	0	0
Uncharacterized protein C17orf62	CQ062_HUMAN	21 kDa	0	0	0	0	1	0	0	0	0
3-keto-steroid reductase	DHB7_HUMAN	38 kDa	0	0	0	0	1	0	0	0	0
Bax inhibitor 1	BI1_HUMAN	27 kDa	0	0	0	0	1	0	0	0	0
Olfactory receptor 5M10	OR5MA_HUMAN	36 kDa	0	0	0	0	1	0	0	0	0
Adenylosuccinate synthetase isozyme 1	PURA1_HUMAN	50 kDa	0	0	0	0	1	0	0	0	0
Ankyrin repeat and FYVE domain-containing protein 1	ANFY1_HUMAN	128 kDa	0	0	0	0	1	0	0	0	0
Phospholemman	PLM_HUMAN	10 kDa	0	0	0	0	1	0	0	0	0
WD repeat-containing protein 81	WDR81_HUMAN	212 kDa	0	0	0	1	0	0	0	0	0
Carboxypeptidase D	CBPD_HUMAN	153 kDa	0	0	0	1	0	0	0	0	0
Ubiquitin carboxyl-terminal hydrolase 24	UBP24_HUMAN	294 kDa	0	0	0	1	0	0	0	0	0
Centromere protein F	CENPF_HUMAN	368 kDa	0	0	0	1	0	0	0	0	0
Lysosomal-trafficking regulator	LYST_HUMAN	429 kDa	0	0	0	1	0	0	0	0	0
Alpha-tectorin	TECTA_HUMAN	240 kDa	0	0	0	1	0	0	0	0	0
Xylosyltransferase 1	XYLT1_HUMAN	108 kDa	0	0	0	1	0	0	0	0	0
TATA element modulatory factor	TMF1_HUMAN	123 kDa	0	0	0	1	0	0	0	0	0
KAT8 regulatory NSL complex subunit 1	KANL1_HUMAN	121 kDa	0	0	0	1	0	0	0	0	0
A-kinase anchor protein 13	AKP13_HUMAN	308 kDa	0	0	0	1	0	0	0	0	0
Probable inactive glycosyltransferase 25 family member 3	GT253_HUMAN	68 kDa	0	0	0	1	0	0	0	0	0
Peroxidasin homolog	PXDN_HUMAN	165 kDa	0	0	0	1	0	0	0	0	0
Ubiquitin carboxyl-terminal hydrolase 15	UBP15_HUMAN	112 kDa	0	0	0	1	0	0	0	0	0
Ribonucleoprotein PTB-binding 2	RAVR2_HUMAN	74 kDa	0	0	0	1	0	0	0	0	0
1-acyl-sn-glycerol-3-phosphate acyltransferase beta	PLCB_HUMAN	31 kDa	0	0	0	1	0	0	0	0	0
Proteasome subunit beta type-8	PSB8_HUMAN	30 kDa	0	0	0	1	0	0	0	0	0
VWFA and cache domain-containing protein 1	CAHD1_HUMAN	142 kDa	0	0	0	1	0	0	0	0	0
Lateral signaling target protein 2 homolog	LST2_HUMAN	96 kDa	0	0	0	0	0	0	1	0	0
ARF GTPase-activating protein GIT1	GIT1_HUMAN	84 kDa	0	0	0	0	0	0	1	0	0
ATP-binding cassette sub-family C member 9	ABCC9_HUMAN	174 kDa	0	0	0	0	0	0	1	0	0
Cortactin-binding protein 2	CTTB2_HUMAN	181 kDa	0	0	0	0	0	0	1	0	0
Adenomatous polyposis coli protein	APC_HUMAN	312 kDa	0	0	0	0	0	0	1	0	0
Striatin-4	STRN4_HUMAN	81 kDa	0	0	0	0	0	0	1	0	0
Inter-alpha-trypsin inhibitor heavy chain H4	ITIH4_HUMAN	103 kDa	0	0	0	0	0	0	1	0	0
Ubiquitin carboxyl-terminal hydrolase 40	UBP40_HUMAN	140 kDa	0	0	0	0	0	0	1	0	0
RIMS-binding protein 3A	RIM3A_HUMAN	181 kDa	0	0	0	0	0	0	1	0	0
E3 ubiquitin-protein ligase CBL-B	CBLB_HUMAN	109 kDa	0	0	0	0	0	0	1	0	0

Description	Accession	MW	Raw spectral counts								
			Frontal Cortex	Cerebellum	Right Ventricle	Mesentric lymph node	Liver	Pancreas	Proximal bile duct	Penis	Prostate
Protein LSM14 homolog A	LS14A_HUMAN	51 kDa	0	0	0	0	0	0	1	0	0
Endoplasmic reticulum aminopeptidase 1	ERAP1_HUMAN	107 kDa	0	0	0	0	0	0	1	0	0
Deleted in malignant brain tumors 1 protein	DMBT1_HUMAN	261 kDa	0	0	0	0	0	0	1	0	0
ATP-binding cassette sub-family C member 11	ABCCB_HUMAN	154 kDa	0	0	0	0	0	0	1	0	0
Metabotropic glutamate receptor 1	GRM1_HUMAN	132 kDa	0	0	0	0	0	0	1	0	0
PML-RARA-regulated adapter molecule 1	PRAM_HUMAN	79 kDa	0	0	0	0	0	0	1	0	0
Protein PML	PML_HUMAN	98 kDa	0	0	0	0	0	0	1	0	0
Complement C2	CO2_HUMAN	83 kDa	0	0	0	0	0	0	1	0	0
Tyrosine-protein kinase SgK223	SG223_HUMAN	149 kDa	0	0	0	0	0	0	1	0	0
Afamin	AFAM_HUMAN	69 kDa	0	0	0	0	0	0	1	0	0
Apolipoprotein D	APOD_HUMAN	21 kDa	0	0	0	0	0	0	1	0	0
ATP-binding cassette sub-family A member 8	ABCA8_HUMAN	179 kDa	0	0	0	0	0	0	1	0	0
Mast cell carboxypeptidase A	CBPA3_HUMAN	49 kDa	0	0	0	0	0	0	1	0	0
RNA-binding protein 39	RBM39_HUMAN	59 kDa	0	0	0	0	0	1	0	0	0
Putative transferase CAF17	CAF17_HUMAN	38 kDa	0	0	0	0	0	1	0	0	0
Dedicator of cytokinesis protein 1	DOCK1_HUMAN	215 kDa	0	0	0	0	0	1	0	0	0
Synaptogyrin-2	SNG2_HUMAN	25 kDa	0	0	0	0	0	1	0	0	0
60S ribosomal protein L37a	RL37A_HUMAN	10 kDa	0	0	0	0	0	1	0	0	0
Mannose-P-dolichol utilization defect 1 protein	MPU1_HUMAN	27 kDa	0	0	0	0	0	1	0	0	0
Dynein heavy chain 9, axonemal	DYH9_HUMAN	512 kDa	0	0	0	0	0	1	0	0	0
E3 ubiquitin-protein ligase RNF149	RN149_HUMAN	43 kDa	0	0	0	0	0	1	0	0	0
Dynein heavy chain 2, axonemal	DYH2_HUMAN	508 kDa	0	0	0	0	0	1	0	0	0
Vacuolar protein sorting-associated protein 13A	VP13A_HUMAN	360 kDa	0	0	0	0	0	1	0	0	0
Protein FAM208B	F208B_HUMAN	269 kDa	0	0	0	0	0	1	0	0	0
DNA transposase THAP9	THAP9_HUMAN	103 kDa	0	0	0	0	0	1	0	0	0
Histone-lysine N-methyltransferase 2B	KMT2B_HUMAN	294 kDa	0	0	0	0	0	1	0	0	0
Protein SERAC1	SRAC1_HUMAN	74 kDa	0	0	0	0	0	1	0	0	0
WD repeat-containing protein 65	WDR65_HUMAN	145 kDa	0	0	0	0	0	1	0	0	0
Neurobeachin	NBEA_HUMAN	328 kDa	0	0	0	0	0	1	0	0	0
Protein SZT2	SZT2_HUMAN	378 kDa	0	0	0	0	0	1	0	0	0
Putative ciliary rootlet coiled-coil protein-like 3 protein	CROL3_HUMAN	248 kDa	0	0	0	0	0	1	0	0	0
Pre-mRNA-processing factor 6	PRP6_HUMAN	107 kDa	0	0	0	0	0	1	0	0	0
Centrosome and spindle pole-associated protein 1	CSPP1_HUMAN	146 kDa	0	0	0	0	0	1	0	0	0
Polypyrimidine tract-binding protein 2	PTBP2_HUMAN	57 kDa	0	0	0	0	0	1	0	0	0
Rab3 GTPase-activating protein non-catalytic subunit	RBGPR_HUMAN	156 kDa	0	0	0	0	0	1	0	0	0
Protein kinase C and casein kinase substrate in neurons protein 2	PACN2_HUMAN	56 kDa	0	0	0	0	0	1	0	0	0
ATP-dependent RNA helicase DDX42	DDX42_HUMAN	103 kDa	0	0	0	0	0	1	0	0	0
Asparagine--tRNA ligase, cytoplasmic	SYNC_HUMAN	63 kDa	0	0	0	0	0	1	0	0	0
EKC/KEOPS complex subunit TPRKB	TPRKB_HUMAN	20 kDa	0	0	0	0	0	1	0	0	0
Trophinin	TROP_HUMAN	144 kDa	0	0	0	0	0	1	0	0	0
Cell division cycle and apoptosis regulator protein 1	CCAR1_HUMAN	133 kDa	0	0	0	0	0	1	0	0	0
Probable aminopeptidase NPEPL1	PEPL1_HUMAN	56 kDa	0	0	0	0	0	1	0	0	0
Inactive L-threonine 3-dehydrogenase	TDH_HUMAN	25 kDa	0	0	0	0	0	1	0	0	0
Translocation protein SEC62	SEC62_HUMAN	46 kDa	0	0	0	0	0	1	0	0	0
N-alpha-acetyltransferase 30	NAA30_HUMAN	39 kDa	0	0	0	0	0	1	0	0	0
Zymogen granule protein 16 homolog B	ZG16B_HUMAN	23 kDa	0	0	0	0	0	1	0	0	0
Coronin-7	CORO7_HUMAN	101 kDa	0	0	0	0	0	1	0	0	0
Protein ITFG3	ITFG3_HUMAN	60 kDa	0	0	0	0	0	1	0	0	0
Protein FAM98A	FA98A_HUMAN	55 kDa	0	0	0	0	0	1	0	0	0
COMM domain-containing protein 9	COMD9_HUMAN	22 kDa	0	0	0	0	0	1	0	0	0

Description	Accession	MW	Raw spectral counts								
			Frontal Cortex	Cerebellum	Right Ventricle	Mesentric lymph node	Liver	Pancreas	Proximal bile duct	Penis	Prostate
E3 ubiquitin-protein ligase synoviolin	SYVN1_HUMAN	68 kDa	0	0	0	0	0	1	0	0	0
Phosphatidylinositol 5-phosphate 4-kinase type-2 gamma	PI42C_HUMAN	47 kDa	0	0	0	0	0	1	0	0	0
C-Myc-binding protein	MYCBP_HUMAN	12 kDa	0	0	0	0	0	1	0	0	0
Serpin-like protein HMSD	HMSD_HUMAN	15 kDa	0	0	0	0	0	1	0	0	0
Eukaryotic translation initiation factor 3 subunit M	EIF3M_HUMAN	43 kDa	0	0	0	0	0	1	0	0	0
E3 ubiquitin-protein ligase TRIM23	TRI23_HUMAN	64 kDa	0	0	0	0	0	1	0	0	0
CDGSH iron-sulfur domain-containing protein 2	CISD2_HUMAN	15 kDa	0	0	0	0	0	1	0	0	0
60S ribosomal protein L34	RL34_HUMAN	13 kDa	0	0	0	0	0	1	0	0	0
Contactin-5	CNTN5_HUMAN	121 kDa	0	0	0	0	0	1	0	0	0
Monocarboxylate transporter 10	MOT10_HUMAN	55 kDa	0	0	0	0	0	1	0	0	0
Protein pelota homolog	PELO_HUMAN	43 kDa	0	0	0	0	0	1	0	0	0
Aldehyde dehydrogenase family 16 member A1	A16A1_HUMAN	85 kDa	0	0	0	0	0	1	0	0	0
PDZ domain-containing protein GIPC2	GIPC2_HUMAN	34 kDa	0	0	0	0	0	1	0	0	0
Diphthine synthase	DPH5_HUMAN	32 kDa	0	0	0	0	0	1	0	0	0
15 kDa selenoprotein	SEP15_HUMAN	18 kDa	0	0	0	0	0	1	0	0	0
ATP-binding cassette sub-family E member 1	ABCE1_HUMAN	67 kDa	0	0	0	0	0	1	0	0	0
Transmembrane protein 97	TMM97_HUMAN	21 kDa	0	0	0	0	0	1	0	0	0
Dolichyl-phosphate beta-glucosyltransferase	ALG5_HUMAN	37 kDa	0	0	0	0	0	1	0	0	0
LDLR chaperone MESD	MESD_HUMAN	26 kDa	0	0	0	0	0	1	0	0	0
Eukaryotic translation initiation factor 3 subunit K	EIF3K_HUMAN	25 kDa	0	0	0	0	0	1	0	0	0
60S ribosomal protein L36a	RL36A_HUMAN	12 kDa	0	0	0	0	0	1	0	0	0
Kallikrein-1	KLK1_HUMAN	29 kDa	0	0	0	0	0	1	0	0	0
Stromal cell-derived factor 2-like protein 1	SDF2L_HUMAN	24 kDa	0	0	0	0	0	1	0	0	0
Branched-chain-amino-acid aminotransferase, cytosolic	BCAT1_HUMAN	43 kDa	0	0	0	0	0	1	0	0	0
Signal recognition particle 19 kDa protein	SRP19_HUMAN	16 kDa	0	0	0	0	0	1	0	0	0
Ubiquitin-fold modifier 1	UFM1_HUMAN	9 kDa	0	0	0	0	0	1	0	0	0
Transmembrane protein 214	TM214_HUMAN	77 kDa	0	0	0	0	0	1	0	0	0
Structural maintenance of chromosomes protein 4	SMC4_HUMAN	147 kDa	0	0	0	0	0	1	0	0	0
Ubiquitin-fold modifier-conjugating enzyme 1	UFC1_HUMAN	19 kDa	0	0	0	0	0	1	0	0	0
Dynein heavy chain 3, axonemal	DYH3_HUMAN	471 kDa	0	0	0	0	0	1	0	0	0
Cleavage and polyadenylation specificity factor subunit 6	CPSF6_HUMAN	59 kDa	0	0	0	0	0	1	0	0	0
Collagen alpha-1(I) chain	CO1A1_HUMAN	139 kDa	0	0	0	0	0	0	0	1	0
NADPH:adrenodoxin oxidoreductase	ADRO_HUMAN	54 kDa	0	0	0	0	0	0	0	1	0
Mitotic checkpoint protein BUB3	BUB3_HUMAN	37 kDa	0	0	0	0	0	0	0	1	0
Small nuclear ribonucleoprotein F	RUXF_HUMAN	10 kDa	0	0	0	0	0	0	0	1	0
Double-stranded RNA-specific adenosine deaminase	DSRAD_HUMAN	136 kDa	0	0	0	0	0	0	0	1	0
UPF0553 protein C9orf64	CI064_HUMAN	39 kDa	0	0	0	0	0	0	0	1	0
Vitronectin	VTNC_HUMAN	54 kDa	0	0	0	0	0	0	0	1	0
Probable tRNA N6-adenosine threonylcarbamoyltransferase	OSGEP_HUMAN	36 kDa	0	0	0	0	0	0	0	1	0
Sorting nexin-6	SNX6_HUMAN	47 kDa	0	0	0	0	0	0	0	1	0
Tyrosine-protein kinase CSK	CSK_HUMAN	51 kDa	0	0	0	0	0	0	0	1	0
Hepatoma-derived growth factor	HDGF_HUMAN	27 kDa	0	0	0	0	0	0	0	1	0
26S proteasome non-ATPase regulatory subunit 10	PSD10_HUMAN	24 kDa	0	0	0	0	0	0	0	1	0
Tropomodulin-3	TMOD3_HUMAN	40 kDa	0	0	0	0	0	0	0	1	0
Thimet oligopeptidase	THOP1_HUMAN	79 kDa	0	0	0	0	0	0	0	1	0
Protein LZIC	LZIC_HUMAN	21 kDa	0	0	0	0	0	0	0	1	0
Translation initiation factor eIF-2B subunit alpha	EI2BA_HUMAN	34 kDa	0	0	0	0	0	0	0	1	0
m7GpppX diphosphatase	DCPS_HUMAN	39 kDa	0	0	0	0	0	0	0	1	0
Isopentenyl-diphosphate Delta-isomerase 1	IDI1_HUMAN	26 kDa	0	0	0	0	0	0	0	1	0
Enoyl-CoA delta isomerase 2	ECI2_HUMAN	44 kDa	0	0	0	0	0	0	0	1	0

Description	Accession	MW	Raw spectral counts								
			Frontal Cortex	Cerebellum	Right Ventricle	Mesentric lymph node	Liver	Pancreas	Proximal bile duct	Penis	Prostate
Cingulin	CING_HUMAN	136 kDa	0	0	0	0	0	0	0	1	0
Protein NOXP20	NXP20_HUMAN	61 kDa	0	0	0	0	0	0	0	1	0
Transcription elongation regulator 1	TCRG1_HUMAN	124 kDa	0	0	0	0	0	0	0	1	0
Phosphoribosylformylglycinamidine synthase	PUR4_HUMAN	145 kDa	0	0	0	0	0	0	0	1	0
Signal transducer and activator of transcription 3	STAT3_HUMAN	88 kDa	0	0	0	0	0	0	0	1	0
Sacsin	SACS_HUMAN	521 kDa	0	0	0	0	0	0	0	1	0
Spectrin beta chain, non-erythrocytic 5	SPTN5_HUMAN	417 kDa	0	0	0	0	0	0	0	1	0
Probable E3 ubiquitin-protein ligase HERC1	HERC1_HUMAN	532 kDa	0	0	0	0	0	0	0	1	0
Neurolysin	NEUL_HUMAN	81 kDa	0	0	0	0	0	0	0	1	0
Alsin	ALS2_HUMAN	184 kDa	0	0	0	0	0	0	0	1	0
Leucine-rich repeat serine/threonine-protein kinase 1	LRRK1_HUMAN	225 kDa	0	0	0	0	0	0	0	1	0
Collagen alpha-2(IV) chain	CO4A2_HUMAN	168 kDa	0	0	0	0	0	0	0	1	0
Ras-related protein Rab-7b	RAB7B_HUMAN	23 kDa	0	0	0	0	0	0	0	1	0
Proteasome-associated protein ECM29 homolog	ECM29_HUMAN	204 kDa	0	0	0	0	0	0	0	1	0
Nuclear pore complex protein Nup205	NU205_HUMAN	228 kDa	0	0	0	0	0	0	0	1	0
Tyrosine-protein phosphatase non-receptor type 23	PTN23_HUMAN	179 kDa	0	0	0	0	0	0	0	1	0
CCR4-NOT transcription complex subunit 1	CNOT1_HUMAN	267 kDa	0	0	0	0	0	0	0	1	0
DNA topoisomerase 2-beta	TOP2B_HUMAN	183 kDa	0	0	0	0	0	0	0	1	0
Merlin	MERL_HUMAN	70 kDa	0	0	0	0	0	0	0	1	0
Transforming acidic coiled-coil-containing protein 2	TACC2_HUMAN	309 kDa	0	0	0	0	0	0	0	1	0
GREB1-like protein	GRB1L_HUMAN	214 kDa	0	0	0	0	0	0	0	1	0
Elongation factor Tu GTP-binding domain-containing protein 1	ETUD1_HUMAN	125 kDa	0	0	0	0	0	0	0	1	0
Protein flightless-1 homolog	FLII_HUMAN	145 kDa	0	0	0	0	0	0	0	1	0
Uncharacterized protein C6orf132	CF132_HUMAN	124 kDa	0	0	0	0	0	0	0	1	0
Huntingtin-interacting protein 1-related protein	HIP1R_HUMAN	119 kDa	0	0	0	0	0	0	0	1	0
Involucrin	INVO_HUMAN	68 kDa	0	0	0	0	0	0	0	1	0
Protein SCAF8	SCAF8_HUMAN	141 kDa	0	0	0	0	0	0	0	1	0
Probable ATP-dependent RNA helicase DDX46	DDX46_HUMAN	117 kDa	0	0	0	0	0	0	0	1	0
C2 domain-containing protein 3	C2CD3_HUMAN	260 kDa	0	0	0	0	0	0	0	1	0
Exocyst complex component 8	EXOC8_HUMAN	82 kDa	0	0	0	0	0	0	0	1	0
Alpha-2-macroglobulin-like protein 1	A2ML1_HUMAN	161 kDa	0	0	0	0	0	0	0	1	0
Serine/threonine-protein kinase MRCK gamma	MRCKG_HUMAN	172 kDa	0	0	0	0	0	0	0	1	0
Zinc finger and BTB domain-containing protein 38	ZBT38_HUMAN	134 kDa	0	0	0	0	0	0	0	1	0
RNA-binding protein EWS	EWS_HUMAN	68 kDa	0	0	0	0	0	0	0	1	0
Myb-binding protein 1A	MBB1A_HUMAN	149 kDa	0	0	0	0	0	0	0	1	0
Syntaxin-binding protein 2	STXB2_HUMAN	66 kDa	0	0	0	0	0	0	0	1	0
Metastasis-associated protein MTA2	MTA2_HUMAN	75 kDa	0	0	0	0	0	0	0	1	0
Mitochondrial intermediate peptidase	MIPEP_HUMAN	81 kDa	0	0	0	0	0	0	0	1	0
ATP-binding cassette sub-family F member 1	ABCF1_HUMAN	96 kDa	0	0	0	0	0	0	0	1	0
Nucleoporin Nup43	NUP43_HUMAN	42 kDa	0	0	0	0	0	0	0	1	0
mRNA export factor	RAE1L_HUMAN	41 kDa	0	0	0	0	0	0	0	1	0
Far upstream element-binding protein 3	FUBP3_HUMAN	62 kDa	0	0	0	0	0	0	0	1	0
Serine/threonine-protein kinase OSR1	OXSR1_HUMAN	58 kDa	0	0	0	0	0	0	0	1	0
Multifunctional protein ADE2	PUR6_HUMAN	47 kDa	0	0	0	0	0	0	0	1	0
tRNA (cytosine(34)-C(5))-methyltransferase	NSUN2_HUMAN	86 kDa	0	0	0	0	0	0	0	1	0
Exocyst complex component 2	EXOC2_HUMAN	104 kDa	0	0	0	0	0	0	0	1	0
Spermatogenesis-associated protein 31A6	S31A6_HUMAN	148 kDa	0	0	0	0	0	0	0	1	0
Lipolysis-stimulated lipoprotein receptor	LSR_HUMAN	71 kDa	0	0	0	0	0	0	0	1	0
Dual oxidase 1	DUOX1_HUMAN	177 kDa	0	0	0	0	0	0	0	1	0
Spastin	SPAST_HUMAN	67 kDa	0	0	0	0	0	0	0	1	0

Description	Accession	MW	Raw spectral counts								
			Frontal Cortex	Cerebellum	Right Ventricle	Mesentric lymph node	Liver	Pancreas	Proximal bile duct	Penis	Prostate
Caprin-1	CAPR1_HUMAN	78 kDa	0	0	0	0	0	0	0	1	0
IST1 homolog	IST1_HUMAN	40 kDa	0	0	0	0	0	0	0	1	0
Dipeptidyl peptidase 1	CATC_HUMAN	52 kDa	0	0	0	0	0	0	0	1	0
Desmocollin-1	DSC1_HUMAN	100 kDa	0	0	0	0	0	0	0	1	0
Uncharacterized protein C1orf131	CA131_HUMAN	33 kDa	0	0	0	0	0	0	0	1	0
Serine/threonine-protein kinase PAK 2	PAK2_HUMAN	58 kDa	0	0	0	0	0	0	0	1	0
Eukaryotic peptide chain release factor subunit 1	ERF1_HUMAN	49 kDa	0	0	0	0	0	0	0	1	0
Gamma-interferon-inducible protein 16	IF16_HUMAN	88 kDa	0	0	0	0	0	0	0	1	0
Kynurenine--oxoglutarate transaminase 3	KAT3_HUMAN	51 kDa	0	0	0	0	0	0	0	1	0
Zinc finger CCCH domain-containing protein 15	ZC3HF_HUMAN	49 kDa	0	0	0	0	0	0	0	1	0
Cytosolic 5'-nucleotidase 3A	5NT3A_HUMAN	38 kDa	0	0	0	0	0	0	0	1	0
Serine--tRNA ligase	SYSM_HUMAN	58 kDa	0	0	0	0	0	0	0	1	0
DNA replication licensing factor MCM4	MCM4_HUMAN	97 kDa	0	0	0	0	0	0	0	1	0
Serine/threonine-protein kinase 38	STK38_HUMAN	54 kDa	0	0	0	0	0	0	0	1	0
Vacuolar protein sorting-associated protein VTA1 homolog	VTA1_HUMAN	34 kDa	0	0	0	0	0	0	0	1	0
Kallikrein-7	KLK7_HUMAN	28 kDa	0	0	0	0	0	0	0	1	0
Beta-2-syntrophin	SNTB2_HUMAN	58 kDa	0	0	0	0	0	0	0	1	0
Pentatricopeptide repeat domain-containing protein 3	PTCD3_HUMAN	79 kDa	0	0	0	0	0	0	0	1	0
Epithelial splicing regulatory protein 2	ESRP2_HUMAN	78 kDa	0	0	0	0	0	0	0	1	0
LEM domain-containing protein 2	LEMD2_HUMAN	57 kDa	0	0	0	0	0	0	0	1	0
DNA-directed RNA polymerases I and III subunit RPAC1	RPAC1_HUMAN	39 kDa	0	0	0	0	0	0	0	1	0
SUN domain-containing protein 2	SUN2_HUMAN	80 kDa	0	0	0	0	0	0	0	1	0
PEST proteolytic signal-containing nuclear protein	PCNP_HUMAN	19 kDa	0	0	0	0	0	0	0	1	0
MICAL-like protein 1	MILK1_HUMAN	93 kDa	0	0	0	0	0	0	0	1	0
Ribonuclease 7	RNAS7_HUMAN	17 kDa	0	0	0	0	0	0	0	1	0
Short-chain dehydrogenase/reductase family 9C member 7	DR9C7_HUMAN	35 kDa	0	0	0	0	0	0	0	1	0
Trafficking protein particle complex subunit 4	TPPC4_HUMAN	24 kDa	0	0	0	0	0	0	0	1	0
28S ribosomal protein S28	RT28_HUMAN	21 kDa	0	0	0	0	0	0	0	1	0
Eukaryotic translation initiation factor 4E	IF4E_HUMAN	25 kDa	0	0	0	0	0	0	0	1	0
39S ribosomal protein L15	RM15_HUMAN	33 kDa	0	0	0	0	0	0	0	1	0
Bicaudal D-related protein 2	BICR2_HUMAN	57 kDa	0	0	0	0	0	0	0	1	0
CDK5 regulatory subunit-associated protein 3	CK5P3_HUMAN	57 kDa	0	0	0	0	0	0	0	1	0
Integrin alpha-2	ITA2_HUMAN	129 kDa	0	0	0	0	0	0	0	1	0
Transmembrane protein 11	TMM11_HUMAN	22 kDa	0	0	0	0	0	0	0	1	0
Interferon-induced guanylate-binding protein 2	GBP2_HUMAN	67 kDa	0	0	0	0	0	0	0	1	0
Perilipin-3	PLIN3_HUMAN	47 kDa	0	0	0	0	0	0	0	1	0
Estrogen sulfotransferase	ST1E1_HUMAN	35 kDa	0	0	0	0	0	0	0	1	0
DNA-directed RNA polymerases I, II, and III subunit RPABC1	RPAB1_HUMAN	25 kDa	0	0	0	0	0	0	0	1	0
Uridine 5'-monophosphate synthase	UMPS_HUMAN	52 kDa	0	0	0	0	0	0	0	1	0
Inositol polyphosphate 1-phosphatase	INPP_HUMAN	44 kDa	0	0	0	0	0	0	0	1	0
Actin-related protein 10	ARP10_HUMAN	46 kDa	0	0	0	0	0	0	0	1	0
Guanylate-binding protein 6	GBP6_HUMAN	72 kDa	0	0	0	0	0	0	0	1	0
Cathepsin L2	CATL2_HUMAN	37 kDa	0	0	0	0	0	0	0	1	0
Serpin B13	SPB13_HUMAN	44 kDa	0	0	0	0	0	0	0	1	0
Proteasomal ubiquitin receptor ADRM1	ADRM1_HUMAN	42 kDa	0	0	0	0	0	0	0	1	0
GMP reductase 2	GMPR2_HUMAN	38 kDa	0	0	0	0	0	0	0	1	0
MOB kinase activator 1B	MOB1B_HUMAN	25 kDa	0	0	0	0	0	0	0	1	0
Dehydrogenase/reductase SDR family member 11	DHR11_HUMAN	28 kDa	0	0	0	0	0	0	0	1	0
Chromatin complexes subunit BAP18	BAP18_HUMAN	18 kDa	0	0	0	0	0	0	0	1	0
Peptidyl-prolyl cis-trans isomerase C	PPIC_HUMAN	23 kDa	0	0	0	0	0	0	0	1	0

Description	Accession	MW	Raw spectral counts								
			Frontal Cortex	Cerebellum	Right Ventricle	Mesentric lymph node	Liver	Pancreas	Proximal bile duct	Penis	Prostate
GDP-fucose protein O-fucosyltransferase 1	OFUT1_HUMAN	44 kDa	0	0	0	0	0	0	0	1	0
Death-associated protein-like 1	DAPL1_HUMAN	12 kDa	0	0	0	0	0	0	0	1	0
Putative uncharacterized protein GSN-AS1	GSAS1_HUMAN	18 kDa	0	0	0	0	0	0	0	1	0
Bis(5'-nucleosyl)-tetraphosphatase [asymmetrical]	AP4A_HUMAN	17 kDa	0	0	0	0	0	0	0	1	0
Apoptosis-associated speck-like protein containing a CARD	ASC_HUMAN	22 kDa	0	0	0	0	0	0	0	1	0
Arachidonate 15-lipoxygenase B	LX15B_HUMAN	76 kDa	0	0	0	0	0	0	0	1	0
Deoxycytidine kinase	DCK_HUMAN	31 kDa	0	0	0	0	0	0	0	1	0
Putative N-acetylglucosamine-6-phosphate deacetylase	NAGA_HUMAN	44 kDa	0	0	0	0	0	0	0	1	0
Ubiquitin-conjugating enzyme E2 H	UBE2H_HUMAN	21 kDa	0	0	0	0	0	0	0	1	0
Ribonuclease 4	RNAS4_HUMAN	17 kDa	0	0	0	0	0	0	0	1	0
Insulin-like growth factor-binding protein 6	IBP6_HUMAN	25 kDa	0	0	0	0	0	0	0	1	0
Pro-cathepsin H	CATH_HUMAN	37 kDa	0	0	0	0	0	0	0	1	0
Putative uncharacterized protein encoded by LINC00615	CL037_HUMAN	15 kDa	0	0	0	0	0	0	0	1	0
Methylosome subunit pICln	ICLN_HUMAN	26 kDa	0	0	0	0	0	0	0	1	0
Septin-10	SEP10_HUMAN	53 kDa	0	0	0	0	0	0	0	1	0
Nucleolar protein 56	NOP56_HUMAN	66 kDa	0	0	0	0	0	0	0	1	0
Histone-binding protein RBBP4	RBBP4_HUMAN	48 kDa	0	0	0	0	0	0	0	1	0
DNA repair protein RAD50	RAD50_HUMAN	154 kDa	0	0	0	0	0	0	0	1	0
Calumenin	CALU_HUMAN	37 kDa	0	0	0	0	0	0	0	1	0
Putative deoxyribose-phosphate aldolase	DEOC_HUMAN	35 kDa	0	0	0	0	0	0	0	1	0
Transcription factor BTF3 homolog 4	BT3L4_HUMAN	17 kDa	0	0	0	0	0	0	0	1	0
Histone-lysine N-methyltransferase ASH1L	ASH1L_HUMAN	333 kDa	0	0	0	0	0	0	0	1	0
O-acetyl-ADP-ribose deacetylase 1	OARD1_HUMAN	17 kDa	0	0	0	0	0	0	0	1	0
Sec1 family domain-containing protein 1	SCFD1_HUMAN	72 kDa	0	0	0	0	0	0	0	1	0
UPF0587 protein C1orf123	CA123_HUMAN	18 kDa	0	0	0	0	0	0	0	1	0
Immunoglobulin J chain	IGJ_HUMAN	18 kDa	0	0	0	0	0	0	0	1	0
Angiotensinogen	ANGT_HUMAN	53 kDa	0	0	0	0	0	0	0	1	0
RNA-binding protein 8A	RBM8A_HUMAN	20 kDa	0	0	0	0	0	0	0	1	0
GMP synthase [glutamine-hydrolyzing]	GUAA_HUMAN	77 kDa	0	0	0	0	0	0	0	1	0
Rho-related GTP-binding protein RhoG	RHOG_HUMAN	21 kDa	0	0	0	0	0	0	0	1	0
Cytoplasmic dynein 1 light intermediate chain 2	DC1L2_HUMAN	54 kDa	0	0	0	0	0	0	0	1	0
Splicing factor U2AF 65 kDa subunit	U2AF2_HUMAN	54 kDa	0	0	0	0	0	0	0	1	0
Calcium-binding protein 39-like	CB39L_HUMAN	39 kDa	0	0	0	0	0	0	0	1	0
COP9 signalosome complex subunit 4	CSN4_HUMAN	46 kDa	0	0	0	0	0	0	0	1	0
Collagen alpha-1(XII) chain	COCA1_HUMAN	333 kDa	0	0	0	0	0	0	0	1	0
Structural maintenance of chromosomes protein 3	SMC3_HUMAN	142 kDa	0	0	0	0	0	0	0	1	0
Complement component C9	CO9_HUMAN	63 kDa	0	0	0	0	0	0	0	1	0
Coronin-1B	COR1B_HUMAN	54 kDa	0	0	0	0	0	0	0	1	0
Protein S100-A7	S10A7_HUMAN	11 kDa	0	0	0	0	0	0	0	1	0
Prothymosin alpha	PTMA_HUMAN	12 kDa	0	0	0	0	0	0	0	1	0
Selenide, water dikinase 1	SPS1_HUMAN	43 kDa	0	0	0	0	0	0	0	0	1
Endoplasmic reticulum-Golgi intermediate compartment protein 1	ERGI1_HUMAN	33 kDa	0	0	0	0	0	0	0	0	1
Arfaptin-1	ARFP1_HUMAN	42 kDa	0	0	0	0	0	0	0	0	1
Lactotransferrin	TRFL_HUMAN	78 kDa	0	0	0	0	0	0	0	0	1
Dedicator of cytokinesis protein 9	DOCK9_HUMAN	236 kDa	0	0	0	0	0	0	0	0	1
Retinoid-inducible serine carboxypeptidase	RISC_HUMAN	51 kDa	0	0	0	0	0	0	0	0	1
Dipeptidyl peptidase 2	DPP2_HUMAN	54 kDa	0	0	0	0	0	0	0	0	1
Lysosomal Pro-X carboxypeptidase	PCP_HUMAN	56 kDa	0	0	0	0	0	0	0	0	1
Pleckstrin homology domain-containing family H member 1	PKHH1_HUMAN	151 kDa	0	0	0	0	0	0	0	0	1
Cadherin-23	CAD23_HUMAN	369 kDa	0	0	0	0	0	0	0	0	1

Description	Accession	MW	Raw spectral counts								
			Frontal Cortex	Cerebellum	Right Ventricle	Mesentric lymph node	Liver	Pancreas	Proximal bile duct	Penis	Prostate
Tight junction protein ZO-1	ZO1_HUMAN	195 kDa	0	0	0	0	0	0	0	0	1
Collagen alpha-4(IV) chain	CO4A4_HUMAN	164 kDa	0	0	0	0	0	0	0	0	1
Poly(ADP-ribose) glycohydrolase	PARG_HUMAN	111 kDa	0	0	0	0	0	0	0	0	1
C2 calcium-dependent domain-containing protein 4B	C2C4B_HUMAN	39 kDa	0	0	0	0	0	0	0	0	1
RRP12-like protein	RRP12_HUMAN	144 kDa	0	0	0	0	0	0	0	0	1
Death-associated protein kinase 1	DAPK1_HUMAN	160 kDa	0	0	0	0	0	0	0	0	1
Peroxisomal acyl-coenzyme A oxidase 3	ACOX3_HUMAN	78 kDa	0	0	0	0	0	0	0	0	1
Lysine-rich nucleolar protein 1	KNOP1_HUMAN	52 kDa	0	0	0	0	0	0	0	0	1
Testin	TES_HUMAN	48 kDa	0	0	0	0	0	0	0	0	1
Histo-blood group ABO system transferase	BGAT_HUMAN	41 kDa	0	0	0	0	0	0	0	0	1
Phosphopantothenate--cysteine ligase	PPCS_HUMAN	34 kDa	0	0	0	0	0	0	0	0	1
Alpha-catulin	CTNL1_HUMAN	82 kDa	0	0	0	0	0	0	0	0	1
Transmembrane emp24 domain-containing protein 4	TMED4_HUMAN	26 kDa	0	0	0	0	0	0	0	0	1
Epididymal secretory protein E1	NPC2_HUMAN	17 kDa	0	0	0	0	0	0	0	0	1
Oxidoreductase HTATIP2	HTAI2_HUMAN	27 kDa	0	0	0	0	0	0	0	0	1
Mannose-6-phosphate isomerase	MPI_HUMAN	47 kDa	0	0	0	0	0	0	0	0	1
Solute carrier family 22 member 3	S22A3_HUMAN	61 kDa	0	0	0	0	0	0	0	0	1
COMM domain-containing protein 3	COMD3_HUMAN	22 kDa	0	0	0	0	0	0	0	0	1
Ras-related protein Rab-27A	RB27A_HUMAN	25 kDa	0	0	0	0	0	0	0	0	1
Methylthioribulose-1-phosphate dehydratase	MTNB_HUMAN	27 kDa	0	0	0	0	0	0	0	0	1
Zinc finger protein 600	ZN600_HUMAN	83 kDa	0	0	0	0	0	0	0	0	1
Zinc finger MYND domain-containing protein 11	ZMY11_HUMAN	71 kDa	0	0	0	0	0	0	0	0	1
P2X purinoceptor 4	P2RX4_HUMAN	43 kDa	0	0	0	0	0	0	0	0	1
LIM and senescent cell antigen-like-containing domain protein 2	LIMS2_HUMAN	39 kDa	0	0	0	0	0	0	0	0	1
Transforming growth factor beta-1-induced transcript 1 protein	TGFI1_HUMAN	50 kDa	0	0	0	0	0	0	0	0	1
Alpha-1,3/1,6-mannosyltransferase ALG2	ALG2_HUMAN	47 kDa	0	0	0	0	0	0	0	0	1
Dopamine beta-hydroxylase	DOPO_HUMAN	69 kDa	0	0	0	0	0	0	0	0	1
Splicing factor 3B subunit 6	SF3B6_HUMAN	15 kDa	0	0	0	0	0	0	0	0	1
cAMP-dependent protein kinase catalytic subunit alpha	KAPCA_HUMAN	41 kDa	0	0	0	0	0	0	0	0	1
Beta-2-glycoprotein 1	APOH_HUMAN	38 kDa	0	0	0	0	0	0	0	0	1
Spermine synthase	SPSY_HUMAN	41 kDa	0	0	1	0	0	0	0	0	0
PDZ and LIM domain protein 3	PDLI3_HUMAN	39 kDa	0	0	1	0	0	0	0	0	0
Haloacid dehalogenase-like hydrolase domain-containing protein 3	HDHD3_HUMAN	28 kDa	0	0	1	0	0	0	0	0	0
Protein phosphatase 1 regulatory subunit 12B	MYPT2_HUMAN	110 kDa	0	0	1	0	0	0	0	0	0
E3 ubiquitin-protein ligase LRSAM1	LRSM1_HUMAN	84 kDa	0	0	1	0	0	0	0	0	0
Serine/threonine-protein kinase SMG1	SMG1_HUMAN	410 kDa	0	0	1	0	0	0	0	0	0
ATP-binding cassette sub-family D member 3	ABCD3_HUMAN	75 kDa	0	0	1	0	0	0	0	0	0
Mitochondrial chaperone BCS1	BCS1_HUMAN	48 kDa	0	0	1	0	0	0	0	0	0
Nitrilase homolog 1	NIT1_HUMAN	36 kDa	0	0	1	0	0	0	0	0	0
UPF0598 protein C8orf82	CH082_HUMAN	24 kDa	0	0	1	0	0	0	0	0	0
Myosin light chain 6B	MYL6B_HUMAN	23 kDa	0	0	1	0	0	0	0	0	0
Phosphorylase b kinase regulatory subunit alpha, skeletal muscle isoform	KPB1_HUMAN	137 kDa	0	0	1	0	0	0	0	0	0
Hexaprenyldihydroxybenzoate methyltransferase	COQ3_HUMAN	41 kDa	0	0	1	0	0	0	0	0	0
Mth938 domain-containing protein	AAMDC_HUMAN	13 kDa	0	0	1	0	0	0	0	0	0
Serine-protein kinase ATM	ATM_HUMAN	351 kDa	0	0	1	0	0	0	0	0	0
Cytoplasmic dynein 2 heavy chain 1	DYHC2_HUMAN	493 kDa	0	0	1	0	0	0	0	0	0
39S ribosomal protein L12	RM12_HUMAN	21 kDa	0	0	1	0	0	0	0	0	0
Histone-lysine N-methyltransferase NSD2	NSD2_HUMAN	152 kDa	0	0	1	0	0	0	0	0	0
Protein furry homolog	FRY_HUMAN	339 kDa	0	0	1	0	0	0	0	0	0
Unconventional myosin-XV	MYO15_HUMAN	395 kDa	0	0	1	0	0	0	0	0	0

Description	Accession	MW	Raw spectral counts								
			Frontal Cortex	Cerebellum	Right Ventricle	Mesentric lymph node	Liver	Pancreas	Proximal bile duct	Penis	Prostate
FYVE, RhoGEF and PH domain-containing protein 6	FGD6_HUMAN	161 kDa	0	0	1	0	0	0	0	0	0
Putative ATP-dependent RNA helicase TDRD9	TDRD9_HUMAN	156 kDa	0	0	1	0	0	0	0	0	0
Rho GTPase-activating protein 42	RHG42_HUMAN	99 kDa	0	0	1	0	0	0	0	0	0
Pyruvate dehydrogenase phosphatase regulatory subunit	PDPR_HUMAN	99 kDa	0	0	1	0	0	0	0	0	0
STE20/SPS1-related proline-alanine-rich protein kinase	STK39_HUMAN	59 kDa	0	0	1	0	0	0	0	0	0
Probable glutamate--tRNA ligase	SYEM_HUMAN	59 kDa	0	0	1	0	0	0	0	0	0
Transcription factor AP-2-beta	AP2B_HUMAN	50 kDa	0	0	1	0	0	0	0	0	0
NADH dehydrogenase [ubiquinone] 1 beta subcomplex subunit 5	NDUB5_HUMAN	22 kDa	0	0	1	0	0	0	0	0	0
Integrin alpha-M	ITAM_HUMAN	127 kDa	0	0	1	0	0	0	0	0	0
Beta-parvin	PARVB_HUMAN	42 kDa	0	0	1	0	0	0	0	0	0
Tyrosine-protein kinase Fer	FER_HUMAN	95 kDa	0	0	1	0	0	0	0	0	0
Nuclear pore complex protein Nup107	NU107_HUMAN	106 kDa	0	0	1	0	0	0	0	0	0
5'-AMP-activated protein kinase subunit gamma-2	AAKG2_HUMAN	63 kDa	0	0	1	0	0	0	0	0	0
28S ribosomal protein S34	RT34_HUMAN	26 kDa	0	0	1	0	0	0	0	0	0
Mitochondrial calcium uniporter regulator 1	MCUR1_HUMAN	40 kDa	0	0	1	0	0	0	0	0	0
Laminin subunit beta-2	LAMB2_HUMAN	196 kDa	0	0	1	0	0	0	0	0	0
Serine beta-lactamase-like protein LACTB	LACTB_HUMAN	61 kDa	0	0	1	0	0	0	0	0	0
Cytochrome c oxidase assembly protein COX11	COX11_HUMAN	31 kDa	0	0	1	0	0	0	0	0	0
Kazrin	KAZRN_HUMAN	86 kDa	0	0	1	0	0	0	0	0	0
Oxysterol-binding protein-related protein 1	OSBL1_HUMAN	108 kDa	0	0	1	0	0	0	0	0	0
Rab GTPase-activating protein 1-like	RBG1L_HUMAN	93 kDa	0	0	1	0	0	0	0	0	0
Complex I assembly factor TIMMDC1	TIDC1_HUMAN	32 kDa	0	0	1	0	0	0	0	0	0
Ras-related protein Rab-12	RAB12_HUMAN	27 kDa	0	0	1	0	0	0	0	0	0
RNA polymerase II-associated protein 3	RPAP3_HUMAN	76 kDa	0	0	1	0	0	0	0	0	0
Kelch-like protein 41	KLH41_HUMAN	68 kDa	0	0	1	0	0	0	0	0	0
IQ domain-containing protein G	IQCG_HUMAN	52 kDa	0	0	1	0	0	0	0	0	0
Vacuolar protein-sorting-associated protein 36	VPS36_HUMAN	44 kDa	0	0	1	0	0	0	0	0	0
Spermatogenesis-associated protein 20	SPT20_HUMAN	88 kDa	0	0	1	0	0	0	0	0	0
39S ribosomal protein L47	RM47_HUMAN	29 kDa	0	0	1	0	0	0	0	0	0
Small G protein signaling modulator 1	SGSM1_HUMAN	130 kDa	0	0	1	0	0	0	0	0	0
Fanconi anemia-associated protein of 100 kDa	FP100_HUMAN	93 kDa	0	0	1	0	0	0	0	0	0
Nuclear pore complex-interacting protein family member B9	NPIB9_HUMAN	49 kDa	0	0	1	0	0	0	0	0	0
NADH dehydrogenase [ubiquinone] 1 beta subcomplex subunit 11	NDUBB_HUMAN	17 kDa	0	0	1	0	0	0	0	0	0
Bcl-2-like protein 13	B2L13_HUMAN	53 kDa	0	0	1	0	0	0	0	0	0
Polymerase delta-interacting protein 2	PDIP2_HUMAN	42 kDa	0	0	1	0	0	0	0	0	0
Mitochondrial import inner membrane translocase subunit TIM14	TIM14_HUMAN	12 kDa	0	0	1	0	0	0	0	0	0
ATP synthase-coupling factor 6	ATP5J_HUMAN	13 kDa	0	0	1	0	0	0	0	0	0
39S ribosomal protein L22	RM22_HUMAN	24 kDa	0	0	1	0	0	0	0	0	0
Uncharacterized protein C6orf136	CF136_HUMAN	36 kDa	0	0	1	0	0	0	0	0	0
39S ribosomal protein L18	RM18_HUMAN	21 kDa	0	0	1	0	0	0	0	0	0
Tryptophan--tRNA ligase	SYWM_HUMAN	40 kDa	0	0	1	0	0	0	0	0	0
28S ribosomal protein S10	RT10_HUMAN	23 kDa	0	0	1	0	0	0	0	0	0
DnaJ homolog subfamily B member 4	DNJB4_HUMAN	38 kDa	0	0	1	0	0	0	0	0	0
Thioredoxin reductase 2	TRXR2_HUMAN	57 kDa	0	0	1	0	0	0	0	0	0
28S ribosomal protein S35	RT35_HUMAN	37 kDa	0	0	1	0	0	0	0	0	0
ATP synthase mitochondrial F1 complex assembly factor 2	ATPF2_HUMAN	33 kDa	0	0	1	0	0	0	0	0	0
Heat shock protein beta-3	HSPB3_HUMAN	17 kDa	0	0	1	0	0	0	0	0	0
Small muscular protein	SMPX_HUMAN	10 kDa	0	0	1	0	0	0	0	0	0
Serine incorporator 1	SERC1_HUMAN	50 kDa	0	0	1	0	0	0	0	0	0
LYR motif-containing protein 5	LYRM5_HUMAN	11 kDa	0	0	1	0	0	0	0	0	0

Description	Accession	MW	Raw spectral counts								
			Frontal Cortex	Cerebellum	Right Ventricle	Mesentric lymph node	Liver	Pancreas	Proximal bile duct	Penis	Prostate
Mitochondrial antiviral-signaling protein	MAVS_HUMAN	57 kDa	0	0	1	0	0	0	0	0	0
6.8 kDa mitochondrial proteolipid	68MP_HUMAN	7 kDa	0	0	1	0	0	0	0	0	0
NADH dehydrogenase (ubiquinone) complex I, assembly factor 6	NDUF6_HUMAN	38 kDa	0	0	1	0	0	0	0	0	0
Carboxypeptidase N subunit 2	CPN2_HUMAN	61 kDa	0	0	1	0	0	0	0	0	0
Mitochondrial import inner membrane translocase subunit Tim8 B	TIM8B_HUMAN	9 kDa	0	0	1	0	0	0	0	0	0
Protein SCO1 homolog	SCO1_HUMAN	34 kDa	0	0	1	0	0	0	0	0	0
28S ribosomal protein S22	RT22_HUMAN	41 kDa	0	0	1	0	0	0	0	0	0
Vacuolar protein-sorting-associated protein 25	VPS25_HUMAN	21 kDa	0	0	1	0	0	0	0	0	0
Iron-sulfur protein NUBPL	NUBPL_HUMAN	34 kDa	0	0	1	0	0	0	0	0	0
[3-methyl-2-oxobutanoate dehydrogenase [lipoamide]] kinase	BCKD_HUMAN	46 kDa	0	0	1	0	0	0	0	0	0
Retinoid isomerohydrolase	RPE65_HUMAN	61 kDa	0	0	1	0	0	0	0	0	0
Serine protease HTRA2	HTRA2_HUMAN	49 kDa	0	0	1	0	0	0	0	0	0
Protein unc-45 homolog A	UN45A_HUMAN	103 kDa	0	0	1	0	0	0	0	0	0
Ribosome-releasing factor 2	RRF2M_HUMAN	87 kDa	0	0	1	0	0	0	0	0	0
Serum amyloid A-4 protein	SAA4_HUMAN	15 kDa	0	0	1	0	0	0	0	0	0
Succinate dehydrogenase [ubiquinone] cytochrome b small subunit	DHSD_HUMAN	17 kDa	0	0	1	0	0	0	0	0	0
39S ribosomal protein L20	RM20_HUMAN	17 kDa	0	0	1	0	0	0	0	0	0
Fructosamine-3-kinase	FN3K_HUMAN	35 kDa	0	0	1	0	0	0	0	0	0
O-acetyl-ADP-ribose deacetylase MACROD1	MACD1_HUMAN	36 kDa	0	0	1	0	0	0	0	0	0
Protein PET100 homolog	PT100_HUMAN	9 kDa	0	0	1	0	0	0	0	0	0
Coiled-coil domain-containing protein 132	CC132_HUMAN	111 kDa	0	0	1	0	0	0	0	0	0
Cathepsin Z	CATZ_HUMAN	34 kDa	0	0	1	0	0	0	0	0	0
Fibrinogen alpha chain	FIBA_HUMAN	95 kDa	0	0	1	0	0	0	0	0	0
von Willebrand factor	VWF_HUMAN	309 kDa	0	0	1	0	0	0	0	0	0
