# Supplementary material for: A Draft Map of Rhesus Monkey Tissue Proteome for Biomedical Research
Source: PLoS One. 2015 May 14;10(5):e0126243. doi: 10.1371/journal.pone.0126243 (PMC4431823; doi:10.1371/journal.pone.0126243)
Supplement: S4 Table — (PDF) [file pone.0126243.s006.pdf]

S4_Table The raw spectral counts acquired from proteomic analysis of multi-organ tissues of female rhesus monkey. List of proteins were acquired from ten tissues, frontal cortex, cerebellum, right ventricle, mesentric lymph node, liver, pancreas, proximal bile duct, breast, ovary and clitoris.

Description	Accession	MW	Raw spectral counts									
			Frontal cortex	Cerebellum	Right ventricle	Mesentric lymph node	Liver	Pancreas	Proximal bile duct	Breast	Ovary	Clitoris
Keratin, type II cytoskeletal 1	K2C1_HUMAN	66 kDa	325	233	372	220	532	458	228	227	203	222
Serum albumin	ALBU_HUMAN	69 kDa	15	17	98	238	51	49	236	155	149	241
Vimentin	VIME_HUMAN	54 kDa	20	16	15	201	17	18	173	82	188	185
Keratin, type II cytoskeletal 8	K2C8_HUMAN	54 kDa	12	0	0	0	59	104	34	81	15	22
Filamin-A	FLNA_HUMAN	281 kDa	1	1	8	251	3	26	435	78	111	140
Keratin, type I cytoskeletal 10	K1C10_HUMAN	59 kDa	231	73	254	65	362	284	50	78	53	83
Keratin, type II cytoskeletal 2 epidermal	K22E_HUMAN	65 kDa	105	82	132	103	236	156	65	73	56	92
Actin, cytoplasmic 1	ACTB_HUMAN	42 kDa	74	134	70	238	67	77	343	67	167	150
Keratin, type I cytoskeletal 19	K1C19_HUMAN	44 kDa	7	0	0	5	11	46	34	65	10	0
ATP synthase subunit beta	ATPB_HUMAN	57 kDa	84	148	187	40	73	140	72	65	41	44
Tubulin beta chain	TBB5_HUMAN	50 kDa	164	228	17	58	34	56	164	63	91	106
Actin, alpha cardiac muscle 1	ACTC_HUMAN	42 kDa	49	100	100	273	47	56	390	63	145	145
Tubulin alpha-1B chain	TBA1B_HUMAN	50 kDa	229	202	16	56	36	73	108	57	54	53
Keratin, type II cytoskeletal 6B	K2C6B_HUMAN	60 kDa	50	0	42	46	104	69	0	56	0	60
Keratin, type I cytoskeletal 9	K1C9_HUMAN	62 kDa	166	75	184	102	280	235	77	55	42	80
Clathrin heavy chain 1	CLH1_HUMAN	192 kDa	147	101	11	12	42	56	12	50	17	33
Tubulin beta-4B chain	TBB4B_HUMAN	50 kDa	155	242	15	0	35	56	138	50	72	86
Tubulin alpha-1A chain	TBA1A_HUMAN	50 kDa	217	206	10	55	21	46	119	49	48	52
Putative elongation factor 1-alpha-like 3	EF1A3_HUMAN	50 kDa	20	17	16	46	52	96	42	48	26	55
Neuroblast differentiation-associated protein AHNAK	AHNK_HUMAN	629 kDa	1	4	17	41	0	12	41	48	86	199
Spectrin alpha chain, non-erythrocytic 1	SPTN1_HUMAN	285 kDa	233	271	33	9	72	72	20	48	72	98
Keratin, type II cytoskeletal 5	K2C5_HUMAN	62 kDa	37	25	27	26	104	74	24	47	19	45
Annexin A2	ANXA2_HUMAN	39 kDa	3	0	23	136	7	33	173	46	117	121
Alpha-actinin-1	ACTN1_HUMAN	103 kDa	33	5	38	42	20	25	85	44	34	50
Tubulin beta-2B chain	TBB2B_HUMAN	50 kDa	160	202	12	47	28	47	132	44	60	77
Annexin A5	ANXA5_HUMAN	36 kDa	18	12	20	40	12	26	52	43	72	29
Keratin, type I cytoskeletal 14	K1C14_HUMAN	52 kDa	27	16	32	15	71	66	14	41	11	24
Hemoglobin subunit alpha	HBA_HUMAN	15 kDa	12	16	59	194	51	14	235	41	37	44
Alpha-actinin-4	ACTN4_HUMAN	105 kDa	17	5	24	39	35	24	59	40	30	58
Alpha-enolase	ENOA_HUMAN	47 kDa	37	77	13	17	25	21	38	38	34	48
Tubulin alpha-4A chain	TBA4A_HUMAN	50 kDa	163	136	12	0	26	0	0	38	0	36
Lumican	LUM_HUMAN	38 kDa	0	0	17	22	2	3	54	36	11	45
Heat shock protein HSP 90-alpha	HS90A_HUMAN	85 kDa	48	51	18	20	28	41	28	36	45	30
Keratin, type II cytoskeletal 6A	K2C6A_HUMAN	60 kDa	33	0	0	28	90	53	0	35	0	43
Hemoglobin subunit beta	HBB_HUMAN	16 kDa	17	17	37	274	30	9	274	35	37	51
Annexin A1	ANXA1_HUMAN	39 kDa	5	27	9	68	0	17	66	34	29	54
Heat shock protein HSP 90-beta	HS90B_HUMAN	83 kDa	40	44	19	22	42	59	23	34	51	22
Spectrin beta chain, non-erythrocytic 1	SPTB2_HUMAN	275 kDa	128	169	12	13	54	48	14	33	46	58
Hemoglobin subunit delta	HBD_HUMAN	16 kDa	16	13	35	268	31	8	241	33	26	49
Tubulin beta-4A chain	TBB4A_HUMAN	50 kDa	152	268	10	39	21	37	111	33	51	67
Transgelin	TAGL_HUMAN	23 kDa	0	0	18	134	0	3	117	32	53	29
Keratin, type II cytoskeletal 7	K2C7_HUMAN	51 kDa	0	0	0	0	0	11	0	31	0	0
14-3-3 protein epsilon	1433E_HUMAN	29 kDa	32	46	21	16	18	25	30	31	45	31
Endoplasmin	ENPL_HUMAN	92 kDa	12	16	10	10	75	114	10	31	35	14
Keratin, type I cytoskeletal 16	K1C16_HUMAN	51 kDa	29	0	0	14	62	49	0	30	0	23
14-3-3 protein zeta/delta	1433Z_HUMAN	28 kDa	34	35	14	16	15	14	31	30	31	33
Malate dehydrogenase	MDHM_HUMAN	36 kDa	36	65	67	48	20	20	39	30	25	23
Protein disulfide-isomerase A3	PDIA3_HUMAN	57 kDa	12	7	15	7	23	33	4	29	33	6

Description	Accession	MW	Raw spectral counts									
			Frontal cortex	Cerebellum	Right ventricle	Mesentric lymph node	Liver	Pancreas	Proximal bile duct	Breast	Ovary	Clitoris
Elongation factor 1-alpha 2	EF1A2_HUMAN	50 kDa	19	18	25	32	24	55	24	29	13	33
ATP synthase subunit alpha	ATPA_HUMAN	60 kDa	47	96	159	17	40	67	29	29	20	23
Filamin-B	FLNB_HUMAN	278 kDa	0	0	0	17	42	32	0	28	36	37
Heterogeneous nuclear ribonucleoproteins A2/B1	ROA2_HUMAN	37 kDa	8	28	9	8	15	25	10	27	42	13
Pyruvate kinase PKM	KPYM_HUMAN	58 kDa	61	55	43	6	0	11	20	27	36	29
Keratin, type I cytoskeletal 17	K1C17_HUMAN	48 kDa	19	0	0	0	19	22	0	26	0	12
Prolargin	PRELP_HUMAN	44 kDa	0	0	8	22	0	1	28	26	19	38
Talin-1	TLN1_HUMAN	270 kDa	3	0	7	28	37	5	28	26	38	51
ADP/ATP translocase 3	ADT3_HUMAN	33 kDa	33	46	53	12	21	19	17	26	3	16
Decorin	PGS2_HUMAN	40 kDa	0	0	6	9	0	0	17	25	13	39
Ig alpha-1 chain C region	IGHA1_HUMAN	38 kDa	2	0	18	22	13	2	23	25	23	18
60 kDa heat shock protein	CH60_HUMAN	61 kDa	33	39	59	12	102	44	6	25	19	4
Keratin, type I cytoskeletal 15	K1C15_HUMAN	49 kDa	0	0	0	0	0	0	0	24	0	0
N-acylethanolamine-hydrolyzing acid amidase	NAAA_HUMAN	40 kDa	0	0	0	0	0	0	0	23	0	0
Beta-actin-like protein 2	ACTBL_HUMAN	42 kDa	17	0	28	0	0	0	0	23	0	45
Heat shock cognate 71 kDa protein	HSP7C_HUMAN	71 kDa	48	75	24	27	35	27	16	23	49	30
ADP-ribosylation factor 3	ARF3_HUMAN	21 kDa	27	28	7	0	13	20	6	22	17	15
Vinculin	VINC_HUMAN	124 kDa	3	0	29	50	15	28	64	22	35	47
POTE ankyrin domain family member I	POTEI_HUMAN	121 kDa	28	34	0	80	34	30	121	22	39	45
Fatty acid-binding protein, adipocyte	FABP4_HUMAN	15 kDa	0	0	0	70	0	0	21	21	0	14
14-3-3 protein beta/alpha	1433B_HUMAN	28 kDa	20	20	9	10	12	12	20	21	29	23
Apolipoprotein A-I	APOA1_HUMAN	31 kDa	5	2	24	39	15	9	55	21	27	20
Asporin	ASPN_HUMAN	43 kDa	0	0	0	5	0	0	10	20	0	38
Protein disulfide-isomerase	PDIA1_HUMAN	57 kDa	4	3	9	5	19	83	1	20	14	2
Phosphoglycerate kinase 1	PGK1_HUMAN	45 kDa	25	37	17	15	16	17	27	20	29	26
L-lactate dehydrogenase B chain	LDHB_HUMAN	37 kDa	40	58	42	34	2	20	23	20	16	12
78 kDa glucose-regulated protein	GRP78_HUMAN	72 kDa	16	18	12	14	61	93	5	20	34	15
Keratin, type I cytoskeletal 13	K1C13_HUMAN	50 kDa	0	0	22	0	0	37	0	19	0	0
Ig lambda-3 chain C regions	LAC3_HUMAN	11 kDa	0	0	9	15	3	0	15	19	18	13
40S ribosomal protein S3	RS3_HUMAN	27 kDa	1	5	1	1	11	37	2	19	15	12
Gelsolin	GELS_HUMAN	86 kDa	7	3	10	14	0	3	16	19	14	23
14-3-3 protein gamma	1433G_HUMAN	28 kDa	23	26	12	12	11	8	24	19	23	20
Elongation factor 2	EF2_HUMAN	95 kDa	10	6	12	5	19	98	4	19	11	16
Histone H1.2	H12_HUMAN	21 kDa	8	36	18	22	20	27	33	19	105	30
Prelamin-A/C	LMNA_HUMAN	74 kDa	0	2	8	23	20	29	16	19	125	142
Xanthine dehydrogenase/oxidase	XDH_HUMAN	146 kDa	0	0	0	0	11	18	0	18	0	0
Alpha-1-antitrypsin	A1AT_HUMAN	47 kDa	2	0	8	26	7	4	21	18	20	26
Selenium-binding protein 1	SBP1_HUMAN	52 kDa	6	0	12	18	34	7	18	18	33	16
ADP/ATP translocase 2	ADT2_HUMAN	33 kDa	22	34	39	11	22	16	13	18	3	16
Tropomyosin alpha-4 chain	TPM4_HUMAN	29 kDa	11	8	20	24	9	15	44	18	45	23
Voltage-dependent anion-selective channel protein 1	VDAC1_HUMAN	31 kDa	20	22	22	5	6	11	9	17	7	4
Glucose-6-phosphate isomerase	G6PI_HUMAN	63 kDa	23	25	18	12	8	8	8	17	12	7
Profilin-1	PROF1_HUMAN	15 kDa	9	3	5	17	14	8	37	17	14	16
Prohibitin	PHB_HUMAN	30 kDa	14	12	19	5	20	21	12	17	15	8
Peroxiredoxin-1	PRDX1_HUMAN	22 kDa	16	15	8	11	7	12	15	17	41	12
Heterogeneous nuclear ribonucleoprotein K	HNRPK_HUMAN	51 kDa	21	27	9	0	14	33	1	17	22	12
Transitional endoplasmic reticulum ATPase	TERA_HUMAN	89 kDa	20	16	13	6	21	27	11	17	13	16
14-3-3 protein theta	1433T_HUMAN	28 kDa	16	25	9	8	7	13	18	17	31	23
Triosephosphate isomerase	TPIS_HUMAN	31 kDa	17	28	11	10	14	11	16	16	23	21
Serotransferrin	TRFE_HUMAN	77 kDa	1	0	18	33	10	13	20	16	38	23
Ubiquitin-40S ribosomal protein S27a	RS27A_HUMAN	18 kDa	14	14	20	12	26	27	24	16	26	34
Catalase	CATA_HUMAN	60 kDa	0	0	8	7	183	11	2	16	3	3

Description	Accession	MW	Raw spectral counts									
			Frontal cortex	Cerebellum	Right ventricle	Mesentric lymph node	Liver	Pancreas	Proximal bile duct	Breast	Ovary	Clitoris
Plectin	PLEC_HUMAN	532 kDa	28	48	3	3	23	11	9	16	15	131
Nucleolin	NUCL_HUMAN	77 kDa	5	6	1	0	4	20	1	15	18	8
Heterogeneous nuclear ribonucleoprotein A1	ROA1_HUMAN	39 kDa	4	10	1	2	3	10	6	15	17	11
40S ribosomal protein S18	RS18_HUMAN	18 kDa	3	7	3	0	9	21	3	15	10	11
Ubiquitin-like modifier-activating enzyme 1	UBA1_HUMAN	118 kDa	14	8	9	2	6	12	4	15	15	10
Phosphoglucomutase-1	PGM1_HUMAN	61 kDa	10	6	14	1	42	15	0	15	13	7
Transketolase	TKT_HUMAN	68 kDa	13	23	0	9	22	23	1	15	15	9
Trifunctional enzyme subunit alpha	ECHA_HUMAN	83 kDa	4	8	96	16	42	23	4	15	13	6
Glyceraldehyde-3-phosphate dehydrogenase	G3P_HUMAN	36 kDa	35	92	27	23	23	11	39	15	35	42
Lysosomal alpha-mannosidase	MA2B1_HUMAN	114 kDa	0	0	0	0	0	0	0	14	0	0
Ras GTPase-activating-like protein IQGAP1	IQGA1_HUMAN	189 kDa	0	0	0	0	0	16	0	14	9	33
Methylmalonate-semialdehyde dehydrogenase [acylating]	MMSA_HUMAN	58 kDa	3	2	10	2	24	15	0	14	6	1
Heterogeneous nuclear ribonucleoprotein A3	ROA3_HUMAN	40 kDa	5	12	1	0	2	11	0	14	22	13
Protein disulfide-isomerase A6	PDIA6_HUMAN	48 kDa	1	1	4	3	13	53	5	14	15	10
Fructose-bisphosphate aldolase A	ALDOA_HUMAN	39 kDa	30	21	16	12	2	10	9	14	9	11
Heat shock 70 kDa protein 1A/1B	HSP71_HUMAN	70 kDa	28	28	13	6	9	10	10	14	52	37
ADP/ATP translocase 1	ADT1_HUMAN	33 kDa	31	30	83	10	17	13	12	14	0	12
Keratin, type II cuticular Hb5	KRT85_HUMAN	56 kDa	11	0	0	0	0	0	0	13	0	0
Cathepsin D	CATD_HUMAN	45 kDa	5	5	7	5	5	5	4	13	9	7
ADP-ribosylation factor 4	ARF4_HUMAN	21 kDa	12	16	0	0	10	17	0	13	7	8
40S ribosomal protein SA	RSSA_HUMAN	33 kDa	6	1	5	4	10	39	5	13	11	14
Putative heat shock protein HSP 90-beta-3	H90B3_HUMAN	68 kDa	20	20	9	0	16	20	0	13	17	0
Histone H2A type 2-A	H2A2A_HUMAN	14 kDa	20	41	11	54	17	30	97	13	71	54
Keratin, type II cuticular Hb3	KRT83_HUMAN	54 kDa	11	0	0	0	0	0	0	12	0	0
Coatomer subunit alpha	COPA_HUMAN	138 kDa	0	0	0	0	1	24	1	12	3	1
Nucleoside diphosphate kinase A	NDKA_HUMAN	17 kDa	13	13	5	0	0	13	0	12	9	7
Peroxiredoxin-6	PRDX6_HUMAN	25 kDa	8	12	4	2	11	4	8	12	17	15
Transaldolase	TALDO_HUMAN	38 kDa	2	7	3	6	6	7	9	12	28	13
Adenosylhomocysteinase	SAHH_HUMAN	48 kDa	0	4	0	7	21	25	9	12	19	11
Prohibitin-2	PHB2_HUMAN	33 kDa	12	13	13	7	17	16	10	12	11	9
Cofilin-1	COF1_HUMAN	19 kDa	12	21	5	6	8	11	8	12	27	14
Phosphate carrier protein	MPCP_HUMAN	40 kDa	12	16	49	4	8	11	4	12	2	7
14-3-3 protein eta	1433F_HUMAN	28 kDa	16	21	8	8	0	10	19	12	24	16
Peroxiredoxin-2	PRDX2_HUMAN	22 kDa	21	28	14	15	12	13	19	12	32	8
Tropomyosin alpha-3 chain	TPM3_HUMAN	33 kDa	10	7	35	16	7	11	33	12	30	15
Tropomyosin alpha-1 chain	TPM1_HUMAN	33 kDa	8	4	57	26	6	8	49	12	40	15
Aconitate hydratase	ACON_HUMAN	85 kDa	26	52	91	16	11	28	14	12	5	8
Myelin protein P0	MYP0_HUMAN	28 kDa	0	0	0	2	0	0	4	11	0	34
14-3-3 protein sigma	1433S_HUMAN	28 kDa	13	0	0	0	0	0	0	11	0	31
Long-chain-fatty-acid--CoA ligase 1	ACSL1_HUMAN	78 kDa	0	0	14	12	47	0	0	11	0	2
Nucleoside diphosphate kinase B	NDKB_HUMAN	17 kDa	12	14	5	3	8	12	6	11	9	8
60S acidic ribosomal protein P0	RLA0_HUMAN	34 kDa	5	3	4	6	6	41	8	11	7	8
Malate dehydrogenase, cytoplasmic	MDHC_HUMAN	36 kDa	13	28	18	17	3	5	17	11	5	7
Polymerase I and transcript release factor	PTRF_HUMAN	43 kDa	0	0	17	42	0	2	33	11	24	36
Filamin-C	FLNC_HUMAN	291 kDa	0	0	32	28	0	4	53	11	16	21
Stress-70 protein	GRP75_HUMAN	74 kDa	22	25	30	11	30	17	2	11	16	8
Histone H4	H4_HUMAN	11 kDa	15	14	4	40	13	25	57	11	56	52
Beta-casein	CASB_HUMAN	25 kDa	0	0	0	0	0	0	0	10	0	0
Eukaryotic initiation factor 4A-I	IF4A1_HUMAN	46 kDa	0	0	0	0	6	7	0	10	7	0
Guanine nucleotide-binding protein subunit beta-2-like 1	GBLP_HUMAN	35 kDa	0	1	4	0	4	19	2	10	9	8
Thioredoxin-dependent peroxide reductase	PRDX3_HUMAN	28 kDa	7	9	11	11	10	4	8	10	7	2
Myelin P2 protein	MYP2_HUMAN	15 kDa	0	0	6	39	0	0	17	10	0	11

Description	Accession	MW	Raw spectral counts									
			Frontal cortex	Cerebellum	Right ventricle	Mesentric lymph node	Liver	Pancreas	Proximal bile duct	Breast	Ovary	Clitoris
Peroxiredoxin-5	PRDX5_HUMAN	22 kDa	19	17	13	2	3	20	0	10	7	1
ATP synthase subunit O	ATPO_HUMAN	23 kDa	17	19	23	5	12	8	9	10	8	7
3-hydroxyacyl-CoA dehydrogenase type-2	HCD2_HUMAN	27 kDa	7	12	10	3	56	16	0	10	7	6
Phosphatidylethanolamine-binding protein 1	PEBP1_HUMAN	21 kDa	20	34	10	6	16	9	13	10	36	7
Histone H2B type 1-J	H2B1J_HUMAN	14 kDa	10	24	3	31	6	20	50	10	44	36
Fatty acid synthase	FAS_HUMAN	273 kDa	0	0	0	233	59	1	20	10	1	10
Lactadherin	MFGM_HUMAN	43 kDa	0	0	0	0	0	0	0	9	0	0
40S ribosomal protein S14	RS14_HUMAN	16 kDa	4	2	3	3	8	16	2	9	3	7
Eukaryotic translation initiation factor 5A-1	IF5A1_HUMAN	17 kDa	1	7	1	3	7	16	6	9	1	7
UTP--glucose-1-phosphate uridylyltransferase	UGPA_HUMAN	57 kDa	8	3	3	7	34	2	1	9	6	2
Keratin, type I cytoskeletal 18	K1C18_HUMAN	48 kDa	0	0	0	0	28	51	0	9	0	0
Peptidyl-prolyl cis-trans isomerase B	PPIB_HUMAN	24 kDa	5	5	5	5	9	18	5	9	23	5
Calnexin	CALX_HUMAN	68 kDa	6	12	6	2	9	25	1	9	19	9
Electron transfer flavoprotein subunit beta	ETFB_HUMAN	28 kDa	6	6	27	10	26	12	8	9	7	7
Aspartate aminotransferase	AATM_HUMAN	48 kDa	13	29	29	2	23	9	5	9	8	7
L-lactate dehydrogenase A chain	LDHA_HUMAN	37 kDa	21	14	5	20	25	9	18	9	11	17
Cytoplasmic dynein 1 heavy chain 1	DYHC1_HUMAN	532 kDa	41	79	4	5	1	10	7	9	16	26
Programmed cell death protein 4	PDCD4_HUMAN	52 kDa	0	0	0	0	0	0	0	8	3	0
Keratin, type I cuticular Ha1	K1H1_HUMAN	47 kDa	11	0	0	0	0	0	0	8	0	0
Fatty acid-binding protein, heart	FABPH_HUMAN	15 kDa	4	1	10	0	0	0	0	8	0	0
T-complex protein 1 subunit eta	TCPH_HUMAN	59 kDa	5	4	3	1	0	2	0	8	3	0
Biliverdin reductase A	BIEA_HUMAN	33 kDa	0	2	3	0	0	3	4	8	10	5
Heterogeneous nuclear ribonucleoprotein H	HNRH1_HUMAN	49 kDa	0	8	0	0	2	5	0	8	12	0
40S ribosomal protein S8	RS8_HUMAN	24 kDa	0	3	0	0	4	15	0	8	5	0
60S ribosomal protein L7	RL7_HUMAN	29 kDa	1	1	1	0	6	17	0	8	5	3
Eukaryotic initiation factor 4A-II	IF4A2_HUMAN	46 kDa	1	6	3	1	6	6	1	8	10	7
Ferritin heavy chain	FRIH_HUMAN	21 kDa	12	11	0	2	10	0	0	8	2	4
Proteasome activator complex subunit 1	PSME1_HUMAN	29 kDa	0	0	3	0	11	9	2	8	12	6
Nucleophosmin	NPM_HUMAN	33 kDa	4	3	0	0	4	18	2	8	7	6
Adenylate kinase 2	KAD2_HUMAN	26 kDa	0	0	9	0	11	11	0	8	7	7
Hydroxyacyl-coenzyme A dehydrogenase	HCDH_HUMAN	34 kDa	2	0	13	9	10	2	4	8	4	3
Heterogeneous nuclear ribonucleoproteins C1/C2	HNRPC_HUMAN	34 kDa	2	12	2	0	5	9	2	8	14	2
Heterogeneous nuclear ribonucleoprotein U	HNRPU_HUMAN	91 kDa	5	12	1	0	4	9	0	8	18	3
Vacuolar protein sorting-associated protein 35	VPS35_HUMAN	92 kDa	16	11	3	0	1	18	1	8	2	5
T-complex protein 1 subunit beta	TCPB_HUMAN	57 kDa	17	12	10	2	5	6	1	8	5	1
High mobility group protein B1	HMGB1_HUMAN	25 kDa	2	10	4	2	3	8	5	8	16	10
Alpha-2-macroglobulin	A2MG_HUMAN	163 kDa	0	0	12	17	2	0	16	8	1	19
Isocitrate dehydrogenase [NADP] cytoplasmic	IDHC_HUMAN	47 kDa	0	0	2	8	22	4	10	8	11	10
Flavin reductase (NADPH)	BLVRB_HUMAN	22 kDa	5	0	6	13	16	4	10	8	5	8
Glutathione S-transferase P	GSTP1_HUMAN	23 kDa	2	4	8	9	4	7	10	8	17	8
Peptidyl-prolyl cis-trans isomerase A	PPIA_HUMAN	18 kDa	9	18	2	2	2	3	4	8	20	10
ATP synthase subunit gamma	ATPG_HUMAN	33 kDa	7	13	20	3	7	5	7	8	4	6
Phosphoglycerate mutase 1	PGAM1_HUMAN	29 kDa	11	22	8	8	5	12	15	8	6	18
EH domain-containing protein 2	EHD2_HUMAN	61 kDa	0	0	16	35	0	0	14	8	22	22
Glycogen phosphorylase, brain form	PYGB_HUMAN	97 kDa	17	25	40	2	7	4	5	8	5	7
Trifunctional enzyme subunit beta	ECHB_HUMAN	51 kDa	2	5	41	16	16	12	13	8	8	5
Cytochrome b-c1 complex subunit 2	QCR2_HUMAN	48 kDa	14	21	35	8	14	8	10	8	6	7
Collagen alpha-1(XIV) chain	COEA1_HUMAN	194 kDa	0	0	0	30	0	0	57	8	97	83
Neprilysin	NEP_HUMAN	86 kDa	0	0	0	0	0	0	0	7	0	0
Cathepsin Z	CATZ_HUMAN	34 kDa	0	0	1	0	2	0	0	7	0	1
Keratin, type II cuticular Hb2	KRT82_HUMAN	57 kDa	4	0	0	0	0	0	0	7	0	0
Calcyphosin	CAYP1_HUMAN	21 kDa	0	0	0	0	0	3	0	7	0	2

Description	Accession	MW	Raw spectral counts									
			Frontal cortex	Cerebellum	Right ventricle	Mesentric lymph node	Liver	Pancreas	Proximal bile duct	Breast	Ovary	Clitoris
Protein SET	SET_HUMAN	33 kDa	2	3	2	0	3	3	2	7	7	2
Heterogeneous nuclear ribonucleoprotein H2	HNRH2_HUMAN	49 kDa	3	5	1	0	1	4	0	7	9	3
Mimecan	MIME_HUMAN	34 kDa	0	0	2	2	0	0	10	7	5	10
40S ribosomal protein S10	RS10_HUMAN	19 kDa	2	2	2	0	4	14	1	7	4	3
T-complex protein 1 subunit delta	TCPD_HUMAN	58 kDa	6	6	8	0	4	3	1	7	4	0
Ras-related protein Rab-11B	RB11B_HUMAN	24 kDa	7	4	2	5	1	2	5	7	4	3
6-phosphogluconate dehydrogenase, decarboxylating	6PGD_HUMAN	53 kDa	1	0	0	4	7	2	5	7	8	8
Polypyrimidine tract-binding protein 1	PTBP1_HUMAN	57 kDa	1	0	0	0	7	15	0	7	6	9
Transgelin-2	TAGL2_HUMAN	22 kDa	0	0	4	7	3	5	8	7	8	7
60S ribosomal protein L7a	RL7A_HUMAN	30 kDa	1	3	0	1	8	18	1	7	9	6
X-ray repair cross-complementing protein 5	XRCC5_HUMAN	83 kDa	2	10	1	0	4	10	2	7	11	9
Rho GDP-dissociation inhibitor 1	GDIR1_HUMAN	23 kDa	6	4	2	2	6	8	4	7	9	9
60S ribosomal protein L18	RL18_HUMAN	22 kDa	1	3	0	0	13	25	0	7	5	5
Ras-related protein Rab-7a	RAB7A_HUMAN	23 kDa	9	6	4	0	9	8	0	7	14	6
40S ribosomal protein S2	RS2_HUMAN	31 kDa	4	3	2	2	11	25	1	7	4	6
Neutral alpha-glucosidase AB	GANAB_HUMAN	107 kDa	2	5	3	3	16	10	1	7	18	6
Complement C3	CO3_HUMAN	187 kDa	0	0	7	10	4	0	12	7	9	29
Dolichyl-diphosphooligosaccharide--protein glycosyltransferase subunit 1	RPN1_HUMAN	69 kDa	0	0	1	0	17	47	0	7	6	0
Dolichyl-diphosphooligosaccharide--protein glycosyltransferase subunit 2	RPN2_HUMAN	69 kDa	0	0	0	0	11	55	1	7	4	1
Moesin	MOES_HUMAN	68 kDa	4	7	5	10	2	6	6	7	13	23
Voltage-dependent anion-selective channel protein 3	VDAC3_HUMAN	31 kDa	9	17	14	2	4	6	11	7	9	4
Rab GDP dissociation inhibitor beta	GDIB_HUMAN	51 kDa	12	19	2	4	7	11	10	7	12	13
Coiled-coil domain-containing protein 180	CC180_HUMAN	191 kDa	9	8	5	23	5	0	29	7	14	13
Voltage-dependent anion-selective channel protein 2	VDAC2_HUMAN	32 kDa	16	35	39	9	8	10	12	7	10	5
Annexin A4	ANXA4_HUMAN	36 kDa	2	0	2	7	13	21	89	7	9	13
Polymeric immunoglobulin receptor	PIGR_HUMAN	83 kDa	0	0	0	0	1	0	0	6	0	0
Ig gamma-1 chain C region	IGHG1_HUMAN	36 kDa	0	1	4	4	0	0	3	6	4	6
Proliferation-associated protein 2G4	PA2G4_HUMAN	44 kDa	0	1	0	1	0	7	2	6	9	5
Ig kappa chain V-I region Gal	KV107_HUMAN	12 kDa	0	0	2	7	2	2	7	6	3	3
40S ribosomal protein S19	RS19_HUMAN	16 kDa	2	2	2	0	2	13	0	6	5	0
Far upstream element-binding protein 2	FUBP2_HUMAN	73 kDa	2	10	0	0	0	1	0	6	12	2
60S ribosomal protein L11	RL11_HUMAN	20 kDa	2	2	2	0	3	11	0	6	5	3
Cystatin-B	CYTB_HUMAN	11 kDa	2	2	3	4	3	2	5	6	4	4
40S ribosomal protein S16	RS16_HUMAN	16 kDa	1	2	1	0	1	15	0	6	8	4
Vesicle-trafficking protein SEC22b	SC22B_HUMAN	25 kDa	4	6	0	0	3	8	1	6	10	0
Proteasome subunit alpha type-7	PSA7_HUMAN	28 kDa	4	1	4	0	7	4	0	6	6	7
40S ribosomal protein S3a	RS3A_HUMAN	30 kDa	3	1	3	0	3	14	1	6	4	5
Septin-2	SEPT2_HUMAN	41 kDa	10	2	1	0	0	0	4	6	10	8
Elongation factor 1-delta	EF1D_HUMAN	31 kDa	0	0	3	1	2	16	2	6	7	4
Catenin alpha-1	CTNA1_HUMAN	100 kDa	2	0	2	0	9	14	0	6	5	5
Destrin	DEST_HUMAN	19 kDa	2	1	4	7	1	6	9	6	3	5
40S ribosomal protein S17-like	RS17L_HUMAN	16 kDa	0	2	2	0	7	19	1	6	3	4
ATP synthase subunit g	ATP5L_HUMAN	11 kDa	5	1	18	0	6	4	0	6	3	3
40S ribosomal protein S7	RS7_HUMAN	22 kDa	3	3	2	1	6	22	4	6	4	2
Heterogeneous nuclear ribonucleoprotein D0	HNRPD_HUMAN	38 kDa	4	9	0	1	2	6	0	6	19	7
AP-1 complex subunit beta-1	AP1B1_HUMAN	105 kDa	20	13	0	0	10	5	0	6	0	0
Serine/threonine-protein phosphatase 2A 65 kDa regulatory subunit A	2AAA_HUMAN	65 kDa	22	11	10	0	1	3	1	6	3	3
40S ribosomal protein S4, X isoform	RS4X_HUMAN	30 kDa	2	3	3	2	10	18	2	6	10	6
Cysteine and glycine-rich protein 1	CSRP1_HUMAN	21 kDa	4	3	2	10	2	5	18	6	8	6
Superoxide dismutase [Mn]	SODM_HUMAN	25 kDa	6	13	8	5	9	5	3	6	7	4
T-complex protein 1 subunit theta	TCPQ_HUMAN	60 kDa	14	13	10	2	4	8	0	6	11	0
Ras-related protein Rap-1b	RAP1B_HUMAN	21 kDa	8	10	6	6	6	5	9	6	7	6

Description	Accession	MW	Raw spectral counts									
			Frontal cortex	Cerebellum	Right ventricle	Mesentric lymph node	Liver	Pancreas	Proximal bile duct	Breast	Ovary	Clitoris
Calreticulin	CALR_HUMAN	48 kDa	6	1	3	1	22	12	6	6	11	2
3-ketoacyl-CoA thiolase	THIM_HUMAN	42 kDa	0	0	12	12	30	11	3	6	4	1
Enoyl-CoA hydratase	ECHM_HUMAN	31 kDa	5	8	12	9	18	14	3	6	7	6
Electron transfer flavoprotein subunit alpha	ETFA_HUMAN	35 kDa	3	7	14	7	24	11	7	6	4	7
Glutamate dehydrogenase 1	DHE3_HUMAN	61 kDa	20	25	17	5	41	10	5	6	8	9
NAD(P) transhydrogenase	NNTM_HUMAN	114 kDa	13	12	78	1	22	12	1	6	2	3
Collagen alpha-3(VI) chain	CO6A3_HUMAN	344 kDa	0	0	19	11	0	0	27	6	17	145
Keratin, type I cuticular Ha3-II	KT33B_HUMAN	46 kDa	0	0	0	0	0	0	0	5	0	0
Beta-hexosaminidase subunit beta	HEXB_HUMAN	63 kDa	0	0	0	0	0	0	0	5	0	0
PRA1 family protein 3	PRAF3_HUMAN	22 kDa	0	1	3	0	0	0	0	5	0	1
Ig heavy chain V-III region TEI	HV316_HUMAN	13 kDa	0	0	2	2	0	0	0	5	1	2
Acid ceramidase	ASAH1_HUMAN	45 kDa	1	0	3	0	0	1	0	5	2	0
RuvB-like 1	RUVB1_HUMAN	50 kDa	0	1	0	0	2	0	0	5	2	3
60S ribosomal protein L35	RL35_HUMAN	15 kDa	2	0	1	0	2	3	0	5	2	1
Tryptase delta	TRYD_HUMAN	27 kDa	0	0	0	0	0	0	1	5	0	12
60S ribosomal protein L14	RL14_HUMAN	23 kDa	0	0	1	0	3	5	0	5	4	2
Coatomer subunit beta	COPB_HUMAN	107 kDa	0	0	1	0	1	12	0	5	0	1
Lupus La protein	LA_HUMAN	47 kDa	0	0	0	0	1	5	2	5	5	4
Tetratricopeptide repeat protein 38	TTC38_HUMAN	53 kDa	0	0	0	0	8	4	0	5	3	3
Spliceosome RNA helicase DDX39B	DX39B_HUMAN	49 kDa	0	4	0	0	0	1	0	5	9	5
D-dopachrome decarboxylase	DOPD_HUMAN	13 kDa	2	0	0	0	6	3	4	5	2	3
Calpain-1 catalytic subunit	CAN1_HUMAN	82 kDa	2	2	2	1	1	6	1	5	4	4
Alpha-centractin	ACTZ_HUMAN	43 kDa	6	6	2	1	0	3	2	5	3	1
Transmembrane emp24 domain-containing protein 10	TMEDA_HUMAN	25 kDa	0	1	0	0	3	12	1	5	6	2
UMP-CMP kinase	KCY_HUMAN	22 kDa	2	2	3	0	6	7	3	5	1	2
Synaptic vesicle membrane protein VAT-1 homolog	VAT1_HUMAN	42 kDa	0	2	0	3	2	4	4	5	8	4
40S ribosomal protein S20	RS20_HUMAN	13 kDa	2	3	3	3	2	6	2	5	3	3
Ras-related protein Rab-5C	RAB5C_HUMAN	23 kDa	7	9	0	0	0	2	0	5	7	4
Elongation factor 1-gamma	EF1G_HUMAN	50 kDa	2	3	1	0	4	13	0	5	5	2
40S ribosomal protein S13	RS13_HUMAN	17 kDa	2	6	1	0	4	13	0	5	4	4
Proteasome subunit alpha type-6	PSA6_HUMAN	27 kDa	4	3	5	2	4	4	1	5	8	5
Ras-related protein Rab-2A	RAB2A_HUMAN	24 kDa	8	10	2	2	3	7	0	5	2	3
Cell division control protein 42 homolog	CDC42_HUMAN	21 kDa	6	4	3	2	7	9	2	5	2	2
Coatomer subunit gamma-1	COPG1_HUMAN	98 kDa	1	0	0	0	8	27	0	5	1	1
40S ribosomal protein S9	RS9_HUMAN	23 kDa	2	3	2	0	6	15	0	5	9	2
Endoplasmic reticulum resident protein 29	ERP29_HUMAN	29 kDa	0	5	1	0	12	12	0	5	7	4
Caveolin-1	CAV1_HUMAN	20 kDa	0	0	10	14	0	2	4	5	2	11
10 kDa heat shock protein	CH10_HUMAN	11 kDa	3	0	7	5	9	7	4	5	1	8
Antithrombin-III	ANT3_HUMAN	53 kDa	0	0	7	9	3	1	10	5	15	5
Elongation factor Tu	EFTU_HUMAN	50 kDa	5	11	13	2	10	2	0	5	8	2
Ras-related protein Rab-1A	RAB1A_HUMAN	23 kDa	12	9	3	1	6	15	2	5	3	2
Guanine nucleotide-binding protein G(I)/G(S)/G(T) subunit beta-2	GBB2_HUMAN	37 kDa	13	19	4	3	0	3	5	5	3	4
Mitochondrial inner membrane protein	IMMT_HUMAN	84 kDa	6	10	28	0	4	6	0	5	1	1
Hypoxia up-regulated protein 1	HYOU1_HUMAN	111 kDa	6	2	0	0	10	38	0	5	4	1
Dihydrolipoyl dehydrogenase	DLDH_HUMAN	54 kDa	7	6	28	9	8	6	6	5	5	3
Cullin-associated NEDD8-dissociated protein 1	CAND1_HUMAN	136 kDa	32	19	0	0	3	6	2	5	17	6
Myosin regulatory light polypeptide 9	MYL9_HUMAN	20 kDa	2	2	5	6	7	6	40	5	18	5
Myosin light polypeptide 6	MYL6_HUMAN	17 kDa	5	3	5	9	5	5	48	5	13	11
Annexin A6	ANXA6_HUMAN	76 kDa	23	31	24	27	30	26	18	5	49	26
Retinoid-inducible serine carboxypeptidase	RISC_HUMAN	51 kDa	0	0	0	0	0	0	0	4	0	0
Dipeptidyl peptidase 2	DPP2_HUMAN	54 kDa	0	0	0	0	0	0	0	4	0	0
Beta-galactosidase	BGAL_HUMAN	76 kDa	0	0	0	0	0	0	0	4	0	0

Description	Accession	MW	Raw spectral counts									
			Frontal cortex	Cerebellum	Right ventricle	Mesentric lymph node	Liver	Pancreas	Proximal bile duct	Breast	Ovary	Clitoris
Protein-arginine deiminase type-2	PADI2_HUMAN	76 kDa	1	0	0	0	0	0	0	4	0	0
Protein S100-A9	S10A9_HUMAN	13 kDa	0	0	0	0	1	0	0	4	0	2
Aminopeptidase N	AMPN_HUMAN	110 kDa	0	0	0	0	2	1	0	4	0	0
Catenin beta-1	CTNB1_HUMAN	85 kDa	1	0	0	0	0	1	1	4	1	0
Keratin, type I cuticular Ha5	KRT35_HUMAN	50 kDa	4	0	0	0	0	0	0	4	0	0
Valine--tRNA ligase	SYVC_HUMAN	140 kDa	0	0	0	0	0	6	0	4	0	0
60S ribosomal protein L38	RL38_HUMAN	8 kDa	0	0	0	0	2	6	0	4	0	0
Small nuclear ribonucleoprotein E	RUXE_HUMAN	11 kDa	0	0	0	0	1	1	0	4	4	3
Peptidyl-prolyl cis-trans isomerase FKBP11	FKB11_HUMAN	22 kDa	0	0	0	0	1	11	0	4	0	0
Hsc70-interacting protein	F10A1_HUMAN	41 kDa	2	0	3	0	1	0	0	4	5	2
60S ribosomal protein L27a	RL27A_HUMAN	17 kDa	0	0	0	0	2	9	0	4	4	0
F-actin-capping protein subunit alpha-1	CAZA1_HUMAN	33 kDa	3	0	0	0	0	4	4	4	3	5
Mitogen-activated protein kinase 1	MK01_HUMAN	41 kDa	9	4	0	0	0	1	0	4	2	3
60S acidic ribosomal protein P2	RLA2_HUMAN	12 kDa	0	3	1	1	2	6	0	4	7	0
Microfibril-associated glycoprotein 4	MFAP4_HUMAN	29 kDa	0	0	1	2	0	0	7	4	3	8
40S ribosomal protein S24	RS24_HUMAN	15 kDa	0	4	0	0	4	9	0	4	4	0
Proteasome subunit beta type-2	PSB2_HUMAN	23 kDa	1	2	2	2	4	4	2	4	2	3
60S ribosomal protein L27	RL27_HUMAN	16 kDa	0	1	1	0	4	12	0	4	2	3
Protein S100-A11	S10AB_HUMAN	12 kDa	0	0	1	2	1	1	3	4	3	14
F-actin-capping protein subunit alpha-2	CAZA2_HUMAN	33 kDa	4	2	2	0	2	3	5	4	3	4
Proteasome subunit alpha type-1	PSA1_HUMAN	30 kDa	2	2	5	0	7	2	1	4	2	6
Kinesin-1 heavy chain	KINH_HUMAN	110 kDa	5	7	2	0	0	7	0	4	2	5
Heterogeneous nuclear ribonucleoprotein D-like	HNRDL_HUMAN	46 kDa	5	4	0	0	0	4	0	4	10	5
60S ribosomal protein L13	RL13_HUMAN	24 kDa	0	1	0	0	2	18	0	4	4	3
40S ribosomal protein S6	RS6_HUMAN	29 kDa	0	2	1	0	3	14	0	4	6	2
T-complex protein 1 subunit zeta	TCPZ_HUMAN	58 kDa	5	5	5	0	5	4	1	4	2	1
NADH dehydrogenase [ubiquinone] iron-sulfur protein 8	NDUS8_HUMAN	24 kDa	5	7	5	1	2	2	3	4	3	0
Chloride intracellular channel protein 1	CLIC1_HUMAN	27 kDa	0	0	1	2	2	4	3	4	11	7
40S ribosomal protein S25	RS25_HUMAN	14 kDa	4	2	3	0	5	9	0	4	5	2
60S ribosomal protein L9	RL9_HUMAN	22 kDa	0	2	1	0	5	19	0	4	2	2
Alcohol dehydrogenase [NADP(+)]	AK1A1_HUMAN	37 kDa	3	0	0	1	9	9	2	4	5	3
Dolichyl-diphosphooligosaccharide--protein glycosyltransferase 48 kDa subunit	OST48_HUMAN	51 kDa	0	0	0	0	7	16	6	4	3	0
Fatty acid-binding protein 9	FABP9_HUMAN	15 kDa	2	0	1	21	0	0	4	4	1	4
Probable ATP-dependent RNA helicase DDX17	DDX17_HUMAN	80 kDa	1	11	0	1	3	2	0	4	12	3
Protein NDRG2	NDRG2_HUMAN	41 kDa	6	10	2	0	8	3	2	4	3	0
Heterogeneous nuclear ribonucleoprotein M	HNRPM_HUMAN	78 kDa	3	9	0	0	1	4	0	4	15	4
Interleukin enhancer-binding factor 2	ILF2_HUMAN	43 kDa	2	6	0	0	6	4	0	4	14	5
Pyruvate dehydrogenase E1 component subunit alpha, somatic form	ODPA_HUMAN	43 kDa	8	6	17	3	1	0	0	4	2	0
Extended synaptotagmin-1	ESYT1_HUMAN	123 kDa	0	0	2	4	4	5	10	4	6	7
Erlin-2	ERLN2_HUMAN	38 kDa	0	0	0	2	8	10	1	4	15	3
V-type proton ATPase subunit B, brain isoform	VATB2_HUMAN	57 kDa	26	16	0	0	0	0	0	4	1	1
Poly(rC)-binding protein 1	PCBP1_HUMAN	37 kDa	3	3	1	0	5	18	0	4	9	6
Importin subunit beta-1	IMB1_HUMAN	97 kDa	12	3	3	1	7	5	2	4	8	4
Ras GTPase-activating-like protein IQGAP2	IQGA2_HUMAN	181 kDa	0	0	0	0	36	14	0	4	0	0
Cytochrome b-c1 complex subunit 1	QCR1_HUMAN	53 kDa	4	13	21	4	5	2	4	4	2	1
Fumarate hydratase	FUMH_HUMAN	55 kDa	6	4	22	3	16	4	0	4	1	0
Staphylococcal nuclease domain-containing protein 1	SND1_HUMAN	102 kDa	1	0	0	0	6	45	0	4	1	4
Puromycin-sensitive aminopeptidase	PSA_HUMAN	103 kDa	18	11	11	1	2	4	3	4	6	3
Mitochondrial 2-oxoglutarate/malate carrier protein	M2OM_HUMAN	34 kDa	10	17	19	0	10	2	1	4	0	3
Succinate dehydrogenase [ubiquinone] flavoprotein subunit	SDHA_HUMAN	73 kDa	4	6	25	2	20	8	1	4	2	1
Citrate synthase	CISY_HUMAN	52 kDa	5	11	16	16	2	3	10	4	5	4
AP-2 complex subunit beta	AP2B1_HUMAN	105 kDa	43	20	1	0	4	1	0	4	4	3

Description	Accession	MW	Raw spectral counts									
			Frontal cortex	Cerebellum	Right ventricle	Mesentric lymph node	Liver	Pancreas	Proximal bile duct	Breast	Ovary	Clitoris
Isocitrate dehydrogenase [NAD] subunit alpha	IDH3A_HUMAN	40 kDa	17	24	15	2	3	3	2	4	5	6
Protein disulfide-isomerase A4	PDIA4_HUMAN	73 kDa	0	0	0	0	26	38	0	4	12	3
2-oxoglutarate dehydrogenase	ODO1_HUMAN	116 kDa	8	10	50	3	4	2	3	4	1	4
Ribosome-binding protein 1	RRBP1_HUMAN	152 kDa	0	0	0	0	19	70	0	4	0	0
Hexokinase-1	HXK1_HUMAN	102 kDa	23	58	20	0	0	0	5	4	0	10
NADH-ubiquinone oxidoreductase 75 kDa subunit	NDUS1_HUMAN	79 kDa	17	28	57	3	6	11	0	4	3	3
Glutathione S-transferase Mu 2	GSTM2_HUMAN	26 kDa	6	8	10	8	25	12	16	4	40	6
Heat shock protein beta-1	HSPB1_HUMAN	23 kDa	5	5	13	22	5	3	26	4	37	19
Isocitrate dehydrogenase [NADP]	IDHP_HUMAN	51 kDa	12	16	63	12	22	25	8	4	4	6
Fructose-bisphosphate aldolase C	ALDOC_HUMAN	39 kDa	24	82	18	22	1	3	10	4	4	7
Signal transducer and activator of transcription 5B	STA5B_HUMAN	90 kDa	0	0	0	0	0	0	0	3	0	0
Neutrophil gelatinase-associated lipocalin	NGAL_HUMAN	23 kDa	0	0	0	0	0	0	0	3	0	0
Lysozyme C	LYSC_HUMAN	17 kDa	0	1	0	0	1	0	0	3	0	0
Golgi apparatus protein 1	GSLG1_HUMAN	135 kDa	0	0	0	0	0	2	0	3	0	0
Lactotransferrin	TRFL_HUMAN	78 kDa	0	0	0	0	3	0	0	3	0	0
Cathepsin B	CATB_HUMAN	38 kDa	0	0	1	0	2	0	0	3	1	0
Transmembrane emp24 domain-containing protein 2	TMED2_HUMAN	23 kDa	0	0	0	0	1	4	0	3	0	0
RuvB-like 2	RUVB2_HUMAN	51 kDa	0	1	0	0	0	0	0	3	4	2
Ig mu chain C region	IGHM_HUMAN	49 kDa	0	0	0	2	1	0	2	3	1	3
UDP-glucose 4-epimerase	GALE_HUMAN	38 kDa	0	0	0	0	2	7	0	3	0	0
Dolichyl-diphosphooligosaccharide--protein glycosyltransferase subunit DAD1	DAD1_HUMAN	12 kDa	0	0	0	0	2	6	0	3	1	0
Coatomer subunit beta'	COPB2_HUMAN	102 kDa	0	0	0	0	0	8	0	3	0	2
Acidic leucine-rich nuclear phosphoprotein 32 family member A	AN32A_HUMAN	29 kDa	0	1	0	0	3	0	4	3	2	1
Protein S100-A1	S10A1_HUMAN	11 kDa	2	0	3	2	0	1	3	3	0	0
60S ribosomal protein L13a	RL13A_HUMAN	24 kDa	0	0	0	0	1	7	0	3	2	2
Rho GDP-dissociation inhibitor 2	GDIR2_HUMAN	23 kDa	0	0	0	0	1	0	4	3	1	7
26S proteasome non-ATPase regulatory subunit 2	PSMD2_HUMAN	100 kDa	1	0	0	0	5	1	0	3	0	6
Heterogeneous nuclear ribonucleoprotein F	HNRPF_HUMAN	46 kDa	0	0	0	0	2	3	0	3	5	3
Exportin-2	XPO2_HUMAN	110 kDa	3	2	0	0	1	1	0	3	5	2
Platelet glycoprotein 4	CD36_HUMAN	53 kDa	0	0	7	2	3	1	0	3	0	1
Acidic leucine-rich nuclear phosphoprotein 32 family member B	AN32B_HUMAN	29 kDa	0	1	1	0	2	3	2	3	5	2
40S ribosomal protein S21	RS21_HUMAN	9 kDa	0	0	0	1	2	6	1	3	3	4
Interleukin enhancer-binding factor 3	ILF3_HUMAN	95 kDa	0	4	1	0	1	6	0	3	5	0
Programmed cell death 6-interacting protein	PDC6I_HUMAN	96 kDa	4	1	0	0	1	0	1	3	5	6
Ig kappa chain V-II region RPMI 6410	KV206_HUMAN	15 kDa	0	0	2	4	1	1	4	3	3	3
60S ribosomal protein L22	RL22_HUMAN	15 kDa	1	2	0	0	2	6	2	3	4	1
Nascent polypeptide-associated complex subunit alpha, muscle-specific form	NACAM_HUMAN	205 kDa	1	1	0	3	0	3	2	3	7	2
Long-chain-fatty-acid--CoA ligase 6	ACSL6_HUMAN	78 kDa	0	7	2	1	9	0	0	3	0	0
Actin-related protein 2	ARP2_HUMAN	45 kDa	3	3	0	1	2	2	0	3	5	4
60S ribosomal protein L12	RL12_HUMAN	18 kDa	1	3	1	1	3	6	0	3	2	3
Chloride intracellular channel protein 4	CLIC4_HUMAN	29 kDa	1	0	4	1	3	4	1	3	2	5
Alpha-aminoadipic semialdehyde dehydrogenase	AL7A1_HUMAN	58 kDa	1	2	0	0	9	2	0	3	5	2
Translocon-associated protein subunit alpha	SSRA_HUMAN	32 kDa	1	0	1	0	4	10	2	3	2	1
Apolipoprotein E	APOE_HUMAN	36 kDa	3	3	0	3	1	0	1	3	10	0
GTP-binding protein SAR1a	SAR1A_HUMAN	22 kDa	3	2	3	0	4	3	0	3	6	0
Proteasome subunit alpha type-3	PSA3_HUMAN	28 kDa	3	1	3	2	3	2	0	3	5	4
Proteasome subunit alpha type-5	PSA5_HUMAN	26 kDa	4	1	2	0	5	3	1	3	6	2
ATP-dependent RNA helicase DDX1	DDX1_HUMAN	82 kDa	3	5	2	0	0	7	0	3	6	2
Elongation factor 1-beta	EF1B_HUMAN	25 kDa	1	4	1	1	3	8	1	3	3	4
Histone H1.5	H15_HUMAN	23 kDa	0	0	0	0	0	1	6	3	16	3
Plastin-2	PLSL_HUMAN	70 kDa	0	0	0	0	2	19	0	3	3	2
Translocon-associated protein subunit delta	SSRD_HUMAN	19 kDa	0	0	0	0	2	23	0	3	1	0

Description	Accession	MW	Raw spectral counts									
			Frontal cortex	Cerebellum	Right ventricle	Mesentric lymph node	Liver	Pancreas	Proximal bile duct	Breast	Ovary	Clitoris
60S ribosomal protein L5	RL5_HUMAN	34 kDa	0	0	0	0	2	22	0	3	2	2
Actin-related protein 2/3 complex subunit 4	ARPC4_HUMAN	20 kDa	4	4	1	0	3	4	3	3	4	6
Heterogeneous nuclear ribonucleoprotein L	HNRPL_HUMAN	64 kDa	1	8	1	0	4	4	0	3	7	6
Galectin-1	LEG1_HUMAN	15 kDa	0	0	1	7	0	3	9	3	7	4
60S ribosomal protein L8	RL8_HUMAN	28 kDa	1	2	1	0	3	17	0	3	4	4
Actin-related protein 2/3 complex subunit 2	ARPC2_HUMAN	34 kDa	6	7	0	1	3	0	3	3	6	7
NADH-cytochrome b5 reductase 3	NB5R3_HUMAN	34 kDa	1	1	3	6	5	1	6	3	5	5
Peroxiredoxin-4	PRDX4_HUMAN	31 kDa	0	0	0	2	0	17	2	3	10	2
Vesicle-associated membrane protein-associated protein A	VAPA_HUMAN	28 kDa	6	4	2	2	4	4	1	3	11	0
Septin-7	SEPT7_HUMAN	51 kDa	13	10	0	0	0	0	2	3	4	6
Integrin beta-1	ITB1_HUMAN	88 kDa	0	0	6	3	3	3	6	3	8	7
NADH dehydrogenase [ubiquinone] flavoprotein 2	NDUV2_HUMAN	27 kDa	5	7	12	1	4	2	2	3	3	0
60S ribosomal protein L3	RL3_HUMAN	46 kDa	0	1	0	0	4	25	0	3	3	4
Lamin-B1	LMNB1_HUMAN	66 kDa	0	4	0	0	3	4	0	3	19	10
Guanine nucleotide-binding protein G(i) subunit alpha-2	GNAI2_HUMAN	40 kDa	17	0	3	5	0	0	4	3	9	3
Unconventional myosin-Ic	MYO1C_HUMAN	122 kDa	0	1	2	7	0	2	3	3	12	19
Pyruvate dehydrogenase E1 component subunit beta	ODPB_HUMAN	39 kDa	12	7	15	2	3	1	0	3	6	3
Alpha-soluble NSF attachment protein	SNAA_HUMAN	33 kDa	6	20	0	0	5	4	3	3	6	6
Carbonic anhydrase 1	CAH1_HUMAN	29 kDa	0	0	7	7	8	0	15	3	4	10
Acetyl-CoA acetyltransferase	THIL_HUMAN	45 kDa	7	4	12	2	10	10	2	3	4	2
GTP:AMP phosphotransferase AK3	KAD3_HUMAN	26 kDa	0	2	8	5	20	9	1	3	8	4
Histone H1.0	H10_HUMAN	21 kDa	2	4	0	2	7	10	7	3	22	6
Calmodulin	CALM_HUMAN	17 kDa	16	15	7	0	8	8	2	3	2	2
2,4-dienoyl-CoA reductase	DECR_HUMAN	36 kDa	3	1	10	4	26	10	4	3	4	2
X-ray repair cross-complementing protein 6	XRCC6_HUMAN	70 kDa	5	10	0	0	7	10	0	3	21	12
Carbonic anhydrase 2	CAH2_HUMAN	29 kDa	7	4	6	3	12	8	21	3	0	7
ATP synthase F(0) complex subunit B1	AT5F1_HUMAN	29 kDa	11	10	26	2	6	6	1	3	4	3
Calponin-1	CNN1_HUMAN	33 kDa	0	0	6	31	0	0	29	3	10	3
Very long-chain specific acyl-CoA dehydrogenase	ACADV_HUMAN	70 kDa	3	7	49	3	18	10	0	3	3	0
Myosin-9	MYH9_HUMAN	227 kDa	10	4	7	19	65	80	18	3	79	120
Sodium/potassium-transporting ATPase subunit alpha-1	AT1A1_HUMAN	113 kDa	153	177	34	0	3	22	3	3	9	22
Glutathione synthetase	GSHB_HUMAN	52 kDa	0	0	0	0	0	0	0	2	0	0
Alpha-galactosidase A	AGAL_HUMAN	49 kDa	0	0	0	0	0	0	0	2	0	0
Regulator of nonsense transcripts 1	RENT1_HUMAN	124 kDa	0	0	0	0	0	0	0	2	0	0
26S proteasome non-ATPase regulatory subunit 3	PSMD3_HUMAN	61 kDa	0	0	0	0	0	0	0	2	0	0
Immunoglobulin J chain	IGJ_HUMAN	18 kDa	0	0	1	0	0	0	0	2	0	0
26S proteasome non-ATPase regulatory subunit 1	PSMD1_HUMAN	106 kDa	0	0	0	0	1	0	0	2	0	0
Acylpyruvase FAHD1	FAHD1_HUMAN	25 kDa	0	0	0	0	1	1	0	2	0	0
Double-stranded RNA-specific adenosine deaminase	DSRAD_HUMAN	136 kDa	0	0	0	0	0	0	0	2	2	0
Keratin, type I cuticular Ha6	KRT36_HUMAN	52 kDa	2	0	0	0	0	0	0	2	0	0
Thioredoxin domain-containing protein 17	TXD17_HUMAN	14 kDa	0	0	0	0	0	0	0	2	1	2
Nuclease-sensitive element-binding protein 1	YBOX1_HUMAN	36 kDa	0	0	0	0	1	0	0	2	2	0
Transcription intermediary factor 1-beta	TIF1B_HUMAN	89 kDa	0	0	0	0	0	0	0	2	3	1
Eukaryotic translation initiation factor 2 subunit 1	IF2A_HUMAN	36 kDa	0	0	0	0	0	2	0	2	1	2
Lysosome-associated membrane glycoprotein 1	LAMP1_HUMAN	45 kDa	0	0	1	0	2	0	0	2	1	1
Toll-interacting protein	TOLIP_HUMAN	30 kDa	3	2	0	0	0	0	0	2	0	0
Adenine phosphoribosyltransferase	APT_HUMAN	20 kDa	0	0	1	0	0	1	0	2	3	0
Myoferlin	MYOF_HUMAN	235 kDa	0	0	0	0	0	0	1	2	2	3
Transthyretin	TTHY_HUMAN	16 kDa	0	0	0	0	1	1	0	2	2	2
Heterogeneous nuclear ribonucleoprotein U-like protein 2	HNRL2_HUMAN	85 kDa	0	5	0	0	0	0	0	2	1	0
Pyridoxine-5'-phosphate oxidase	PNPO_HUMAN	30 kDa	0	3	0	0	3	0	0	2	0	0
Thioredoxin domain-containing protein 5	TXND5_HUMAN	48 kDa	0	0	0	0	0	5	0	2	1	0

Description	Accession	MW	Raw spectral counts									
			Frontal cortex	Cerebellum	Right ventricle	Mesentric lymph node	Liver	Pancreas	Proximal bile duct	Breast	Ovary	Clitoris
Golgi-associated plant pathogenesis-related protein 1	GAPR1_HUMAN	17 kDa	1	0	0	0	0	0	0	2	5	0
Hemopexin	HEMO_HUMAN	52 kDa	0	0	0	4	0	0	1	2	0	2
Coatomer subunit delta	COPD_HUMAN	57 kDa	0	0	0	0	0	6	0	2	1	0
Aspartate--tRNA ligase, cytoplasmic	SYDC_HUMAN	57 kDa	1	0	0	0	0	5	0	2	1	0
Macrophage-capping protein	CAPG_HUMAN	38 kDa	0	0	0	0	0	0	0	2	4	4
Vitamin D-binding protein	VTDB_HUMAN	53 kDa	0	0	0	3	0	0	1	2	1	4
Low molecular weight phosphotyrosine protein phosphatase	PPAC_HUMAN	18 kDa	3	0	0	0	0	2	0	2	3	1
40S ribosomal protein S26	RS26_HUMAN	13 kDa	0	0	0	0	2	6	0	2	1	0
Proteasome subunit beta type-5	PSB5_HUMAN	28 kDa	0	0	0	0	5	1	0	2	2	2
ADP-ribosylation factor 6	ARF6_HUMAN	20 kDa	2	0	0	0	4	2	0	2	1	1
Lysine--tRNA ligase	SYK_HUMAN	68 kDa	0	0	1	0	1	6	0	2	2	0
Signal peptidase complex catalytic subunit SEC11C	SC11C_HUMAN	22 kDa	0	0	0	0	1	9	0	2	0	0
26S proteasome non-ATPase regulatory subunit 11	PSD11_HUMAN	47 kDa	0	0	0	0	0	2	0	2	4	5
DNA-(apurinic or apyrimidinic site) lyase	APEX1_HUMAN	36 kDa	0	0	1	0	0	3	0	2	5	2
ELAV-like protein 1	ELAV1_HUMAN	36 kDa	2	3	0	0	0	0	0	2	6	0
3-hydroxyisobutyryl-CoA hydrolase	HIBCH_HUMAN	43 kDa	0	1	4	1	1	2	0	2	2	0
Protein ERGIC-53	LMAN1_HUMAN	58 kDa	0	0	0	0	3	7	0	2	1	0
Protein canopy homolog 2	CNPY2_HUMAN	21 kDa	1	0	0	0	3	5	0	2	2	0
Receptor expression-enhancing protein 5	REEP5_HUMAN	21 kDa	2	1	4	0	0	4	0	2	0	1
Serpin B5	SPB5_HUMAN	42 kDa	0	0	0	0	0	0	0	2	0	13
Histidine-rich glycoprotein	HRG_HUMAN	60 kDa	0	0	2	2	0	0	2	2	3	4
Platelet-activating factor acetylhydrolase IB subunit beta	PA1B2_HUMAN	26 kDa	3	2	0	0	0	2	2	2	2	2
Ornithine aminotransferase	OAT_HUMAN	49 kDa	0	1	0	0	10	0	2	2	0	0
Cytochrome b-c1 complex subunit 9	QCR9_HUMAN	7 kDa	4	0	5	0	2	2	0	2	0	0
Plasma protease C1 inhibitor	IC1_HUMAN	55 kDa	0	0	1	1	1	0	4	2	3	4
tRNA-splicing ligase RtcB homolog	RTCB_HUMAN	55 kDa	4	3	0	0	0	3	0	2	4	0
Mitochondrial fission 1 protein	FIS1_HUMAN	17 kDa	3	1	5	0	3	2	0	2	0	0
Annexin A11	ANX11_HUMAN	54 kDa	3	0	7	0	0	1	0	2	2	3
6-phosphogluconolactonase	6PGL_HUMAN	28 kDa	2	0	1	0	2	2	1	2	5	3
Sulfide:quinone oxidoreductase	SQRD_HUMAN	50 kDa	0	0	3	1	6	0	4	2	0	2
40S ribosomal protein S28	RS28_HUMAN	8 kDa	0	0	0	0	3	8	0	2	3	2
CDGSH iron-sulfur domain-containing protein 1	CISD1_HUMAN	12 kDa	5	4	3	0	2	0	1	2	1	0
AFG3-like protein 2	AFG32_HUMAN	89 kDa	4	3	9	0	0	0	0	2	1	0
Rho GTPase-activating protein 1	RHG01_HUMAN	50 kDa	3	0	0	1	0	2	2	2	4	6
Translin	TSN_HUMAN	26 kDa	4	3	0	0	1	1	0	2	5	4
Calpain small subunit 1	CPNS1_HUMAN	28 kDa	2	2	3	0	3	2	1	2	3	2
Heat shock 70 kDa protein 4	HSP74_HUMAN	94 kDa	5	2	0	0	0	0	0	2	9	2
Translationally-controlled tumor protein	TCTP_HUMAN	20 kDa	1	1	2	1	3	6	0	2	3	1
Proteasome subunit alpha type-2	PSA2_HUMAN	26 kDa	4	1	1	0	5	2	0	2	4	1
Fumarylacetoacetate hydrolase domain-containing protein 2A	FAH2A_HUMAN	35 kDa	4	1	3	0	2	3	0	2	5	0
Serine/arginine-rich splicing factor 3	SRSF3_HUMAN	19 kDa	0	4	0	0	2	2	0	2	7	4
Prenylcysteine oxidase 1	PCYOX_HUMAN	57 kDa	2	0	2	4	3	2	2	2	2	2
Keratin, type II cytoskeletal 4	K2C4_HUMAN	57 kDa	1	0	6	0	1	11	0	2	0	0
Proteasome activator complex subunit 2	PSME2_HUMAN	27 kDa	0	0	1	0	7	5	1	2	4	2
Dynamin-2	DYN2_HUMAN	98 kDa	10	8	0	0	0	0	1	2	0	1
Cleavage and polyadenylation specificity factor subunit 5	CPSF5_HUMAN	26 kDa	3	6	0	0	2	1	0	2	7	1
Nuclear mitotic apparatus protein 1	NUMA1_HUMAN	238 kDa	0	2	0	0	1	3	0	2	9	6
S-formylglutathione hydrolase	ESTD_HUMAN	31 kDa	2	0	1	0	6	5	0	2	4	3
Ras-related protein Rab-14	RAB14_HUMAN	24 kDa	8	5	0	2	2	1	1	2	1	1
Ubiquitin thioesterase OTUB1	OTUB1_HUMAN	31 kDa	8	3	1	0	0	1	1	2	6	1
Serum deprivation-response protein	SDPR_HUMAN	47 kDa	0	0	7	8	0	1	2	2	2	1
Heterogeneous nuclear ribonucleoprotein H3	HNRH3_HUMAN	37 kDa	2	3	0	0	0	1	0	2	15	0

Description	Accession	MW	Raw spectral counts									
			Frontal cortex	Cerebellum	Right ventricle	Mesentric lymph node	Liver	Pancreas	Proximal bile duct	Breast	Ovary	Clitoris
Bifunctional glutamate/proline--tRNA ligase	SYEP_HUMAN	171 kDa	0	0	1	0	0	20	0	2	0	0
Proteasome subunit beta type-3	PSB3_HUMAN	23 kDa	3	3	2	0	4	4	0	2	2	4
cAMP-dependent protein kinase type II-alpha regulatory subunit	KAP2_HUMAN	46 kDa	4	1	5	0	2	0	1	2	6	3
T-complex protein 1 subunit epsilon	TCPE_HUMAN	60 kDa	6	6	1	1	2	0	1	2	3	2
60S ribosomal protein L26	RL26_HUMAN	17 kDa	0	0	0	0	4	10	0	2	5	4
Serine/threonine-protein phosphatase PP1-beta catalytic subunit	PP1B_HUMAN	37 kDa	4	2	4	0	2	6	0	2	2	3
Lon protease homolog	LONM_HUMAN	106 kDa	2	2	8	0	4	1	0	2	6	0
GTP-binding nuclear protein Ran	RAN_HUMAN	24 kDa	2	7	0	0	2	1	1	2	6	5
Enoyl-CoA delta isomerase 1	ECI1_HUMAN	33 kDa	2	2	3	2	5	4	1	2	3	2
Junction plakoglobin	PLAK_HUMAN	82 kDa	5	0	3	0	2	5	0	2	0	10
Actin-related protein 3	ARP3_HUMAN	47 kDa	3	4	0	3	1	4	2	2	4	4
Quinone oxidoreductase	QOR_HUMAN	35 kDa	3	2	3	0	4	7	3	2	3	0
Complement C4-A	CO4A_HUMAN	193 kDa	0	0	3	2	3	1	2	2	7	8
Inorganic pyrophosphatase	IPYR_HUMAN	33 kDa	5	2	1	0	3	13	0	2	2	0
Proteasome subunit beta type-1	PSB1_HUMAN	26 kDa	4	0	4	0	8	5	0	2	4	3
Heterogeneous nuclear ribonucleoprotein R	HNRPR_HUMAN	71 kDa	2	11	0	0	0	3	0	2	12	0
Histone H1x	H1X_HUMAN	22 kDa	0	3	0	0	0	2	1	2	22	0
Serine/arginine-rich splicing factor 1	SRSF1_HUMAN	28 kDa	0	4	1	0	2	3	1	2	15	4
Omega-amidase NIT2	NIT2_HUMAN	31 kDa	2	1	5	0	9	2	2	2	5	4
Ras-related protein Rab-5A	RAB5A_HUMAN	24 kDa	6	7	3	2	0	1	1	2	7	3
Ceruloplasmin	CERU_HUMAN	122 kDa	0	0	6	2	2	0	8	2	4	9
ATP-dependent RNA helicase A	DHX9_HUMAN	141 kDa	4	4	0	0	5	6	0	2	9	3
Cytoplasmic aconitate hydratase	ACOC_HUMAN	98 kDa	3	1	0	1	20	3	0	2	4	1
Eukaryotic translation initiation factor 3 subunit A	EIF3A_HUMAN	167 kDa	2	0	4	0	3	16	0	2	8	0
Thy-1 membrane glycoprotein	THY1_HUMAN	18 kDa	10	6	0	4	0	1	5	2	3	6
Glutathione S-transferase omega-1	GSTO1_HUMAN	28 kDa	3	3	1	1	12	1	3	2	5	6
Cytosol aminopeptidase	AMPL_HUMAN	56 kDa	6	2	7	0	13	0	1	2	3	3
Fructose-1,6-bisphosphatase 1	F16P1_HUMAN	37 kDa	0	0	0	0	34	3	0	2	0	0
Dihydropteridine reductase	DHPR_HUMAN	26 kDa	8	9	3	1	9	1	1	2	5	1
Cytochrome b-c1 complex subunit Rieske	UCRI_HUMAN	30 kDa	7	3	18	2	3	6	1	2	0	0
Membrane primary amine oxidase	AOC3_HUMAN	85 kDa	0	0	2	25	0	0	10	2	1	4
Succinyl-CoA ligase [GDP-forming] subunit beta	SUCB2_HUMAN	47 kDa	0	0	12	1	13	7	3	2	4	2
Protein DJ-1	PARK7_HUMAN	20 kDa	4	8	2	3	13	5	0	2	6	1
3-hydroxyisobutyrate dehydrogenase	3HIDH_HUMAN	35 kDa	1	1	11	1	15	8	0	2	7	0
Dihydropyrimidinase-related protein 3	DPYL3_HUMAN	62 kDa	20	0	0	1	0	0	4	2	17	5
Heat shock protein 75 kDa	TRAP1_HUMAN	80 kDa	4	9	4	5	4	6	4	2	8	3
Collagen alpha-1(VI) chain	CO6A1_HUMAN	109 kDa	0	0	4	7	0	1	4	2	5	28
Argininosuccinate synthase	ASSY_HUMAN	47 kDa	0	1	0	0	47	0	0	2	0	1
Reticulon-3	RTN3_HUMAN	113 kDa	17	5	1	3	4	7	5	2	2	6
Glycerol-3-phosphate dehydrogenase [NAD(+)], cytoplasmic	GPDA_HUMAN	38 kDa	0	0	2	32	11	0	4	2	0	2
ATP synthase subunit d	ATP5H_HUMAN	18 kDa	8	10	23	0	10	5	0	2	4	0
Glutathione S-transferase Mu 3	GSTM3_HUMAN	27 kDa	6	22	8	0	0	0	3	2	36	2
Vesicle-fusing ATPase	NSF_HUMAN	83 kDa	28	50	0	0	0	13	0	2	3	0
Glycogen phosphorylase, muscle form	PYGM_HUMAN	97 kDa	12	35	46	3	7	1	6	2	0	1
Aldehyde dehydrogenase	ALDH2_HUMAN	56 kDa	9	9	14	26	40	13	30	2	23	14
Dihydropyrimidinase-related protein 2	DPYL2_HUMAN	62 kDa	82	108	3	1	0	2	1	2	5	9
Myosin-11	MYH11_HUMAN	227 kDa	5	2	5	57	0	18	143	2	96	83
Proteasome activator complex subunit 3	PSME3_HUMAN	30 kDa	0	0	0	0	0	0	0	1	0	0
Zinc finger protein 40	ZEP1_HUMAN	297 kDa	0	0	0	0	0	0	0	1	0	0
N-alpha-acetyltransferase 35, NatC auxiliary subunit	NAA35_HUMAN	84 kDa	0	0	0	0	0	0	0	1	0	0
Serrate RNA effector molecule homolog	SRRT_HUMAN	101 kDa	0	0	0	0	0	0	0	1	0	0
Dedicator of cytokinesis protein 2	DOCK2_HUMAN	212 kDa	0	0	0	0	0	0	0	1	0	0

Description	Accession	MW	Raw spectral counts									
			Frontal cortex	Cerebellum	Right ventricle	Mesentric lymph node	Liver	Pancreas	Proximal bile duct	Breast	Ovary	Clitoris
Lactase-phlorizin hydrolase	LPH_HUMAN	219 kDa	0	0	0	0	0	0	0	1	0	0
Tyrosine-protein phosphatase non-receptor type 14	PTN14_HUMAN	135 kDa	0	0	0	0	0	0	0	1	0	0
Tubulin polyglutamylase TTLL13	TTL13_HUMAN	94 kDa	0	0	0	0	0	0	0	1	0	0
CCR4-NOT transcription complex subunit 3	CNOT3_HUMAN	82 kDa	0	0	0	0	0	0	0	1	0	0
BET1-like protein	BET1L_HUMAN	12 kDa	0	0	0	0	0	0	0	1	0	0
Xylosyltransferase 2	XYLT2_HUMAN	97 kDa	0	0	0	0	0	0	0	1	0	0
Neutral alpha-glucosidase C	GANC_HUMAN	104 kDa	0	0	0	0	0	0	0	1	0	0
Adenylosuccinate synthetase isozyme 2	PURA2_HUMAN	50 kDa	0	0	0	0	0	0	0	1	0	0
Tumor protein p53-inducible protein 11	P5I11_HUMAN	21 kDa	0	0	0	0	0	0	0	1	0	0
Kappa-casein	CASK_HUMAN	20 kDa	0	0	0	0	0	0	0	1	0	0
Ragulator complex protein LAMTOR3	LTOR3_HUMAN	14 kDa	0	0	0	0	0	0	0	1	0	0
Marginal zone B- and B1-cell-specific protein	MZB1_HUMAN	21 kDa	0	0	0	0	0	0	0	1	0	0
Mitogen-activated protein kinase kinase kinase 13	M3K13_HUMAN	108 kDa	0	0	0	0	0	0	0	1	0	0
Replication protein A 32 kDa subunit	RFA2_HUMAN	29 kDa	0	0	0	0	0	0	0	1	0	0
WD repeat-containing protein 81	WDR81_HUMAN	212 kDa	0	0	0	0	0	0	0	1	0	0
ATP-binding cassette sub-family C member 9	ABCC9_HUMAN	174 kDa	0	0	0	0	0	0	0	1	0	0
Dedicator of cytokinesis protein 1	DOCK1_HUMAN	215 kDa	0	0	0	0	0	0	0	1	0	0
Synaptogyrin-2	SNG2_HUMAN	25 kDa	0	0	0	0	0	0	0	1	0	0
60S ribosomal protein L37a	RL37A_HUMAN	10 kDa	0	0	0	0	0	0	0	1	0	0
Mannose-P-dolichol utilization defect 1 protein	MPU1_HUMAN	27 kDa	0	0	0	0	0	0	0	1	0	0
Transmembrane 9 superfamily member 4	TM9S4_HUMAN	75 kDa	0	0	0	0	0	0	0	1	0	0
Transmembrane 9 superfamily member 2	TM9S2_HUMAN	76 kDa	0	0	0	0	0	0	0	1	0	0
Signal transducer and activator of transcription 3	STAT3_HUMAN	88 kDa	0	0	0	0	0	0	0	1	0	0
Actin-related protein 2/3 complex subunit 1B	ARC1B_HUMAN	41 kDa	0	0	0	0	0	0	0	1	0	0
Cellular retinoic acid-binding protein 2	RABP2_HUMAN	16 kDa	0	0	0	0	0	0	0	1	0	0
Clustered mitochondria protein homolog	CLU_HUMAN	147 kDa	0	0	0	0	0	0	0	1	0	0
Catenin delta-1	CTND1_HUMAN	108 kDa	0	0	0	0	0	0	0	1	0	0
Lysosomal Pro-X carboxypeptidase	PCP_HUMAN	56 kDa	0	0	0	0	0	0	0	1	0	0
N-sulphoglucosamine sulphohydrolase	SPHM_HUMAN	57 kDa	0	0	0	0	0	0	0	1	0	0
Alkaline phosphatase, tissue-nonspecific isozyme	PPBT_HUMAN	57 kDa	0	0	0	0	0	0	0	1	0	0
Mth938 domain-containing protein	AAMDC_HUMAN	13 kDa	0	0	0	0	0	0	0	1	0	0
Alpha-1-antichymotrypsin	AACT_HUMAN	48 kDa	0	0	1	0	0	0	0	1	0	0
AP-1 complex subunit gamma-1	AP1G1_HUMAN	91 kDa	0	0	0	0	0	1	0	1	0	0
Pyrroline-5-carboxylate reductase 1	P5CR1_HUMAN	33 kDa	0	0	0	0	0	1	0	1	0	0
Pyridoxal-dependent decarboxylase domain-containing protein 1	PDXD1_HUMAN	87 kDa	0	0	0	0	0	1	0	1	0	0
Lipopolysaccharide-responsive and beige-like anchor protein	LRBA_HUMAN	319 kDa	0	0	0	0	0	0	1	1	0	0
Probable tRNA N6-adenosine threonylcarbamoyltransferase	OSGEP_HUMAN	36 kDa	0	0	0	0	0	0	0	1	1	0
Cytosolic non-specific dipeptidase	CNDP2_HUMAN	53 kDa	0	0	0	0	0	0	0	1	1	0
Protein S100-A16	S10AG_HUMAN	12 kDa	0	0	0	0	0	0	0	1	0	2
Deoxynucleoside triphosphate triphosphohydrolase SAMHD1	SAMH1_HUMAN	72 kDa	0	0	0	0	0	0	0	1	0	2
Eukaryotic translation initiation factor 6	IF6_HUMAN	27 kDa	0	0	0	0	1	0	0	1	0	1
Adenylosuccinate lyase	PUR8_HUMAN	55 kDa	0	0	1	0	0	0	0	1	1	0
Epithelial cell adhesion molecule	EPCAM_HUMAN	35 kDa	0	0	0	0	0	2	0	1	0	0
Methionine--tRNA ligase, cytoplasmic	SYMC_HUMAN	101 kDa	0	0	0	0	0	1	0	1	1	0
60S ribosomal protein L35a	RL35A_HUMAN	13 kDa	0	0	0	0	1	1	0	1	0	0
Coatomer subunit epsilon	COPE_HUMAN	34 kDa	0	0	0	0	1	1	0	1	0	0
Pyrroline-5-carboxylate reductase 3	P5CR3_HUMAN	29 kDa	0	0	0	0	0	0	0	1	2	0
Peptidyl-prolyl cis-trans isomerase H	PPIH_HUMAN	19 kDa	0	0	0	0	0	0	0	1	2	0
26S proteasome non-ATPase regulatory subunit 12	PSD12_HUMAN	53 kDa	0	0	0	0	0	1	0	1	1	1
COP9 signalosome complex subunit 3	CSN3_HUMAN	48 kDa	0	0	0	0	0	1	0	1	1	1
Lysosomal protective protein	PPGB_HUMAN	54 kDa	0	0	0	0	1	0	0	1	1	1
40S ribosomal protein S27	RS27_HUMAN	9 kDa	0	0	0	0	0	3	0	1	0	0

Description	Accession	MW	Raw spectral counts									
			Frontal cortex	Cerebellum	Right ventricle	Mesentric lymph node	Liver	Pancreas	Proximal bile duct	Breast	Ovary	Clitoris
Eukaryotic translation initiation factor 2 subunit 3	IF2G_HUMAN	51 kDa	0	0	0	0	0	3	0	1	0	0
Eukaryotic translation initiation factor 2 subunit 2	IF2B_HUMAN	38 kDa	0	0	0	0	0	2	0	1	1	0
GDP-L-fucose synthase	FCL_HUMAN	36 kDa	0	0	0	0	0	2	0	1	1	0
Glutamine--tRNA ligase	SYQ_HUMAN	88 kDa	0	0	0	0	1	2	0	1	0	0
Glia-derived nexin	GDN_HUMAN	44 kDa	0	0	0	0	0	1	0	1	2	0
TP53-regulating kinase	PRPK_HUMAN	28 kDa	0	0	0	0	0	0	0	1	3	0
Transportin-1	TNPO1_HUMAN	102 kDa	0	0	0	0	0	0	0	1	3	0
Beta-centractin	ACTY_HUMAN	42 kDa	3	0	0	0	0	0	0	1	0	0
Methylcrotonoyl-CoA carboxylase subunit alpha	MCCA_HUMAN	80 kDa	0	0	2	0	0	1	0	1	0	1
60S ribosomal protein L28	RL28_HUMAN	16 kDa	0	0	0	0	2	1	0	1	0	1
Structural maintenance of chromosomes protein 3	SMC3_HUMAN	142 kDa	0	0	0	0	0	0	0	1	3	1
Mitochondrial carrier homolog 1	MTCH1_HUMAN	42 kDa	0	2	0	0	0	2	0	1	0	0
Titin	TITIN_HUMAN	3816 kDa	1	0	1	0	1	0	1	1	0	0
Copine-1	CPNE1_HUMAN	59 kDa	0	0	0	0	2	2	0	1	0	0
Glucosamine 6-phosphate N-acetyltransferase	GNA1_HUMAN	21 kDa	0	0	0	0	2	2	0	1	0	0
Structural maintenance of chromosomes protein 1A	SMC1A_HUMAN	143 kDa	0	0	0	0	1	0	0	1	3	0
Clusterin	CLUS_HUMAN	52 kDa	1	0	0	0	0	0	0	1	3	0
Putative pre-mRNA-splicing factor ATP-dependent RNA helicase DHX15	DHX15_HUMAN	91 kDa	0	2	0	0	0	0	0	1	1	2
Galectin-3	LEG3_HUMAN	26 kDa	0	0	0	0	0	0	0	1	3	2
Bleomycin hydrolase	BLMH_HUMAN	53 kDa	0	0	0	1	2	0	1	1	0	1
NADH-ubiquinone oxidoreductase chain 4	NU4M_HUMAN	52 kDa	0	0	4	0	0	1	0	1	0	0
Phosphomannomutase 2	PMM2_HUMAN	28 kDa	0	0	0	0	1	4	0	1	0	0
Endoplasmic reticulum resident protein 44	ERP44_HUMAN	47 kDa	0	0	0	0	1	3	0	1	1	0
Mannosyl-oligosaccharide glucosidase	MOGS_HUMAN	92 kDa	0	0	0	0	2	3	0	1	0	0
U5 small nuclear ribonucleoprotein 200 kDa helicase	U520_HUMAN	245 kDa	0	0	0	0	1	1	0	1	3	0
Periostin	POSTN_HUMAN	93 kDa	0	0	0	0	0	0	0	1	0	6
Protein NipSnap homolog 3A	NPS3A_HUMAN	28 kDa	0	0	2	0	2	0	0	1	0	2
3-mercaptopyruvate sulfurtransferase	THTM_HUMAN	33 kDa	0	0	0	0	2	2	0	1	1	1
Protein Niban	NIBAN_HUMAN	103 kDa	0	0	6	0	0	0	0	1	0	0
60S ribosomal protein L18a	RL18A_HUMAN	21 kDa	0	0	0	0	0	6	0	1	0	0
Dipeptidyl peptidase 3	DPP3_HUMAN	83 kDa	0	0	0	0	0	6	0	1	0	0
Phenylalanine--tRNA ligase alpha subunit	SYFA_HUMAN	58 kDa	2	0	0	0	0	4	0	1	0	0
Alpha/beta hydrolase domain-containing protein 14B	ABHEB_HUMAN	22 kDa	0	0	0	0	2	2	0	1	2	0
Integrin alpha-6	ITA6_HUMAN	127 kDa	0	0	0	0	0	1	1	1	0	5
Annexin A7	ANXA7_HUMAN	53 kDa	0	1	1	0	0	0	1	1	1	3
ADP-ribosylation factor-like protein 8B	ARL8B_HUMAN	22 kDa	2	1	0	0	1	1	0	1	0	2
Lysosome membrane protein 2	SCRB2_HUMAN	54 kDa	0	0	1	0	1	0	0	1	3	2
Early endosome antigen 1	EEA1_HUMAN	162 kDa	2	0	0	0	0	3	0	1	0	2
Up-regulated during skeletal muscle growth protein 5	USMG5_HUMAN	6 kDa	1	0	3	1	1	0	0	1	0	1
60S ribosomal protein L36	RL36_HUMAN	12 kDa	0	0	0	0	1	3	0	1	2	1
Myotrophin	MTPN_HUMAN	13 kDa	1	0	0	0	1	1	1	1	2	1
Nucleoside diphosphate kinase 3	NDK3_HUMAN	19 kDa	0	1	0	0	3	2	0	1	1	0
Mesencephalic astrocyte-derived neurotrophic factor	MANF_HUMAN	21 kDa	0	0	0	0	2	4	0	1	1	0
26S protease regulatory subunit 4	PRS4_HUMAN	49 kDa	2	0	0	0	0	0	0	1	5	0
Coagulation factor XIII A chain	F13A_HUMAN	83 kDa	0	0	0	0	0	0	0	1	0	8
Ribonuclease inhibitor	RINI_HUMAN	50 kDa	0	0	0	0	1	0	0	1	4	3
RNA-binding protein Raly	RALY_HUMAN	32 kDa	0	1	0	0	0	2	0	1	3	2
Eukaryotic translation initiation factor 3 subunit I	EIF3I_HUMAN	37 kDa	0	0	0	0	0	2	1	1	3	2
60S ribosomal protein L21	RL21_HUMAN	19 kDa	0	1	0	0	1	5	0	1	0	1
Eukaryotic translation initiation factor 3 subunit H	EIF3H_HUMAN	40 kDa	0	0	0	0	0	5	0	1	2	1
40S ribosomal protein S15a	RS15A_HUMAN	15 kDa	0	0	0	0	2	3	0	1	2	1
Delta-1-pyrroline-5-carboxylate synthase	P5CS_HUMAN	87 kDa	0	0	1	0	0	7	0	1	0	0

Description	Accession	MW	Raw spectral counts									
			Frontal cortex	Cerebellum	Right ventricle	Mesentric lymph node	Liver	Pancreas	Proximal bile duct	Breast	Ovary	Clitoris
Actin-related protein 2/3 complex subunit 5	ARPC5_HUMAN	16 kDa	1	0	1	0	0	2	1	1	3	0
Phenylalanine--tRNA ligase beta subunit	SYFB_HUMAN	66 kDa	2	0	0	0	0	6	0	1	0	0
Bifunctional purine biosynthesis protein PURH	PUR9_HUMAN	65 kDa	3	0	0	0	1	2	0	1	2	0
Acyl-CoA-binding protein	ACBP_HUMAN	10 kDa	2	0	0	1	2	1	2	1	0	0
Protein S100-A10	S10AA_HUMAN	11 kDa	0	0	0	0	0	0	1	1	1	7
Small nuclear ribonucleoprotein-associated proteins B and B'	RSMB_HUMAN	25 kDa	0	2	0	0	0	0	0	1	6	1
Platelet-activating factor acetylhydrolase IB subunit gamma	PA1B3_HUMAN	26 kDa	3	2	0	0	0	0	0	1	3	1
Glyoxalase domain-containing protein 4	GLOD4_HUMAN	35 kDa	0	0	0	0	0	2	1	1	5	1
26S protease regulatory subunit 10B	PRS10_HUMAN	44 kDa	1	0	0	0	1	1	0	1	5	1
Proteasome subunit beta type-6	PSB6_HUMAN	25 kDa	2	2	0	0	2	2	0	1	1	0
Pyruvate dehydrogenase protein X component	ODPX_HUMAN	54 kDa	2	0	7	0	0	0	0	1	0	0
Serine--tRNA ligase, cytoplasmic	SYSC_HUMAN	59 kDa	1	0	0	0	0	6	0	1	2	0
Very-long-chain enoyl-CoA reductase	TECR_HUMAN	36 kDa	0	0	0	4	2	2	0	1	1	0
Galectin-7	LEG7_HUMAN	15 kDa	0	0	0	0	0	0	0	1	0	10
26S proteasome non-ATPase regulatory subunit 13	PSD13_HUMAN	43 kDa	2	1	0	0	1	4	0	1	0	2
60S acidic ribosomal protein P1	RLA1_HUMAN	12 kDa	1	1	0	1	0	3	0	1	2	2
26S proteasome non-ATPase regulatory subunit 5	PSMD5_HUMAN	56 kDa	1	0	0	0	0	2	0	1	5	2
26S proteasome non-ATPase regulatory subunit 8	PSMD8_HUMAN	40 kDa	1	1	1	0	1	3	0	1	3	0
Glycerol-3-phosphate dehydrogenase 1-like protein	GPD1L_HUMAN	38 kDa	0	0	7	0	0	2	0	1	1	0
Small nuclear ribonucleoprotein Sm D2	SMD2_HUMAN	14 kDa	0	2	0	0	1	2	0	1	4	2
26S protease regulatory subunit 6A	PRS6A_HUMAN	49 kDa	1	0	0	0	3	1	0	1	5	1
Small nuclear ribonucleoprotein Sm D1	SMD1_HUMAN	13 kDa	1	3	0	0	0	4	0	1	2	2
Peptidyl-prolyl cis-trans isomerase FKBP4	FKBP4_HUMAN	52 kDa	0	8	0	0	0	0	0	1	4	0
N(G),N(G)-dimethylarginine dimethylaminohydrolase 2	DDAH2_HUMAN	30 kDa	0	0	0	0	0	0	2	1	7	4
60S ribosomal protein L4	RL4_HUMAN	48 kDa	0	0	0	0	1	8	0	1	2	2
Protein phosphatase 1 regulatory subunit 7	PP1R7_HUMAN	42 kDa	3	1	3	0	2	1	0	1	2	1
DNA-dependent protein kinase catalytic subunit	PRKDC_HUMAN	469 kDa	0	0	0	0	0	3	0	1	9	1
Ubiquitin-conjugating enzyme E2 variant 1	UB2V1_HUMAN	16 kDa	4	2	1	0	1	3	0	1	2	0
Tricarboxylate transport protein	TXTP_HUMAN	34 kDa	0	0	0	4	5	1	0	1	3	0
Alcohol dehydrogenase class-3	ADHX_HUMAN	40 kDa	0	2	1	0	7	1	0	1	2	1
Exportin-1	XPO1_HUMAN	123 kDa	6	2	0	0	0	0	0	1	5	1
ATP-dependent RNA helicase DDX3X	DDX3X_HUMAN	73 kDa	0	4	0	0	0	6	0	1	4	0
NADH dehydrogenase [ubiquinone] 1 beta subcomplex subunit 10	NDUBA_HUMAN	21 kDa	2	2	4	1	2	2	0	1	1	0
Malectin	MLEC_HUMAN	32 kDa	0	0	0	0	3	8	0	1	3	0
UDP-glucose 6-dehydrogenase	UGDH_HUMAN	55 kDa	0	0	0	0	11	1	0	1	2	0
Sideroflexin-3	SFXN3_HUMAN	36 kDa	3	5	0	0	0	0	1	1	1	5
Transmembrane protein 43	TMM43_HUMAN	45 kDa	0	0	0	3	0	1	2	1	6	3
Aflatoxin B1 aldehyde reductase member 2	ARK72_HUMAN	40 kDa	1	1	0	2	2	2	3	1	2	2
Fumarylacetoacetase	FAAA_HUMAN	46 kDa	0	0	0	1	13	0	1	1	0	0
Thioredoxin	THIO_HUMAN	12 kDa	1	0	0	0	3	2	3	1	3	4
High mobility group protein B2	HMGB2_HUMAN	24 kDa	0	2	1	0	0	4	1	1	7	1
Thioredoxin-like protein 1	TXNL1_HUMAN	32 kDa	1	0	1	0	3	4	0	1	6	1
Adenylyl cyclase-associated protein 1	CAP1_HUMAN	52 kDa	5	2	0	0	0	0	0	1	6	4
Four and a half LIM domains protein 1	FHL1_HUMAN	36 kDa	0	1	3	2	0	1	3	1	4	3
60S ribosomal protein L24	RL24_HUMAN	18 kDa	0	1	0	0	2	6	0	1	5	3
WD repeat-containing protein 1	WDR1_HUMAN	66 kDa	1	0	5	2	0	3	1	1	2	3
60S ribosomal protein L15	RL15_HUMAN	24 kDa	0	2	0	0	2	10	0	1	2	1
Thiosulfate sulfurtransferase	THTR_HUMAN	33 kDa	0	1	2	1	8	2	1	1	1	1
Cytochrome c oxidase subunit 4 isoform 1	COX41_HUMAN	20 kDa	2	5	3	1	3	1	0	1	2	0
S-phase kinase-associated protein 1	SKP1_HUMAN	19 kDa	4	1	1	0	2	3	0	1	6	0
Non-specific lipid-transfer protein	NLTP_HUMAN	59 kDa	0	0	0	1	14	1	0	1	1	1
60S ribosomal protein L17	RL17_HUMAN	21 kDa	0	1	0	0	3	12	0	1	2	0

Description	Accession	MW	Raw spectral counts									
			Frontal cortex	Cerebellum	Right ventricle	Mesentric lymph node	Liver	Pancreas	Proximal bile duct	Breast	Ovary	Clitoris
Serine/arginine-rich splicing factor 7	SRSF7_HUMAN	27 kDa	0	4	1	0	1	3	0	1	8	2
Ubiquitin-conjugating enzyme E2 N	UBE2N_HUMAN	17 kDa	4	3	2	0	3	3	1	1	2	1
Prostaglandin E synthase 2	PGES2_HUMAN	42 kDa	4	2	5	1	3	2	0	1	2	0
General vesicular transport factor p115	USO1_HUMAN	108 kDa	2	1	0	0	0	15	0	1	1	0
Dehydrogenase/reductase SDR family member 7	DHRS7_HUMAN	38 kDa	0	0	1	0	5	12	1	1	0	0
Prostaglandin reductase 1	PTGR1_HUMAN	36 kDa	0	0	0	0	19	0	0	1	0	0
Ubiquitin-conjugating enzyme E2 L3	UB2L3_HUMAN	18 kDa	2	2	2	0	3	5	0	1	3	3
Fascin	FSCN1_HUMAN	55 kDa	13	2	0	0	0	0	0	1	2	3
Ras-related protein R-Ras	RRAS_HUMAN	23 kDa	0	0	3	7	0	0	4	1	4	2
Coiled-coil-helix-coiled-coil-helix domain-containing protein 3	CHCH3_HUMAN	26 kDa	5	3	7	0	0	2	0	1	2	1
Aldose reductase	ALDR_HUMAN	36 kDa	2	2	6	2	0	0	3	1	4	2
Caldesmon	CALD1_HUMAN	93 kDa	0	0	0	3	0	0	9	1	7	2
Annexin A3	ANXA3_HUMAN	36 kDa	0	0	3	1	0	5	5	1	6	1
F-actin-capping protein subunit beta	CAPZB_HUMAN	31 kDa	2	5	0	1	1	3	1	1	5	4
Haptoglobin	HPT_HUMAN	45 kDa	0	0	1	6	1	0	8	1	3	3
Non-POU domain-containing octamer-binding protein	NONO_HUMAN	54 kDa	1	10	0	0	2	3	0	1	4	2
60S ribosomal protein L23	RL23_HUMAN	15 kDa	3	3	0	0	2	10	0	1	3	2
Transcriptional activator protein Pur-alpha	PURA_HUMAN	35 kDa	6	4	3	0	1	4	0	1	4	1
Sulfotransferase 1A1	ST1A1_HUMAN	34 kDa	0	0	0	1	14	0	0	1	6	3
60S ribosomal protein L23a	RL23A_HUMAN	18 kDa	1	1	0	0	2	12	0	1	7	1
Succinyl-CoA ligase [ADP/GDP-forming] subunit alpha	SUCA_HUMAN	36 kDa	2	3	7	2	6	2	1	1	1	0
NADPH--cytochrome P450 reductase	NCPR_HUMAN	77 kDa	0	0	0	2	18	0	0	1	4	0
Sepiapterin reductase	SPRE_HUMAN	28 kDa	2	0	2	0	10	5	0	1	6	0
Lactoylglutathione lyase	LGUL_HUMAN	21 kDa	6	1	2	0	6	2	1	1	6	2
Dihydrolipoyllysine-residue succinyltransferase component of 2-oxoglutarate	ODO2_HUMAN	49 kDa	6	2	9	1	4	1	1	1	1	1
UDP-glucose:glycoprotein glucosyltransferase 1	UGGG1_HUMAN	177 kDa	0	0	0	0	4	21	0	1	1	0
Extracellular superoxide dismutase [Cu-Zn]	SODE_HUMAN	26 kDa	0	0	2	4	0	0	8	1	4	9
B-cell receptor-associated protein 31	BAP31_HUMAN	28 kDa	1	2	2	4	4	5	2	1	1	6
Perilipin-1	PLIN1_HUMAN	56 kDa	0	0	0	24	0	0	2	1	0	1
Ras suppressor protein 1	RSU1_HUMAN	32 kDa	0	0	3	5	1	3	8	1	3	6
NADH dehydrogenase [ubiquinone] 1 alpha subcomplex subunit 10	NDUAA_HUMAN	41 kDa	3	5	12	0	1	6	1	1	1	0
Leucine-rich PPR motif-containing protein	LPPRC_HUMAN	158 kDa	10	1	13	0	0	1	1	1	3	0
Ras-related C3 botulinum toxin substrate 1	RAC1_HUMAN	21 kDa	5	7	2	0	4	3	0	1	6	3
Trypsin-3	TRY3_HUMAN	33 kDa	1	2	0	0	0	25	1	1	0	1
Succinyl-CoA:3-ketoacid coenzyme A transferase 1	SCOT1_HUMAN	56 kDa	11	5	15	0	0	0	0	1	0	0
Collagen alpha-2(VI) chain	CO6A2_HUMAN	109 kDa	0	0	2	4	0	0	3	1	1	23
Peroxisomal multifunctional enzyme type 2	DHB4_HUMAN	80 kDa	0	0	0	1	30	1	0	1	1	0
Splicing factor, proline- and glutamine-rich	SFPQ_HUMAN	76 kDa	3	14	0	0	3	2	2	1	8	2
NADH dehydrogenase [ubiquinone] iron-sulfur protein 2	NDUS2_HUMAN	53 kDa	7	8	14	0	4	1	2	1	0	0
Bifunctional ATP-dependent dihydroxyacetone kinase/FAD-AMP lyase (cyclizing)	DHAK_HUMAN	59 kDa	0	0	0	0	36	0	0	1	0	0
Matrin-3	MATR3_HUMAN	95 kDa	7	18	0	0	3	2	0	1	9	0
60S ribosomal protein L6	RL6_HUMAN	33 kDa	0	1	0	0	4	28	0	1	4	3
D-3-phosphoglycerate dehydrogenase	SERA_HUMAN	57 kDa	6	5	0	0	3	25	0	1	0	2
Electron transfer flavoprotein-ubiquinone oxidoreductase	ETFD_HUMAN	68 kDa	0	1	17	0	19	5	0	1	0	0
NADH dehydrogenase [ubiquinone] iron-sulfur protein 3	NDUS3_HUMAN	30 kDa	8	8	13	0	6	5	1	1	2	0
Myosin light chain kinase, smooth muscle	MYLK_HUMAN	211 kDa	0	0	0	5	1	0	35	1	6	1
Adenylate kinase isoenzyme 1	KAD1_HUMAN	22 kDa	9	12	11	0	0	7	3	1	9	1
Vigilin	VIGLN_HUMAN	141 kDa	1	0	0	0	4	51	0	1	2	0
Retinal dehydrogenase 1	AL1A1_HUMAN	55 kDa	1	0	0	1	27	11	7	1	33	2
Dynamin-1	DYN1_HUMAN	97 kDa	41	54	0	0	0	0	0	1	0	0
Calcium-binding mitochondrial carrier protein Aralar1	CMC1_HUMAN	75 kDa	26	30	36	0	6	0	0	1	1	0
Myelin basic protein	MBP_HUMAN	33 kDa	69	133	0	0	1	0	0	1	0	15

Description	Accession	MW	Raw spectral counts									
			Frontal cortex	Cerebellum	Right ventricle	Mesentric lymph node	Liver	Pancreas	Proximal bile duct	Breast	Ovary	Clitoris
Dynein heavy chain 10, axonemal	DYH10_HUMAN	515 kDa	0	0	0	0	0	0	0	0	0	1
Dynein heavy chain 1, axonemal	DYH1_HUMAN	494 kDa	0	0	0	0	0	0	0	0	0	1
Adenylate kinase 9	KAD9_HUMAN	221 kDa	0	0	0	0	0	0	0	0	0	1
Zinc finger protein 516	ZN516_HUMAN	124 kDa	0	0	0	0	0	0	0	0	0	1
Putative ATP-dependent RNA helicase DDX12	DDX12_HUMAN	106 kDa	0	0	0	0	0	0	0	0	0	1
Ras-associated and pleckstrin homology domains-containing protein 1	RAPH1_HUMAN	135 kDa	0	0	0	0	0	0	0	0	0	1
Ectonucleoside triphosphate diphosphohydrolase 1	ENTP1_HUMAN	58 kDa	0	0	0	0	0	0	0	0	0	1
Endonuclease domain-containing 1 protein	ENDD1_HUMAN	55 kDa	0	0	0	0	0	0	0	0	0	1
Thrombospondin-4	TSP4_HUMAN	106 kDa	0	0	0	0	0	0	0	0	0	1
Splicing factor 1	SF01_HUMAN	68 kDa	0	0	0	0	0	0	0	0	0	1
Collagen alpha-1(XXVI) chain	COQA1_HUMAN	45 kDa	0	0	0	0	0	0	0	0	0	1
U6 snRNA-associated Sm-like protein LSm6	LSM6_HUMAN	9 kDa	0	0	0	0	0	0	0	0	0	1
Transcription regulator protein BACH2	BACH2_HUMAN	93 kDa	0	0	0	0	0	0	0	0	0	1
Transmembrane 9 superfamily member 3	TM9S3_HUMAN	68 kDa	0	0	0	0	0	0	0	0	0	1
Troponin C, skeletal muscle	TNNC2_HUMAN	18 kDa	0	0	0	0	0	0	0	0	0	1
Syntenin-1	SDCB1_HUMAN	32 kDa	0	0	0	0	0	0	0	0	0	1
Hematopoietic prostaglandin D synthase	HPGDS_HUMAN	23 kDa	0	0	0	0	0	0	0	0	0	1
Glucosamine-6-phosphate isomerase 1	GNPI1_HUMAN	33 kDa	0	0	0	0	0	0	0	0	0	1
Protein tyrosine phosphatase type IVA 2	TP4A2_HUMAN	19 kDa	0	0	0	0	0	0	0	0	0	1
Tyrosine-protein kinase SgK223	SG223_HUMAN	149 kDa	0	0	0	0	0	0	0	0	0	1
Afamin	AFAM_HUMAN	69 kDa	0	0	0	0	0	0	0	0	0	1
Protein S100-A7	S10A7_HUMAN	11 kDa	0	0	0	0	0	0	0	0	0	1
Eukaryotic translation initiation factor 4 gamma 3	IF4G3_HUMAN	177 kDa	0	0	0	0	0	0	0	0	0	1
Sister chromatid cohesion protein PDS5 homolog B	PDS5B_HUMAN	165 kDa	0	0	0	0	0	0	0	0	0	1
Ribose-phosphate pyrophosphokinase 2	PRPS2_HUMAN	35 kDa	0	0	0	0	0	0	0	0	0	1
Protein S100-A14	S10AE_HUMAN	12 kDa	0	0	0	0	0	0	0	0	0	1
Sodium/potassium-transporting ATPase subunit beta-3	AT1B3_HUMAN	32 kDa	0	0	0	0	0	0	0	0	0	1
Thioredoxin-related transmembrane protein 4	TMX4_HUMAN	39 kDa	0	0	0	0	0	0	0	0	0	1
Aldehyde dehydrogenase family 3 member B1	AL3B1_HUMAN	52 kDa	0	0	0	0	0	0	0	0	0	1
Putative RNA-binding protein 3	RBM3_HUMAN	17 kDa	0	0	0	0	0	0	0	0	0	1
cAMP-dependent protein kinase catalytic subunit alpha	KAPCA_HUMAN	41 kDa	0	0	0	0	0	0	0	0	0	1
Dynein light chain 2, cytoplasmic	DYL2_HUMAN	10 kDa	0	0	0	0	0	0	0	0	0	1
Collagen alpha-1(XV) chain	COFA1_HUMAN	142 kDa	0	0	0	0	0	0	0	0	0	1
Heme-binding protein 2	HEBP2_HUMAN	23 kDa	0	0	0	0	0	0	0	0	0	1
Tetratricopeptide repeat protein 28	TTC28_HUMAN	271 kDa	0	1	0	0	0	0	0	0	0	0
Protein KRI1 homolog	KRI1_HUMAN	83 kDa	0	1	0	0	0	0	0	0	0	0
Beta-adrenergic receptor kinase 1	ARBK1_HUMAN	80 kDa	0	1	0	0	0	0	0	0	0	0
Vacuolar protein sorting-associated protein 13D	VP13D_HUMAN	492 kDa	0	1	0	0	0	0	0	0	0	0
Activating signal cointegrator 1 complex subunit 3	ASCC3_HUMAN	251 kDa	0	1	0	0	0	0	0	0	0	0
Tyrosine-protein kinase BAZ1B	BAZ1B_HUMAN	171 kDa	0	1	0	0	0	0	0	0	0	0
PDZ domain-containing protein 2	PDZD2_HUMAN	302 kDa	0	1	0	0	0	0	0	0	0	0
Retinal-specific ATP-binding cassette transporter	ABCA4_HUMAN	256 kDa	0	1	0	0	0	0	0	0	0	0
Collagen alpha-1(XIII) chain	CODA1_HUMAN	70 kDa	0	1	0	0	0	0	0	0	0	0
ATP-binding cassette sub-family A member 7	ABCA7_HUMAN	234 kDa	0	1	0	0	0	0	0	0	0	0
DNA-directed RNA polymerase	RPOM_HUMAN	139 kDa	0	1	0	0	0	0	0	0	0	0
Tyrosine-protein kinase JAK3	JAK3_HUMAN	125 kDa	0	1	0	0	0	0	0	0	0	0
Sterile alpha motif domain-containing protein 9-like	SAM9L_HUMAN	185 kDa	0	1	0	0	0	0	0	0	0	0
Immunoglobulin superfamily member 10	IGS10_HUMAN	291 kDa	0	1	0	0	0	0	0	0	0	0
Coiled-coil domain-containing protein 136	CC136_HUMAN	134 kDa	0	1	0	0	0	0	0	0	0	0
Pleckstrin homology-like domain family B member 3	PHLB3_HUMAN	72 kDa	0	1	0	0	0	0	0	0	0	0
Myelin expression factor 2	MYEF2_HUMAN	64 kDa	0	1	0	0	0	0	0	0	0	0
Coiled-coil domain-containing protein 144A	C144A_HUMAN	165 kDa	0	1	0	0	0	0	0	0	0	0

Description	Accession	MW	Raw spectral counts									
			Frontal cortex	Cerebellum	Right ventricle	Mesentric lymph node	Liver	Pancreas	Proximal bile duct	Breast	Ovary	Clitoris
Protein phosphatase 1 regulatory subunit 3A	PPR3A_HUMAN	126 kDa	0	1	0	0	0	0	0	0	0	0
PDZ domain-containing protein 7	PDZD7_HUMAN	56 kDa	0	1	0	0	0	0	0	0	0	0
G2/mitotic-specific cyclin-B1	CCNB1_HUMAN	48 kDa	0	1	0	0	0	0	0	0	0	0
Protein kinase C epsilon type	KPCE_HUMAN	84 kDa	0	1	0	0	0	0	0	0	0	0
Importin subunit alpha-7	IMA7_HUMAN	60 kDa	0	1	0	0	0	0	0	0	0	0
Hyaluronan and proteoglycan link protein 1	HPLN1_HUMAN	40 kDa	0	1	0	0	0	0	0	0	0	0
Glutamate decarboxylase 1	DCE1_HUMAN	67 kDa	0	1	0	0	0	0	0	0	0	0
Chromodomain Y-like protein 2	CDYL2_HUMAN	57 kDa	0	1	0	0	0	0	0	0	0	0
Prolyl endopeptidase-like	PPCEL_HUMAN	84 kDa	0	1	0	0	0	0	0	0	0	0
Inward rectifier potassium channel 18	KCJ18_HUMAN	49 kDa	0	1	0	0	0	0	0	0	0	0
5'-nucleotidase domain-containing protein 3	NT5D3_HUMAN	63 kDa	0	1	0	0	0	0	0	0	0	0
Transmembrane protein 35	TMM35_HUMAN	18 kDa	0	1	0	0	0	0	0	0	0	0
39S ribosomal protein L50	RM50_HUMAN	18 kDa	0	1	0	0	0	0	0	0	0	0
Protein piccolo	PCLO_HUMAN	553 kDa	0	1	0	0	0	0	0	0	0	0
Sn1-specific diacylglycerol lipase alpha	DGLA_HUMAN	115 kDa	0	1	0	0	0	0	0	0	0	0
Cullin-4A	CUL4A_HUMAN	88 kDa	0	1	0	0	0	0	0	0	0	0
Monofunctional C1-tetrahydrofolate synthase	C1TM_HUMAN	106 kDa	0	1	0	0	0	0	0	0	0	0
Protein rogdi homolog	ROGDI_HUMAN	32 kDa	0	1	0	0	0	0	0	0	0	0
Na(+)/H(+) exchange regulatory cofactor NHE-RF3	NHRF3_HUMAN	57 kDa	0	1	0	0	0	0	0	0	0	0
Phosphatidate cytidylyltransferase 2	CDS2_HUMAN	51 kDa	0	1	0	0	0	0	0	0	0	0
Kinesin-like protein KIF1B	KIF1B_HUMAN	204 kDa	0	1	0	0	0	0	0	0	0	0
Cytoplasmic dynein 1 intermediate chain 1	DC1I1_HUMAN	73 kDa	0	1	0	0	0	0	0	0	0	0
Diphosphoinositol polyphosphate phosphohydrolase 1	NUDT3_HUMAN	19 kDa	0	1	0	0	0	0	0	0	0	0
Huntingtin	HD_HUMAN	348 kDa	0	1	0	0	0	0	0	0	0	0
Guanine nucleotide-binding protein-like 1	GNL1_HUMAN	69 kDa	0	1	0	0	0	0	0	0	0	0
Phospholipid-transporting ATPase IA	AT8A1_HUMAN	131 kDa	0	1	0	0	0	0	0	0	0	0
Neutral amino acid transporter A	SATT_HUMAN	56 kDa	0	1	0	0	0	0	0	0	0	0
Enolase-phosphatase E1	ENOPH_HUMAN	29 kDa	0	1	0	0	0	0	0	0	0	0
Voltage-gated potassium channel subunit beta-2	KCAB2_HUMAN	41 kDa	0	1	0	0	0	0	0	0	0	0
Ankyrin repeat and FYVE domain-containing protein 1	ANFY1_HUMAN	128 kDa	0	1	0	0	0	0	0	0	0	0
Cytoskeleton-associated protein 5	CKAP5_HUMAN	226 kDa	0	1	0	0	0	0	0	0	0	0
Src substrate cortactin	SRC8_HUMAN	62 kDa	0	1	0	0	0	0	0	0	0	0
Platelet-activating factor acetylhydrolase IB subunit alpha	LIS1_HUMAN	47 kDa	0	1	0	0	0	0	0	0	0	0
Coiled-coil domain-containing protein 132	CC132_HUMAN	111 kDa	0	1	0	0	0	0	0	0	0	0
StAR-related lipid transfer protein 9	STAR9_HUMAN	516 kDa	0	1	0	0	0	0	0	0	0	0
Mucin-16	MUC16_HUMAN	2353 kDa	0	0	1	0	0	0	0	0	0	0
Phosphorylase b kinase regulatory subunit alpha, liver isoform	KPB2_HUMAN	138 kDa	0	0	1	0	0	0	0	0	0	0
Brefeldin A-inhibited guanine nucleotide-exchange protein 2	BIG2_HUMAN	202 kDa	0	0	1	0	0	0	0	0	0	0
Laminin subunit alpha-2	LAMA2_HUMAN	344 kDa	0	0	1	0	0	0	0	0	0	0
Xin actin-binding repeat-containing protein 1	XIRP1_HUMAN	199 kDa	0	0	1	0	0	0	0	0	0	0
von Willebrand factor A domain-containing protein 8	VWA8_HUMAN	215 kDa	0	0	1	0	0	0	0	0	0	0
Intersectin-2	ITSN2_HUMAN	193 kDa	0	0	1	0	0	0	0	0	0	0
Prosaposin receptor GPR37	GPR37_HUMAN	67 kDa	0	0	1	0	0	0	0	0	0	0
Protocadherin-23	PCD23_HUMAN	322 kDa	0	0	1	0	0	0	0	0	0	0
T-lymphoma invasion and metastasis-inducing protein 2	TIAM2_HUMAN	190 kDa	0	0	1	0	0	0	0	0	0	0
Caspase recruitment domain-containing protein 14	CAR14_HUMAN	113 kDa	0	0	1	0	0	0	0	0	0	0
Aminomethyltransferase	GCST_HUMAN	44 kDa	0	0	1	0	0	0	0	0	0	0
Peroxidasin-like protein	PXDNL_HUMAN	164 kDa	0	0	1	0	0	0	0	0	0	0
Adenylate cyclase type 1	ADCY1_HUMAN	123 kDa	0	0	1	0	0	0	0	0	0	0
Vam6/Vps39-like protein	VPS39_HUMAN	102 kDa	0	0	1	0	0	0	0	0	0	0
Protein-cysteine N-palmitoyltransferase HHAT-like protein	HHATL_HUMAN	57 kDa	0	0	1	0	0	0	0	0	0	0
Adenylate cyclase type 3	ADCY3_HUMAN	129 kDa	0	0	1	0	0	0	0	0	0	0

Description	Accession	MW	Raw spectral counts									
			Frontal cortex	Cerebellum	Right ventricle	Mesentric lymph node	Liver	Pancreas	Proximal bile duct	Breast	Ovary	Clitoris
Protein DENND6B	DEN6B_HUMAN	66 kDa	0	0	1	0	0	0	0	0	0	0
Enhancer of filamentation 1	CASL_HUMAN	93 kDa	0	0	1	0	0	0	0	0	0	0
Cytokine receptor common subunit beta	IL3RB_HUMAN	97 kDa	0	0	1	0	0	0	0	0	0	0
[Pyruvate dehydrogenase (acetyl-transferring)] kinase isozyme 2	PDK2_HUMAN	46 kDa	0	0	1	0	0	0	0	0	0	0
Protein TBRG4	TBRG4_HUMAN	71 kDa	0	0	1	0	0	0	0	0	0	0
FERM domain-containing protein 7	FRMD7_HUMAN	82 kDa	0	0	1	0	0	0	0	0	0	0
Steroid 21-hydroxylase	CP21A_HUMAN	56 kDa	0	0	1	0	0	0	0	0	0	0
Ig lambda chain V-I region NEW	LV103_HUMAN	11 kDa	0	0	1	0	0	0	0	0	0	0
NEDD8-activating enzyme E1 regulatory subunit	ULA1_HUMAN	60 kDa	0	0	1	0	0	0	0	0	0	0
Muscle-related coiled-coil protein	MURC_HUMAN	42 kDa	0	0	1	0	0	0	0	0	0	0
Calmegin	CLGN_HUMAN	70 kDa	0	0	1	0	0	0	0	0	0	0
Uncharacterized protein C6orf203	CF203_HUMAN	28 kDa	0	0	1	0	0	0	0	0	0	0
PGC-1 and ERR-induced regulator in muscle protein 1	PERM1_HUMAN	81 kDa	0	0	1	0	0	0	0	0	0	0
Probable cysteine--tRNA ligase	SYCM_HUMAN	62 kDa	0	0	1	0	0	0	0	0	0	0
Tropomodulin-1	TMOD1_HUMAN	41 kDa	0	0	1	0	0	0	0	0	0	0
NADH dehydrogenase [ubiquinone] 1 beta subcomplex subunit 4	NDUB4_HUMAN	15 kDa	0	0	1	0	0	0	0	0	0	0
Inositol-3-phosphate synthase 1	INO1_HUMAN	61 kDa	0	0	1	0	0	0	0	0	0	0
Homeodomain-interacting protein kinase 2	HIPK2_HUMAN	131 kDa	0	0	1	0	0	0	0	0	0	0
Optic atrophy 3 protein	OPA3_HUMAN	20 kDa	0	0	1	0	0	0	0	0	0	0
28S ribosomal protein S25	RT25_HUMAN	20 kDa	0	0	1	0	0	0	0	0	0	0
NADH-ubiquinone oxidoreductase chain 1	NU1M_HUMAN	36 kDa	0	0	1	0	0	0	0	0	0	0
Chromatin modification-related protein MEAF6	EAF6_HUMAN	22 kDa	0	0	1	0	0	0	0	0	0	0
Cytochrome b-c1 complex subunit 6	QCR6_HUMAN	11 kDa	0	0	1	0	0	0	0	0	0	0
Trans-2-enoyl-CoA reductase	MECR_HUMAN	40 kDa	0	0	1	0	0	0	0	0	0	0
Adenylosuccinate synthetase isozyme 1	PURA1_HUMAN	50 kDa	0	0	1	0	0	0	0	0	0	0
Nidogen-1	NID1_HUMAN	136 kDa	0	0	1	0	0	0	0	0	0	0
Structural maintenance of chromosomes protein 4	SMC4_HUMAN	147 kDa	0	0	1	0	0	0	0	0	0	0
Angiotensinogen	ANGT_HUMAN	53 kDa	0	0	1	0	0	0	0	0	0	0
DCC-interacting protein 13-alpha	DP13A_HUMAN	80 kDa	0	0	1	0	0	0	0	0	0	0
Retinoid isomerohydrolase	RPE65_HUMAN	61 kDa	0	0	1	0	0	0	0	0	0	0
Serine protease HTRA2	HTRA2_HUMAN	49 kDa	0	0	1	0	0	0	0	0	0	0
Protein unc-45 homolog A	UN45A_HUMAN	103 kDa	0	0	1	0	0	0	0	0	0	0
Ribosome-releasing factor 2	RRF2M_HUMAN	87 kDa	0	0	1	0	0	0	0	0	0	0
Serum amyloid A-4 protein	SAA4_HUMAN	15 kDa	0	0	1	0	0	0	0	0	0	0
Succinate dehydrogenase [ubiquinone] cytochrome b small subunit	DHSD_HUMAN	17 kDa	0	0	1	0	0	0	0	0	0	0
39S ribosomal protein L20	RM20_HUMAN	17 kDa	0	0	1	0	0	0	0	0	0	0
Transmembrane protein 65	TMM65_HUMAN	25 kDa	0	0	1	0	0	0	0	0	0	0
Alpha-1B-glycoprotein	A1BG_HUMAN	54 kDa	0	0	1	0	0	0	0	0	0	0
Gamma-sarcoglycan	SGCG_HUMAN	32 kDa	0	0	1	0	0	0	0	0	0	0
Reticulon-4-interacting protein 1	RT4I1_HUMAN	44 kDa	0	0	1	0	0	0	0	0	0	0
Ubiquinol-cytochrome-c reductase complex assembly factor 1	UQCC1_HUMAN	35 kDa	0	0	1	0	0	0	0	0	0	0
Protein NipSnap homolog 3B	NPS3B_HUMAN	28 kDa	0	0	1	0	0	0	0	0	0	0
Apolipoprotein C-II	APOC2_HUMAN	11 kDa	0	0	1	0	0	0	0	0	0	0
DENN domain-containing protein 5A	DEN5A_HUMAN	147 kDa	0	0	1	0	0	0	0	0	0	0
3-oxoacyl-[acyl-carrier-protein] synthase	OXSM_HUMAN	49 kDa	0	0	1	0	0	0	0	0	0	0
Protein FAM162A	F162A_HUMAN	17 kDa	0	0	1	0	0	0	0	0	0	0
OCIA domain-containing protein 1	OCAD1_HUMAN	28 kDa	0	0	1	0	0	0	0	0	0	0
Protein QIL1	QIL1_HUMAN	13 kDa	0	0	1	0	0	0	0	0	0	0
Isoamyl acetate-hydrolyzing esterase 1 homolog	IAH1_HUMAN	28 kDa	0	0	1	0	0	0	0	0	0	0
Calpastatin	ICAL_HUMAN	77 kDa	0	0	1	0	0	0	0	0	0	0
Cytochrome c oxidase subunit 1	COX1_HUMAN	57 kDa	0	0	1	0	0	0	0	0	0	0
Protein unc-45 homolog B	UN45B_HUMAN	104 kDa	0	0	1	0	0	0	0	0	0	0

Description	Accession	MW	Raw spectral counts									
			Frontal cortex	Cerebellum	Right ventricle	Mesentric lymph node	Liver	Pancreas	Proximal bile duct	Breast	Ovary	Clitoris
Histone-lysine N-methyltransferase SMYD1	SMYD1_HUMAN	57 kDa	0	0	1	0	0	0	0	0	0	0
Protein SON	SON_HUMAN	264 kDa	0	0	0	0	0	1	0	0	0	0
Hypoxia-inducible factor 3-alpha	HIF3A_HUMAN	72 kDa	0	0	0	0	0	1	0	0	0	0
Collagen alpha-5(VI) chain	CO6A5_HUMAN	290 kDa	0	0	0	0	0	1	0	0	0	0
Alpha-mannosidase 2x	MA2A2_HUMAN	131 kDa	0	0	0	0	0	1	0	0	0	0
Centrosomal protein of 83 kDa	CEP83_HUMAN	82 kDa	0	0	0	0	0	1	0	0	0	0
Neuron navigator 3	NAV3_HUMAN	256 kDa	0	0	0	0	0	1	0	0	0	0
A-kinase anchor protein 4	AKAP4_HUMAN	94 kDa	0	0	0	0	0	1	0	0	0	0
AT-rich interactive domain-containing protein 4B	ARI4B_HUMAN	148 kDa	0	0	0	0	0	1	0	0	0	0
Methylthioribose-1-phosphate isomerase	MTNA_HUMAN	39 kDa	0	0	0	0	0	1	0	0	0	0
SEC23-interacting protein	S23IP_HUMAN	111 kDa	0	0	0	0	0	1	0	0	0	0
Histone acetyltransferase KAT2B	KAT2B_HUMAN	93 kDa	0	0	0	0	0	1	0	0	0	0
Beta-1-syntrophin	SNTB1_HUMAN	58 kDa	0	0	0	0	0	1	0	0	0	0
Superkiller viralicidic activity 2-like 2	SK2L2_HUMAN	118 kDa	0	0	0	0	0	1	0	0	0	0
Lymphoid-restricted membrane protein	LRMP_HUMAN	62 kDa	0	0	0	0	0	1	0	0	0	0
28S ribosomal protein S29	RT29_HUMAN	46 kDa	0	0	0	0	0	1	0	0	0	0
Oligoribonuclease	ORN_HUMAN	27 kDa	0	0	0	0	0	1	0	0	0	0
RIMS-binding protein 2	RIMB2_HUMAN	116 kDa	0	0	0	0	0	1	0	0	0	0
DnaJ homolog subfamily C member 10	DJC10_HUMAN	91 kDa	0	0	0	0	0	1	0	0	0	0
Plasmalemma vesicle-associated protein	PLVAP_HUMAN	51 kDa	0	0	0	0	0	1	0	0	0	0
Prospero homeobox protein 1	PROX1_HUMAN	83 kDa	0	0	0	0	0	1	0	0	0	0
Small nuclear ribonucleoprotein G	RUXG_HUMAN	8 kDa	0	0	0	0	0	1	0	0	0	0
FACT complex subunit SSRP1	SSRP1_HUMAN	81 kDa	0	0	0	0	0	1	0	0	0	0
Elongation factor G	EFGM_HUMAN	83 kDa	0	0	0	0	0	1	0	0	0	0
Methylmalonyl-CoA epimerase	MCEE_HUMAN	19 kDa	0	0	0	0	0	1	0	0	0	0
Small glutamine-rich tetratricopeptide repeat-containing protein alpha	SGTA_HUMAN	34 kDa	0	0	0	0	0	1	0	0	0	0
Mitochondrial import receptor subunit TOM40 homolog	TOM40_HUMAN	38 kDa	0	0	0	0	0	1	0	0	0	0
cTAGE family member 9	CTGE9_HUMAN	88 kDa	0	0	0	0	0	1	0	0	0	0
Cell adhesion molecule-related/down-regulated by oncogenes	CDON_HUMAN	139 kDa	0	0	0	0	0	1	0	0	0	0
Calcium uniporter protein	MCU_HUMAN	40 kDa	0	0	0	0	0	1	0	0	0	0
ER membrane protein complex subunit 8	EMC8_HUMAN	24 kDa	0	0	0	0	0	1	0	0	0	0
Carbonic anhydrase 14	CAH14_HUMAN	38 kDa	0	0	0	0	0	1	0	0	0	0
Phosphoacetylglucosamine mutase	AGM1_HUMAN	60 kDa	0	0	0	0	0	1	0	0	0	0
Calcium and integrin-binding protein 1	CIB1_HUMAN	22 kDa	0	0	0	0	0	1	0	0	0	0
60S ribosomal protein L3-like	RL3L_HUMAN	46 kDa	0	0	0	0	0	1	0	0	0	0
Ufm1-specific protease 2	UFSP2_HUMAN	53 kDa	0	0	0	0	0	1	0	0	0	0
DnaJ homolog subfamily C member 25	DJC25_HUMAN	42 kDa	0	0	0	0	0	1	0	0	0	0
Zinc transporter SLC39A7	S39A7_HUMAN	50 kDa	0	0	0	0	0	1	0	0	0	0
RWD domain-containing protein 1	RWDD1_HUMAN	28 kDa	0	0	0	0	0	1	0	0	0	0
Tumor necrosis factor alpha-induced protein 8	TFIP8_HUMAN	23 kDa	0	0	0	0	0	1	0	0	0	0
DNA-directed RNA polymerase II subunit RPB4	RPB4_HUMAN	16 kDa	0	0	0	0	0	1	0	0	0	0
60S ribosomal protein L22-like 1	RL22L_HUMAN	15 kDa	0	0	0	0	0	1	0	0	0	0
Eukaryotic translation initiation factor 1b	EIF1B_HUMAN	13 kDa	0	0	0	0	0	1	0	0	0	0
Serine/threonine-protein phosphatase 2A 56 kDa regulatory subunit	2A5G_HUMAN	61 kDa	0	0	0	0	0	1	0	0	0	0
Putative peptidyl-tRNA hydrolase PTRHD1	PTRD1_HUMAN	16 kDa	0	0	0	0	0	1	0	0	0	0
Ubiquilin-1	UBQL1_HUMAN	63 kDa	0	0	0	0	0	1	0	0	0	0
Peripheral plasma membrane protein CASK	CSKP_HUMAN	105 kDa	0	0	0	0	0	1	0	0	0	0
VWFA and cache domain-containing protein 1	CAHD1_HUMAN	142 kDa	0	0	0	0	0	1	0	0	0	0
60S ribosomal protein L34	RL34_HUMAN	13 kDa	0	0	0	0	0	1	0	0	0	0
Contactin-5	CNTN5_HUMAN	121 kDa	0	0	0	0	0	1	0	0	0	0
Monocarboxylate transporter 10	MOT10_HUMAN	55 kDa	0	0	0	0	0	1	0	0	0	0
Protein pelota homolog	PELO_HUMAN	43 kDa	0	0	0	0	0	1	0	0	0	0

Description	Accession	MW	Raw spectral counts									
			Frontal cortex	Cerebellum	Right ventricle	Mesentric lymph node	Liver	Pancreas	Proximal bile duct	Breast	Ovary	Clitoris
Aldehyde dehydrogenase family 16 member A1	A16A1_HUMAN	85 kDa	0	0	0	0	0	1	0	0	0	0
PDZ domain-containing protein GIPC2	GIPC2_HUMAN	34 kDa	0	0	0	0	0	1	0	0	0	0
Diphthine synthase	DPH5_HUMAN	32 kDa	0	0	0	0	0	1	0	0	0	0
15 kDa selenoprotein	SEP15_HUMAN	18 kDa	0	0	0	0	0	1	0	0	0	0
GDP-mannose 4,6 dehydratase	GMDS_HUMAN	42 kDa	0	0	0	0	0	1	0	0	0	0
Inosine-5'-monophosphate dehydrogenase 2	IMDH2_HUMAN	56 kDa	0	0	0	0	0	1	0	0	0	0
Probable D-tyrosyl-tRNA(Tyr) deacylase 2	DTD2_HUMAN	19 kDa	0	0	0	0	0	1	0	0	0	0
Eukaryotic translation initiation factor 1A, Y-chromosomal	IF1AY_HUMAN	16 kDa	0	0	0	0	0	1	0	0	0	0
Thioredoxin-related transmembrane protein 1	TMX1_HUMAN	32 kDa	0	0	0	0	0	1	0	0	0	0
Asparagine synthetase [glutamine-hydrolyzing]	ASNS_HUMAN	64 kDa	0	0	0	0	0	1	0	0	0	0
Histone-lysine N-methyltransferase ASH1L	ASH1L_HUMAN	333 kDa	0	0	0	0	0	1	0	0	0	0
O-acetyl-ADP-ribose deacetylase 1	OARD1_HUMAN	17 kDa	0	0	0	0	0	1	0	0	0	0
40S ribosomal protein S12	RS12_HUMAN	15 kDa	0	0	0	0	0	1	0	0	0	0
Prolyl endopeptidase	PPCE_HUMAN	81 kDa	0	0	0	0	0	1	0	0	0	0
Pre-mRNA-processing-splicing factor 8	PRP8_HUMAN	274 kDa	0	0	0	0	0	1	0	0	0	0
Y-box-binding protein 3	YBOX3_HUMAN	40 kDa	0	0	0	0	0	1	0	0	0	0
Calponin-2	CNN2_HUMAN	34 kDa	0	0	0	0	0	1	0	0	0	0
Translocation protein SEC63 homolog	SEC63_HUMAN	88 kDa	0	0	0	0	0	1	0	0	0	0
Echinoderm microtubule-associated protein-like 2	EMAL2_HUMAN	71 kDa	0	0	0	0	0	1	0	0	0	0
Utrophin	UTRO_HUMAN	394 kDa	0	0	0	0	0	1	0	0	0	0
DNA damage-binding protein 1	DDB1_HUMAN	127 kDa	0	0	0	0	0	1	0	0	0	0
Nucleosome-remodeling factor subunit BPTF	BPTF_HUMAN	338 kDa	0	0	0	0	0	1	0	0	0	0
Catechol O-methyltransferase domain-containing protein 1	CMTD1_HUMAN	29 kDa	0	0	0	0	0	1	0	0	0	0
Dynein heavy chain 5, axonemal	DYH5_HUMAN	529 kDa	0	0	0	0	0	0	1	0	0	0
Myosin phosphatase Rho-interacting protein	MPRIP_HUMAN	117 kDa	0	0	0	0	0	0	1	0	0	0
Tensin-1	TENS1_HUMAN	186 kDa	0	0	0	0	0	0	1	0	0	0
Protein Daple	DAPLE_HUMAN	228 kDa	0	0	0	0	0	0	1	0	0	0
Sodium channel protein type 3 subunit alpha	SCN3A_HUMAN	226 kDa	0	0	0	0	0	0	1	0	0	0
Ephrin type-A receptor 2	EPHA2_HUMAN	108 kDa	0	0	0	0	0	0	1	0	0	0
Ankyrin repeat and KH domain-containing protein 1	ANKH1_HUMAN	269 kDa	0	0	0	0	0	0	1	0	0	0
Kinesin-like protein KIF15	KIF15_HUMAN	160 kDa	0	0	0	0	0	0	1	0	0	0
Neuron navigator 1	NAV1_HUMAN	202 kDa	0	0	0	0	0	0	1	0	0	0
Cyclin-dependent kinase 12	CDK12_HUMAN	164 kDa	0	0	0	0	0	0	1	0	0	0
NEDD8 ultimate buster 1	NUB1_HUMAN	71 kDa	0	0	0	0	0	0	1	0	0	0
Mitogen-activated protein kinase kinase kinase MLK4	M3KL4_HUMAN	114 kDa	0	0	0	0	0	0	1	0	0	0
Plasminogen	PLMN_HUMAN	91 kDa	0	0	0	0	0	0	1	0	0	0
Adenylate cyclase type 5	ADCY5_HUMAN	139 kDa	0	0	0	0	0	0	1	0	0	0
Constitutive coactivator of PPAR-gamma-like protein 1	F120A_HUMAN	122 kDa	0	0	0	0	0	0	1	0	0	0
Inositol-trisphosphate 3-kinase C	IP3KC_HUMAN	75 kDa	0	0	0	0	0	0	1	0	0	0
Transmembrane protein 2	TMEM2_HUMAN	154 kDa	0	0	0	0	0	0	1	0	0	0
Mitotic checkpoint serine/threonine-protein kinase BUB1 beta	BUB1B_HUMAN	120 kDa	0	0	0	0	0	0	1	0	0	0
Constitutive coactivator of PPAR-gamma-like protein 2	F120C_HUMAN	121 kDa	0	0	0	0	0	0	1	0	0	0
Histone lysine demethylase PHF8	PHF8_HUMAN	118 kDa	0	0	0	0	0	0	1	0	0	0
Chromodomain-helicase-DNA-binding protein 1	CHD1_HUMAN	197 kDa	0	0	0	0	0	0	1	0	0	0
Integrin alpha-3	ITA3_HUMAN	117 kDa	0	0	0	0	0	0	1	0	0	0
PDZ and LIM domain protein 4	PDLI4_HUMAN	35 kDa	0	0	0	0	0	0	1	0	0	0
Kelch repeat and BTB domain-containing protein 2	KBTB2_HUMAN	71 kDa	0	0	0	0	0	0	1	0	0	0
B-lymphocyte antigen CD19	CD19_HUMAN	61 kDa	0	0	0	0	0	0	1	0	0	0
Anterior gradient protein 2 homolog	AGR2_HUMAN	20 kDa	0	0	0	0	0	0	1	0	0	0
Septin-10	SEP10_HUMAN	53 kDa	0	0	0	0	0	0	1	0	0	0
Target of Nesh-SH3	TARSH_HUMAN	119 kDa	0	0	0	0	0	0	1	0	0	0
Retinal dehydrogenase 2	AL1A2_HUMAN	57 kDa	0	0	0	0	0	0	1	0	0	0

Description	Accession	MW	Raw spectral counts									
			Frontal cortex	Cerebellum	Right ventricle	Mesentric lymph node	Liver	Pancreas	Proximal bile duct	Breast	Ovary	Clitoris
Transcription factor TFIIIB component B'' homolog	BDP1_HUMAN	294 kDa	0	0	0	0	0	0	0	0	1	0
Centrosomal protein of 152 kDa	CE152_HUMAN	196 kDa	0	0	0	0	0	0	0	0	1	0
Sickle tail protein homolog	SKT_HUMAN	214 kDa	0	0	0	0	0	0	0	0	1	0
Teneurin-2	TEN2_HUMAN	308 kDa	0	0	0	0	0	0	0	0	1	0
RUN domain-containing protein 3A	RUN3A_HUMAN	50 kDa	0	0	0	0	0	0	0	0	1	0
Translation initiation factor eIF-2B subunit epsilon	EI2BE_HUMAN	80 kDa	0	0	0	0	0	0	0	0	1	0
Microtubule-associated serine/threonine-protein kinase 4	MAST4_HUMAN	284 kDa	0	0	0	0	0	0	0	0	1	0
Golgin subfamily A member 6-like protein 22	GG6LV_HUMAN	108 kDa	0	0	0	0	0	0	0	0	1	0
Probable ubiquitin carboxyl-terminal hydrolase FAF-X	USP9X_HUMAN	292 kDa	0	0	0	0	0	0	0	0	1	0
Citron Rho-interacting kinase	CTRO_HUMAN	231 kDa	0	0	0	0	0	0	0	0	1	0
Collagen alpha-1(XXI) chain	COLA1_HUMAN	99 kDa	0	0	0	0	0	0	0	0	1	0
Coiled-coil domain-containing protein 22	CCD22_HUMAN	71 kDa	0	0	0	0	0	0	0	0	1	0
Unconventional myosin-Id	MYO1D_HUMAN	116 kDa	0	0	0	0	0	0	0	0	1	0
HEAT repeat-containing protein 5B	HTR5B_HUMAN	224 kDa	0	0	0	0	0	0	0	0	1	0
General transcription factor II-I	GTF2I_HUMAN	112 kDa	0	0	0	0	0	0	0	0	1	0
Uncharacterized protein CXorf22	CX022_HUMAN	110 kDa	0	0	0	0	0	0	0	0	1	0
Cohesin subunit SA-2	STAG2_HUMAN	141 kDa	0	0	0	0	0	0	0	0	1	0
COP9 signalosome complex subunit 2	CSN2_HUMAN	52 kDa	0	0	0	0	0	0	0	0	1	0
DNA topoisomerase 2-alpha	TOP2A_HUMAN	174 kDa	0	0	0	0	0	0	0	0	1	0
Transmembrane and TPR repeat-containing protein 3	TMTC3_HUMAN	104 kDa	0	0	0	0	0	0	0	0	1	0
U4/U6.U5 tri-snRNP-associated protein 1	SNUT1_HUMAN	90 kDa	0	0	0	0	0	0	0	0	1	0
Probable global transcription activator SNF2L2	SMCA2_HUMAN	181 kDa	0	0	0	0	0	0	0	0	1	0
Pleckstrin homology domain-containing family M member 2	PKHM2_HUMAN	113 kDa	0	0	0	0	0	0	0	0	1	0
Glypican-6	GPC6_HUMAN	63 kDa	0	0	0	0	0	0	0	0	1	0
Protein Red	RED_HUMAN	66 kDa	0	0	0	0	0	0	0	0	1	0
Serine/threonine-protein kinase Nek9	NEK9_HUMAN	107 kDa	0	0	0	0	0	0	0	0	1	0
Protein Hook homolog 3	HOOK3_HUMAN	83 kDa	0	0	0	0	0	0	0	0	1	0
Protein FAM200A	F200A_HUMAN	66 kDa	0	0	0	0	0	0	0	0	1	0
Bifunctional protein NCOAT	NCOAT_HUMAN	103 kDa	0	0	0	0	0	0	0	0	1	0
Echinoderm microtubule-associated protein-like 4	EMAL4_HUMAN	109 kDa	0	0	0	0	0	0	0	0	1	0
Ceramide synthase 2	CERS2_HUMAN	45 kDa	0	0	0	0	0	0	0	0	1	0
Proliferating cell nuclear antigen	PCNA_HUMAN	29 kDa	0	0	0	0	0	0	0	0	1	0
Dermatan-sulfate epimerase	DSE_HUMAN	110 kDa	0	0	0	0	0	0	0	0	1	0
Selenoprotein H	SELH_HUMAN	13 kDa	0	0	0	0	0	0	0	0	1	0
HD domain-containing protein 2	HDDC2_HUMAN	23 kDa	0	0	0	0	0	0	0	0	1	0
RNA-binding protein 4B	RBM4B_HUMAN	40 kDa	0	0	0	0	0	0	0	0	1	0
Synembryn-A	RIC8A_HUMAN	60 kDa	0	0	0	0	0	0	0	0	1	0
N-acetylated-alpha-linked acidic dipeptidase 2	NALD2_HUMAN	84 kDa	0	0	0	0	0	0	0	0	1	0
Splicing factor U2AF 35 kDa subunit	U2AF1_HUMAN	28 kDa	0	0	0	0	0	0	0	0	1	0
Parkinson disease 7 domain-containing protein 1	PDDC1_HUMAN	23 kDa	0	0	0	0	0	0	0	0	1	0
UBX domain-containing protein 1	UBXN1_HUMAN	33 kDa	0	0	0	0	0	0	0	0	1	0
Ubiquitin-conjugating enzyme E2 Z	UBE2Z_HUMAN	38 kDa	0	0	0	0	0	0	0	0	1	0
Serine/threonine-protein kinase Nek7	NEK7_HUMAN	35 kDa	0	0	0	0	0	0	0	0	1	0
DENN domain-containing protein 1B	DEN1B_HUMAN	48 kDa	0	0	0	0	0	0	0	0	1	0
Signal peptide peptidase-like 3	SPPL3_HUMAN	43 kDa	0	0	0	0	0	0	0	0	1	0
Ribonuclease T2	RNT2_HUMAN	29 kDa	0	0	0	0	0	0	0	0	1	0
Pre-mRNA-splicing factor ISY1 homolog	ISY1_HUMAN	33 kDa	0	0	0	0	0	0	0	0	1	0
Dolichol-phosphate mannosyltransferase subunit 3	DPM3_HUMAN	10 kDa	0	0	0	0	0	0	0	0	1	0
Retinol dehydrogenase 13	RDH13_HUMAN	36 kDa	0	0	0	0	0	0	0	0	1	0
Nucleoporin SEH1	SEH1_HUMAN	40 kDa	0	0	0	0	0	0	0	0	1	0
Prefoldin subunit 1	PFD1_HUMAN	14 kDa	0	0	0	0	0	0	0	0	1	0
BAG family molecular chaperone regulator 5	BAG5_HUMAN	51 kDa	0	0	0	0	0	0	0	0	1	0

Description	Accession	MW	Raw spectral counts									
			Frontal cortex	Cerebellum	Right ventricle	Mesentric lymph node	Liver	Pancreas	Proximal bile duct	Breast	Ovary	Clitoris
U6 snRNA-associated Sm-like protein LSm3	LSM3_HUMAN	12 kDa	0	0	0	0	0	0	0	0	1	0
Phytanoyl-CoA dioxygenase domain-containing protein 1	PHYD1_HUMAN	32 kDa	0	0	0	0	0	0	0	0	1	0
U1 small nuclear ribonucleoprotein C	RU1C_HUMAN	17 kDa	0	0	0	0	0	0	0	0	1	0
2'-deoxynucleoside 5'-phosphate N-hydrolase 1	DNPH1_HUMAN	19 kDa	0	0	0	0	0	0	0	0	1	0
PDZ domain-containing protein 11	PDZ11_HUMAN	16 kDa	0	0	0	0	0	0	0	0	1	0
Protein FAM98B	FA98B_HUMAN	37 kDa	0	0	0	0	0	0	0	0	1	0
Malignant T-cell-amplified sequence 1	MCTS1_HUMAN	21 kDa	0	0	0	0	0	0	0	0	1	0
CLIP-associating protein 2	CLAP2_HUMAN	141 kDa	0	0	0	0	0	0	0	0	1	0
TIP41-like protein	TIPRL_HUMAN	31 kDa	0	0	0	0	0	0	0	0	1	0
Phosphoribosyl pyrophosphate synthase-associated protein 1	KPRA_HUMAN	39 kDa	0	0	0	0	0	0	0	0	1	0
U1 small nuclear ribonucleoprotein 70 kDa	RU17_HUMAN	52 kDa	0	0	0	0	0	0	0	0	1	0
Acylamino-acid-releasing enzyme	ACPH_HUMAN	81 kDa	0	0	0	0	0	0	0	0	1	0
Sorting nexin-6	SNX6_HUMAN	47 kDa	0	0	0	0	0	0	0	0	1	0
Tyrosine-protein kinase CSK	CSK_HUMAN	51 kDa	0	0	0	0	0	0	0	0	1	0
Hepatoma-derived growth factor	HDGF_HUMAN	27 kDa	0	0	0	0	0	0	0	0	1	0
26S proteasome non-ATPase regulatory subunit 10	PSD10_HUMAN	24 kDa	0	0	0	0	0	0	0	0	1	0
Tropomodulin-3	TMOD3_HUMAN	40 kDa	0	0	0	0	0	0	0	0	1	0
Thimet oligopeptidase	THOP1_HUMAN	79 kDa	0	0	0	0	0	0	0	0	1	0
Protein LZIC	LZIC_HUMAN	21 kDa	0	0	0	0	0	0	0	0	1	0
Translation initiation factor eIF-2B subunit alpha	EI2BA_HUMAN	34 kDa	0	0	0	0	0	0	0	0	1	0
m7GpppX diphosphatase	DCPS_HUMAN	39 kDa	0	0	0	0	0	0	0	0	1	0
Dynactin subunit 3	DCTN3_HUMAN	21 kDa	0	0	0	0	0	0	0	0	1	0
Latexin	LXN_HUMAN	26 kDa	0	0	0	0	0	0	0	0	1	0
E3 ubiquitin-protein ligase TRIP12	TRIPC_HUMAN	220 kDa	0	0	0	0	0	0	0	0	1	0
CAD protein	PYR1_HUMAN	243 kDa	0	0	0	0	0	0	0	0	1	0
Epidermal growth factor receptor	EGFR_HUMAN	134 kDa	0	0	0	0	0	0	0	0	1	0
RNA-binding protein 25	RBM25_HUMAN	100 kDa	0	0	0	0	0	0	0	0	1	0
Eukaryotic initiation factor 4A-III	IF4A3_HUMAN	47 kDa	0	0	0	0	0	0	0	0	1	0
Cell cycle and apoptosis regulator protein 2	CCAR2_HUMAN	103 kDa	0	0	0	0	0	0	0	0	1	0
E3 SUMO-protein ligase RanBP2	RBP2_HUMAN	358 kDa	0	0	0	0	0	0	0	0	1	0
SWI/SNF complex subunit SMARCC2	SMRC2_HUMAN	133 kDa	0	0	0	0	0	0	0	0	1	0
Dual specificity protein phosphatase 23	DUS23_HUMAN	17 kDa	0	0	0	0	0	0	0	0	1	0
DnaJ homolog subfamily B member 1	DNJB1_HUMAN	38 kDa	0	0	0	0	0	0	0	0	1	0
Selenide, water dikinase 1	SPS1_HUMAN	43 kDa	0	0	0	0	0	0	0	0	1	0
Endoplasmic reticulum-Golgi intermediate compartment protein 1	ERGI1_HUMAN	33 kDa	0	0	0	0	0	0	0	0	1	0
Arfaptin-1	ARFP1_HUMAN	42 kDa	0	0	0	0	0	0	0	0	1	0
Integrin alpha-5	ITA5_HUMAN	115 kDa	0	0	0	0	0	0	0	0	1	0
Nicastrin	NICA_HUMAN	78 kDa	0	0	0	0	0	0	0	0	1	0
Thrombospondin-1	TSP1_HUMAN	129 kDa	0	0	0	0	0	0	0	0	1	0
Protein phosphatase 1 regulatory subunit 12B	MYPT2_HUMAN	110 kDa	0	0	0	0	0	0	0	0	1	0
E3 ubiquitin-protein ligase LRSAM1	LRSM1_HUMAN	84 kDa	0	0	0	0	0	0	0	0	1	0
NAD-dependent protein deacylase sirtuin-5	SIR5_HUMAN	34 kDa	0	0	0	0	0	0	0	0	1	0
COMM domain-containing protein 2	COMD2_HUMAN	23 kDa	0	0	0	0	0	0	0	0	1	0
Serine/threonine-protein phosphatase 2A 55 kDa regulatory subunit B	2ABA_HUMAN	52 kDa	0	0	0	0	0	0	0	0	1	0
Crk-like protein	CRKL_HUMAN	34 kDa	0	0	0	0	0	0	0	0	1	0
Unconventional myosin-IXa	MYO9A_HUMAN	293 kDa	0	0	0	1	0	0	0	0	0	0
Constitutive coactivator of peroxisome proliferator-activated receptor gamma	F120B_HUMAN	104 kDa	0	0	0	1	0	0	0	0	0	0
Epidermal growth factor receptor substrate 15	EPS15_HUMAN	99 kDa	0	0	0	1	0	0	0	0	0	0
Laminin subunit alpha-5	LAMA5_HUMAN	400 kDa	0	0	0	1	0	0	0	0	0	0
Uncharacterized protein C20orf194	CT194_HUMAN	132 kDa	0	0	0	1	0	0	0	0	0	0
Coiled-coil domain-containing protein 150	CC150_HUMAN	129 kDa	0	0	0	1	0	0	0	0	0	0
Homeobox protein Hox-A4	HXA4_HUMAN	34 kDa	0	0	0	1	0	0	0	0	0	0

Description	Accession	MW	Raw spectral counts									
			Frontal cortex	Cerebellum	Right ventricle	Mesentric lymph node	Liver	Pancreas	Proximal bile duct	Breast	Ovary	Clitoris
FERM and PDZ domain-containing protein 3	FRPD3_HUMAN	199 kDa	0	0	0	1	0	0	0	0	0	0
Centrosomal protein of 170 kDa protein B	C170B_HUMAN	172 kDa	0	0	0	1	0	0	0	0	0	0
Protein bicaudal D homolog 1	BICD1_HUMAN	111 kDa	0	0	0	1	0	0	0	0	0	0
Helicase SRCAP	SRCAP_HUMAN	344 kDa	0	0	0	1	0	0	0	0	0	0
WD repeat-containing protein 49	WDR49_HUMAN	79 kDa	0	0	0	1	0	0	0	0	0	0
Solute carrier family 15 member 2	S15A2_HUMAN	82 kDa	0	0	0	1	0	0	0	0	0	0
Collagen alpha-3(IV) chain	CO4A3_HUMAN	162 kDa	0	0	0	1	0	0	0	0	0	0
Solute carrier family 12 member 7	S12A7_HUMAN	119 kDa	0	0	0	1	0	0	0	0	0	0
Leucine-rich repeats and immunoglobulin-like domains protein 1	LRIG1_HUMAN	119 kDa	0	0	0	1	0	0	0	0	0	0
Prostaglandin E2 receptor EP4 subtype	PE2R4_HUMAN	53 kDa	0	0	0	1	0	0	0	0	0	0
Gamma-aminobutyric acid receptor subunit alpha-5	GBRA5_HUMAN	52 kDa	0	0	0	1	0	0	0	0	0	0
Protein HEATR9	HEAT9_HUMAN	66 kDa	0	0	0	1	0	0	0	0	0	0
Zinc finger protein 764	ZN764_HUMAN	45 kDa	0	0	0	1	0	0	0	0	0	0
DNA-binding protein RFX5	RFX5_HUMAN	65 kDa	0	0	0	1	0	0	0	0	0	0
Uncharacterized protein C16orf71	CP071_HUMAN	56 kDa	0	0	0	1	0	0	0	0	0	0
Sal-like protein 3	SALL3_HUMAN	135 kDa	0	0	0	1	0	0	0	0	0	0
Coagulation factor VII	FA7_HUMAN	52 kDa	0	0	0	1	0	0	0	0	0	0
Inactive phospholipase C-like protein 2	PLCL2_HUMAN	126 kDa	0	0	0	1	0	0	0	0	0	0
Aspartyl/asparaginyl beta-hydroxylase	ASPH_HUMAN	86 kDa	0	0	0	1	0	0	0	0	0	0
Acetyl-CoA carboxylase 1	ACACA_HUMAN	266 kDa	0	0	0	1	0	0	0	0	0	0
Lateral signaling target protein 2 homolog	LST2_HUMAN	96 kDa	0	0	0	1	0	0	0	0	0	0
Hormone-sensitive lipase	LIPS_HUMAN	117 kDa	0	0	0	1	0	0	0	0	0	0
Serine/threonine-protein kinase SMG1	SMG1_HUMAN	410 kDa	0	0	0	1	0	0	0	0	0	0
Alstrom syndrome protein 1	ALMS1_HUMAN	461 kDa	0	0	0	0	1	0	0	0	0	0
Biorientation of chromosomes in cell division protein 1-like 1	BD1L1_HUMAN	330 kDa	0	0	0	0	1	0	0	0	0	0
Laminin subunit gamma-2	LAMC2_HUMAN	131 kDa	0	0	0	0	1	0	0	0	0	0
KAT8 regulatory NSL complex subunit 1-like protein	KAL1L_HUMAN	112 kDa	0	0	0	0	1	0	0	0	0	0
ATP synthase subunit delta	ATPD_HUMAN	17 kDa	0	0	0	0	1	0	0	0	0	0
Stabilin-2	STAB2_HUMAN	277 kDa	0	0	0	0	1	0	0	0	0	0
Protein NPAT	NPAT_HUMAN	154 kDa	0	0	0	0	1	0	0	0	0	0
Tudor domain-containing protein 15	TDR15_HUMAN	222 kDa	0	0	0	0	1	0	0	0	0	0
RNA exonuclease 1 homolog	REXO1_HUMAN	132 kDa	0	0	0	0	1	0	0	0	0	0
Kynureninase	KYNU_HUMAN	52 kDa	0	0	0	0	1	0	0	0	0	0
Intraflagellar transport protein 172 homolog	IF172_HUMAN	198 kDa	0	0	0	0	1	0	0	0	0	0
Magnesium-dependent phosphatase 1	MGDP1_HUMAN	20 kDa	0	0	0	0	1	0	0	0	0	0
Dual specificity tyrosine-phosphorylation-regulated kinase 3	DYRK3_HUMAN	66 kDa	0	0	0	0	1	0	0	0	0	0
Leucine-rich repeat-containing protein 48	LRC48_HUMAN	61 kDa	0	0	0	0	1	0	0	0	0	0
Contactin-6	CNTN6_HUMAN	114 kDa	0	0	0	0	1	0	0	0	0	0
Secretogranin-1	SCG1_HUMAN	78 kDa	0	0	0	0	1	0	0	0	0	0
Mitochondrial enolase superfamily member 1	ENOF1_HUMAN	50 kDa	0	0	0	0	1	0	0	0	0	0
Dihydroorotate dehydrogenase (quinone)	PYRD_HUMAN	43 kDa	0	0	0	0	1	0	0	0	0	0
Unconventional myosin-Ib	MYO1B_HUMAN	132 kDa	0	0	0	0	1	0	0	0	0	0
Beta-2-microglobulin	B2MG_HUMAN	14 kDa	0	0	0	0	1	0	0	0	0	0
Solute carrier organic anion transporter family member 1B1	SO1B1_HUMAN	76 kDa	0	0	0	0	1	0	0	0	0	0
Alpha-2-macroglobulin receptor-associated protein	AMRP_HUMAN	41 kDa	0	0	0	0	1	0	0	0	0	0
Acyl-coenzyme A synthetase ACSM1	ACSM1_HUMAN	65 kDa	0	0	0	0	1	0	0	0	0	0
Tyrosine--tRNA ligase	SYYM_HUMAN	53 kDa	0	0	0	0	1	0	0	0	0	0
28S ribosomal protein S27	RT27_HUMAN	48 kDa	0	0	0	0	1	0	0	0	0	0
Lysophospholipid acyltransferase 5	MBOA5_HUMAN	56 kDa	0	0	0	0	1	0	0	0	0	0
Elongation factor Ts	EFTS_HUMAN	35 kDa	0	0	0	0	1	0	0	0	0	0
Hydroxyacid-oxoacid transhydrogenase	HOT_HUMAN	50 kDa	0	0	0	0	1	0	0	0	0	0
Aldo-keto reductase family 1 member C-like protein 1	AKCL1_HUMAN	15 kDa	0	0	0	0	1	0	0	0	0	0

Description	Accession	MW	Raw spectral counts									
			Frontal cortex	Cerebellum	Right ventricle	Mesentric lymph node	Liver	Pancreas	Proximal bile duct	Breast	Ovary	Clitoris
Kynurenine 3-monooxygenase	KMO_HUMAN	56 kDa	0	0	0	0	1	0	0	0	0	0
Glyoxalase domain-containing protein 5	GLOD5_HUMAN	18 kDa	0	0	0	0	1	0	0	0	0	0
Threonine synthase-like 1	THNS1_HUMAN	83 kDa	0	0	0	0	1	0	0	0	0	0
Sigma non-opioid intracellular receptor 1	SGMR1_HUMAN	25 kDa	0	0	0	0	1	0	0	0	0	0
Ubiquilin-3	UBQL3_HUMAN	71 kDa	0	0	0	0	1	0	0	0	0	0
Xylulose kinase	XYLB_HUMAN	58 kDa	0	0	0	0	1	0	0	0	0	0
UDP-glucuronosyltransferase 3A2	UD3A2_HUMAN	60 kDa	0	0	0	0	1	0	0	0	0	0
Cysteine sulfinic acid decarboxylase	CSAD_HUMAN	55 kDa	0	0	0	0	1	0	0	0	0	0
Dihydrofolate reductase	DYR_HUMAN	21 kDa	0	0	0	0	1	0	0	0	0	0
Cat eye syndrome critical region protein 5	CECR5_HUMAN	46 kDa	0	0	0	0	1	0	0	0	0	0
Sulfite oxidase	SUOX_HUMAN	60 kDa	0	0	0	0	1	0	0	0	0	0
Transcription factor SOX-6	SOX6_HUMAN	92 kDa	0	0	0	0	1	0	0	0	0	0
Diamine acetyltransferase 2	SAT2_HUMAN	19 kDa	0	0	0	0	1	0	0	0	0	0
Ectonucleoside triphosphate diphosphohydrolase 8	ENTP8_HUMAN	54 kDa	0	0	0	0	1	0	0	0	0	0
Mitochondrial peptide methionine sulfoxide reductase	MSRA_HUMAN	26 kDa	0	0	0	0	1	0	0	0	0	0
Nicotinamide N-methyltransferase	NNMT_HUMAN	30 kDa	0	0	0	0	1	0	0	0	0	0
Lanosterol 14-alpha demethylase	CP51A_HUMAN	57 kDa	0	0	0	0	1	0	0	0	0	0
Fucose mutarotase	FUCM_HUMAN	17 kDa	0	0	0	0	1	0	0	0	0	0
Glycine cleavage system H protein	GCSH_HUMAN	19 kDa	0	0	0	0	1	0	0	0	0	0
BH3-interacting domain death agonist	BID_HUMAN	22 kDa	0	0	0	0	1	0	0	0	0	0
Claudin-1	CLD1_HUMAN	23 kDa	0	0	0	0	1	0	0	0	0	0
UDP-glucuronic acid/UDP-N-acetylgalactosamine transporter	S35D1_HUMAN	39 kDa	0	0	0	0	1	0	0	0	0	0
Diacylglycerol kinase epsilon	DGKE_HUMAN	64 kDa	0	0	0	0	1	0	0	0	0	0
Mitochondrial dicarboxylate carrier	DIC_HUMAN	31 kDa	0	0	0	0	1	0	0	0	0	0
Putative transferase CAF17	CAF17_HUMAN	38 kDa	0	0	0	0	1	0	0	0	0	0
Cingulin	CING_HUMAN	136 kDa	0	0	0	0	1	0	0	0	0	0
Protein NOXP20	NXP20_HUMAN	61 kDa	0	0	0	0	1	0	0	0	0	0
Aspartoacylase	ACY2_HUMAN	36 kDa	0	0	0	0	1	0	0	0	0	0
Mitochondrial chaperone BCS1	BCS1_HUMAN	48 kDa	0	0	0	0	1	0	0	0	0	0
Nitrilase homolog 1	NIT1_HUMAN	36 kDa	0	0	0	0	1	0	0	0	0	0
UPF0598 protein C8orf82	CH082_HUMAN	24 kDa	0	0	0	0	1	0	0	0	0	0
39S ribosomal protein L13	RM13_HUMAN	21 kDa	0	0	0	0	1	0	0	0	0	0
NAD(P)H dehydrogenase [quinone] 1	NQO1_HUMAN	31 kDa	0	0	0	0	1	0	0	0	0	0
ATP-dependent Clp protease proteolytic subunit	CLPP_HUMAN	30 kDa	0	0	0	0	1	0	0	0	0	0
DnaJ homolog subfamily A member 3	DNJA3_HUMAN	52 kDa	0	0	0	0	1	0	0	0	0	0
Prostaglandin reductase 2	PTGR2_HUMAN	38 kDa	0	0	0	0	1	0	0	0	0	0
Acyl-coenzyme A thioesterase 1	ACOT1_HUMAN	46 kDa	0	0	0	0	1	0	0	0	0	0
Glycerol kinase	GLPK_HUMAN	61 kDa	0	0	0	0	1	0	0	0	0	0
Peptidyl-tRNA hydrolase ICT1	ICT1_HUMAN	24 kDa	0	0	0	0	1	0	0	0	0	0
Cardiomyopathy-associated protein 5	CMYA5_HUMAN	449 kDa	1	0	0	0	0	0	0	0	0	0
Microtubule-associated protein 1S	MAP1S_HUMAN	112 kDa	1	0	0	0	0	0	0	0	0	0
NACHT, LRR and PYD domains-containing protein 1	NALP1_HUMAN	166 kDa	1	0	0	0	0	0	0	0	0	0
Zinc finger protein 407	ZN407_HUMAN	247 kDa	1	0	0	0	0	0	0	0	0	0
Protein diaphanous homolog 3	DIAP3_HUMAN	137 kDa	1	0	0	0	0	0	0	0	0	0
CDK5 regulatory subunit-associated protein 2	CK5P2_HUMAN	215 kDa	1	0	0	0	0	0	0	0	0	0
Protocadherin-7	PCDH7_HUMAN	116 kDa	1	0	0	0	0	0	0	0	0	0
Exocyst complex component 4	EXOC4_HUMAN	111 kDa	1	0	0	0	0	0	0	0	0	0
Serine/threonine-protein kinase TAO1	TAOK1_HUMAN	116 kDa	1	0	0	0	0	0	0	0	0	0
Disks large homolog 3	DLG3_HUMAN	90 kDa	1	0	0	0	0	0	0	0	0	0
SPARC-like protein 1	SPRL1_HUMAN	75 kDa	1	0	0	0	0	0	0	0	0	0
Inverted formin-2	INF2_HUMAN	136 kDa	1	0	0	0	0	0	0	0	0	0
GRIP1-associated protein 1	GRAP1_HUMAN	96 kDa	1	0	0	0	0	0	0	0	0	0

Description	Accession	MW	Raw spectral counts									
			Frontal cortex	Cerebellum	Right ventricle	Mesentric lymph node	Liver	Pancreas	Proximal bile duct	Breast	Ovary	Clitoris
Protein phosphatase 1 regulatory subunit 21	PPR21_HUMAN	88 kDa	1	0	0	0	0	0	0	0	0	0
ProSAAS	PCSK1_HUMAN	27 kDa	1	0	0	0	0	0	0	0	0	0
E3 ubiquitin-protein ligase HECTD3	HECD3_HUMAN	97 kDa	1	0	0	0	0	0	0	0	0	0
Matrix metalloproteinase-16	MMP16_HUMAN	70 kDa	1	0	0	0	0	0	0	0	0	0
Immunoglobulin superfamily member 21	IGS21_HUMAN	52 kDa	1	0	0	0	0	0	0	0	0	0
Intercellular adhesion molecule 5	ICAM5_HUMAN	97 kDa	1	0	0	0	0	0	0	0	0	0
Cullin-associated NEDD8-dissociated protein 2	CAND2_HUMAN	135 kDa	1	0	0	0	0	0	0	0	0	0
Cullin-2	CUL2_HUMAN	87 kDa	1	0	0	0	0	0	0	0	0	0
Anaphase-promoting complex subunit 4	APC4_HUMAN	92 kDa	1	0	0	0	0	0	0	0	0	0
Transportin-2	TNPO2_HUMAN	101 kDa	1	0	0	0	0	0	0	0	0	0
Phosphatidylinositol 5-phosphate 4-kinase type-2 alpha	PI42A_HUMAN	46 kDa	1	0	0	0	0	0	0	0	0	0
Arfaptin-2	ARFP2_HUMAN	38 kDa	1	0	0	0	0	0	0	0	0	0
Ubiquitin carboxyl-terminal hydrolase 11	UBP11_HUMAN	110 kDa	1	0	0	0	0	0	0	0	0	0
Amyloid beta A4 protein	A4_HUMAN	87 kDa	1	0	0	0	0	0	0	0	0	0
Drebrin-like protein	DBNL_HUMAN	48 kDa	1	0	0	0	0	0	0	0	0	0
Calcium/calmodulin-dependent 3',5'-cyclic nucleotide phosphodiesterase 1B	PDE1B_HUMAN	61 kDa	1	0	0	0	0	0	0	0	0	0
OX-2 membrane glycoprotein	OX2G_HUMAN	31 kDa	1	0	0	0	0	0	0	0	0	0
Rap1 GTPase-activating protein 1	RPGP1_HUMAN	73 kDa	1	0	0	0	0	0	0	0	0	0
Cyclin-Y	CCNY_HUMAN	39 kDa	1	0	0	0	0	0	0	0	0	0
Prefoldin subunit 4	PFD4_HUMAN	15 kDa	1	0	0	0	0	0	0	0	0	0
HERV-K_11q22.1 provirus ancestral Pol protein	POK17_HUMAN	107 kDa	1	0	0	0	0	0	0	0	0	0
Desmoglein-4	DSG4_HUMAN	114 kDa	1	0	0	0	0	0	0	0	0	0
Prefoldin subunit 6	PFD6_HUMAN	15 kDa	1	0	0	0	0	0	0	0	0	0
Dual specificity protein phosphatase 14	DUS14_HUMAN	22 kDa	1	0	0	0	0	0	0	0	0	0
Liprin-alpha-1	LIPA1_HUMAN	136 kDa	1	0	0	0	0	0	0	0	0	0
Exportin-7	XPO7_HUMAN	124 kDa	1	0	0	0	0	0	0	0	0	0
Protocadherin-12	PCD12_HUMAN	129 kDa	1	0	0	0	0	0	0	0	0	0
Latrophilin-3	LPHN3_HUMAN	162 kDa	1	0	0	0	0	0	0	0	0	0
Microtubule cross-linking factor 1	MTCL1_HUMAN	210 kDa	1	0	0	0	0	0	0	0	0	0
Eukaryotic translation initiation factor 4 gamma 2	IF4G2_HUMAN	102 kDa	1	0	0	0	0	0	0	0	0	0
Contactin-2	CNTN2_HUMAN	113 kDa	1	0	0	0	0	0	0	0	0	0
WD repeat-containing protein 37	WDR37_HUMAN	55 kDa	1	0	0	0	0	0	0	0	0	0
Claudin-11	CLD11_HUMAN	22 kDa	1	0	0	0	0	0	0	0	0	0
Oligodendrocyte-myelin glycoprotein	OMGP_HUMAN	50 kDa	1	0	0	0	0	0	0	0	0	0
Uncharacterized protein C10orf35	CJ035_HUMAN	13 kDa	1	0	0	0	0	0	0	0	0	0
ELKS/Rab6-interacting/CAST family member 1	RB6I2_HUMAN	128 kDa	1	0	0	0	0	0	0	0	0	0
Protein unc-13 homolog A	UN13A_HUMAN	193 kDa	1	0	0	0	0	0	0	0	0	0
UDP-N-acetylglucosamine--peptide N-acetylglucosaminyltransferase 110 kDa subunit	OGT1_HUMAN	117 kDa	1	0	0	0	0	0	0	0	0	0
Glutamate decarboxylase 2	DCE2_HUMAN	65 kDa	1	0	0	0	0	0	0	0	0	0
Syntaxin-6	STX6_HUMAN	29 kDa	1	0	0	0	0	0	0	0	0	0
Mitochondrial uncoupling protein 4	UCP4_HUMAN	36 kDa	1	0	0	0	0	0	0	0	0	0
Histidine triad nucleotide-binding protein 3	HINT3_HUMAN	20 kDa	1	0	0	0	0	0	0	0	0	0
SLIT-ROBO Rho GTPase-activating protein 2	SRGP2_HUMAN	121 kDa	1	0	0	0	0	0	0	0	0	0
Protein kinase C gamma type	KPCG_HUMAN	78 kDa	1	0	0	0	0	0	0	0	0	0
AP2-associated protein kinase 1	AAK1_HUMAN	104 kDa	1	0	0	0	0	0	0	0	0	0
Probable E3 ubiquitin-protein ligase HERC4	HERC4_HUMAN	119 kDa	1	0	0	0	0	0	0	0	0	0
E2/E3 hybrid ubiquitin-protein ligase UBE2O	UBE2O_HUMAN	141 kDa	1	0	0	0	0	0	0	0	0	0
Serine/threonine-protein phosphatase 5	PPP5_HUMAN	57 kDa	1	0	0	0	0	0	0	0	0	0
Dual specificity mitogen-activated protein kinase kinase 4	MP2K4_HUMAN	44 kDa	1	0	0	0	0	0	0	0	0	0
Reticulocalbin-2	RCN2_HUMAN	37 kDa	1	0	0	0	0	0	0	0	0	0
Mammalian ependymin-related protein 1	EPDR1_HUMAN	25 kDa	1	0	0	0	0	0	0	0	0	0
Rabphilin-3A	RP3A_HUMAN	77 kDa	1	0	0	0	0	0	0	0	0	0

Description	Accession	MW	Raw spectral counts									
			Frontal cortex	Cerebellum	Right ventricle	Mesentric lymph node	Liver	Pancreas	Proximal bile duct	Breast	Ovary	Clitoris
MAGUK p55 subfamily member 6	MPP6_HUMAN	61 kDa	1	0	0	0	0	0	0	0	0	0
Protein SCAI	SCAI_HUMAN	70 kDa	1	0	0	0	0	0	0	0	0	0
Ganglioside-induced differentiation-associated protein 1-like 1	GD1L1_HUMAN	42 kDa	1	0	0	0	0	0	0	0	0	0
Unconventional myosin-Va	MYO5A_HUMAN	215 kDa	1	0	0	0	0	0	0	0	0	0
Rho GTPase-activating protein 44	RHG44_HUMAN	89 kDa	1	0	0	0	0	0	0	0	0	0
Ubiquitin-like modifier-activating enzyme 6	UBA6_HUMAN	118 kDa	1	0	0	0	0	0	0	0	0	0
Transcription elongation regulator 1	TCRG1_HUMAN	124 kDa	1	0	0	0	0	0	0	0	0	0
Phosphoribosylformylglycinamidine synthase	PUR4_HUMAN	145 kDa	1	0	0	0	0	0	0	0	0	0
Adapter molecule crk	CRK_HUMAN	34 kDa	1	0	0	0	0	0	0	0	0	0
Tyrosine-protein phosphatase non-receptor type 11	PTN11_HUMAN	68 kDa	1	0	0	0	0	0	0	0	0	0
Prostaglandin E synthase 3	TEBP_HUMAN	19 kDa	1	0	0	0	0	0	0	0	0	0
E3 ubiquitin-protein ligase HUWE1	HUWE1_HUMAN	482 kDa	1	0	0	0	0	0	0	0	0	0
Peptidyl-prolyl cis-trans isomerase-like 1	PPIL1_HUMAN	18 kDa	1	0	0	0	0	0	0	0	0	0
CAP-Gly domain-containing linker protein 1	CLIP1_HUMAN	162 kDa	1	0	0	0	0	0	0	0	0	0
Dedicator of cytokinesis protein 9	DOCK9_HUMAN	236 kDa	1	0	0	0	0	0	0	0	0	0
Nuclear protein localization protein 4 homolog	NPL4_HUMAN	68 kDa	1	0	0	0	0	0	0	0	0	0
Phosphorylase b kinase regulatory subunit alpha, skeletal muscle isoform	KPB1_HUMAN	137 kDa	1	0	0	0	0	0	0	0	0	0
Hexaprenyldihydroxybenzoate methyltransferase	COQ3_HUMAN	41 kDa	1	0	0	0	0	0	0	0	0	0
Phosphofurin acidic cluster sorting protein 1	PACS1_HUMAN	105 kDa	1	0	0	0	0	0	0	0	0	0
Guanine nucleotide-binding protein G(I)/G(S)/G(O) subunit gamma-7	GBG7_HUMAN	8 kDa	1	0	0	0	0	0	0	0	0	0
5'-AMP-activated protein kinase catalytic subunit alpha-1	AAPK1_HUMAN	64 kDa	1	0	0	0	0	0	0	0	0	0
UV excision repair protein RAD23 homolog A	RD23A_HUMAN	40 kDa	1	0	0	0	0	0	0	0	0	0
Collagen alpha-6(VI) chain	CO6A6_HUMAN	247 kDa	1	0	0	0	0	0	0	0	0	0
Thioredoxin	THIOM_HUMAN	18 kDa	1	0	0	0	0	0	0	0	0	0
ATPase family AAA domain-containing protein 1	ATAD1_HUMAN	41 kDa	1	0	0	0	0	0	0	0	0	0
Transcription termination factor 3	MTEF3_HUMAN	48 kDa	0	0	0	0	0	0	0	0	0	2
Myosin light chain 1/3, skeletal muscle isoform	MYL1_HUMAN	21 kDa	0	0	0	0	0	0	0	0	0	2
L-lactate dehydrogenase C chain	LDHC_HUMAN	36 kDa	0	0	0	0	0	0	0	0	0	2
Erlin-1	ERLN1_HUMAN	39 kDa	0	0	0	0	0	0	0	0	0	2
Inter-alpha-trypsin inhibitor heavy chain H3	ITIH3_HUMAN	100 kDa	0	0	0	0	0	0	0	0	0	2
Desmocollin-3	DSC3_HUMAN	100 kDa	0	0	0	0	0	0	0	0	0	2
Quinone oxidoreductase PIG3	QORX_HUMAN	36 kDa	0	0	0	0	0	0	0	0	0	2
Transforming growth factor-beta-induced protein ig-h3	BGH3_HUMAN	75 kDa	0	0	0	0	0	0	0	0	0	2
Splicing factor 3B subunit 3	SF3B3_HUMAN	136 kDa	0	1	0	0	0	0	0	0	0	1
Unconventional myosin-If	MYO1F_HUMAN	125 kDa	0	0	0	0	0	1	0	0	0	1
Zinc-alpha-2-glycoprotein	ZA2G_HUMAN	34 kDa	0	0	0	0	0	1	0	0	0	1
Guanylate-binding protein 7	GBP7_HUMAN	73 kDa	0	0	0	0	0	0	1	0	0	1
Hermansky-Pudlak syndrome 3 protein	HPS3_HUMAN	114 kDa	0	0	0	0	0	0	0	0	1	1
Coronin-1B	COR1B_HUMAN	54 kDa	0	0	0	0	0	0	0	0	1	1
Programmed cell death protein 10	PDC10_HUMAN	25 kDa	0	0	0	0	0	0	0	0	1	1
Calcium-binding mitochondrial carrier protein SCaMC-1	SCMC1_HUMAN	53 kDa	0	0	0	0	0	0	0	0	1	1
Glutathione reductase	GSHR_HUMAN	56 kDa	0	0	0	0	0	0	0	0	1	1
Parathymosin	PTMS_HUMAN	12 kDa	0	0	0	0	1	0	0	0	0	1
Tetratricopeptide repeat protein 7B	TTC7B_HUMAN	94 kDa	0	2	0	0	0	0	0	0	0	0
Rho GTPase-activating protein 26	RHG26_HUMAN	92 kDa	0	2	0	0	0	0	0	0	0	0
Homer protein homolog 3	HOME3_HUMAN	40 kDa	0	2	0	0	0	0	0	0	0	0
Vesicle-associated membrane protein 1	VAMP1_HUMAN	13 kDa	0	2	0	0	0	0	0	0	0	0
Hepatocyte cell adhesion molecule	HECAM_HUMAN	46 kDa	0	2	0	0	0	0	0	0	0	0
Septin-6	SEPT6_HUMAN	50 kDa	0	2	0	0	0	0	0	0	0	0
Dual specificity mitogen-activated protein kinase kinase 2	MP2K2_HUMAN	44 kDa	0	2	0	0	0	0	0	0	0	0
Melanoma inhibitory activity protein 3	MIA3_HUMAN	214 kDa	0	1	0	0	0	1	0	0	0	0
Splicing factor U2AF 65 kDa subunit	U2AF2_HUMAN	54 kDa	0	1	0	0	0	1	0	0	0	0

Description	Accession	MW	Raw spectral counts									
			Frontal cortex	Cerebellum	Right ventricle	Mesentric lymph node	Liver	Pancreas	Proximal bile duct	Breast	Ovary	Clitoris
Protocadherin Fat 1	FAT1_HUMAN	506 kDa	0	1	0	0	0	0	1	0	0	0
Apoptosis inhibitor 5	API5_HUMAN	59 kDa	0	1	0	0	0	0	0	0	1	0
Sorting nexin-4	SNX4_HUMAN	52 kDa	0	1	0	0	0	0	0	0	1	0
WD repeat-containing protein 7	WDR7_HUMAN	164 kDa	0	1	0	0	0	0	0	0	1	0
GMP synthase [glutamine-hydrolyzing]	GUAA_HUMAN	77 kDa	0	1	0	0	0	0	0	0	1	0
Rho-related GTP-binding protein RhoG	RHOG_HUMAN	21 kDa	0	1	0	0	0	0	0	0	1	0
Casein kinase II subunit beta	CSK2B_HUMAN	25 kDa	0	1	0	0	0	0	0	0	1	0
Trafficking protein particle complex subunit 3	TPPC3_HUMAN	20 kDa	0	1	0	0	0	0	0	0	1	0
Splicing factor 3A subunit 1	SF3A1_HUMAN	89 kDa	0	1	0	0	0	0	0	0	1	0
Eukaryotic translation initiation factor 3 subunit L	EIF3L_HUMAN	67 kDa	0	1	0	0	0	0	0	0	1	0
Coactosin-like protein	COTL1_HUMAN	16 kDa	0	1	0	0	1	0	0	0	0	0
Astrotactin-1	ASTN1_HUMAN	145 kDa	1	1	0	0	0	0	0	0	0	0
Type I inositol 3,4-bisphosphate 4-phosphatase	INP4A_HUMAN	110 kDa	1	1	0	0	0	0	0	0	0	0
Pyridoxal kinase	PDXK_HUMAN	35 kDa	1	1	0	0	0	0	0	0	0	0
V-type proton ATPase subunit d 2	VA0D2_HUMAN	40 kDa	1	1	0	0	0	0	0	0	0	0
Cyclin-dependent kinase 5	CDK5_HUMAN	33 kDa	1	1	0	0	0	0	0	0	0	0
ELAV-like protein 4	ELAV4_HUMAN	42 kDa	1	1	0	0	0	0	0	0	0	0
Putative tyrosine-protein phosphatase auxilin	AUXI_HUMAN	100 kDa	1	1	0	0	0	0	0	0	0	0
Caskin-1	CSKI1_HUMAN	150 kDa	1	1	0	0	0	0	0	0	0	0
Ras-related protein Rab-4B	RAB4B_HUMAN	24 kDa	1	1	0	0	0	0	0	0	0	0
Histone H3.1	H31_HUMAN	15 kDa	1	1	0	0	0	0	0	0	0	0
Acyl-protein thioesterase 2	LYPA2_HUMAN	25 kDa	1	1	0	0	0	0	0	0	0	0
Sorting nexin-12	SNX12_HUMAN	20 kDa	1	1	0	0	0	0	0	0	0	0
Flotillin-1	FLOT1_HUMAN	47 kDa	1	1	0	0	0	0	0	0	0	0
Basic leucine zipper and W2 domain-containing protein 2	BZW2_HUMAN	48 kDa	0	0	2	0	0	0	0	0	0	0
Ubiquinone biosynthesis monooxygenase COQ6	COQ6_HUMAN	51 kDa	0	0	2	0	0	0	0	0	0	0
Caseinolytic peptidase B protein homolog	CLPB_HUMAN	79 kDa	0	0	2	0	0	0	0	0	0	0
O-acetyl-ADP-ribose deacetylase MACROD1	MACD1_HUMAN	36 kDa	0	0	2	0	0	0	0	0	0	0
Protein PET100 homolog	PT100_HUMAN	9 kDa	0	0	2	0	0	0	0	0	0	0
Cytochrome c oxidase subunit 7C	COX7C_HUMAN	7 kDa	0	0	2	0	0	0	0	0	0	0
Presequence protease	PREP_HUMAN	117 kDa	0	0	2	0	0	0	0	0	0	0
Alpha-sarcoglycan	SGCA_HUMAN	43 kDa	0	0	2	0	0	0	0	0	0	0
NADH dehydrogenase [ubiquinone] 1 alpha subcomplex subunit 12	NDUAC_HUMAN	17 kDa	0	0	2	0	0	0	0	0	0	0
Nexilin	NEXN_HUMAN	81 kDa	0	0	2	0	0	0	0	0	0	0
39S ribosomal protein L17	RM17_HUMAN	20 kDa	0	0	2	0	0	0	0	0	0	0
Heat shock protein beta-7	HSPB7_HUMAN	19 kDa	0	0	2	0	0	0	0	0	0	0
Ubiquinone biosynthesis protein COQ7 homolog	COQ7_HUMAN	24 kDa	0	0	2	0	0	0	0	0	0	0
Mitochondrial import inner membrane translocase subunit TIM50	TIM50_HUMAN	40 kDa	0	0	2	0	0	0	0	0	0	0
Four and a half LIM domains protein 2	FHL2_HUMAN	32 kDa	0	0	2	0	0	0	0	0	0	0
Glycogenin-1	GLYG_HUMAN	39 kDa	0	0	2	0	0	0	0	0	0	0
Fat storage-inducing transmembrane protein 2	FITM2_HUMAN	30 kDa	0	0	2	0	0	0	0	0	0	0
RNA-binding motif protein, X-linked-like-3	RMXL3_HUMAN	115 kDa	0	0	1	0	0	1	0	0	0	0
Dynein heavy chain 3, axonemal	DYH3_HUMAN	471 kDa	0	0	1	0	0	1	0	0	0	0
Tax1-binding protein 3	TX1B3_HUMAN	14 kDa	0	0	1	0	0	0	0	0	1	0
UPF0587 protein C1orf123	CA123_HUMAN	18 kDa	0	0	1	0	0	0	0	0	1	0
COP9 signalosome complex subunit 7a	CSN7A_HUMAN	30 kDa	0	0	1	0	0	0	0	0	1	0
Mitochondrial-processing peptidase subunit beta	MPPB_HUMAN	54 kDa	0	0	1	0	0	0	0	0	1	0
NFU1 iron-sulfur cluster scaffold homolog	NFU1_HUMAN	28 kDa	0	0	1	1	0	0	0	0	0	0
HLA class II histocompatibility antigen, DR beta 5 chain	DRB5_HUMAN	30 kDa	0	0	1	0	1	0	0	0	0	0
[3-methyl-2-oxobutanoate dehydrogenase [lipoamide]] kinase	BCKD_HUMAN	46 kDa	0	0	1	0	1	0	0	0	0	0
Coiled-coil domain-containing protein 18	CCD18_HUMAN	169 kDa	0	0	1	0	1	0	0	0	0	0
UV excision repair protein RAD23 homolog B	RD23B_HUMAN	43 kDa	0	0	1	0	1	0	0	0	0	0

Description	Accession	MW	Raw spectral counts									
			Frontal cortex	Cerebellum	Right ventricle	Mesentric lymph node	Liver	Pancreas	Proximal bile duct	Breast	Ovary	Clitoris
Malonyl-CoA decarboxylase	DCMC_HUMAN	55 kDa	0	0	1	0	1	0	0	0	0	0
Mitochondrial pyruvate carrier 1	MPC1_HUMAN	12 kDa	0	0	1	0	1	0	0	0	0	0
Enoyl-CoA hydratase domain-containing protein 2	ECHD2_HUMAN	31 kDa	0	0	1	0	1	0	0	0	0	0
Complement component 1 Q subcomponent-binding protein	C1QBP_HUMAN	31 kDa	0	0	1	0	1	0	0	0	0	0
Cullin-1	CUL1_HUMAN	90 kDa	1	0	1	0	0	0	0	0	0	0
NAD-dependent protein deacetylase sirtuin-3	SIR3_HUMAN	44 kDa	1	0	1	0	0	0	0	0	0	0
Golgin subfamily A member 2	GOGA2_HUMAN	113 kDa	0	0	0	0	0	2	0	0	0	0
Keratin, type II cytoskeletal 78	K2C78_HUMAN	57 kDa	0	0	0	0	0	2	0	0	0	0
Fructose-1,6-bisphosphatase isozyme 2	F16P2_HUMAN	37 kDa	0	0	0	0	0	2	0	0	0	0
Hornerin	HORN_HUMAN	282 kDa	0	0	0	0	0	2	0	0	0	0
Protoporphyrinogen oxidase	PPOX_HUMAN	51 kDa	0	0	0	0	0	2	0	0	0	0
Galectin-12	LEG12_HUMAN	38 kDa	0	0	0	0	0	2	0	0	0	0
ER lumen protein-retaining receptor 3	ERD23_HUMAN	25 kDa	0	0	0	0	0	2	0	0	0	0
Ras-related protein Rab-33B	RB33B_HUMAN	26 kDa	0	0	0	0	0	2	0	0	0	0
Leucine zipper transcription factor-like protein 1	LZTL1_HUMAN	35 kDa	0	0	0	0	0	2	0	0	0	0
Interferon-induced, double-stranded RNA-activated protein kinase	E2AK2_HUMAN	62 kDa	0	0	0	0	0	2	0	0	0	0
ATP-binding cassette sub-family E member 1	ABCE1_HUMAN	67 kDa	0	0	0	0	0	2	0	0	0	0
Transmembrane protein 97	TMM97_HUMAN	21 kDa	0	0	0	0	0	2	0	0	0	0
Dolichyl-phosphate beta-glucosyltransferase	ALG5_HUMAN	37 kDa	0	0	0	0	0	2	0	0	0	0
LDLR chaperone MESD	MESD_HUMAN	26 kDa	0	0	0	0	0	2	0	0	0	0
Eukaryotic translation initiation factor 3 subunit K	EIF3K_HUMAN	25 kDa	0	0	0	0	0	2	0	0	0	0
60S ribosomal protein L36a	RL36A_HUMAN	12 kDa	0	0	0	0	0	2	0	0	0	0
Kallikrein-1	KLK1_HUMAN	29 kDa	0	0	0	0	0	2	0	0	0	0
Stromal cell-derived factor 2-like protein 1	SDF2L_HUMAN	24 kDa	0	0	0	0	0	2	0	0	0	0
Cysteine--tRNA ligase, cytoplasmic	SYCC_HUMAN	85 kDa	0	0	0	0	0	2	0	0	0	0
Protein transport protein Sec61 subunit beta	SC61B_HUMAN	10 kDa	0	0	0	0	0	2	0	0	0	0
PCTP-like protein	PCTL_HUMAN	33 kDa	0	0	0	0	0	2	0	0	0	0
Chymotrypsinogen B	CTRB1_HUMAN	28 kDa	0	0	0	0	0	2	0	0	0	0
ER lumen protein-retaining receptor 1	ERD21_HUMAN	25 kDa	0	0	0	0	0	2	0	0	0	0
28S ribosomal protein S15	RT15_HUMAN	30 kDa	0	0	0	0	0	2	0	0	0	0
Transmembrane emp24 domain-containing protein 6	TMED6_HUMAN	28 kDa	0	0	0	0	0	2	0	0	0	0
Keratinocyte-associated protein 2	KTAP2_HUMAN	15 kDa	0	0	0	0	0	2	0	0	0	0
Pancreatic lipase-related protein 2	LIPR2_HUMAN	52 kDa	0	0	0	0	0	2	0	0	0	0
Eukaryotic translation initiation factor 3 subunit D	EIF3D_HUMAN	64 kDa	0	0	0	0	0	2	0	0	0	0
UPF0556 protein C19orf10	CS010_HUMAN	19 kDa	0	0	0	0	0	2	0	0	0	0
Eukaryotic translation elongation factor 1 epsilon-1	MCA3_HUMAN	20 kDa	0	0	0	0	0	2	0	0	0	0
Peptidyl-prolyl cis-trans isomerase D	PPID_HUMAN	41 kDa	0	0	0	0	0	2	0	0	0	0
Eukaryotic translation initiation factor 3 subunit G	EIF3G_HUMAN	36 kDa	0	0	0	0	0	2	0	0	0	0
Protein SEC13 homolog	SEC13_HUMAN	36 kDa	0	0	0	0	0	2	0	0	0	0
Golgin subfamily B member 1	GOGB1_HUMAN	376 kDa	0	0	0	0	0	2	0	0	0	0
28S ribosomal protein S22	RT22_HUMAN	41 kDa	0	0	0	0	0	2	0	0	0	0
Vacuolar protein-sorting-associated protein 25	VPS25_HUMAN	21 kDa	0	0	0	0	0	2	0	0	0	0
Protein transport protein Sec24D	SC24D_HUMAN	113 kDa	0	0	0	0	0	2	0	0	0	0
E3 UFM1-protein ligase 1	UFL1_HUMAN	90 kDa	0	0	0	0	0	2	0	0	0	0
Phosphotriesterase-related protein	PTER_HUMAN	39 kDa	0	0	0	0	0	1	0	0	1	0
Clathrin interactor 1	EPN4_HUMAN	68 kDa	0	0	0	0	0	1	0	0	1	0
Eukaryotic translation initiation factor 3 subunit M	EIF3M_HUMAN	43 kDa	0	0	0	0	0	1	0	0	1	0
Putative deoxyribose-phosphate aldolase	DEOC_HUMAN	35 kDa	0	0	0	0	0	1	0	0	1	0
Transcription elongation factor A protein 1	TCEA1_HUMAN	34 kDa	0	0	0	0	0	1	0	0	1	0
Histidine--tRNA ligase, cytoplasmic	SYHC_HUMAN	57 kDa	0	0	0	0	0	1	0	0	1	0
Tight junction protein ZO-2	ZO2_HUMAN	134 kDa	0	0	0	0	0	1	0	0	1	0
Acidic leucine-rich nuclear phosphoprotein 32 family member E	AN32E_HUMAN	31 kDa	0	0	0	0	0	1	0	0	1	0

Description	Accession	MW	Raw spectral counts									
			Frontal cortex	Cerebellum	Right ventricle	Mesentric lymph node	Liver	Pancreas	Proximal bile duct	Breast	Ovary	Clitoris
26S proteasome non-ATPase regulatory subunit 6	PSMD6_HUMAN	46 kDa	0	0	0	0	0	1	0	0	1	0
Vacuolar protein sorting-associated protein 26A	VP26A_HUMAN	38 kDa	0	0	0	0	0	1	0	0	1	0
Charged multivesicular body protein 4b	CHM4B_HUMAN	25 kDa	0	0	0	0	0	1	0	0	1	0
Transcription factor BTF3	BTF3_HUMAN	22 kDa	0	0	0	0	0	1	0	0	1	0
E3 ubiquitin-protein ligase TRIM23	TRI23_HUMAN	64 kDa	0	0	0	0	1	1	0	0	0	0
CDGSH iron-sulfur domain-containing protein 2	CISD2_HUMAN	15 kDa	0	0	0	0	1	1	0	0	0	0
Peptidyl-tRNA hydrolase 2	PTH2_HUMAN	19 kDa	0	0	0	0	1	1	0	0	0	0
Acetyl-coenzyme A transporter 1	ACATN_HUMAN	61 kDa	0	0	0	0	1	1	0	0	0	0
Signal recognition particle 54 kDa protein	SRP54_HUMAN	56 kDa	0	0	0	0	1	1	0	0	0	0
Transcription factor BTF3 homolog 4	BT3L4_HUMAN	17 kDa	0	0	0	0	1	1	0	0	0	0
Nucleolar and coiled-body phosphoprotein 1	NOLC1_HUMAN	74 kDa	1	0	0	0	0	1	0	0	0	0
Importin-9	IPO9_HUMAN	116 kDa	1	0	0	0	0	1	0	0	0	0
Charged multivesicular body protein 5	CHMP5_HUMAN	25 kDa	1	0	0	0	0	1	0	0	0	0
Carboxypeptidase E	CBPE_HUMAN	53 kDa	1	0	0	0	0	1	0	0	0	0
L-aminoadipate-semialdehyde dehydrogenase-phosphopantetheinyl transferase	ADPPT_HUMAN	36 kDa	1	0	0	0	0	1	0	0	0	0
Tryptophan--tRNA ligase, cytoplasmic	SYWC_HUMAN	53 kDa	1	0	0	0	0	1	0	0	0	0
Eukaryotic translation initiation factor 4 gamma 1	IF4G1_HUMAN	175 kDa	1	0	0	0	0	1	0	0	0	0
Phosphatidylinositol 3,4,5-trisphosphate 5-phosphatase 1	SHIP1_HUMAN	133 kDa	0	0	0	0	0	0	2	0	0	0
Sorting nexin-20	SNX20_HUMAN	36 kDa	0	0	0	0	0	0	2	0	0	0
Leiomodin-1	LMOD1_HUMAN	67 kDa	0	0	0	0	0	0	2	0	0	0
Collagen alpha-1(XXVIII) chain	COSA1_HUMAN	117 kDa	0	0	0	0	0	0	2	0	0	0
Palladin	PALLD_HUMAN	151 kDa	0	0	0	0	0	0	2	0	0	0
Sorbin and SH3 domain-containing protein 1	SRBS1_HUMAN	143 kDa	0	0	0	0	0	0	2	0	0	0
Leucine-rich repeat-containing G-protein coupled receptor 4	LGR4_HUMAN	104 kDa	0	0	0	0	0	0	1	0	1	0
Integrin alpha-V	ITAV_HUMAN	116 kDa	0	0	0	0	0	0	1	0	1	0
Protein S100-A6	S10A6_HUMAN	10 kDa	0	0	0	1	0	0	1	0	0	0
Putative cytochrome c oxidase subunit 7A3	COX7S_HUMAN	12 kDa	0	0	0	0	1	0	1	0	0	0
Ribosomal L1 domain-containing protein 1	RL1D1_HUMAN	55 kDa	0	0	0	0	0	0	0	0	2	0
Gem-associated protein 2	GEMI2_HUMAN	32 kDa	0	0	0	0	0	0	0	0	2	0
Vinexin	VINEX_HUMAN	75 kDa	0	0	0	0	0	0	0	0	2	0
Adipocyte enhancer-binding protein 1	AEBP1_HUMAN	131 kDa	0	0	0	0	0	0	0	0	2	0
Sorting nexin-5	SNX5_HUMAN	47 kDa	0	0	0	0	0	0	0	0	2	0
Protein AMBP	AMBP_HUMAN	39 kDa	0	0	0	0	0	0	0	0	2	0
Insulin-like growth factor 2 mRNA-binding protein 3	IF2B3_HUMAN	64 kDa	0	0	0	0	0	0	0	0	2	0
E3 ubiquitin/ISG15 ligase TRIM25	TRI25_HUMAN	71 kDa	0	0	0	0	0	0	0	0	2	0
Torsin-1A-interacting protein 1	TOIP1_HUMAN	66 kDa	0	0	0	0	0	0	0	0	2	0
KDEL motif-containing protein 2	KDEL2_HUMAN	59 kDa	0	0	0	0	0	0	0	0	2	0
Protein canopy homolog 4	CNPY4_HUMAN	28 kDa	0	0	0	0	0	0	0	0	2	0
Neudesin	NENF_HUMAN	19 kDa	0	0	0	0	0	0	0	0	2	0
Protein Hikeshi	HIKES_HUMAN	22 kDa	0	0	0	0	0	0	0	0	2	0
Protein DEK	DEK_HUMAN	43 kDa	0	0	0	0	0	0	0	0	2	0
Sorting nexin-2	SNX2_HUMAN	58 kDa	0	0	0	0	0	0	0	0	2	0
Flotillin-2	FLOT2_HUMAN	47 kDa	0	0	0	0	0	0	0	0	2	0
RNA-binding protein 39	RBM39_HUMAN	59 kDa	0	0	0	0	0	0	0	0	2	0
UPF0553 protein C9orf64	CI064_HUMAN	39 kDa	0	0	0	0	0	0	0	0	2	0
Vitronectin	VTNC_HUMAN	54 kDa	0	0	0	0	0	0	0	0	2	0
DnaJ homolog subfamily C member 8	DNJC8_HUMAN	30 kDa	0	0	0	0	0	0	0	0	2	0
Reticulocalbin-1	RCN1_HUMAN	39 kDa	0	0	0	0	0	0	0	0	2	0
Ras-related protein Rab-21	RAB21_HUMAN	24 kDa	0	0	0	0	0	0	0	0	2	0
Collagen alpha-2(I) chain	CO1A2_HUMAN	129 kDa	0	0	0	0	0	0	0	0	2	0
H/ACA ribonucleoprotein complex subunit 2	NHP2_HUMAN	17 kDa	0	0	0	0	0	0	0	0	2	0
Nucleoprotein TPR	TPR_HUMAN	267 kDa	0	0	0	0	0	0	0	0	2	0

Description	Accession	MW	Raw spectral counts									
			Frontal cortex	Cerebellum	Right ventricle	Mesentric lymph node	Liver	Pancreas	Proximal bile duct	Breast	Ovary	Clitoris
SUMO-activating enzyme subunit 2	SAE2_HUMAN	71 kDa	0	0	0	0	0	0	0	0	2	0
Regulator of chromosome condensation	RCC1_HUMAN	45 kDa	0	0	0	0	0	0	0	0	2	0
Developmentally-regulated GTP-binding protein 1	DRG1_HUMAN	41 kDa	0	0	0	0	0	0	0	0	2	0
28 kDa heat- and acid-stable phosphoprotein	HAP28_HUMAN	21 kDa	0	0	0	0	0	0	0	0	2	0
Glutathione peroxidase 3	GPX3_HUMAN	26 kDa	0	0	0	0	0	0	0	0	2	0
NmrA-like family domain-containing protein 1	NMRL1_HUMAN	33 kDa	0	0	0	0	0	0	0	0	2	0
PDZ and LIM domain protein 3	PDLI3_HUMAN	39 kDa	0	0	0	0	0	0	0	0	2	0
Twinfilin-2	TWF2_HUMAN	40 kDa	0	0	0	0	0	0	0	0	2	0
Serologically defined colon cancer antigen 8	SDCG8_HUMAN	83 kDa	0	0	0	0	0	0	0	0	2	0
Prothrombin	THRB_HUMAN	70 kDa	0	0	0	1	0	0	0	0	1	0
Ectonucleotide pyrophosphatase/phosphodiesterase family member 1	ENPP1_HUMAN	105 kDa	0	0	0	0	1	0	0	0	1	0
Heterogeneous nuclear ribonucleoprotein U-like protein 1	HNRL1_HUMAN	96 kDa	1	0	0	0	0	0	0	0	1	0
Phosphoribosyl pyrophosphate synthase-associated protein 2	KPRB_HUMAN	41 kDa	1	0	0	0	0	0	0	0	1	0
Acylphosphatase-1	ACYP1_HUMAN	11 kDa	1	0	0	0	0	0	0	0	1	0
Interferon-inducible double-stranded RNA-dependent protein kinase activator A	PRKRA_HUMAN	34 kDa	1	0	0	0	0	0	0	0	1	0
Transient receptor potential cation channel subfamily V member 1	TRPV1_HUMAN	95 kDa	0	0	0	2	0	0	0	0	0	0
Keratin, type II cuticular Hb4	KRT84_HUMAN	65 kDa	0	0	0	2	0	0	0	0	0	0
Bile salt export pump	ABCBB_HUMAN	146 kDa	0	0	0	0	2	0	0	0	0	0
Separin	ESPL1_HUMAN	233 kDa	0	0	0	0	2	0	0	0	0	0
LINE-1 type transposase domain-containing protein 1	LITD1_HUMAN	99 kDa	0	0	0	0	2	0	0	0	0	0
Glutaminase liver isoform	GLSL_HUMAN	66 kDa	0	0	0	0	2	0	0	0	0	0
Valacyclovir hydrolase	BPHL_HUMAN	33 kDa	0	0	0	0	2	0	0	0	0	0
Legumain	LGMN_HUMAN	49 kDa	0	0	0	0	2	0	0	0	0	0
Cytochrome P450 1A1	CP1A1_HUMAN	58 kDa	0	0	0	0	2	0	0	0	0	0
Beta-ureidopropionase	BUP1_HUMAN	43 kDa	0	0	0	0	2	0	0	0	0	0
5-formyltetrahydrofolate cyclo-ligase	MTHFS_HUMAN	23 kDa	0	0	0	0	2	0	0	0	0	0
Retinol-binding protein 5	RET5_HUMAN	16 kDa	0	0	0	0	2	0	0	0	0	0
Serum paraoxonase/arylesterase 2	PON2_HUMAN	39 kDa	0	0	0	0	2	0	0	0	0	0
Indoleamine 2,3-dioxygenase 2	I23O2_HUMAN	45 kDa	0	0	0	0	2	0	0	0	0	0
Putative inactive carboxylesterase 4	CES1P_HUMAN	31 kDa	0	0	0	0	2	0	0	0	0	0
Arylamine N-acetyltransferase 2	ARY2_HUMAN	34 kDa	0	0	0	0	2	0	0	0	0	0
Dihydropyrimidinase	DPYS_HUMAN	57 kDa	0	0	0	0	2	0	0	0	0	0
7-alpha-hydroxycholest-4-en-3-one 12-alpha-hydroxylase	CP8B1_HUMAN	58 kDa	0	0	0	0	2	0	0	0	0	0
Probable imidazolonepropionase	HUTI_HUMAN	47 kDa	0	0	0	0	2	0	0	0	0	0
Glutaryl-CoA dehydrogenase	GCDH_HUMAN	48 kDa	0	0	0	0	2	0	0	0	0	0
1,2-dihydroxy-3-keto-5-methylthiopentene dioxygenase	MTND_HUMAN	21 kDa	0	0	0	0	2	0	0	0	0	0
Calcium-regulated heat stable protein 1	CHSP1_HUMAN	16 kDa	0	0	0	0	2	0	0	0	0	0
Dimethylaniline monooxygenase [N-oxide-forming] 5	FMO5_HUMAN	60 kDa	0	0	0	0	2	0	0	0	0	0
Cytochrome P450 2B6	CP2B6_HUMAN	56 kDa	0	0	0	0	2	0	0	0	0	0
UDP-glucuronosyltransferase 3A1	UD3A1_HUMAN	59 kDa	0	0	0	0	2	0	0	0	0	0
Saccharopine dehydrogenase-like oxidoreductase	SCPDL_HUMAN	47 kDa	0	0	0	0	2	0	0	0	0	0
Ferritin light chain	FRIL_HUMAN	20 kDa	0	0	0	0	2	0	0	0	0	0
Estradiol 17-beta-dehydrogenase 11	DHB11_HUMAN	33 kDa	0	0	0	0	2	0	0	0	0	0
Enoyl-CoA delta isomerase 2	ECI2_HUMAN	44 kDa	0	0	0	0	2	0	0	0	0	0
Acetyl-CoA acetyltransferase, cytosolic	THIC_HUMAN	41 kDa	0	0	0	0	2	0	0	0	0	0
WD repeat-containing protein 61	WDR61_HUMAN	34 kDa	0	0	0	0	2	0	0	0	0	0
Tripeptidyl-peptidase 2	TPP2_HUMAN	138 kDa	0	0	0	0	2	0	0	0	0	0
Gamma-glutamyl hydrolase	GGH_HUMAN	36 kDa	0	0	0	0	2	0	0	0	0	0
SRC kinase signaling inhibitor 1	SRCN1_HUMAN	112 kDa	1	0	0	0	1	0	0	0	0	0
AMP deaminase 2	AMPD2_HUMAN	101 kDa	1	0	0	0	1	0	0	0	0	0
Iron-sulfur cluster assembly 2 homolog	ISCA2_HUMAN	16 kDa	1	0	0	0	1	0	0	0	0	0
STE20-like serine/threonine-protein kinase	SLK_HUMAN	143 kDa	2	0	0	0	0	0	0	0	0	0

Description	Accession	MW	Raw spectral counts									
			Frontal cortex	Cerebellum	Right ventricle	Mesentric lymph node	Liver	Pancreas	Proximal bile duct	Breast	Ovary	Clitoris
Multidrug resistance-associated protein 1	MRP1_HUMAN	172 kDa	2	0	0	0	0	0	0	0	0	0
Keratin, type I cuticular Ha2	K1H2_HUMAN	50 kDa	2	0	0	0	0	0	0	0	0	0
Leucine-rich repeat-containing protein 15	LRC15_HUMAN	64 kDa	2	0	0	0	0	0	0	0	0	0
Gamma-glutamyltransferase 7	GGT7_HUMAN	70 kDa	2	0	0	0	0	0	0	0	0	0
TOM1-like protein 2	TM1L2_HUMAN	56 kDa	2	0	0	0	0	0	0	0	0	0
Neuronal growth regulator 1	NEGR1_HUMAN	39 kDa	2	0	0	0	0	0	0	0	0	0
Glutamate receptor 3	GRIA3_HUMAN	101 kDa	2	0	0	0	0	0	0	0	0	0
Ubiquilin-2	UBQL2_HUMAN	66 kDa	2	0	0	0	0	0	0	0	0	0
Target of Myb protein 1	TOM1_HUMAN	54 kDa	2	0	0	0	0	0	0	0	0	0
Disks large homolog 4	DLG4_HUMAN	80 kDa	2	0	0	0	0	0	0	0	0	0
Guanine nucleotide-binding protein G(I)/G(S)/G(O) subunit gamma-2	GBG2_HUMAN	8 kDa	2	0	0	0	0	0	0	0	0	0
Disks large homolog 2	DLG2_HUMAN	98 kDa	2	0	0	0	0	0	0	0	0	0
Sodium channel subunit beta-2	SCN2B_HUMAN	24 kDa	2	0	0	0	0	0	0	0	0	0
Glucose 1,6-bisphosphate synthase	PGM2L_HUMAN	70 kDa	2	0	0	0	0	0	0	0	0	0
Epsin-1	EPN1_HUMAN	60 kDa	2	0	0	0	0	0	0	0	0	0
Guanine nucleotide-binding protein G(I)/G(S)/G(O) subunit gamma-3	GBG3_HUMAN	8 kDa	2	0	0	0	0	0	0	0	0	0
Secretory carrier-associated membrane protein 1	SCAM1_HUMAN	38 kDa	2	0	0	0	0	0	0	0	0	0
MAGUK p55 subfamily member 2	MPP2_HUMAN	65 kDa	2	0	0	0	0	0	0	0	0	0
Phosphatidylinositol 4-kinase alpha	PI4KA_HUMAN	231 kDa	2	0	0	0	0	0	0	0	0	0
Band 4.1-like protein 1	E41L1_HUMAN	99 kDa	2	0	0	0	0	0	0	0	0	0
Dematin	DEMA_HUMAN	46 kDa	2	0	0	0	0	0	0	0	0	0
Neurocalcin-delta	NCALD_HUMAN	22 kDa	2	0	0	0	0	0	0	0	0	0
Glutamate receptor 2	GRIA2_HUMAN	99 kDa	2	0	0	0	0	0	0	0	0	0
Protein FAM49A	FA49A_HUMAN	37 kDa	2	0	0	0	0	0	0	0	0	0
DmX-like protein 2	DMXL2_HUMAN	340 kDa	2	0	0	0	0	0	0	0	0	0
ARF GTPase-activating protein GIT1	GIT1_HUMAN	84 kDa	2	0	0	0	0	0	0	0	0	0
Leucine-rich repeat-containing protein 57	LRC57_HUMAN	27 kDa	2	0	0	0	0	0	0	0	0	0
Myosin light chain 6B	MYL6B_HUMAN	23 kDa	2	0	0	0	0	0	0	0	0	0
Cytochrome c oxidase subunit 7A-related protein	COX7R_HUMAN	13 kDa	2	0	0	0	0	0	0	0	0	0
ATP-binding cassette sub-family A member 8	ABCA8_HUMAN	179 kDa	0	0	0	0	0	0	0	0	0	3
Cystatin-A	CYTA_HUMAN	11 kDa	0	0	0	0	0	0	0	0	0	3
Protein NDRG1	NDRG1_HUMAN	43 kDa	0	0	0	0	0	0	0	0	1	2
Inositol monophosphatase 1	IMPA1_HUMAN	30 kDa	1	0	0	0	0	0	0	0	0	2
Metallo-beta-lactamase domain-containing protein 2	MBLC2_HUMAN	31 kDa	0	1	0	0	0	1	0	0	0	1
Cullin-3	CUL3_HUMAN	89 kDa	1	1	0	0	0	0	0	0	0	1
116 kDa U5 small nuclear ribonucleoprotein component	U5S1_HUMAN	109 kDa	0	0	0	0	0	1	0	0	1	1
LIM and senescent cell antigen-like-containing domain protein 1	LIMS1_HUMAN	37 kDa	0	0	0	0	0	1	0	0	1	1
Fetuin-B	FETUB_HUMAN	42 kDa	0	0	0	0	0	0	2	0	0	1
Fermitin family homolog 2	FERM2_HUMAN	78 kDa	0	0	0	0	0	0	2	0	0	1
Fibulin-1	FBLN1_HUMAN	77 kDa	0	0	0	1	0	0	1	0	0	1
Ribose-phosphate pyrophosphokinase 1	PRPS1_HUMAN	35 kDa	0	0	0	0	1	0	1	0	0	1
Insulin-like growth factor-binding protein complex acid labile subunit	ALS_HUMAN	66 kDa	0	0	0	0	0	0	0	0	2	1
Cytoplasmic dynein 1 intermediate chain 2	DC1I2_HUMAN	71 kDa	0	0	0	0	0	0	0	0	2	1
ATP-binding cassette sub-family A member 13	ABCAD_HUMAN	576 kDa	1	0	0	0	0	0	0	0	1	1
Parvalbumin alpha	PRVA_HUMAN	12 kDa	0	3	0	0	0	0	0	0	0	0
Septin-4	SEPT4_HUMAN	55 kDa	0	3	0	0	0	0	0	0	0	0
Cell adhesion molecule 3	CADM3_HUMAN	43 kDa	0	3	0	0	0	0	0	0	0	0
Inositol 1,4,5-trisphosphate receptor type 3	ITPR3_HUMAN	304 kDa	0	2	0	0	0	1	0	0	0	0
Cleavage and polyadenylation specificity factor subunit 6	CPSF6_HUMAN	59 kDa	0	2	0	0	0	1	0	0	0	0
Serine/arginine-rich splicing factor 10	SRS10_HUMAN	31 kDa	0	2	0	0	0	0	0	0	1	0
Synaptotagmin-12	SYT12_HUMAN	47 kDa	1	2	0	0	0	0	0	0	0	0
Calcium/calmodulin-dependent protein kinase kinase 2	KKCC2_HUMAN	65 kDa	1	2	0	0	0	0	0	0	0	0

Description	Accession	MW	Raw spectral counts									
			Frontal cortex	Cerebellum	Right ventricle	Mesentric lymph node	Liver	Pancreas	Proximal bile duct	Breast	Ovary	Clitoris
Sodium- and chloride-dependent GABA transporter 1	SC6A1_HUMAN	67 kDa	1	2	0	0	0	0	0	0	0	0
Intersectin-1	ITSN1_HUMAN	195 kDa	1	2	0	0	0	0	0	0	0	0
Phytanoyl-CoA hydroxylase-interacting protein	PHYIP_HUMAN	38 kDa	1	2	0	0	0	0	0	0	0	0
Leukocyte surface antigen CD47	CD47_HUMAN	35 kDa	1	2	0	0	0	0	0	0	0	0
AP-2 complex subunit sigma	AP2S1_HUMAN	17 kDa	1	2	0	0	0	0	0	0	0	0
COP9 signalosome complex subunit 4	CSN4_HUMAN	46 kDa	1	2	0	0	0	0	0	0	0	0
Septin-9	SEPT9_HUMAN	65 kDa	1	2	0	0	0	0	0	0	0	0
Epimerase family protein SDR39U1	D39U1_HUMAN	35 kDa	0	1	1	0	0	1	0	0	0	0
Calcium-binding protein 39-like	CB39L_HUMAN	39 kDa	1	1	1	0	0	0	0	0	0	0
Activator of 90 kDa heat shock protein ATPase homolog 1	AHSA1_HUMAN	38 kDa	1	1	1	0	0	0	0	0	0	0
EPM2A-interacting protein 1	EPMIP_HUMAN	70 kDa	1	1	0	0	0	1	0	0	0	0
Dolichyl-diphosphooligosaccharide--protein glycosyltransferase subunit STT3B	STT3B_HUMAN	94 kDa	0	1	0	0	0	0	1	0	1	0
Tubulin-folding cofactor B	TBCB_HUMAN	27 kDa	0	1	0	0	0	0	0	0	2	0
TAR DNA-binding protein 43	TADBP_HUMAN	45 kDa	0	1	0	0	0	0	0	0	2	0
Serine/arginine-rich splicing factor 2	SRSF2_HUMAN	25 kDa	0	1	0	0	0	0	0	0	2	0
Nesprin-1	SYNE1_HUMAN	1011 kDa	1	1	0	0	0	0	0	0	1	0
BRI3-binding protein	BRI3B_HUMAN	28 kDa	0	1	0	0	2	0	0	0	0	0
V-type proton ATPase 16 kDa proteolipid subunit	VATL_HUMAN	16 kDa	2	1	0	0	0	0	0	0	0	0
LanC-like protein 2	LANC2_HUMAN	51 kDa	2	1	0	0	0	0	0	0	0	0
Kelch repeat and BTB domain-containing protein 11	KBTBB_HUMAN	66 kDa	2	1	0	0	0	0	0	0	0	0
Gamma-aminobutyric acid receptor-associated protein-like 2	GBRL2_HUMAN	14 kDa	2	1	0	0	0	0	0	0	0	0
Armadillo repeat-containing protein 1	ARMC1_HUMAN	31 kDa	2	1	0	0	0	0	0	0	0	0
Cytoplasmic dynein 1 light intermediate chain 2	DC1L2_HUMAN	54 kDa	2	1	0	0	0	0	0	0	0	0
2-oxoisovalerate dehydrogenase subunit alpha	ODBA_HUMAN	50 kDa	0	0	3	0	0	0	0	0	0	0
Ribosome-recycling factor	RRFM_HUMAN	29 kDa	0	0	3	0	0	0	0	0	0	0
Plasminogen receptor (KT)	PLRKT_HUMAN	17 kDa	0	0	3	0	0	0	0	0	0	0
Coenzyme Q-binding protein COQ10 homolog A	CQ10A_HUMAN	28 kDa	0	0	3	0	0	0	0	0	0	0
Microtubule-associated protein 4	MAP4_HUMAN	121 kDa	0	0	3	0	0	0	0	0	0	0
Basigin	BASI_HUMAN	42 kDa	0	0	3	0	0	0	0	0	0	0
HLA class II histocompatibility antigen, DR alpha chain	DRA_HUMAN	29 kDa	0	0	3	0	0	0	0	0	0	0
Junctophilin-2	JPH2_HUMAN	74 kDa	0	0	3	0	0	0	0	0	0	0
Sodium/calcium exchanger 1	NAC1_HUMAN	109 kDa	0	0	3	0	0	0	0	0	0	0
Cardiac phospholamban	PPLA_HUMAN	6 kDa	0	0	3	0	0	0	0	0	0	0
Regulator of microtubule dynamics protein 1	RMD1_HUMAN	36 kDa	0	0	3	0	0	0	0	0	0	0
SH3 domain-binding glutamic acid-rich protein	SH3BG_HUMAN	26 kDa	0	0	3	0	0	0	0	0	0	0
39S ribosomal protein L49	RM49_HUMAN	19 kDa	0	0	2	0	0	1	0	0	0	0
Acyl-protein thioesterase 1	LYPA1_HUMAN	25 kDa	0	0	2	0	0	1	0	0	0	0
Persulfide dioxygenase ETHE1	ETHE1_HUMAN	28 kDa	0	0	2	0	0	0	0	0	1	0
Putative hexokinase HKDC1	HKDC1_HUMAN	103 kDa	0	0	2	0	1	0	0	0	0	0
39S ribosomal protein L11	RM11_HUMAN	21 kDa	0	0	2	0	1	0	0	0	0	0
Transmembrane protein 126A	T126A_HUMAN	22 kDa	0	0	2	0	1	0	0	0	0	0
Glutaredoxin-1	GLRX1_HUMAN	12 kDa	0	0	2	0	1	0	0	0	0	0
Cytochrome c oxidase subunit 6C	COX6C_HUMAN	9 kDa	1	0	2	0	0	0	0	0	0	0
NADH dehydrogenase [ubiquinone] 1 beta subcomplex subunit 9	NDUB9_HUMAN	22 kDa	1	0	2	0	0	0	0	0	0	0
Isobutyryl-CoA dehydrogenase	ACAD8_HUMAN	45 kDa	0	0	1	0	0	2	0	0	0	0
Ubiquitin-fold modifier-conjugating enzyme 1	UFC1_HUMAN	19 kDa	0	0	1	0	0	1	0	0	1	0
Ubiquitin carboxyl-terminal hydrolase 14	UBP14_HUMAN	56 kDa	0	0	1	0	0	1	0	0	1	0
Mitochondrial import inner membrane translocase subunit TIM16	TIM16_HUMAN	14 kDa	1	0	1	0	0	1	0	0	0	0
Extended synaptotagmin-2	ESYT2_HUMAN	102 kDa	1	0	1	0	0	0	0	0	1	0
Integrin alpha-7	ITA7_HUMAN	129 kDa	0	0	1	2	0	0	0	0	0	0
Iron-sulfur protein NUBPL	NUBPL_HUMAN	34 kDa	0	0	1	0	2	0	0	0	0	0
Mitochondrial import inner membrane translocase subunit Tim8 A	TIM8A_HUMAN	11 kDa	0	0	1	0	2	0	0	0	0	0

Description	Accession	MW	Raw spectral counts									
			Frontal cortex	Cerebellum	Right ventricle	Mesentric lymph node	Liver	Pancreas	Proximal bile duct	Breast	Ovary	Clitoris
Tripeptidyl-peptidase 1	TPP1_HUMAN	61 kDa	1	0	1	0	1	0	0	0	0	0
Cytochrome c oxidase assembly factor 3 homolog	COA3_HUMAN	12 kDa	2	0	1	0	0	0	0	0	0	0
Isocitrate dehydrogenase [NAD] subunit gamma	IDH3G_HUMAN	43 kDa	2	0	1	0	0	0	0	0	0	0
Coiled-coil domain-containing protein 47	CCD47_HUMAN	56 kDa	0	0	0	0	0	3	0	0	0	0
Branched-chain-amino-acid aminotransferase, cytosolic	BCAT1_HUMAN	43 kDa	0	0	0	0	0	3	0	0	0	0
Signal recognition particle 19 kDa protein	SRP19_HUMAN	16 kDa	0	0	0	0	0	3	0	0	0	0
Protein transport protein Sec24C	SC24C_HUMAN	118 kDa	0	0	0	0	0	3	0	0	0	0
Prolactin regulatory element-binding protein	PREB_HUMAN	45 kDa	0	0	0	0	0	3	0	0	0	0
Mannose-1-phosphate guanyltransferase beta	GMPPB_HUMAN	40 kDa	0	0	0	0	0	3	0	0	0	0
Transducin beta-like protein 2	TBL2_HUMAN	50 kDa	0	0	0	0	0	3	0	0	0	0
Signal recognition particle receptor subunit alpha	SRPR_HUMAN	70 kDa	0	0	0	0	0	3	0	0	0	0
Translocating chain-associated membrane protein 1	TRAM1_HUMAN	43 kDa	0	0	0	0	0	3	0	0	0	0
ER membrane protein complex subunit 2	EMC2_HUMAN	35 kDa	0	0	0	0	0	3	0	0	0	0
Eukaryotic translation initiation factor 3 subunit C-like protein	EIFCL_HUMAN	105 kDa	0	0	0	0	0	3	0	0	0	0
Gamma-glutamyltranspeptidase 1	GGT1_HUMAN	61 kDa	0	0	0	0	0	3	0	0	0	0
Arginine--tRNA ligase, cytoplasmic	SYRC_HUMAN	75 kDa	0	0	0	0	0	3	0	0	0	0
Aminoacyl tRNA synthase complex-interacting multifunctional protein 1	AIMP1_HUMAN	34 kDa	0	0	0	0	0	2	0	0	1	0
60S ribosomal protein L32	RL32_HUMAN	16 kDa	0	0	0	0	0	2	0	0	1	0
Transmembrane emp24 domain-containing protein 9	TMED9_HUMAN	27 kDa	0	0	0	0	0	2	0	0	1	0
Peroxisomal biogenesis factor 19	PEX19_HUMAN	33 kDa	0	0	0	0	1	2	0	0	0	0
U6 snRNA-associated Sm-like protein LSm8	LSM8_HUMAN	10 kDa	1	0	0	0	0	2	0	0	0	0
DNA repair protein RAD50	RAD50_HUMAN	154 kDa	0	0	0	0	0	1	0	0	2	0
Calumenin	CALU_HUMAN	37 kDa	0	0	0	0	0	1	0	0	2	0
Splicing factor 3B subunit 2	SF3B2_HUMAN	100 kDa	0	0	0	0	0	1	0	0	2	0
H/ACA ribonucleoprotein complex subunit 4	DKC1_HUMAN	58 kDa	0	0	0	0	0	1	0	0	2	0
Transcriptional activator protein Pur-beta	PURB_HUMAN	33 kDa	0	0	0	0	0	1	0	0	2	0
Heterogeneous nuclear ribonucleoprotein A/B	ROAA_HUMAN	36 kDa	0	0	0	0	0	1	0	0	2	0
Eukaryotic translation initiation factor 3 subunit E	EIF3E_HUMAN	52 kDa	0	0	0	0	0	1	0	0	2	0
Protein SCO1 homolog	SCO1_HUMAN	34 kDa	0	0	0	0	0	1	0	0	2	0
COP9 signalosome complex subunit 6	CSN6_HUMAN	36 kDa	0	0	0	0	0	1	0	0	2	0
Small nuclear ribonucleoprotein Sm D3	SMD3_HUMAN	14 kDa	0	0	0	0	1	1	0	0	1	0
Catechol O-methyltransferase	COMT_HUMAN	30 kDa	0	0	0	0	1	1	0	0	1	0
Prosaposin	SAP_HUMAN	58 kDa	1	0	0	0	0	1	0	0	1	0
cAMP-dependent protein kinase catalytic subunit gamma	KAPCG_HUMAN	40 kDa	1	0	0	0	0	1	0	0	1	0
Acyl-CoA synthetase family member 2	ACSF2_HUMAN	68 kDa	0	0	0	0	2	1	0	0	0	0
60S ribosomal protein L30	RL30_HUMAN	13 kDa	0	0	0	0	2	1	0	0	0	0
Carbonyl reductase family member 4	CBR4_HUMAN	25 kDa	0	0	0	0	2	1	0	0	0	0
Mitochondrial import receptor subunit TOM20 homolog	TOM20_HUMAN	16 kDa	1	0	0	0	1	1	0	0	0	0
Tumor protein D52	TPD52_HUMAN	24 kDa	2	0	0	0	0	1	0	0	0	0
Galectin-2	LEG2_HUMAN	15 kDa	0	0	0	0	0	0	3	0	0	0
Putative histone H2B type 2-D	H2B2D_HUMAN	18 kDa	0	0	0	0	0	0	3	0	0	0
Tudor domain-containing protein 7	TDRD7_HUMAN	124 kDa	0	0	0	1	0	0	2	0	0	0
Plexin-B2	PLXB2_HUMAN	205 kDa	0	0	0	0	0	0	0	0	3	0
Cyclin-dependent kinase 4	CDK4_HUMAN	34 kDa	0	0	0	0	0	0	0	0	3	0
Inactive tyrosine-protein kinase 7	PTK7_HUMAN	118 kDa	0	0	0	0	0	0	0	0	3	0
AH receptor-interacting protein	AIP_HUMAN	38 kDa	0	0	0	0	0	0	0	0	3	0
Dipeptidase 1	DPEP1_HUMAN	46 kDa	0	0	0	0	0	0	0	0	3	0
Peptidyl-prolyl cis-trans isomerase FKBP7	FKBP7_HUMAN	30 kDa	0	0	0	0	0	0	0	0	3	0
Cytoglobin	CYGB_HUMAN	21 kDa	0	0	0	0	0	0	0	0	3	0
Protein-arginine deiminase type-6	PADI6_HUMAN	78 kDa	0	0	0	0	0	0	0	0	3	0
ADP-ribosyl cyclase/cyclic ADP-ribose hydrolase 2	BST1_HUMAN	36 kDa	0	0	0	0	0	0	0	0	3	0
U6 snRNA-associated Sm-like protein LSm4	LSM4_HUMAN	15 kDa	0	0	0	0	0	0	0	0	3	0

Description	Accession	MW	Raw spectral counts									
			Frontal cortex	Cerebellum	Right ventricle	Mesentric lymph node	Liver	Pancreas	Proximal bile duct	Breast	Ovary	Clitoris
Non-histone chromosomal protein HMG-14	HMGN1_HUMAN	11 kDa	0	0	0	0	0	0	0	0	3	0
L-lactate dehydrogenase A-like 6A	LDH6A_HUMAN	37 kDa	0	0	0	0	0	0	0	0	3	0
NADPH:adrenodoxin oxidoreductase	ADRO_HUMAN	54 kDa	0	0	0	0	0	0	0	0	3	0
Mitotic checkpoint protein BUB3	BUB3_HUMAN	37 kDa	0	0	0	0	0	0	0	0	3	0
Small nuclear ribonucleoprotein F	RUXF_HUMAN	10 kDa	0	0	0	0	0	0	0	0	3	0
Apoptosis regulator BAX	BAX_HUMAN	21 kDa	0	0	0	0	0	0	0	0	3	0
High mobility group protein B3	HMGB3_HUMAN	23 kDa	0	0	0	0	0	0	0	0	3	0
U2 small nuclear ribonucleoprotein B''	RU2B_HUMAN	25 kDa	0	0	0	0	0	0	0	0	3	0
Casein kinase II subunit alpha	CSK21_HUMAN	45 kDa	0	0	0	0	0	0	0	0	3	0
Proteolipid protein 2	PLP2_HUMAN	17 kDa	0	0	0	0	0	0	0	0	3	0
Poly(ADP-ribose) glycohydrolase ARH3	ARHL2_HUMAN	39 kDa	0	0	0	0	0	0	0	0	3	0
SAP domain-containing ribonucleoprotein	SARNP_HUMAN	24 kDa	0	0	0	0	0	0	0	0	3	0
Tubulin--tyrosine ligase-like protein 12	TTL12_HUMAN	74 kDa	0	0	0	0	0	0	0	0	3	0
Spermine synthase	SPSY_HUMAN	41 kDa	0	0	0	0	0	0	0	0	3	0
26S protease regulatory subunit 6B	PRS6B_HUMAN	47 kDa	0	0	0	0	0	0	0	0	3	0
N-acylneuraminate cytidylyltransferase	NEUA_HUMAN	48 kDa	0	0	0	0	1	0	0	0	2	0
DnaJ homolog subfamily A member 1	DNJA1_HUMAN	45 kDa	0	0	0	0	2	0	0	0	1	0
Farnesyl pyrophosphate synthase	FPPS_HUMAN	48 kDa	0	0	0	0	2	0	0	0	1	0
Glutaredoxin-related protein 5	GLRX5_HUMAN	17 kDa	1	0	0	0	1	0	0	0	1	0
Acetyl-CoA carboxylase 2	ACACB_HUMAN	277 kDa	0	0	0	3	0	0	0	0	0	0
Histidine ammonia-lyase	HUTH_HUMAN	73 kDa	0	0	0	2	1	0	0	0	0	0
Fibronectin	FINC_HUMAN	263 kDa	0	0	0	1	2	0	0	0	0	0
Calcineurin B homologous protein 1	CHP1_HUMAN	22 kDa	2	0	0	1	0	0	0	0	0	0
Probable 2-oxoglutarate dehydrogenase E1 component DHKTD1	DHTK1_HUMAN	103 kDa	0	0	0	0	3	0	0	0	0	0
Macrophage mannose receptor 1	MRC1_HUMAN	166 kDa	0	0	0	0	3	0	0	0	0	0
Mitochondrial ornithine transporter 1	ORNT1_HUMAN	33 kDa	0	0	0	0	3	0	0	0	0	0
Cytochrome P450 2J2	CP2J2_HUMAN	58 kDa	0	0	0	0	3	0	0	0	0	0
Adrenodoxin	ADX_HUMAN	19 kDa	0	0	0	0	3	0	0	0	0	0
Arsenite methyltransferase	AS3MT_HUMAN	42 kDa	0	0	0	0	3	0	0	0	0	0
Hydroxyacid oxidase 2	HAOX2_HUMAN	39 kDa	0	0	0	0	3	0	0	0	0	0
5'-AMP-activated protein kinase subunit gamma-1	AAKG1_HUMAN	38 kDa	0	0	0	0	3	0	0	0	0	0
Delta(14)-sterol reductase	ERG24_HUMAN	46 kDa	0	0	0	0	3	0	0	0	0	0
SEC14-like protein 4	S14L4_HUMAN	47 kDa	0	0	0	0	3	0	0	0	0	0
Beta-lactamase-like protein 2	LACB2_HUMAN	33 kDa	0	0	0	0	3	0	0	0	0	0
Putative L-aspartate dehydrogenase	ASPD_HUMAN	30 kDa	0	0	0	0	3	0	0	0	0	0
Solute carrier family 2, facilitated glucose transporter member 2	GTR2_HUMAN	57 kDa	0	0	0	0	3	0	0	0	0	0
Gephyrin	GEPH_HUMAN	80 kDa	0	0	0	0	3	0	0	0	0	0
Annexin A8	ANXA8_HUMAN	37 kDa	0	0	0	0	3	0	0	0	0	0
ATP-binding cassette sub-family D member 3	ABCD3_HUMAN	75 kDa	0	0	0	0	3	0	0	0	0	0
Mitochondrial carnitine/acylcarnitine carrier protein	MCAT_HUMAN	33 kDa	0	0	0	0	3	0	0	0	0	0
Syntaxin-7	STX7_HUMAN	30 kDa	2	0	0	0	1	0	0	0	0	0
Keratin, type I cuticular Ha7	KRT37_HUMAN	50 kDa	3	0	0	0	0	0	0	0	0	0
SLIT-ROBO Rho GTPase-activating protein 3	SRGP3_HUMAN	125 kDa	3	0	0	0	0	0	0	0	0	0
Kinesin light chain 2	KLC2_HUMAN	69 kDa	3	0	0	0	0	0	0	0	0	0
Liprin-alpha-3	LIPA3_HUMAN	134 kDa	3	0	0	0	0	0	0	0	0	0
Catenin delta-2	CTND2_HUMAN	133 kDa	3	0	0	0	0	0	0	0	0	0
Pseudouridine-5'-monophosphatase	HDHD1_HUMAN	25 kDa	3	0	0	0	0	0	0	0	0	0
LisH domain and HEAT repeat-containing protein KIAA1468	K1468_HUMAN	135 kDa	3	0	0	0	0	0	0	0	0	0
Stathmin-2	STMN2_HUMAN	21 kDa	3	0	0	0	0	0	0	0	0	0
Protein KIAA1045	K1045_HUMAN	45 kDa	3	0	0	0	0	0	0	0	0	0
Sodium-driven chloride bicarbonate exchanger	S4A10_HUMAN	126 kDa	3	0	0	0	0	0	0	0	0	0
Wiskott-Aldrich syndrome protein family member 1	WASF1_HUMAN	62 kDa	3	0	0	0	0	0	0	0	0	0

Description	Accession	MW	Raw spectral counts									
			Frontal cortex	Cerebellum	Right ventricle	Mesentric lymph node	Liver	Pancreas	Proximal bile duct	Breast	Ovary	Clitoris
Adaptin ear-binding coat-associated protein 1	NECP1_HUMAN	30 kDa	3	0	0	0	0	0	0	0	0	0
Synaptogyrin-3	SNG3_HUMAN	25 kDa	3	0	0	0	0	0	0	0	0	0
Neural cell adhesion molecule L1-like protein	CHL1_HUMAN	135 kDa	3	0	0	0	0	0	0	0	0	0
Neural cell adhesion molecule 2	NCAM2_HUMAN	93 kDa	3	0	0	0	0	0	0	0	0	0
Synaptopodin	SYNPO_HUMAN	99 kDa	3	0	0	0	0	0	0	0	0	0
Carbonyl reductase [NADPH] 3	CBR3_HUMAN	31 kDa	3	0	0	0	0	0	0	0	0	0
Copine-6	CPNE6_HUMAN	62 kDa	3	0	0	0	0	0	0	0	0	0
Rho-related GTP-binding protein RhoB	RHOB_HUMAN	22 kDa	3	0	0	0	0	0	0	0	0	0
Ubiquitin-conjugating enzyme E2 K	UBE2K_HUMAN	22 kDa	3	0	0	0	0	0	0	0	0	0
Nucleosome assembly protein 1-like 4	NP1L4_HUMAN	43 kDa	3	0	0	0	0	0	0	0	0	0
von Willebrand factor	VWF_HUMAN	309 kDa	0	0	1	0	0	0	0	0	0	3
Insulin-degrading enzyme	IDE_HUMAN	118 kDa	0	0	0	0	0	1	0	0	0	3
Protein AHNAK2	AHNK2_HUMAN	617 kDa	0	0	0	0	0	0	1	0	0	3
1-phosphatidylinositol 4,5-bisphosphate phosphodiesterase delta-1	PLCD1_HUMAN	86 kDa	1	1	0	0	0	0	0	0	0	2
Fibrinogen alpha chain	FIBA_HUMAN	95 kDa	0	0	2	0	0	0	0	0	0	2
CD44 antigen	CD44_HUMAN	82 kDa	0	0	0	0	0	1	0	0	1	2
Peflin	PEF1_HUMAN	30 kDa	1	0	0	0	0	1	0	0	0	2
Apolipoprotein D	APOD_HUMAN	21 kDa	0	0	0	0	0	0	2	0	0	2
Gamma-glutamyltransferase 5	GGT5_HUMAN	62 kDa	0	0	0	0	0	0	0	0	2	2
Inter-alpha-trypsin inhibitor heavy chain H2	ITIH2_HUMAN	106 kDa	0	0	0	0	0	0	0	0	2	2
Protein transport protein Sec23A	SC23A_HUMAN	86 kDa	0	0	0	0	0	0	0	0	2	2
Dermatopontin	DERM_HUMAN	24 kDa	0	0	0	0	0	0	0	0	2	2
Spectrin alpha chain, erythrocytic 1	SPTA1_HUMAN	280 kDa	0	2	0	0	1	0	0	0	0	1
Nck-associated protein 1	NCKP1_HUMAN	129 kDa	1	2	0	0	0	0	0	0	0	1
Leucine-rich repeat-containing protein 47	LRC47_HUMAN	63 kDa	0	1	1	0	0	0	0	0	1	1
Small ubiquitin-related modifier 2	SUMO2_HUMAN	11 kDa	0	1	0	0	1	1	0	0	0	1
Microtubule-actin cross-linking factor 1, isoforms 1/2/3/5	MACF1_HUMAN	838 kDa	1	1	0	0	0	0	0	0	1	1
Ig lambda chain V-III region LOI	LV302_HUMAN	12 kDa	0	0	1	0	0	0	1	0	1	1
Unconventional myosin-VI	MYO6_HUMAN	150 kDa	0	0	0	0	0	3	0	0	0	1
Leucine--tRNA ligase, cytoplasmic	SYLC_HUMAN	134 kDa	1	0	0	0	0	2	0	0	0	1
Olfactomedin-like protein 3	OLFL3_HUMAN	46 kDa	0	0	0	0	0	0	2	0	1	1
Peptidyl-prolyl cis-trans isomerase FKBP1A	FKB1A_HUMAN	12 kDa	1	0	0	0	0	0	1	0	1	1
Complement component C9	CO9_HUMAN	63 kDa	0	0	0	0	0	0	0	0	3	1
Prostaglandin F2 receptor negative regulator	FPRP_HUMAN	99 kDa	0	0	0	0	1	0	0	0	2	1
HLA class I histocompatibility antigen, Cw-17 alpha chain	1C17_HUMAN	41 kDa	0	0	0	0	2	0	0	0	1	1
Secretory carrier-associated membrane protein 5	SCAM5_HUMAN	26 kDa	0	4	0	0	0	0	0	0	0	0
Transcription elongation factor A protein-like 3	TCAL3_HUMAN	23 kDa	0	4	0	0	0	0	0	0	0	0
Putative adenosylhomocysteinase 2	SAHH2_HUMAN	59 kDa	0	4	0	0	0	0	0	0	0	0
PI-PLC X domain-containing protein 3	PLCX3_HUMAN	36 kDa	1	3	0	0	0	0	0	0	0	0
Cell adhesion molecule 4	CADM4_HUMAN	43 kDa	1	3	0	0	0	0	0	0	0	0
AP-2 complex subunit mu	AP2M1_HUMAN	50 kDa	1	3	0	0	0	0	0	0	0	0
Contactin-associated protein 1	CNTP1_HUMAN	156 kDa	1	3	0	0	0	0	0	0	0	0
Synaptic vesicle membrane protein VAT-1 homolog-like	VAT1L_HUMAN	46 kDa	1	3	0	0	0	0	0	0	0	0
Cytochrome c oxidase subunit 2	COX2_HUMAN	26 kDa	0	2	2	0	0	0	0	0	0	0
Transformer-2 protein homolog beta	TRA2B_HUMAN	34 kDa	0	2	0	0	0	0	0	0	2	0
Na(+)/H(+) exchange regulatory cofactor NHE-RF1	NHRF1_HUMAN	39 kDa	2	2	0	0	0	0	0	0	0	0
GTP-binding protein Di-Ras2	DIRA2_HUMAN	22 kDa	2	2	0	0	0	0	0	0	0	0
Neurotrimin	NTRI_HUMAN	38 kDa	2	2	0	0	0	0	0	0	0	0
Actin-related protein 2/3 complex subunit 5-like protein	ARP5L_HUMAN	17 kDa	2	2	0	0	0	0	0	0	0	0
Mitochondrial import receptor subunit TOM22 homolog	TOM22_HUMAN	16 kDa	1	1	1	0	1	0	0	0	0	0
Histidine triad nucleotide-binding protein 2	HINT2_HUMAN	17 kDa	2	1	1	0	0	0	0	0	0	0
Xaa-Pro aminopeptidase 1	XPP1_HUMAN	70 kDa	0	1	0	0	0	1	0	0	2	0

Description	Accession	MW	Raw spectral counts									
			Frontal cortex	Cerebellum	Right ventricle	Mesentric lymph node	Liver	Pancreas	Proximal bile duct	Breast	Ovary	Clitoris
Splicing factor 3B subunit 1	SF3B1_HUMAN	146 kDa	0	1	0	0	0	0	0	0	3	0
Profilin-2	PROF2_HUMAN	15 kDa	2	1	0	0	0	0	0	0	1	0
PH and SEC7 domain-containing protein 3	PSD3_HUMAN	116 kDa	3	1	0	0	0	0	0	0	0	0
Vesicular glutamate transporter 1	VGLU1_HUMAN	62 kDa	3	1	0	0	0	0	0	0	0	0
Mitochondrial glutamate carrier 1	GHC1_HUMAN	34 kDa	3	1	0	0	0	0	0	0	0	0
Probable D-lactate dehydrogenase	LDHD_HUMAN	55 kDa	0	0	4	0	0	0	0	0	0	0
Probable C->U-editing enzyme APOBEC-2	ABEC2_HUMAN	26 kDa	0	0	4	0	0	0	0	0	0	0
Sorting and assembly machinery component 50 homolog	SAM50_HUMAN	52 kDa	0	0	4	0	0	0	0	0	0	0
Tripartite motif-containing protein 72	TRI72_HUMAN	53 kDa	0	0	4	0	0	0	0	0	0	0
Polyribonucleotide nucleotidyltransferase 1	PNPT1_HUMAN	86 kDa	0	0	3	0	0	1	0	0	0	0
Chaperone activity of bc1 complex-like	ADCK3_HUMAN	72 kDa	0	0	3	0	0	1	0	0	0	0
Delta-sarcoglycan	SGCD_HUMAN	32 kDa	0	0	3	0	0	0	1	0	0	0
Uncharacterized protein KIAA1210	K1210_HUMAN	187 kDa	1	0	3	0	0	0	0	0	0	0
Cadherin-2	CADH2_HUMAN	100 kDa	1	0	3	0	0	0	0	0	0	0
39S ribosomal protein L43	RM43_HUMAN	23 kDa	0	0	2	0	0	2	0	0	0	0
Mitochondrial import inner membrane translocase subunit Tim13	TIM13_HUMAN	11 kDa	0	0	2	0	1	1	0	0	0	0
Prefoldin subunit 5	PFD5_HUMAN	17 kDa	1	0	2	0	0	0	0	0	1	0
2-oxoisovalerate dehydrogenase subunit beta	ODBB_HUMAN	43 kDa	0	0	2	0	2	0	0	0	0	0
Fructosamine-3-kinase	FN3K_HUMAN	35 kDa	2	0	2	0	0	0	0	0	0	0
NADH dehydrogenase [ubiquinone] iron-sulfur protein 6	NDUS6_HUMAN	14 kDa	2	0	2	0	0	0	0	0	0	0
Filaggrin-2	FILA2_HUMAN	248 kDa	1	0	1	0	0	2	0	0	0	0
Importin subunit alpha-3	IMA3_HUMAN	58 kDa	2	0	1	0	0	1	0	0	0	0
Acyl-coenzyme A thioesterase 13	ACO13_HUMAN	15 kDa	1	0	1	0	2	0	0	0	0	0
LYR motif-containing protein 4	LYRM4_HUMAN	11 kDa	2	0	1	0	1	0	0	0	0	0
Ubiquitin-fold modifier 1	UFM1_HUMAN	9 kDa	0	0	0	0	0	4	0	0	0	0
UDP-N-acetylhexosamine pyrophosphorylase	UAP1_HUMAN	59 kDa	0	0	0	0	0	4	0	0	0	0
Carboxypeptidase A2	CBPA2_HUMAN	47 kDa	0	0	0	0	0	4	0	0	0	0
BTB/POZ domain-containing protein KCTD14	KCD14_HUMAN	30 kDa	0	0	0	0	0	4	0	0	0	0
Sialic acid synthase	SIAS_HUMAN	40 kDa	0	0	0	0	0	4	0	0	0	0
Plasminogen activator inhibitor 1 RNA-binding protein	PAIRB_HUMAN	45 kDa	0	0	0	0	0	4	0	0	0	0
Eukaryotic translation initiation factor 3 subunit B	EIF3B_HUMAN	92 kDa	0	0	0	0	0	3	0	0	1	0
Poly [ADP-ribose] polymerase 1	PARP1_HUMAN	113 kDa	0	0	0	0	0	3	0	0	1	0
Multiple coagulation factor deficiency protein 2	MCFD2_HUMAN	16 kDa	0	0	0	0	1	3	0	0	0	0
Cystathionine beta-synthase	CBS_HUMAN	61 kDa	0	0	0	0	1	3	0	0	0	0
Mannose-1-phosphate guanyltransferase alpha	GMPPA_HUMAN	46 kDa	0	0	0	0	1	3	0	0	0	0
Fatty-acid amide hydrolase 1	FAAH1_HUMAN	63 kDa	1	0	0	0	0	3	0	0	0	0
Nucleolar protein 58	NOP58_HUMAN	60 kDa	0	0	0	0	0	2	0	0	2	0
26S proteasome non-ATPase regulatory subunit 7	PSMD7_HUMAN	37 kDa	0	0	0	0	0	2	0	0	2	0
Membrane-associated progesterone receptor component 2	PGRC2_HUMAN	24 kDa	0	0	0	0	1	2	0	0	1	0
Transmembrane and coiled-coil domain-containing protein 1	TMCO1_HUMAN	21 kDa	0	0	0	0	2	2	0	0	0	0
Dolichol-phosphate mannosyltransferase subunit 1	DPM1_HUMAN	30 kDa	0	0	0	0	2	2	0	0	0	0
NEDD8-conjugating enzyme Ubc12	UBC12_HUMAN	21 kDa	2	0	0	0	0	2	0	0	0	0
Osteoclast-stimulating factor 1	OSTF1_HUMAN	24 kDa	0	0	0	0	0	1	1	0	2	0
Nucleolar protein 56	NOP56_HUMAN	66 kDa	0	0	0	0	0	1	0	0	3	0
Histone-binding protein RBBP4	RBBP4_HUMAN	48 kDa	0	0	0	0	0	1	0	0	3	0
Ribosome maturation protein SBDS	SBDS_HUMAN	29 kDa	0	0	0	0	2	1	0	0	1	0
Selenocysteine lyase	SCLY_HUMAN	48 kDa	0	0	0	0	3	1	0	0	0	0
Coronin-1C	COR1C_HUMAN	53 kDa	0	0	0	0	0	0	3	0	1	0
Ig kappa chain C region	IGKC_HUMAN	12 kDa	0	0	0	0	3	0	1	0	0	0
Chromodomain-helicase-DNA-binding protein 4	CHD4_HUMAN	218 kDa	0	0	0	0	0	0	0	0	4	0
Programmed cell death protein 5	PDCD5_HUMAN	14 kDa	0	0	0	0	0	0	0	0	4	0
Guanine nucleotide-binding protein subunit alpha-13	GNA13_HUMAN	44 kDa	0	0	0	0	0	0	0	0	4	0

Description	Accession	MW	Raw spectral counts									
			Frontal cortex	Cerebellum	Right ventricle	Mesentric lymph node	Liver	Pancreas	Proximal bile duct	Breast	Ovary	Clitoris
MARCKS-related protein	MRP_HUMAN	20 kDa	0	0	0	0	0	0	0	0	4	0
Regulation of nuclear pre-mRNA domain-containing protein 1B	RPR1B_HUMAN	37 kDa	0	0	0	0	0	0	0	0	4	0
Ubiquitin carboxyl-terminal hydrolase isozyme L3	UCHL3_HUMAN	26 kDa	0	0	0	0	0	0	0	0	4	0
Nucleosome assembly protein 1-like 1	NP1L1_HUMAN	45 kDa	0	0	0	0	0	0	0	0	4	0
Far upstream element-binding protein 1	FUBP1_HUMAN	68 kDa	0	0	0	0	0	0	0	0	4	0
Chromobox protein homolog 3	CBX3_HUMAN	21 kDa	0	0	0	0	0	0	0	0	4	0
COP9 signalosome complex subunit 8	CSN8_HUMAN	23 kDa	1	0	0	0	1	0	0	0	2	0
Ubiquitin carboxyl-terminal hydrolase 7	UBP7_HUMAN	128 kDa	2	0	0	0	0	0	0	0	2	0
ADP-ribosylation factor-like protein 2	ARL2_HUMAN	21 kDa	2	0	0	0	0	0	0	0	2	0
Cysteine-rich protein 2	CRIP2_HUMAN	22 kDa	2	0	0	0	0	0	0	0	2	0
Retinol-binding protein 4	RET4_HUMAN	23 kDa	0	0	0	0	3	0	0	0	1	0
Haloacid dehalogenase-like hydrolase domain-containing protein 3	HDHD3_HUMAN	28 kDa	1	0	0	0	2	0	0	0	1	0
Asialoglycoprotein receptor 1	ASGR1_HUMAN	33 kDa	0	0	0	0	4	0	0	0	0	0
Cytochrome P450 2E1	CP2E1_HUMAN	57 kDa	0	0	0	0	4	0	0	0	0	0
Alpha-tocopherol transfer protein	TTPA_HUMAN	32 kDa	0	0	0	0	4	0	0	0	0	0
UDP-glucuronosyltransferase 2B15	UDB15_HUMAN	61 kDa	0	0	0	0	4	0	0	0	0	0
Very long-chain acyl-CoA synthetase	S27A2_HUMAN	70 kDa	0	0	0	0	4	0	0	0	0	0
Methyltransferase-like protein 7B	MET7B_HUMAN	28 kDa	0	0	0	0	4	0	0	0	0	0
Tetratricopeptide repeat protein 36	TTC36_HUMAN	21 kDa	0	0	0	0	4	0	0	0	0	0
Sterol 26-hydroxylase	CP27A_HUMAN	60 kDa	0	0	0	0	4	0	0	0	0	0
Phosphoenolpyruvate carboxykinase, cytosolic [GTP]	PCKGC_HUMAN	69 kDa	0	0	0	0	4	0	0	0	0	0
Fatty aldehyde dehydrogenase	AL3A2_HUMAN	55 kDa	0	0	0	0	4	0	0	0	0	0
1,4-alpha-glucan-branching enzyme	GLGB_HUMAN	80 kDa	0	0	0	0	4	0	0	0	0	0
Regulator of microtubule dynamics protein 3	RMD3_HUMAN	52 kDa	3	0	0	0	1	0	0	0	0	0
Sorting nexin-1	SNX1_HUMAN	59 kDa	3	0	0	0	1	0	0	0	0	0
Tripartite motif-containing protein 2	TRIM2_HUMAN	82 kDa	4	0	0	0	0	0	0	0	0	0
Cytoplasmic dynein 1 light intermediate chain 1	DC1L1_HUMAN	57 kDa	4	0	0	0	0	0	0	0	0	0
Rho guanine nucleotide exchange factor 2	ARHG2_HUMAN	112 kDa	4	0	0	0	0	0	0	0	0	0
Neurogranin	NEUG_HUMAN	8 kDa	4	0	0	0	0	0	0	0	0	0
Plexin-A1	PLXA1_HUMAN	211 kDa	4	0	0	0	0	0	0	0	0	0
Heme oxygenase 2	HMOX2_HUMAN	36 kDa	4	0	0	0	0	0	0	0	0	0
Endophilin-A3	SH3G3_HUMAN	39 kDa	4	0	0	0	0	0	0	0	0	0
AP-3 complex subunit beta-2	AP3B2_HUMAN	119 kDa	4	0	0	0	0	0	0	0	0	0
Adenylate kinase isoenzyme 5	KAD5_HUMAN	63 kDa	4	0	0	0	0	0	0	0	0	0
V-type proton ATPase subunit G 2	VATG2_HUMAN	14 kDa	4	0	0	0	0	0	0	0	0	0
Ras-related protein Rap-2a	RAP2A_HUMAN	21 kDa	4	0	0	0	0	0	0	0	0	0
Rho-associated protein kinase 2	ROCK2_HUMAN	161 kDa	4	0	0	0	0	0	0	0	0	0
Microtubule-associated protein RP/EB family member 2	MARE2_HUMAN	37 kDa	4	0	0	0	0	0	0	0	0	0
Mast cell carboxypeptidase A	CBPA3_HUMAN	49 kDa	0	0	0	0	0	0	0	0	0	5
Inter-alpha-trypsin inhibitor heavy chain H1	ITIH1_HUMAN	101 kDa	0	0	0	0	0	0	0	0	1	4
Pterin-4-alpha-carbinolamine dehydratase	PHS_HUMAN	12 kDa	1	0	0	0	1	1	0	0	0	2
SUMO-conjugating enzyme UBC9	UBC9_HUMAN	18 kDa	0	2	0	0	1	0	0	0	1	1
Dystroglycan	DAG1_HUMAN	97 kDa	0	0	4	0	0	0	0	0	0	1
1,5-anhydro-D-fructose reductase	AKCL2_HUMAN	37 kDa	0	0	1	0	1	1	1	0	0	1
Complement factor B	CFAB_HUMAN	86 kDa	0	0	0	0	0	0	0	0	4	1
KH domain-containing, RNA-binding, signal transduction-associated protein 1	KHDR1_HUMAN	48 kDa	2	0	0	0	0	0	0	0	2	1
Dihydropyrimidinase-related protein 5	DPYL5_HUMAN	61 kDa	1	4	0	0	0	0	0	0	0	0
NADH dehydrogenase [ubiquinone] 1 alpha subcomplex subunit 7	NDUA7_HUMAN	13 kDa	1	3	1	0	0	0	0	0	0	0
LanC-like protein 1	LANC1_HUMAN	45 kDa	1	3	0	0	0	0	0	0	1	0
Protein tweety homolog 1	TTYH1_HUMAN	49 kDa	2	3	0	0	0	0	0	0	0	0
Ras GTPase-activating protein-binding protein 2	G3BP2_HUMAN	54 kDa	2	3	0	0	0	0	0	0	0	0
Guanine nucleotide-binding protein subunit beta-5	GBB5_HUMAN	44 kDa	2	3	0	0	0	0	0	0	0	0

Description	Accession	MW	Raw spectral counts									
			Frontal cortex	Cerebellum	Right ventricle	Mesentric lymph node	Liver	Pancreas	Proximal bile duct	Breast	Ovary	Clitoris
Ras-related protein Ral-A	RALA_HUMAN	24 kDa	2	3	0	0	0	0	0	0	0	0
Neutral cholesterol ester hydrolase 1	NCEH1_HUMAN	46 kDa	0	2	3	0	0	0	0	0	0	0
Palmitoyl-protein thioesterase 1	PPT1_HUMAN	34 kDa	2	2	1	0	0	0	0	0	0	0
Leukotriene A-4 hydrolase	LKHA4_HUMAN	69 kDa	0	2	0	0	0	1	0	0	2	0
Hepatoma-derived growth factor-related protein 2	HDGR2_HUMAN	74 kDa	0	2	0	0	0	0	0	0	3	0
Probable ATP-dependent RNA helicase DDX6	DDX6_HUMAN	54 kDa	0	2	0	0	0	0	0	0	3	0
Ras-related protein Ral-B	RALB_HUMAN	23 kDa	1	2	0	0	0	0	0	0	2	0
Complexin-1	CPLX1_HUMAN	15 kDa	3	2	0	0	0	0	0	0	0	0
DnaJ homolog subfamily C member 5	DNJC5_HUMAN	22 kDa	3	2	0	0	0	0	0	0	0	0
Phospholemman	PLM_HUMAN	10 kDa	0	1	2	0	1	0	0	0	1	0
NADH dehydrogenase [ubiquinone] 1 alpha subcomplex subunit 5	NDUA5_HUMAN	13 kDa	2	1	1	0	0	1	0	0	0	0
Clathrin light chain B	CLCB_HUMAN	25 kDa	3	1	1	0	0	0	0	0	0	0
ATP-dependent (S)-NAD(P)H-hydrate dehydratase	NNRD_HUMAN	37 kDa	0	1	0	0	1	2	0	0	1	0
RNA-binding protein 8A	RBM8A_HUMAN	20 kDa	0	1	0	0	1	0	0	0	3	0
Regulator of G-protein signaling 7	RGS7_HUMAN	58 kDa	4	1	0	0	0	0	0	0	0	0
C-terminal-binding protein 1	CTBP1_HUMAN	48 kDa	4	1	0	0	0	0	0	0	0	0
Neurocan core protein	NCAN_HUMAN	143 kDa	4	1	0	0	0	0	0	0	0	0
FXYD domain-containing ion transport regulator 6	FXYD6_HUMAN	11 kDa	4	1	0	0	0	0	0	0	0	0
Sarcolemmal membrane-associated protein	SLMAP_HUMAN	95 kDa	0	0	5	0	0	0	0	0	0	0
Heat shock protein beta-2	HSPB2_HUMAN	20 kDa	0	0	5	0	0	0	0	0	0	0
Ubiquinone biosynthesis protein COQ9	COQ9_HUMAN	36 kDa	0	0	4	0	0	1	0	0	0	0
cAMP-dependent protein kinase type I-alpha regulatory subunit	KAP0_HUMAN	43 kDa	0	0	4	0	0	1	0	0	0	0
2-methoxy-6-polyprenyl-1,4-benzoquinol methylase	COQ5_HUMAN	37 kDa	0	0	4	0	0	0	0	0	1	0
Laminin subunit gamma-1	LAMC1_HUMAN	178 kDa	0	0	4	1	0	0	0	0	0	0
Protein kinase C and casein kinase substrate in neurons protein 3	PACN3_HUMAN	48 kDa	0	0	4	0	1	0	0	0	0	0
Microtubule-associated protein RP/EB family member 1	MARE1_HUMAN	30 kDa	2	0	2	0	0	1	0	0	0	0
NSFL1 cofactor p47	NSF1C_HUMAN	41 kDa	0	0	2	0	0	0	0	0	3	0
Estradiol 17-beta-dehydrogenase 8	DHB8_HUMAN	27 kDa	0	0	2	0	3	0	0	0	0	0
Acylphosphatase-2	ACYP2_HUMAN	11 kDa	3	0	2	0	0	0	0	0	0	0
Tumor protein D54	TPD54_HUMAN	22 kDa	0	0	1	1	0	1	0	0	2	0
Isochorismatase domain-containing protein 2	ISOC2_HUMAN	22 kDa	0	0	1	0	2	1	0	0	1	0
Lipoamide acyltransferase component of branched-chain alpha-keto acid	ODB2_HUMAN	53 kDa	1	0	1	0	2	1	0	0	0	0
Alpha-2-HS-glycoprotein	FETUA_HUMAN	39 kDa	0	0	1	3	0	0	1	0	0	0
CD81 antigen	CD81_HUMAN	26 kDa	2	0	1	0	1	0	0	0	1	0
Hydroxymethylglutaryl-CoA lyase	HMGCL_HUMAN	34 kDa	0	0	1	0	4	0	0	0	0	0
DDRGK domain-containing protein 1	DDRGK_HUMAN	36 kDa	0	0	0	0	0	5	0	0	0	0
Vesicle transport protein GOT1B	GOT1B_HUMAN	15 kDa	0	0	0	0	0	5	0	0	0	0
Colipase	COL_HUMAN	12 kDa	0	0	0	0	0	5	0	0	0	0
Chymotrypsin-like protease CTRL-1	CTRL_HUMAN	28 kDa	0	0	0	0	0	5	0	0	0	0
Chymotrypsin-like elastase family member 1	CELA1_HUMAN	28 kDa	0	0	0	0	0	5	0	0	0	0
Aromatic-L-amino-acid decarboxylase	DDC_HUMAN	54 kDa	0	0	0	0	0	5	0	0	0	0
DnaJ homolog subfamily C member 3	DNJC3_HUMAN	58 kDa	0	0	0	0	0	5	0	0	0	0
Serine/arginine-rich splicing factor 6	SRSF6_HUMAN	40 kDa	0	0	0	0	0	4	0	0	1	0
Galactose-1-phosphate uridylyltransferase	GALT_HUMAN	43 kDa	0	0	0	0	1	4	0	0	0	0
Protein CutA	CUTA_HUMAN	19 kDa	1	0	0	0	0	4	0	0	0	0
Sec1 family domain-containing protein 1	SCFD1_HUMAN	72 kDa	0	0	0	0	0	3	0	0	2	0
ATPase ASNA1	ASNA_HUMAN	39 kDa	2	0	0	0	0	2	0	0	1	0
26S proteasome non-ATPase regulatory subunit 14	PSDE_HUMAN	35 kDa	2	0	0	0	0	2	0	0	1	0
Guanidinoacetate N-methyltransferase	GAMT_HUMAN	26 kDa	0	0	0	0	3	2	0	0	0	0
Retinol dehydrogenase 11	RDH11_HUMAN	35 kDa	0	0	0	0	3	2	0	0	0	0
Dehydrogenase/reductase SDR family member 1	DHRS1_HUMAN	34 kDa	0	0	0	0	3	2	0	0	0	0
Citrate lyase subunit beta-like protein	CLYBL_HUMAN	37 kDa	0	0	0	0	3	2	0	0	0	0

Description	Accession	MW	Raw spectral counts									
			Frontal cortex	Cerebellum	Right ventricle	Mesentric lymph node	Liver	Pancreas	Proximal bile duct	Breast	Ovary	Clitoris
Transmembrane protein 14C	TM14C_HUMAN	12 kDa	2	0	0	0	1	2	0	0	0	0
Serine/arginine-rich splicing factor 9	SRSF9_HUMAN	26 kDa	0	0	0	0	0	1	0	0	4	0
Ran-specific GTPase-activating protein	RANG_HUMAN	23 kDa	2	0	0	0	0	1	0	0	2	0
Aflatoxin B1 aldehyde reductase member 3	ARK73_HUMAN	37 kDa	0	0	0	0	4	1	0	0	0	0
Fibulin-5	FBLN5_HUMAN	50 kDa	0	0	0	2	0	0	2	0	1	0
Heat shock 70 kDa protein 12B	HS12B_HUMAN	76 kDa	0	0	0	0	0	0	0	0	5	0
Methyl-CpG-binding protein 2	MECP2_HUMAN	52 kDa	0	0	0	0	0	0	0	0	5	0
Collagen alpha-1(I) chain	CO1A1_HUMAN	139 kDa	0	0	0	0	0	0	0	0	5	0
60 kDa SS-A/Ro ribonucleoprotein	RO60_HUMAN	61 kDa	0	0	0	0	0	0	0	0	5	0
Fibromodulin	FMOD_HUMAN	43 kDa	0	0	0	0	0	0	0	0	5	0
Mitogen-activated protein kinase 3	MK03_HUMAN	43 kDa	3	0	0	0	0	0	0	0	2	0
Basement membrane-specific heparan sulfate proteoglycan core protein	PGBM_HUMAN	469 kDa	0	0	0	4	0	0	0	0	1	0
ATP-dependent 6-phosphofructokinase, liver type	PFKAL_HUMAN	85 kDa	2	0	0	0	2	0	0	0	1	0
Protein kinase C beta type	KPCB_HUMAN	77 kDa	4	0	0	0	0	0	0	0	1	0
Pyridoxal phosphate phosphatase	PLPP_HUMAN	32 kDa	4	0	0	0	0	0	0	0	1	0
Importin-7	IPO7_HUMAN	120 kDa	4	0	0	0	0	0	0	0	1	0
Immunoglobulin lambda-like polypeptide 1	IGLL1_HUMAN	23 kDa	0	0	0	5	0	0	0	0	0	0
Alanine--glyoxylate aminotransferase 2	AGT2_HUMAN	57 kDa	0	0	0	0	5	0	0	0	0	0
Alanine aminotransferase 1	ALAT1_HUMAN	55 kDa	0	0	0	0	5	0	0	0	0	0
Gamma-butyrobetaine dioxygenase	BODG_HUMAN	45 kDa	0	0	0	0	5	0	0	0	0	0
Oxygen-dependent coproporphyrinogen-III oxidase	HEM6_HUMAN	50 kDa	0	0	0	0	5	0	0	0	0	0
Formimidoyltransferase-cyclodeaminase	FTCD_HUMAN	59 kDa	0	0	0	0	5	0	0	0	0	0
Acyl-coenzyme A synthetase ACSM5	ACSM5_HUMAN	65 kDa	0	0	0	0	5	0	0	0	0	0
Transferrin receptor protein 2	TFR2_HUMAN	89 kDa	0	0	0	0	5	0	0	0	0	0
Cytochrome P450 2C19	CP2CJ_HUMAN	56 kDa	0	0	0	0	5	0	0	0	0	0
Cytosolic beta-glucosidase	GBA3_HUMAN	54 kDa	0	0	0	0	5	0	0	0	0	0
Solute carrier family 22 member 1	S22A1_HUMAN	61 kDa	0	0	0	0	5	0	0	0	0	0
Keratin, type I cuticular Ha4	KRT34_HUMAN	49 kDa	5	0	0	0	0	0	0	0	0	0
Drebrin	DREB_HUMAN	71 kDa	5	0	0	0	0	0	0	0	0	0
Neuron-specific calcium-binding protein hippocalcin	HPCA_HUMAN	22 kDa	5	0	0	0	0	0	0	0	0	0
Solute carrier family 2, facilitated glucose transporter member 1	GTR1_HUMAN	54 kDa	0	3	0	0	0	0	0	0	0	3
Atlastin-3	ATLA3_HUMAN	61 kDa	0	0	0	0	2	0	0	0	1	3
Arachidonate 15-lipoxygenase	LOX15_HUMAN	75 kDa	0	0	1	1	2	0	0	0	0	2
Calpain-2 catalytic subunit	CAN2_HUMAN	80 kDa	0	0	0	0	0	0	0	0	4	2
Serum amyloid P-component	SAMP_HUMAN	25 kDa	0	0	0	0	2	0	0	0	2	2
Phosphatidylinositol transfer protein	PIPNA_HUMAN	32 kDa	2	1	0	0	0	1	0	0	1	1
Ferrochelatase	HEMH_HUMAN	48 kDa	1	0	1	0	2	0	0	0	1	1
Cell surface glycoprotein MUC18	MUC18_HUMAN	72 kDa	0	0	0	2	0	0	3	0	0	1
26S protease regulatory subunit 7	PRS7_HUMAN	49 kDa	0	0	0	0	0	0	0	0	5	1
Serpin B9	SPB9_HUMAN	42 kDa	0	0	0	0	0	0	0	0	5	1
Probable ATP-dependent RNA helicase DDX5	DDX5_HUMAN	69 kDa	0	6	0	0	0	0	0	0	0	0
Carbonic anhydrase-related protein	CAH8_HUMAN	33 kDa	0	6	0	0	0	0	0	0	0	0
Hyaluronan and proteoglycan link protein 2	HPLN2_HUMAN	38 kDa	0	6	0	0	0	0	0	0	0	0
RNA-binding protein FUS	FUS_HUMAN	53 kDa	0	4	0	0	0	0	0	0	2	0
cAMP-dependent protein kinase catalytic subunit beta	KAPCB_HUMAN	41 kDa	1	3	0	1	1	0	0	0	0	0
Syntaxin-12	STX12_HUMAN	32 kDa	3	3	0	0	0	0	0	0	0	0
F-box/LRR-repeat protein 19	FXL19_HUMAN	76 kDa	1	2	3	0	0	0	0	0	0	0
T-complex protein 1 subunit alpha	TCPA_HUMAN	60 kDa	1	2	1	0	0	0	0	0	2	0
Cytochrome b5 type B	CYB5B_HUMAN	16 kDa	1	2	1	0	2	0	0	0	0	0
Programmed cell death protein 6	PDCD6_HUMAN	22 kDa	0	2	0	0	1	0	0	0	3	0
Glia maturation factor beta	GMFB_HUMAN	17 kDa	3	2	0	0	1	0	0	0	0	0
Tubulin alpha chain-like 3	TBAL3_HUMAN	50 kDa	4	2	0	0	0	0	0	0	0	0

Description	Accession	MW	Raw spectral counts									
			Frontal cortex	Cerebellum	Right ventricle	Mesentric lymph node	Liver	Pancreas	Proximal bile duct	Breast	Ovary	Clitoris
V-type proton ATPase subunit S1	VAS1_HUMAN	52 kDa	4	2	0	0	0	0	0	0	0	0
Serine/threonine-protein kinase PAK 1	PAK1_HUMAN	61 kDa	4	2	0	0	0	0	0	0	0	0
Sorting nexin-3	SNX3_HUMAN	19 kDa	1	1	2	0	1	0	0	0	1	0
Endothelial differentiation-related factor 1	EDF1_HUMAN	16 kDa	0	1	1	0	0	1	0	0	3	0
Transcription factor A	TFAM_HUMAN	29 kDa	1	1	1	0	1	1	0	0	1	0
Coronin-1A	COR1A_HUMAN	51 kDa	4	1	0	0	0	0	1	0	0	0
Microtubule-associated protein 6	MAP6_HUMAN	87 kDa	5	1	0	0	0	0	0	0	0	0
Alpha-1-syntrophin	SNTA1_HUMAN	54 kDa	0	0	6	0	0	0	0	0	0	0
PDZ and LIM domain protein 5	PDLI5_HUMAN	64 kDa	0	0	6	0	0	0	0	0	0	0
Methylcrotonoyl-CoA carboxylase beta chain	MCCB_HUMAN	61 kDa	0	0	4	0	1	1	0	0	0	0
Copper transport protein ATOX1	ATOX1_HUMAN	7 kDa	0	0	2	0	2	2	0	0	0	0
Cytospin-B	CYTSB_HUMAN	119 kDa	2	0	1	0	3	0	0	0	0	0
Transmembrane protein 214	TM214_HUMAN	77 kDa	0	0	0	0	0	6	0	0	0	0
ERO1-like protein beta	ERO1B_HUMAN	54 kDa	0	0	0	0	0	6	0	0	0	0
Protein sel-1 homolog 1	SE1L1_HUMAN	89 kDa	0	0	0	0	0	6	0	0	0	0
Translocon-associated protein subunit gamma	SSRG_HUMAN	21 kDa	0	0	0	0	0	6	0	0	0	0
Serpin B6	SPB6_HUMAN	43 kDa	0	0	0	0	0	5	0	0	1	0
40S ribosomal protein S15	RS15_HUMAN	17 kDa	0	0	0	0	0	4	0	0	2	0
Glutaredoxin-3	GLRX3_HUMAN	37 kDa	0	0	0	0	1	3	0	0	2	0
40S ribosomal protein S11	RS11_HUMAN	18 kDa	0	0	0	0	1	2	0	0	3	0
Unconventional myosin-XVIIIa	MY18A_HUMAN	233 kDa	2	0	0	0	2	2	0	0	0	0
Transmembrane protein 205	TM205_HUMAN	21 kDa	0	0	0	0	5	1	0	0	0	0
Serine/threonine-protein phosphatase PGAM5	PGAM5_HUMAN	32 kDa	0	0	0	0	4	0	2	0	0	0
Glyoxylate reductase/hydroxypyruvate reductase	GRHPR_HUMAN	36 kDa	0	0	0	0	3	0	1	0	2	0
Core histone macro-H2A.2	H2AW_HUMAN	40 kDa	0	0	0	0	0	0	0	0	6	0
Kinesin light chain 1	KLC1_HUMAN	65 kDa	5	0	0	0	0	0	0	0	1	0
Growth factor receptor-bound protein 2	GRB2_HUMAN	25 kDa	5	0	0	0	0	0	0	0	1	0
Alcohol dehydrogenase 6	ADH6_HUMAN	39 kDa	0	0	0	0	6	0	0	0	0	0
Putative dehydrogenase/reductase SDR family member 4-like 2	DR4L1_HUMAN	31 kDa	0	0	0	0	6	0	0	0	0	0
Pyruvate kinase PKLR	KPYR_HUMAN	62 kDa	0	0	0	0	6	0	0	0	0	0
Ectonucleoside triphosphate diphosphohydrolase 5	ENTP5_HUMAN	48 kDa	0	0	0	0	6	0	0	0	0	0
4-hydroxy-2-oxoglutarate aldolase	HOGA1_HUMAN	35 kDa	0	0	0	0	6	0	0	0	0	0
Peroxisomal membrane protein 2	PXMP2_HUMAN	22 kDa	0	0	0	0	6	0	0	0	0	0
Bile salt sulfotransferase	ST2A1_HUMAN	34 kDa	0	0	0	0	6	0	0	0	0	0
Isopentenyl-diphosphate Delta-isomerase 1	IDI1_HUMAN	26 kDa	0	0	0	0	6	0	0	0	0	0
Lambda-crystallin homolog	CRYL1_HUMAN	35 kDa	0	0	0	0	6	0	0	0	0	0
Nicotinate phosphoribosyltransferase	PNCB_HUMAN	58 kDa	0	0	0	0	6	0	0	0	0	0
Keratin, type I cytoskeletal 24	K1C24_HUMAN	55 kDa	6	0	0	0	0	0	0	0	0	0
V-type proton ATPase subunit F	VATF_HUMAN	13 kDa	6	0	0	0	0	0	0	0	0	0
Adenylyl cyclase-associated protein 2	CAP2_HUMAN	53 kDa	6	0	0	0	0	0	0	0	0	0
Ig gamma-2 chain C region	IGHG2_HUMAN	36 kDa	0	0	0	0	0	0	1	0	0	6
Periplakin	PEPL_HUMAN	205 kDa	0	0	0	0	1	0	0	0	0	6
PDZ and LIM domain protein 1	PDLI1_HUMAN	36 kDa	0	0	4	0	0	0	0	0	0	3
Fibrillin-1	FBN1_HUMAN	312 kDa	0	0	0	3	0	0	1	0	0	3
Translin-associated protein X	TSNAX_HUMAN	33 kDa	1	0	0	0	0	2	0	0	2	2
Proteasome subunit beta type-7	PSB7_HUMAN	30 kDa	2	0	0	0	3	0	0	0	0	2
Phospholipid hydroperoxide glutathione peroxidase	GPX4_HUMAN	22 kDa	0	3	0	0	3	0	0	0	0	1
Calcium-binding protein 39	CAB39_HUMAN	40 kDa	0	2	0	0	0	2	0	0	2	1
NADH-cytochrome b5 reductase 1	NB5R1_HUMAN	34 kDa	2	1	2	0	0	1	0	0	0	1
NAD(P)H-hydrate epimerase	NNRE_HUMAN	32 kDa	1	1	2	0	0	0	0	0	2	1
Transforming protein RhoA	RHOA_HUMAN	22 kDa	2	1	0	0	1	1	0	0	1	1
Alpha-parvin	PARVA_HUMAN	42 kDa	0	0	1	1	1	0	1	0	2	1

Description	Accession	MW	Raw spectral counts									
			Frontal cortex	Cerebellum	Right ventricle	Mesentric lymph node	Liver	Pancreas	Proximal bile duct	Breast	Ovary	Clitoris
Eukaryotic translation initiation factor 3 subunit F	EIF3F_HUMAN	38 kDa	0	0	0	0	0	3	0	0	3	1
26S protease regulatory subunit 8	PRS8_HUMAN	46 kDa	0	0	0	0	3	0	0	0	3	1
Synaptojanin-1	SYNJ1_HUMAN	173 kDa	2	5	0	0	0	0	0	0	0	0
Aquaporin-4	AQP4_HUMAN	35 kDa	3	4	0	0	0	0	0	0	0	0
Ras-related protein Rab-3C	RAB3C_HUMAN	26 kDa	3	4	0	0	0	0	0	0	0	0
Guanine nucleotide-binding protein G(z) subunit alpha	GNAZ_HUMAN	41 kDa	4	3	0	0	0	0	0	0	0	0
Synaptic vesicle glycoprotein 2B	SV2B_HUMAN	77 kDa	4	3	0	0	0	0	0	0	0	0
Limbic system-associated membrane protein	LSAMP_HUMAN	37 kDa	5	2	0	0	0	0	0	0	0	0
Protein RUFY3	RUFY3_HUMAN	53 kDa	5	2	0	0	0	0	0	0	0	0
Protein bassoon	BSN_HUMAN	416 kDa	5	2	0	0	0	0	0	0	0	0
Prefoldin subunit 3	PFD3_HUMAN	23 kDa	4	1	1	0	0	1	0	0	0	0
Diablo homolog	DBLOH_HUMAN	27 kDa	1	1	0	0	0	3	0	0	2	0
Eukaryotic translation initiation factor 5	IF5_HUMAN	49 kDa	3	1	0	0	0	2	0	0	1	0
SH3-containing GRB2-like protein 3-interacting protein 1	SGIP1_HUMAN	89 kDa	6	1	0	0	0	0	0	0	0	0
Neuronal calcium sensor 1	NCS1_HUMAN	22 kDa	6	1	0	0	0	0	0	0	0	0
Plakophilin-2	PKP2_HUMAN	97 kDa	0	0	7	0	0	0	0	0	0	0
Carnitine O-palmitoyltransferase 1, muscle isoform	CPT1B_HUMAN	88 kDa	0	0	7	0	0	0	0	0	0	0
Apolipoprotein A-IV	APOA4_HUMAN	45 kDa	0	0	4	1	0	0	1	0	1	0
Peptidyl-prolyl cis-trans isomerase F	PPIF_HUMAN	22 kDa	0	0	3	0	4	0	0	0	0	0
Branched-chain-amino-acid aminotransferase	BCAT2_HUMAN	44 kDa	0	0	2	0	0	4	0	0	1	0
NEDD8	NEDD8_HUMAN	9 kDa	1	0	2	0	1	2	1	0	0	0
Prefoldin subunit 2	PFD2_HUMAN	17 kDa	2	0	2	0	0	2	0	0	1	0
Hsp90 co-chaperone Cdc37	CDC37_HUMAN	44 kDa	1	0	2	0	0	0	0	0	4	0
GrpE protein homolog 1	GRPE1_HUMAN	24 kDa	1	0	2	0	3	0	0	0	1	0
All-trans-retinol 13,14-reductase	RETST_HUMAN	67 kDa	0	0	2	1	4	0	0	0	0	0
Carnitine O-acetyltransferase	CACP_HUMAN	71 kDa	0	0	2	0	5	0	0	0	0	0
Eukaryotic translation initiation factor 3 subunit J	EIF3J_HUMAN	29 kDa	2	0	1	0	0	4	0	0	0	0
40S ribosomal protein S5	RS5_HUMAN	23 kDa	1	0	1	0	2	3	0	0	0	0
Guanine nucleotide-binding protein subunit alpha-11	GNA11_HUMAN	42 kDa	3	0	1	0	0	3	0	0	0	0
DnaJ homolog subfamily A member 2	DNJA2_HUMAN	46 kDa	2	0	1	0	2	0	0	0	2	0
Nucleobindin-2	NUCB2_HUMAN	50 kDa	0	0	0	0	0	7	0	0	0	0
Secretagogin	SEGN_HUMAN	32 kDa	0	0	0	0	0	7	0	0	0	0
Chymotrypsin-C	CTRC_HUMAN	29 kDa	0	0	0	0	0	7	0	0	0	0
Signal peptidase complex subunit 3	SPCS3_HUMAN	20 kDa	0	0	0	0	1	6	0	0	0	0
Desmoglein-1	DSG1_HUMAN	114 kDa	0	0	0	0	1	6	0	0	0	0
40S ribosomal protein S23	RS23_HUMAN	16 kDa	0	0	0	0	1	5	0	0	1	0
ADP-ribosylation factor-like protein 1	ARL1_HUMAN	20 kDa	0	0	0	0	2	5	0	0	0	0
Translational activator GCN1	GCN1L_HUMAN	293 kDa	0	0	0	0	2	5	0	0	0	0
Coatomer subunit zeta-1	COPZ1_HUMAN	20 kDa	0	0	0	0	2	5	0	0	0	0
ADP-ribosylation factor-like protein 3	ARL3_HUMAN	20 kDa	2	0	0	0	0	1	0	0	4	0
Bifunctional 3'-phosphoadenosine 5'-phosphosulfate synthase 2	PAPS2_HUMAN	70 kDa	0	0	0	0	3	1	0	0	3	0
Sterol-4-alpha-carboxylate 3-dehydrogenase, decarboxylating	NSDHL_HUMAN	42 kDa	0	0	0	0	6	1	0	0	0	0
Galectin-4	LEG4_HUMAN	36 kDa	0	0	0	0	0	0	7	0	0	0
Zona pellucida sperm-binding protein 4	ZP4_HUMAN	59 kDa	0	0	0	0	0	0	0	0	7	0
Ras-related protein Rap-2b	RAP2B_HUMAN	21 kDa	4	0	0	0	0	0	0	0	3	0
Glutathione S-transferase theta-1	GSTT1_HUMAN	27 kDa	0	0	0	0	5	0	0	0	2	0
Cytochrome P450 2C8	CP2C8_HUMAN	56 kDa	0	0	0	0	7	0	0	0	0	0
Cytochrome P450 3A5	CP3A5_HUMAN	57 kDa	0	0	0	0	7	0	0	0	0	0
Peroxisomal sarcosine oxidase	SOX_HUMAN	44 kDa	0	0	0	0	7	0	0	0	0	0
Cytochrome P450 4A11	CP4AB_HUMAN	59 kDa	0	0	0	0	7	0	0	0	0	0
Serine/threonine-protein kinase DCLK1	DCLK1_HUMAN	82 kDa	7	0	0	0	0	0	0	0	0	0
Oxidation resistance protein 1	OXR1_HUMAN	98 kDa	7	0	0	0	0	0	0	0	0	0

Description	Accession	MW	Raw spectral counts									
			Frontal cortex	Cerebellum	Right ventricle	Mesentric lymph node	Liver	Pancreas	Proximal bile duct	Breast	Ovary	Clitoris
CD109 antigen	CD109_HUMAN	162 kDa	0	0	0	0	0	0	0	0	0	8
Band 3 anion transport protein	B3AT_HUMAN	102 kDa	0	0	1	0	0	0	2	0	0	5
Isochorismatase domain-containing protein 1	ISOC1_HUMAN	32 kDa	0	0	2	0	1	1	0	0	1	3
Barrier-to-autointegration factor	BAF_HUMAN	10 kDa	0	0	1	0	1	1	1	0	1	3
Beta-2-glycoprotein 1	APOH_HUMAN	38 kDa	0	0	1	0	0	0	4	0	0	3
Integrin-linked protein kinase	ILK_HUMAN	51 kDa	0	0	1	1	0	0	2	0	2	2
Tetranectin	TETN_HUMAN	23 kDa	0	0	0	2	0	0	0	0	4	2
Acylglycerol kinase	AGK_HUMAN	47 kDa	0	3	1	0	1	1	0	0	1	1
Vacuolar protein sorting-associated protein 29	VPS29_HUMAN	21 kDa	2	2	0	0	1	1	0	0	1	1
Band 4.1-like protein 2	E41L2_HUMAN	113 kDa	2	2	0	0	0	0	0	0	3	1
Monoglyceride lipase	MGLL_HUMAN	33 kDa	3	2	0	2	0	0	0	0	0	1
Signal peptidase complex catalytic subunit SEC11A	SC11A_HUMAN	21 kDa	0	0	0	0	2	3	0	0	2	1
Cullin-5	CUL5_HUMAN	91 kDa	2	4	1	0	1	0	0	0	0	0
Secernin-1	SCRN1_HUMAN	46 kDa	4	4	0	0	0	0	0	0	0	0
NAD-dependent protein deacetylase sirtuin-2	SIR2_HUMAN	43 kDa	4	4	0	0	0	0	0	0	0	0
Proline-rich transmembrane protein 2	PRRT2_HUMAN	35 kDa	4	4	0	0	0	0	0	0	0	0
Gamma-synuclein	SYUG_HUMAN	13 kDa	4	4	0	0	0	0	0	0	0	0
Dihydropyrimidinase-related protein 4	DPYL4_HUMAN	62 kDa	4	4	0	0	0	0	0	0	0	0
Protein lin-7 homolog A	LIN7A_HUMAN	26 kDa	4	4	0	0	0	0	0	0	0	0
PC4 and SFRS1-interacting protein	PSIP1_HUMAN	60 kDa	0	3	0	0	0	0	0	0	5	0
NADH dehydrogenase [ubiquinone] 1 alpha subcomplex subunit 6	NDUA6_HUMAN	18 kDa	2	2	2	0	1	0	0	0	1	0
Activated RNA polymerase II transcriptional coactivator p15	TCP4_HUMAN	14 kDa	1	2	1	0	0	1	0	0	3	0
U1 small nuclear ribonucleoprotein A	SNRPA_HUMAN	31 kDa	1	2	0	0	0	1	0	0	4	0
Neuronal cell adhesion molecule	NRCAM_HUMAN	144 kDa	7	1	0	0	0	0	0	0	0	0
NADP-dependent malic enzyme	MAON_HUMAN	67 kDa	7	1	0	0	0	0	0	0	0	0
NLR family member X1	NLRX1_HUMAN	108 kDa	0	0	8	0	0	0	0	0	0	0
Protein-glutamine gamma-glutamyltransferase 2	TGM2_HUMAN	77 kDa	0	0	7	0	1	0	0	0	0	0
Ryanodine receptor 2	RYR2_HUMAN	565 kDa	1	0	7	0	0	0	0	0	0	0
Cytochrome c oxidase subunit 6B1	CX6B1_HUMAN	10 kDa	2	0	5	0	0	1	0	0	0	0
NADH dehydrogenase [ubiquinone] 1 alpha subcomplex subunit 2	NDUA2_HUMAN	11 kDa	2	0	3	0	1	2	0	0	0	0
Proline synthase co-transcribed bacterial homolog protein	PROSC_HUMAN	30 kDa	1	0	3	0	2	1	0	0	1	0
Polyadenylate-binding protein 4	PABP4_HUMAN	71 kDa	0	0	0	0	0	8	0	0	0	0
Serine/threonine-protein phosphatase PP1-alpha catalytic subunit	PP1A_HUMAN	38 kDa	0	0	0	0	0	8	0	0	0	0
Signal recognition particle subunit SRP68	SRP68_HUMAN	71 kDa	0	0	0	0	0	7	0	0	1	0
DnaJ homolog subfamily B member 11	DJB11_HUMAN	41 kDa	0	0	0	0	1	7	0	0	0	0
Isoleucine--tRNA ligase, cytoplasmic	SYIC_HUMAN	145 kDa	2	0	0	0	0	6	0	0	0	0
S-methyl-5'-thioadenosine phosphorylase	MTAP_HUMAN	31 kDa	0	0	0	0	1	4	0	0	3	0
Receptor expression-enhancing protein 6	REEP6_HUMAN	21 kDa	0	0	0	0	4	4	0	0	0	0
Putative hydroxypyruvate isomerase	HYI_HUMAN	30 kDa	0	0	0	0	0	3	0	0	5	0
Glutathione S-transferase A5	GSTA5_HUMAN	26 kDa	0	0	0	0	6	2	0	0	0	0
Phenazine biosynthesis-like domain-containing protein	PBLD_HUMAN	32 kDa	0	0	0	0	7	1	0	0	0	0
Cytochrome P450 2C18	CP2CI_HUMAN	56 kDa	0	0	0	0	7	0	1	0	0	0
Retinol-binding protein 1	RET1_HUMAN	16 kDa	0	0	0	0	1	0	0	0	7	0
Microsomal glutathione S-transferase 1	MGST1_HUMAN	18 kDa	0	0	0	2	6	0	0	0	0	0
Phenylalanine-4-hydroxylase	PH4H_HUMAN	52 kDa	0	0	0	0	8	0	0	0	0	0
Bile acid-CoA:amino acid N-acyltransferase	BAAT_HUMAN	46 kDa	0	0	0	0	8	0	0	0	0	0
Dimethylaniline monooxygenase [N-oxide-forming] 3	FMO3_HUMAN	60 kDa	0	0	0	0	8	0	0	0	0	0
Peroxisomal 2,4-dienoyl-CoA reductase	DECR2_HUMAN	31 kDa	0	0	0	0	8	0	0	0	0	0
GDH/6PGL endoplasmic bifunctional protein	G6PE_HUMAN	89 kDa	0	0	0	0	8	0	0	0	0	0
Glycine N-acyltransferase	GLYAT_HUMAN	34 kDa	0	0	0	0	8	0	0	0	0	0
Cocaine esterase	EST2_HUMAN	62 kDa	0	0	0	0	8	0	0	0	0	0
Aldehyde dehydrogenase, dimeric NADP-preferring	AL3A1_HUMAN	50 kDa	0	0	0	0	0	0	0	0	0	9

Description	Accession	MW	Raw spectral counts									
			Frontal cortex	Cerebellum	Right ventricle	Mesentric lymph node	Liver	Pancreas	Proximal bile duct	Breast	Ovary	Clitoris
Alcohol dehydrogenase class 4 mu/sigma chain	ADH7_HUMAN	41 kDa	0	0	0	0	0	0	3	0	0	6
Gamma-adducin	ADDG_HUMAN	79 kDa	0	1	0	0	0	0	0	0	5	3
Fibrinogen gamma chain	FIBG_HUMAN	52 kDa	0	0	1	0	0	0	4	0	1	3
5'-nucleotidase	5NTD_HUMAN	63 kDa	0	0	0	0	1	0	0	0	6	2
Tropomodulin-2	TMOD2_HUMAN	40 kDa	6	2	0	0	0	0	0	0	0	1
Protein S100-B	S100B_HUMAN	11 kDa	3	0	0	2	0	0	3	0	0	1
Fatty acid-binding protein, brain	FABP7_HUMAN	15 kDa	2	7	0	0	0	0	0	0	0	0
Ganglioside-induced differentiation-associated protein 1	GDAP1_HUMAN	41 kDa	3	6	0	0	0	0	0	0	0	0
Neuroplastin	NPTN_HUMAN	44 kDa	3	6	0	0	0	0	0	0	0	0
Endophilin-B2	SHLB2_HUMAN	44 kDa	4	5	0	0	0	0	0	0	0	0
Septin-5	SEPT5_HUMAN	43 kDa	5	4	0	0	0	0	0	0	0	0
Dipeptidyl aminopeptidase-like protein 6	DPP6_HUMAN	98 kDa	5	4	0	0	0	0	0	0	0	0
NADH dehydrogenase [ubiquinone] iron-sulfur protein 7	NDUS7_HUMAN	24 kDa	0	3	6	0	0	0	0	0	0	0
High mobility group protein HMG-I/HMG-Y	HMGA1_HUMAN	12 kDa	1	3	0	0	0	2	0	0	3	0
Neuromodulin	NEUM_HUMAN	25 kDa	6	3	0	0	0	0	0	0	0	0
Mitochondrial pyruvate carrier 2	MPC2_HUMAN	14 kDa	0	2	5	0	2	0	0	0	0	0
NADH dehydrogenase [ubiquinone] iron-sulfur protein 4	NDUS4_HUMAN	20 kDa	1	2	3	0	2	0	0	0	1	0
Estradiol 17-beta-dehydrogenase 12	DHB12_HUMAN	34 kDa	1	1	2	0	3	2	0	0	0	0
Metaxin-2	MTX2_HUMAN	30 kDa	2	1	2	0	2	2	0	0	0	0
CDP-diacylglycerol--inositol 3-phosphatidyltransferase	CDIPT_HUMAN	24 kDa	0	1	0	0	0	6	1	0	1	0
NADH dehydrogenase [ubiquinone] 1 alpha subcomplex subunit 4	NDUA4_HUMAN	9 kDa	0	0	9	0	0	0	0	0	0	0
Ezrin	EZRI_HUMAN	69 kDa	0	0	3	0	0	6	0	0	0	0
Alpha-aminoadipic semialdehyde synthase	AASS_HUMAN	102 kDa	0	0	3	0	6	0	0	0	0	0
Single-stranded DNA-binding protein	SSBP_HUMAN	17 kDa	3	0	1	0	1	2	0	0	2	0
Aminoacylase-1	ACY1_HUMAN	46 kDa	0	0	1	1	6	0	0	0	1	0
Maleylacetoacetate isomerase	MAAI_HUMAN	24 kDa	0	0	1	0	8	0	0	0	0	0
Protein PRRC1	PRRC1_HUMAN	47 kDa	0	0	0	0	0	9	0	0	0	0
Pancreatic secretory granule membrane major glycoprotein GP2	GP2_HUMAN	59 kDa	0	0	0	0	0	9	0	0	0	0
Zymogen granule membrane protein 16	ZG16_HUMAN	18 kDa	0	0	0	0	0	9	0	0	0	0
Threonine--tRNA ligase, cytoplasmic	SYTC_HUMAN	83 kDa	0	0	0	0	0	9	0	0	0	0
Nodal modulator 1	NOMO1_HUMAN	134 kDa	0	0	0	0	0	8	1	0	0	0
Eukaryotic translation initiation factor 5B	IF2P_HUMAN	139 kDa	0	0	0	0	0	7	0	0	2	0
Aldose 1-epimerase	GALM_HUMAN	38 kDa	0	0	0	0	3	5	0	0	1	0
Vesicular integral-membrane protein VIP36	LMAN2_HUMAN	40 kDa	0	0	0	0	4	5	0	0	0	0
U2 small nuclear ribonucleoprotein A'	RU2A_HUMAN	28 kDa	0	0	0	0	0	4	0	0	5	0
Acyl-coenzyme A synthetase ACSM3	ACSM3_HUMAN	66 kDa	0	0	0	0	5	4	0	0	0	0
SPRY domain-containing protein 4	SPRY4_HUMAN	23 kDa	0	0	0	0	6	3	0	0	0	0
Guanine nucleotide-binding protein G(s) subunit s XLas	GNAS1_HUMAN	111 kDa	0	0	0	0	0	2	0	0	7	0
Ethanolamine-phosphate cytidylyltransferase	PCY2_HUMAN	44 kDa	0	0	0	0	8	1	0	0	0	0
Integrin alpha-1	ITA1_HUMAN	131 kDa	0	0	0	1	5	0	3	0	0	0
CD166 antigen	CD166_HUMAN	65 kDa	3	0	0	0	2	0	0	0	4	0
Leukotriene-B(4) omega-hydroxylase 1	CP4F2_HUMAN	60 kDa	0	0	0	0	9	0	0	0	0	0
Bile acyl-CoA synthetase	S27A5_HUMAN	75 kDa	0	0	0	0	9	0	0	0	0	0
Serum paraoxonase/lactonase 3	PON3_HUMAN	40 kDa	0	0	0	0	9	0	0	0	0	0
Aldehyde dehydrogenase family 8 member A1	AL8A1_HUMAN	53 kDa	0	0	0	0	9	0	0	0	0	0
S-methylmethionine--homocysteine S-methyltransferase BHMT2	BHMT2_HUMAN	40 kDa	0	0	0	0	9	0	0	0	0	0
Cytochrome P450 4F11	CP4FB_HUMAN	60 kDa	0	0	0	0	9	0	0	0	0	0
Cell adhesion molecule 2	CADM2_HUMAN	48 kDa	9	0	0	0	0	0	0	0	0	0
Brevican core protein	PGCB_HUMAN	99 kDa	9	0	0	0	0	0	0	0	0	0
CaM kinase-like vesicle-associated protein	CAMKV_HUMAN	54 kDa	9	0	0	0	0	0	0	0	0	0
Plakophilin-1	PKP1_HUMAN	83 kDa	0	0	0	0	0	0	0	0	0	10
Guanine nucleotide-binding protein G(I)/G(S)/G(O) subunit gamma-12	GBG12_HUMAN	8 kDa	1	0	0	1	0	2	2	0	0	4

Description	Accession	MW	Raw spectral counts									
			Frontal cortex	Cerebellum	Right ventricle	Mesentric lymph node	Liver	Pancreas	Proximal bile duct	Breast	Ovary	Clitoris
Phosphatidylinositol transfer protein	PIPNB_HUMAN	32 kDa	1	0	0	0	0	2	2	0	2	3
Peptidyl-prolyl cis-trans isomerase NIMA-interacting 1	PIN1_HUMAN	18 kDa	2	4	1	0	0	1	0	0	1	1
Guanine nucleotide-binding protein G(q) subunit alpha	GNAQ_HUMAN	42 kDa	5	3	0	0	0	1	0	0	0	1
Kinectin	KTN1_HUMAN	156 kDa	0	0	2	0	2	1	0	0	4	1
Aspartyl aminopeptidase	DNPEP_HUMAN	52 kDa	0	0	1	0	4	0	0	0	4	1
rRNA 2'-O-methyltransferase fibrillarin	FBRL_HUMAN	34 kDa	0	0	0	0	0	3	0	0	6	1
Nicotinate-nucleotide pyrophosphorylase [carboxylating]	NADC_HUMAN	31 kDa	0	0	0	0	5	0	1	0	3	1
Electrogenic sodium bicarbonate cotransporter 1	S4A4_HUMAN	121 kDa	0	10	0	0	0	0	0	0	0	0
Microtubule-associated protein RP/EB family member 3	MARE3_HUMAN	32 kDa	5	5	0	0	0	0	0	0	0	0
Heterogeneous nuclear ribonucleoprotein A0	ROA0_HUMAN	31 kDa	0	4	0	0	0	0	0	0	6	0
Tyrosine-protein phosphatase non-receptor type substrate 1	SHPS1_HUMAN	55 kDa	6	4	0	0	0	0	0	0	0	0
Protein NipSnap homolog 1	NIPS1_HUMAN	33 kDa	0	3	0	0	5	0	0	0	2	0
Rap1 GTPase-GDP dissociation stimulator 1	GDS1_HUMAN	66 kDa	7	3	0	0	0	0	0	0	0	0
V-type proton ATPase subunit H	VATH_HUMAN	56 kDa	7	3	0	0	0	0	0	0	0	0
Neuronal-specific septin-3	SEPT3_HUMAN	41 kDa	7	3	0	0	0	0	0	0	0	0
Protein FAM49B	FA49B_HUMAN	37 kDa	7	3	0	0	0	0	0	0	0	0
4F2 cell-surface antigen heavy chain	4F2_HUMAN	68 kDa	7	3	0	0	0	0	0	0	0	0
Serine/threonine-protein phosphatase 2A activator	PTPA_HUMAN	41 kDa	1	2	0	0	0	3	1	0	3	0
Nuclear migration protein nudC	NUDC_HUMAN	38 kDa	0	2	0	0	1	1	0	0	6	0
Purine nucleoside phosphorylase	PNPH_HUMAN	32 kDa	0	2	0	0	8	0	0	0	0	0
Nebulette	NEBL_HUMAN	116 kDa	0	1	9	0	0	0	0	0	0	0
Cytochrome b-c1 complex subunit 7	QCR7_HUMAN	14 kDa	2	1	3	0	2	2	0	0	0	0
Acyl-CoA dehydrogenase family member 9	ACAD9_HUMAN	69 kDa	3	1	3	0	3	0	0	0	0	0
Calcyclin-binding protein	CYBP_HUMAN	26 kDa	2	1	1	0	0	1	1	0	4	0
Myozenin-2	MYOZ2_HUMAN	30 kDa	0	0	10	0	0	0	0	0	0	0
Basal cell adhesion molecule	BCAM_HUMAN	67 kDa	0	0	5	0	0	4	1	0	0	0
Inorganic pyrophosphatase 2	IPYR2_HUMAN	38 kDa	1	0	3	0	2	4	0	0	0	0
Talin-2	TLN2_HUMAN	272 kDa	7	0	3	0	0	0	0	0	0	0
Voltage-dependent calcium channel subunit alpha-2/delta-1	CA2D1_HUMAN	125 kDa	8	0	1	0	0	0	0	0	1	0
Glutamine--fructose-6-phosphate aminotransferase [isomerizing] 1	GFPT1_HUMAN	79 kDa	0	0	0	0	0	10	0	0	0	0
Signal recognition particle subunit SRP72	SRP72_HUMAN	75 kDa	0	0	0	0	0	8	0	0	2	0
ER lumen protein-retaining receptor 2	ERD22_HUMAN	24 kDa	0	0	0	0	5	5	0	0	0	0
Methionine adenosyltransferase 2 subunit beta	MAT2B_HUMAN	38 kDa	0	0	0	0	0	4	1	0	5	0
17-beta-hydroxysteroid dehydrogenase 13	DHB13_HUMAN	34 kDa	0	0	0	0	8	2	0	0	0	0
Serine--pyruvate aminotransferase	SPYA_HUMAN	43 kDa	0	0	0	0	9	1	0	0	0	0
Prostacyclin synthase	PTGIS_HUMAN	57 kDa	0	0	0	4	0	0	4	0	2	0
Galactokinase	GALK1_HUMAN	42 kDa	0	0	0	0	9	0	0	0	1	0
Leukotriene-B(4) omega-hydroxylase 2	CP4F3_HUMAN	60 kDa	0	0	0	0	10	0	0	0	0	0
Corticosteroid 11-beta-dehydrogenase isozyme 1	DHI1_HUMAN	32 kDa	0	0	0	0	10	0	0	0	0	0
Glycine dehydrogenase (decarboxylating)	GCSP_HUMAN	113 kDa	0	0	0	0	10	0	0	0	0	0
Acyl-CoA synthetase short-chain family member 3	ACSS3_HUMAN	75 kDa	0	0	0	0	10	0	0	0	0	0
Putative keratin-87 protein	KR87P_HUMAN	29 kDa	10	0	0	0	0	0	0	0	0	0
Paralemmin-1	PALM_HUMAN	42 kDa	10	0	0	0	0	0	0	0	0	0
Actin-related protein 2/3 complex subunit 3	ARPC3_HUMAN	21 kDa	3	1	0	0	2	1	1	0	2	1
Sodium/calcium exchanger 2	NAC2_HUMAN	100 kDa	3	8	0	0	0	0	0	0	0	0
LETM1 and EF-hand domain-containing protein 1	LETM1_HUMAN	83 kDa	4	4	3	0	0	0	0	0	0	0
Ras-related protein Rab-6A	RAB6A_HUMAN	24 kDa	3	4	0	0	0	0	0	0	4	0
CB1 cannabinoid receptor-interacting protein 1	CNRP1_HUMAN	19 kDa	5	3	0	0	0	0	0	0	3	0
Glutamine synthetase	GLNA_HUMAN	42 kDa	6	3	0	0	2	0	0	0	0	0
EH domain-containing protein 3	EHD3_HUMAN	61 kDa	6	2	1	0	0	0	0	0	2	0
EF-hand domain-containing protein D2	EFHD2_HUMAN	27 kDa	6	1	0	0	0	4	0	0	0	0
Myosin light chain 5	MYL5_HUMAN	20 kDa	0	0	11	0	0	0	0	0	0	0

Description	Accession	MW	Raw spectral counts									
			Frontal cortex	Cerebellum	Right ventricle	Mesentric lymph node	Liver	Pancreas	Proximal bile duct	Breast	Ovary	Clitoris
Carnitine O-palmitoyltransferase 2	CPT2_HUMAN	74 kDa	0	0	8	0	2	1	0	0	0	0
Importin-5	IPO5_HUMAN	124 kDa	1	0	1	0	0	3	0	0	6	0
Signal peptidase complex subunit 2	SPCS2_HUMAN	25 kDa	0	0	0	0	3	8	0	0	0	0
Spermidine synthase	SPEE_HUMAN	34 kDa	0	0	0	0	0	7	0	0	4	0
N-acetyl-D-glucosamine kinase	NAGK_HUMAN	37 kDa	0	0	0	0	0	6	0	0	5	0
Surfeit locus protein 4	SURF4_HUMAN	30 kDa	0	0	0	0	5	6	0	0	0	0
A-kinase anchor protein 12	AKA12_HUMAN	191 kDa	3	0	0	0	0	1	0	0	7	0
cAMP-dependent protein kinase type II-beta regulatory subunit	KAP3_HUMAN	46 kDa	6	0	0	0	0	0	0	0	5	0
SEC14-like protein 2	S14L2_HUMAN	46 kDa	0	0	0	0	11	0	0	0	0	0
Annexin A8-like protein 2	AXA82_HUMAN	37 kDa	0	0	0	0	2	0	0	0	1	9
Protein S100-A4	S10A4_HUMAN	12 kDa	0	0	2	1	0	0	1	0	0	8
Fibrinogen beta chain	FIBB_HUMAN	56 kDa	0	0	2	0	0	0	1	0	3	6
Pigment epithelium-derived factor	PEDF_HUMAN	46 kDa	0	0	0	2	0	0	3	0	5	2
Prothymosin alpha	PTMA_HUMAN	12 kDa	0	0	0	0	0	0	0	0	10	2
GTPase NRas	RASN_HUMAN	21 kDa	3	3	0	1	0	0	0	0	4	1
Hepatoma-derived growth factor-related protein 3	HDGR3_HUMAN	23 kDa	0	7	0	0	0	0	0	0	5	0
Septin-8	SEPT8_HUMAN	56 kDa	6	6	0	0	0	0	0	0	0	0
Apolipoprotein O	APOO_HUMAN	22 kDa	2	5	5	0	0	0	0	0	0	0
Succinate-semialdehyde dehydrogenase	SSDH_HUMAN	57 kDa	1	5	3	0	3	0	0	0	0	0
Hippocalcin-like protein 1	HPCL1_HUMAN	22 kDa	5	5	0	0	0	0	0	0	2	0
Glutaminase kidney isoform	GLSK_HUMAN	73 kDa	8	4	0	0	0	0	0	0	0	0
Isoleucine--tRNA ligase	SYIM_HUMAN	114 kDa	2	2	4	0	3	0	0	0	1	0
Glycine--tRNA ligase	SYG_HUMAN	83 kDa	3	1	0	0	0	8	0	0	0	0
Stathmin	STMN1_HUMAN	17 kDa	9	1	0	0	0	0	0	0	2	0
Heat shock protein 105 kDa	HS105_HUMAN	97 kDa	10	1	0	0	0	0	0	0	1	0
Acetyl-coenzyme A synthetase 2-like	ACS2L_HUMAN	75 kDa	0	0	12	0	0	0	0	0	0	0
Dermcidin	DCD_HUMAN	11 kDa	2	0	2	0	4	4	0	0	0	0
Dynactin subunit 2	DCTN2_HUMAN	44 kDa	5	0	1	0	0	0	2	0	4	0
Endoplasmic reticulum resident protein 27	ERP27_HUMAN	30 kDa	0	0	0	0	0	12	0	0	0	0
Leukocyte elastase inhibitor	ILEU_HUMAN	43 kDa	0	0	0	0	0	12	0	0	0	0
2-amino-3-ketobutyrate coenzyme A ligase	KBL_HUMAN	45 kDa	0	0	0	0	5	7	0	0	0	0
Guanine nucleotide-binding protein G(k) subunit alpha	GNAI3_HUMAN	41 kDa	0	0	0	0	0	5	0	0	7	0
Mitochondrial 10-formyltetrahydrofolate dehydrogenase	AL1L2_HUMAN	102 kDa	0	0	0	0	10	2	0	0	0	0
Peroxisomal trans-2-enoyl-CoA reductase	PECR_HUMAN	33 kDa	0	0	0	1	11	0	0	0	0	0
UDP-glucuronosyltransferase 2B7	UD2B7_HUMAN	61 kDa	0	0	0	0	12	0	0	0	0	0
Urocanate hydratase	HUTU_HUMAN	75 kDa	0	0	0	0	12	0	0	0	0	0
NAD kinase 2	NAKD2_HUMAN	49 kDa	0	0	0	0	12	0	0	0	0	0
Guanine deaminase	GUAD_HUMAN	51 kDa	12	0	0	0	0	0	0	0	0	0
Biglycan	PGS1_HUMAN	42 kDa	0	0	2	1	0	0	2	0	1	7
Olfactomedin-like protein 1	OLFL1_HUMAN	46 kDa	0	0	0	0	0	0	3	0	5	5
60S ribosomal protein L19	RL19_HUMAN	23 kDa	0	0	0	0	2	8	0	0	0	3
Erythrocyte band 7 integral membrane protein	STOM_HUMAN	32 kDa	0	0	0	2	5	0	1	0	2	3
Proteasome subunit beta type-4	PSB4_HUMAN	29 kDa	2	1	3	1	3	0	1	0	1	1
Synaptotagmin-2	SYT2_HUMAN	47 kDa	0	13	0	0	0	0	0	0	0	0
Ankyrin-1	ANK1_HUMAN	206 kDa	0	13	0	0	0	0	0	0	0	0
Ras-related protein Rab-5B	RAB5B_HUMAN	24 kDa	0	7	0	0	0	0	0	0	6	0
Calcium-dependent secretion activator 1	CAPS1_HUMAN	153 kDa	6	7	0	0	0	0	0	0	0	0
V-type proton ATPase subunit D	VATD_HUMAN	28 kDa	7	6	0	0	0	0	0	0	0	0
Succinyl-CoA ligase [ADP-forming] subunit beta	SUCB1_HUMAN	50 kDa	1	4	8	0	0	0	0	0	0	0
ATP synthase subunit f	ATPK_HUMAN	11 kDa	0	4	4	0	3	1	0	0	1	0
Astrocytic phosphoprotein PEA-15	PEA15_HUMAN	15 kDa	7	4	0	0	0	0	0	0	2	0
Mitochondrial import receptor subunit TOM70	TOM70_HUMAN	67 kDa	7	4	0	0	2	0	0	0	0	0

Description	Accession	MW	Raw spectral counts									
			Frontal cortex	Cerebellum	Right ventricle	Mesentric lymph node	Liver	Pancreas	Proximal bile duct	Breast	Ovary	Clitoris
Hippocalcin-like protein 4	HPCL4_HUMAN	22 kDa	11	2	0	0	0	0	0	0	0	0
V-type proton ATPase subunit C 1	VATC1_HUMAN	44 kDa	9	1	0	0	1	2	0	0	0	0
Myoglobin	MYG_HUMAN	17 kDa	0	0	13	0	0	0	0	0	0	0
EH domain-containing protein 1	EHD1_HUMAN	61 kDa	4	0	4	0	0	0	0	0	5	0
Ras-related protein Rab-18	RAB18_HUMAN	23 kDa	2	0	2	0	3	3	0	0	3	0
Glutathione peroxidase 1	GPX1_HUMAN	22 kDa	0	0	1	0	9	0	0	0	3	0
S-adenosylmethionine synthase isoform type-1	METK1_HUMAN	44 kDa	0	0	0	0	8	5	0	0	0	0
Enoyl-CoA hydratase domain-containing protein 3	ECHD3_HUMAN	33 kDa	0	0	0	0	7	4	0	0	2	0
5-oxoprolinase	OPLA_HUMAN	137 kDa	0	0	0	0	12	1	0	0	0	0
Lamina-associated polypeptide 2, isoforms beta/gamma	LAP2B_HUMAN	51 kDa	0	0	0	0	0	0	0	0	13	0
Glutathione S-transferase Mu 1	GSTM1_HUMAN	26 kDa	0	0	0	0	0	0	0	0	13	0
Heme-binding protein 1	HEBP1_HUMAN	21 kDa	4	0	0	0	4	0	0	0	5	0
Cytochrome P450 2A7	CP2A7_HUMAN	56 kDa	0	0	0	0	13	0	0	0	0	0
Peroxisomal acyl-coenzyme A oxidase 2	ACOX2_HUMAN	77 kDa	0	0	0	0	13	0	0	0	0	0
UDP-glucuronosyltransferase 2B4	UD2B4_HUMAN	61 kDa	0	0	0	0	13	0	0	0	0	0
UDP-glucuronosyltransferase 2B11	UDB11_HUMAN	61 kDa	0	0	0	0	13	0	0	0	0	0
Retinol dehydrogenase 16	RDH16_HUMAN	36 kDa	0	0	0	0	13	0	0	0	0	0
Bifunctional epoxide hydrolase 2	HYES_HUMAN	63 kDa	0	0	0	0	13	0	0	0	0	0
Keratin, type II cuticular Hb6	KRT86_HUMAN	53 kDa	13	0	0	0	0	0	0	0	0	0
Serine/threonine-protein phosphatase 2B catalytic subunit	PP2BB_HUMAN	59 kDa	13	0	0	0	0	0	0	0	0	0
Thymidine phosphorylase	TYPH_HUMAN	50 kDa	0	0	0	0	12	0	0	0	0	2
Very-long-chain (3R)-3-hydroxyacyl-CoA dehydratase 3	HACD3_HUMAN	43 kDa	1	4	0	0	1	4	0	0	3	1
Signal recognition particle 14 kDa protein	SRP14_HUMAN	15 kDa	2	4	0	0	1	3	0	0	3	1
Myelin-associated glycoprotein	MAG_HUMAN	69 kDa	5	9	0	0	0	0	0	0	0	0
T-complex protein 1 subunit gamma	TCPG_HUMAN	61 kDa	3	6	1	0	0	1	0	0	3	0
Gamma-soluble NSF attachment protein	SNAG_HUMAN	35 kDa	8	4	0	0	0	0	0	0	2	0
Peptidyl-prolyl cis-trans isomerase FKBP3	FKBP3_HUMAN	25 kDa	1	3	1	0	2	3	0	0	4	0
Clathrin light chain A	CLCA_HUMAN	27 kDa	5	3	0	0	2	2	0	0	2	0
LIM and SH3 domain protein 1	LASP1_HUMAN	30 kDa	6	2	0	0	0	4	0	0	2	0
Dual specificity protein phosphatase 3	DUS3_HUMAN	20 kDa	5	1	6	0	0	0	2	0	0	0
Atlastin-1	ATLA1_HUMAN	64 kDa	13	1	0	0	0	0	0	0	0	0
Stomatin-like protein 2	STML2_HUMAN	39 kDa	1	0	1	0	3	5	1	0	3	0
S-adenosylmethionine synthase isoform type-2	METK2_HUMAN	44 kDa	0	0	0	0	3	6	1	0	4	0
Choline dehydrogenase	CHDH_HUMAN	65 kDa	0	0	0	0	10	4	0	0	0	0
Acyl-coenzyme A synthetase ACSM2A	ACS2A_HUMAN	64 kDa	0	0	0	0	14	0	0	0	0	0
Hydroxyacid oxidase 1	HAOX1_HUMAN	41 kDa	0	0	0	0	14	0	0	0	0	0
Catenin alpha-2	CTNA2_HUMAN	105 kDa	14	0	0	0	0	0	0	0	0	0
Apolipoprotein B-100	APOB_HUMAN	516 kDa	0	0	0	0	4	0	1	0	0	10
SH3 domain-binding glutamic acid-rich-like protein 3	SH3L3_HUMAN	10 kDa	1	0	0	4	0	2	3	0	0	5
Haloacid dehalogenase-like hydrolase domain-containing protein 2	HDHD2_HUMAN	29 kDa	5	2	2	0	0	0	1	0	3	2
Plastin-3	PLST_HUMAN	71 kDa	0	0	0	0	11	3	0	0	0	1
Signal-regulatory protein beta-1	SIRB1_HUMAN	43 kDa	8	7	0	0	0	0	0	0	0	0
Receptor-type tyrosine-protein phosphatase zeta	PTPRZ_HUMAN	255 kDa	9	6	0	0	0	0	0	0	0	0
NADH dehydrogenase [ubiquinone] 1 alpha subcomplex subunit 13	NDUAD_HUMAN	17 kDa	4	4	5	0	0	1	0	0	1	0
Methylglutaconyl-CoA hydratase	AUHM_HUMAN	36 kDa	4	4	4	0	2	1	0	0	0	0
Adenylate kinase 4	KAD4_HUMAN	25 kDa	2	4	3	1	5	0	0	0	0	0
Neural cell adhesion molecule L1	L1CAM_HUMAN	140 kDa	10	4	0	0	0	0	1	0	0	0
Cold-inducible RNA-binding protein	CIRBP_HUMAN	19 kDa	0	3	2	0	0	4	0	0	6	0
Eukaryotic translation initiation factor 4H	IF4H_HUMAN	27 kDa	3	2	1	0	0	4	0	0	5	0
Myomesin-1	MYOM1_HUMAN	188 kDa	0	0	15	0	0	0	0	0	0	0
Protein transport protein Sec23B	SC23B_HUMAN	86 kDa	0	0	0	0	0	15	0	0	0	0
Regucalcin	RGN_HUMAN	33 kDa	1	0	0	0	6	7	0	0	1	0

Description	Accession	MW	Raw spectral counts									
			Frontal cortex	Cerebellum	Right ventricle	Mesentric lymph node	Liver	Pancreas	Proximal bile duct	Breast	Ovary	Clitoris
Phosphoserine aminotransferase	SERC_HUMAN	40 kDa	3	0	0	0	8	4	0	0	0	0
3-hydroxyanthranilate 3,4-dioxygenase	3HAO_HUMAN	33 kDa	0	0	0	0	14	0	0	0	1	0
Acyl-coenzyme A synthetase ACSM2B	ACS2B_HUMAN	64 kDa	0	0	0	0	15	0	0	0	0	0
UDP-glucuronosyltransferase 1-3	UD13_HUMAN	60 kDa	0	0	0	0	15	0	0	0	0	0
Acyl-CoA dehydrogenase family member 11	ACD11_HUMAN	87 kDa	0	0	0	0	15	0	0	0	0	0
Cytochrome P450 2D6	CP2D6_HUMAN	56 kDa	0	0	0	0	15	0	0	0	0	0
Sulfotransferase 1A2	ST1A2_HUMAN	34 kDa	0	0	0	0	15	0	0	0	0	0
Arginase-1	ARGI1_HUMAN	35 kDa	0	0	0	0	15	0	0	0	0	0
Serine/threonine-protein phosphatase 2A catalytic subunit	PP2AA_HUMAN	36 kDa	5	3	1	0	0	2	1	0	1	3
Heterochromatin protein 1-binding protein 3	HP1B3_HUMAN	61 kDa	0	4	0	0	0	2	0	0	9	1
RNA-binding motif protein, X chromosome	RBMX_HUMAN	42 kDa	0	4	0	0	2	2	0	0	7	1
60S ribosomal protein L10a	RL10A_HUMAN	25 kDa	0	0	0	0	2	8	1	0	4	1
Calretinin	CALB2_HUMAN	32 kDa	2	14	0	0	0	0	0	0	0	0
Ras-related protein Rab-6B	RAB6B_HUMAN	23 kDa	4	7	2	0	1	1	0	0	1	0
Kinesin heavy chain isoform 5C	KIF5C_HUMAN	109 kDa	9	7	0	0	0	0	0	0	0	0
Peptidyl-prolyl cis-trans isomerase FKBP2	FKBP2_HUMAN	16 kDa	0	3	1	0	5	6	1	0	0	0
Dual specificity mitogen-activated protein kinase kinase 1	MP2K1_HUMAN	43 kDa	10	3	0	0	0	2	0	0	1	0
Cytochrome b5	CYB5_HUMAN	15 kDa	2	0	2	2	6	2	0	0	2	0
Propionyl-CoA carboxylase alpha chain	PCCA_HUMAN	80 kDa	0	0	1	1	14	0	0	0	0	0
Glucagon	GLUC_HUMAN	21 kDa	0	0	0	0	0	16	0	0	0	0
Dolichyl-diphosphooligosaccharide--protein glycosyltransferase subunit STT3A	STT3A_HUMAN	81 kDa	0	0	0	0	1	15	0	0	0	0
Glycine N-methyltransferase	GNMT_HUMAN	33 kDa	0	0	0	0	14	2	0	0	0	0
LIM and cysteine-rich domains protein 1	LMCD1_HUMAN	41 kDa	0	0	0	0	0	0	0	0	16	0
Fatty acid-binding protein, liver	FABPL_HUMAN	14 kDa	0	0	0	0	16	0	0	0	0	0
Cytochrome P450 2A6	CP2A6_HUMAN	57 kDa	0	0	0	0	16	0	0	0	0	0
Aldo-keto reductase family 1 member C4	AK1C4_HUMAN	37 kDa	0	0	0	0	16	0	0	0	0	0
Cystathionine gamma-lyase	CGL_HUMAN	45 kDa	0	0	0	0	13	1	0	0	0	3
60S ribosomal protein L31	RL31_HUMAN	14 kDa	3	0	0	0	1	10	0	0	1	2
Polyadenylate-binding protein 1	PABP1_HUMAN	71 kDa	2	4	0	0	1	7	0	0	2	1
Perilipin-4	PLIN4_HUMAN	134 kDa	0	0	8	8	0	0	0	0	0	1
Adenosine kinase	ADK_HUMAN	41 kDa	2	0	0	0	8	2	0	0	4	1
Ankyrin-3	ANK3_HUMAN	480 kDa	3	14	0	0	0	0	0	0	0	0
Sodium/potassium-transporting ATPase subunit beta-2	AT1B2_HUMAN	33 kDa	5	12	0	0	0	0	0	0	0	0
Synaptophysin	SYPH_HUMAN	34 kDa	10	7	0	0	0	0	0	0	0	0
V-type proton ATPase subunit d 1	VA0D1_HUMAN	40 kDa	10	6	0	0	0	1	0	0	0	0
Mitochondrial carrier homolog 2	MTCH2_HUMAN	33 kDa	4	4	1	0	7	1	0	0	0	0
Brain acid soluble protein 1	BASP1_HUMAN	23 kDa	13	4	0	0	0	0	0	0	0	0
Calsequestrin-2	CASQ2_HUMAN	46 kDa	0	0	17	0	0	0	0	0	0	0
Dystrophin	DMD_HUMAN	427 kDa	0	0	16	0	0	0	0	0	1	0
Hydroxysteroid dehydrogenase-like protein 2	HSDL2_HUMAN	45 kDa	0	0	9	1	5	0	0	0	2	0
Keratin, type II cytoskeletal 71	K2C71_HUMAN	57 kDa	0	0	5	4	4	3	1	0	0	0
U8 snoRNA-decapping enzyme	NUD16_HUMAN	21 kDa	0	0	2	0	9	2	0	0	4	0
Protein transport protein Sec31A	SC31A_HUMAN	133 kDa	0	0	1	0	0	16	0	0	0	0
Inactive pancreatic lipase-related protein 1	LIPR1_HUMAN	52 kDa	0	0	0	0	0	17	0	0	0	0
Leucine-rich repeat-containing protein 59	LRC59_HUMAN	35 kDa	0	0	0	0	1	14	0	0	2	0
Signal recognition particle receptor subunit beta	SRPRB_HUMAN	30 kDa	0	0	0	0	2	13	0	0	2	0
Serine hydroxymethyltransferase	GLYM_HUMAN	56 kDa	0	0	0	0	14	3	0	0	0	0
Serum paraoxonase/arylesterase 1	PON1_HUMAN	40 kDa	0	0	0	0	17	0	0	0	0	0
Argininosuccinate lyase	ARLY_HUMAN	52 kDa	0	0	0	0	17	0	0	0	0	0
Ornithine carbamoyltransferase	OTC_HUMAN	40 kDa	0	0	0	0	17	0	0	0	0	0
UDP-glucuronosyltransferase 1-6	UD16_HUMAN	61 kDa	0	0	0	0	17	0	0	0	0	0
Ras-related protein Rab-10	RAB10_HUMAN	23 kDa	5	3	2	1	1	3	0	0	0	3

Description	Accession	MW	Raw spectral counts									
			Frontal cortex	Cerebellum	Right ventricle	Mesentric lymph node	Liver	Pancreas	Proximal bile duct	Breast	Ovary	Clitoris
Creatine kinase U-type	KCRU_HUMAN	47 kDa	6	7	0	0	0	2	2	0	0	1
Ubiquitin carboxyl-terminal hydrolase 5	UBP5_HUMAN	96 kDa	8	2	1	0	3	0	0	0	3	1
Phosphoglycerate mutase 2	PGAM2_HUMAN	29 kDa	0	12	6	0	0	0	0	0	0	0
Endophilin-A1	SH3G2_HUMAN	40 kDa	8	10	0	0	0	0	0	0	0	0
Synaptogyrin-1	SNG1_HUMAN	25 kDa	10	7	0	0	0	0	0	0	1	0
2-oxoglutarate dehydrogenase-like	OGDHL_HUMAN	114 kDa	9	7	0	0	2	0	0	0	0	0
Myc box-dependent-interacting protein 1	BIN1_HUMAN	65 kDa	13	5	0	0	0	0	0	0	0	0
Protein NipSnap homolog 2	NIPS2_HUMAN	34 kDa	2	3	6	1	0	1	1	0	4	0
Cytochrome c oxidase subunit 5A	COX5A_HUMAN	17 kDa	3	1	4	1	2	4	1	0	2	0
GTP-binding protein SAR1b	SAR1B_HUMAN	22 kDa	0	0	3	0	7	8	0	0	0	0
Delta-aminolevulinic acid dehydratase	HEM2_HUMAN	36 kDa	0	0	1	0	10	4	0	0	3	0
Cytoskeleton-associated protein 4	CKAP4_HUMAN	66 kDa	0	0	0	0	0	11	0	0	7	0
Peroxisomal bifunctional enzyme	ECHP_HUMAN	79 kDa	0	0	0	0	18	0	0	0	0	0
Homogentisate 1,2-dioxygenase	HGD_HUMAN	50 kDa	0	0	0	0	18	0	0	0	0	0
Core histone macro-H2A.1	H2AY_HUMAN	40 kDa	0	2	0	0	0	4	1	0	7	5
Proteasome subunit alpha type-4	PSA4_HUMAN	29 kDa	1	2	0	0	4	1	2	0	5	4
Spectrin beta chain, erythrocytic	SPTB1_HUMAN	246 kDa	0	13	3	0	0	0	0	0	0	3
3-hydroxybutyrate dehydrogenase type 2	BDH2_HUMAN	27 kDa	3	3	0	0	4	1	0	0	5	3
SH3 domain-binding glutamic acid-rich-like protein	SH3L1_HUMAN	13 kDa	2	0	0	3	0	1	6	0	4	3
Phosphatidylinositol-binding clathrin assembly protein	PICAL_HUMAN	71 kDa	6	11	0	0	0	0	0	0	0	2
Glucosidase 2 subunit beta	GLU2B_HUMAN	59 kDa	0	0	0	0	6	2	0	0	9	2
UPF0568 protein C14orf166	CN166_HUMAN	28 kDa	3	1	1	0	2	1	0	0	10	1
Calcineurin subunit B type 1	CANB1_HUMAN	19 kDa	8	11	0	0	0	0	0	0	0	0
ATP-dependent 6-phosphofructokinase, platelet type	PFKAP_HUMAN	86 kDa	9	10	0	0	0	0	0	0	0	0
Sideroflexin-1	SFXN1_HUMAN	36 kDa	2	8	0	0	8	1	0	0	0	0
Myelin-oligodendrocyte glycoprotein	MOG_HUMAN	28 kDa	11	8	0	0	0	0	0	0	0	0
Isocitrate dehydrogenase [NAD] subunit beta	IDH3B_HUMAN	42 kDa	6	6	5	0	0	0	0	0	2	0
4-aminobutyrate aminotransferase	GABT_HUMAN	56 kDa	5	6	0	0	7	1	0	0	0	0
Reticulon-1	RTN1_HUMAN	84 kDa	13	6	0	0	0	0	0	0	0	0
Stress-induced-phosphoprotein 1	STIP1_HUMAN	63 kDa	8	3	0	0	2	2	0	0	4	0
Hydroxyacylglutathione hydrolase	GLO2_HUMAN	34 kDa	4	1	2	0	7	3	1	0	1	0
Glutathione S-transferase kappa 1	GSTK1_HUMAN	25 kDa	0	0	6	1	5	3	0	0	4	0
Glutathione S-transferase Mu 4	GSTM4_HUMAN	26 kDa	0	0	2	0	0	3	2	0	12	0
Chymotrypsin-like elastase family member 2B	CEL2B_HUMAN	29 kDa	0	0	0	0	0	19	0	0	0	0
Ethylmalonyl-CoA decarboxylase	ECHD1_HUMAN	34 kDa	0	0	0	8	9	0	2	0	0	0
Peroxisomal acyl-coenzyme A oxidase 1	ACOX1_HUMAN	74 kDa	0	0	0	0	19	0	0	0	0	0
Hydroxymethylglutaryl-CoA synthase	HMCS2_HUMAN	57 kDa	0	0	0	0	19	0	0	0	0	0
Ras-related protein Rab-1B	RAB1B_HUMAN	22 kDa	12	0	0	0	7	0	0	0	0	0
Ketimine reductase mu-crystallin	CRYM_HUMAN	34 kDa	19	0	0	0	0	0	0	0	0	0
Cadherin-13	CAD13_HUMAN	78 kDa	0	0	12	0	0	0	0	0	4	4
Ras-related protein Rab-35	RAB35_HUMAN	23 kDa	6	6	0	0	0	0	0	0	5	3
Alpha-1-acid glycoprotein 2	A1AG2_HUMAN	24 kDa	0	0	4	4	2	0	5	0	3	2
Myristoylated alanine-rich C-kinase substrate	MARCS_HUMAN	32 kDa	6	13	0	0	0	1	0	0	0	0
Beta-adducin	ADDB_HUMAN	81 kDa	11	9	0	0	0	0	0	0	0	0
Protein kinase C and casein kinase substrate in neurons protein 1	PACN1_HUMAN	51 kDa	13	7	0	0	0	0	0	0	0	0
Ras-related protein Rab-3D	RAB3D_HUMAN	24 kDa	9	5	0	0	0	6	0	0	0	0
Membrane-associated progesterone receptor component 1	PGRC1_HUMAN	22 kDa	4	2	0	0	9	0	0	0	5	0
Delta(3,5)-Delta(2,4)-dienoyl-CoA isomerase	ECH1_HUMAN	36 kDa	1	0	10	0	7	1	0	0	1	0
Microsomal triglyceride transfer protein large subunit	MTP_HUMAN	99 kDa	0	0	0	0	20	0	0	0	0	0
Ubiquitin carboxyl-terminal hydrolase isozyme L1	UCHL1_HUMAN	25 kDa	8	6	0	0	0	0	3	0	2	2
Obg-like ATPase 1	OLA1_HUMAN	45 kDa	3	2	2	0	2	7	0	0	4	1
3-oxo-5-beta-steroid 4-dehydrogenase	AK1D1_HUMAN	37 kDa	0	0	0	0	20	0	0	0	0	1

Description	Accession	MW	Raw spectral counts									
			Frontal cortex	Cerebellum	Right ventricle	Mesentric lymph node	Liver	Pancreas	Proximal bile duct	Breast	Ovary	Clitoris
Ras-related protein Rab-3A	RAB3A_HUMAN	25 kDa	11	10	0	0	0	0	0	0	0	0
Amphiphysin	AMPH_HUMAN	76 kDa	14	7	0	0	0	0	0	0	0	0
Calcium/calmodulin-dependent protein kinase type II subunit gamma	KCC2G_HUMAN	63 kDa	14	7	0	0	0	0	0	0	0	0
N(G),N(G)-dimethylarginine dimethylaminohydrolase 1	DDAH1_HUMAN	31 kDa	11	6	0	0	3	1	0	0	0	0
Glycerol-3-phosphate dehydrogenase	GPDM_HUMAN	81 kDa	9	5	2	0	0	3	0	0	2	0
Dihydrolipoyllysine-residue acetyltransferase component of pyruvate dehydrogenase	ODP2_HUMAN	69 kDa	6	3	7	0	1	3	0	0	1	0
NAD-dependent malic enzyme	MAOM_HUMAN	65 kDa	0	2	19	0	0	0	0	0	0	0
Propionyl-CoA carboxylase beta chain	PCCB_HUMAN	58 kDa	0	0	8	0	13	0	0	0	0	0
Annexin A13	ANX13_HUMAN	35 kDa	0	0	0	0	0	20	1	0	0	0
Protein transport protein Sec61 subunit 1	S61A1_HUMAN	52 kDa	0	0	0	0	1	20	0	0	0	0
Carboxymethylenebutenolidase homolog	CMBL_HUMAN	28 kDa	0	0	0	0	17	3	0	0	1	0
Periaxin	PRAX_HUMAN	155 kDa	0	0	0	0	0	0	0	0	0	22
Prolow-density lipoprotein receptor-related protein 1	LRP1_HUMAN	505 kDa	0	0	0	1	2	0	1	0	13	5
ES1 protein homolog	ES1_HUMAN	28 kDa	2	4	5	1	4	1	0	0	1	4
Septin-11	SEP11_HUMAN	49 kDa	13	4	0	0	0	0	0	0	3	2
L-xylulose reductase	DCXR_HUMAN	26 kDa	0	1	0	0	15	0	0	0	5	1
Redox-regulatory protein FAM213A	F213A_HUMAN	26 kDa	3	0	1	10	5	2	0	0	0	1
Excitatory amino acid transporter 4	EAA4_HUMAN	62 kDa	0	22	0	0	0	0	0	0	0	0
Troponin C, slow skeletal and cardiac muscles	TNNC1_HUMAN	18 kDa	0	0	22	0	0	0	0	0	0	0
Cytochrome P450 3A7	CP3A7_HUMAN	58 kDa	0	0	0	0	22	0	0	0	0	0
3-ketoacyl-CoA thiolase, peroxisomal	THIK_HUMAN	44 kDa	0	0	0	0	22	0	0	0	0	0
Sorcin	SORCN_HUMAN	22 kDa	3	2	1	0	2	4	0	0	8	3
Solute carrier family 12 member 5	S12A5_HUMAN	126 kDa	5	18	0	0	0	0	0	0	0	0
Mycophenolic acid acyl-glucuronide esterase	ABHDA_HUMAN	34 kDa	4	3	6	0	8	0	0	0	2	0
60S ribosomal protein L10	RL10_HUMAN	25 kDa	1	3	1	0	2	13	0	0	3	0
Dehydrogenase/reductase SDR family member 4	DHRS4_HUMAN	30 kDa	0	0	2	1	17	1	0	0	2	0
Epiplakin	EPIPL_HUMAN	556 kDa	0	0	0	0	6	0	0	0	0	18
Beta-enolase	ENOB_HUMAN	47 kDa	0	0	15	0	0	0	0	0	0	9
4-trimethylaminobutyraldehyde dehydrogenase	AL9A1_HUMAN	54 kDa	2	0	2	1	9	1	0	0	5	4
Glycogen debranching enzyme	GDE_HUMAN	175 kDa	1	3	12	0	7	0	0	0	0	1
Versican core protein	CSPG2_HUMAN	373 kDa	11	11	0	0	0	0	0	0	2	0
Tubulin polymerization-promoting protein	TPPP_HUMAN	24 kDa	14	10	0	0	0	0	0	0	0	0
NADH dehydrogenase [ubiquinone] 1 alpha subcomplex subunit 9	NDUA9_HUMAN	43 kDa	4	4	8	1	3	4	0	0	0	0
Myomesin-2	MYOM2_HUMAN	165 kDa	0	0	24	0	0	0	0	0	0	0
Cytosolic 10-formyltetrahydrofolate dehydrogenase	AL1L1_HUMAN	99 kDa	0	0	0	0	24	0	0	0	0	0
17-beta-hydroxysteroid dehydrogenase type 6	H17B6_HUMAN	36 kDa	0	0	0	0	24	0	0	0	0	0
BTB/POZ domain-containing protein KCTD12	KCD12_HUMAN	36 kDa	0	4	0	0	0	0	4	0	11	6
Adipocyte plasma membrane-associated protein	APMAP_HUMAN	46 kDa	2	3	0	6	5	0	1	0	6	2
Protein kinase C delta-binding protein	PRDBP_HUMAN	28 kDa	0	0	3	8	0	0	5	0	7	2
Glycogen phosphorylase, liver form	PYGL_HUMAN	97 kDa	0	0	0	0	24	0	0	0	0	1
Calbindin	CALB1_HUMAN	30 kDa	4	21	0	0	0	0	0	0	0	0
Guanine nucleotide-binding protein G(i) subunit alpha-1	GNAI1_HUMAN	40 kDa	17	8	0	0	0	0	0	0	0	0
Dynamin-like 120 kDa protein	OPA1_HUMAN	112 kDa	8	7	10	0	0	0	0	0	0	0
Isovaleryl-CoA dehydrogenase	IVD_HUMAN	46 kDa	0	0	5	0	13	7	0	0	0	0
Cytochrome P450 3A4	CP3A4_HUMAN	57 kDa	0	0	0	0	25	0	0	0	0	0
Phosphoglucomutase-like protein 5	PGM5_HUMAN	62 kDa	0	0	11	2	0	0	9	0	2	2
Collagen alpha-1(XII) chain	COCA1_HUMAN	333 kDa	0	0	0	0	1	0	0	0	24	1
Neuronal membrane glycoprotein M6-a	GPM6A_HUMAN	31 kDa	13	13	0	0	0	0	0	0	0	0
Neurochondrin	NCDN_HUMAN	79 kDa	18	8	0	0	0	0	0	0	0	0
D-beta-hydroxybutyrate dehydrogenase	BDH_HUMAN	38 kDa	2	1	6	1	14	0	2	0	0	0
Troponin T, cardiac muscle	TNNT2_HUMAN	36 kDa	0	0	26	0	0	0	0	0	0	0
Glutathione S-transferase A3	GSTA3_HUMAN	25 kDa	0	0	0	0	21	2	0	0	3	0

Description	Accession	MW	Raw spectral counts									
			Frontal cortex	Cerebellum	Right ventricle	Mesentric lymph node	Liver	Pancreas	Proximal bile duct	Breast	Ovary	Clitoris
Serine hydroxymethyltransferase, cytosolic	GLYC_HUMAN	53 kDa	0	0	0	0	26	0	0	0	0	0
Arylacetamide deacetylase	AAAD_HUMAN	46 kDa	0	0	0	0	26	0	0	0	0	0
Succinate dehydrogenase [ubiquinone] iron-sulfur subunit	SDHB_HUMAN	32 kDa	3	2	8	0	4	4	1	0	3	2
Calcium/calmodulin-dependent protein kinase type II subunit delta	KCC2D_HUMAN	56 kDa	16	8	3	0	0	0	0	0	0	0
Alpha-synuclein	SYUA_HUMAN	14 kDa	18	8	1	0	0	0	0	0	0	0
NADH dehydrogenase [ubiquinone] flavoprotein 1	NDUV1_HUMAN	51 kDa	5	6	11	1	1	1	2	0	0	0
Vesicle-associated membrane protein-associated protein B/C	VAPB_HUMAN	27 kDa	5	5	3	0	5	3	0	0	6	0
Ribonuclease UK114	UK114_HUMAN	14 kDa	4	2	0	0	12	6	1	0	2	0
Alanine--tRNA ligase, cytoplasmic	SYAC_HUMAN	107 kDa	9	1	0	0	6	9	0	0	2	0
Chymotrypsin-like elastase family member 3B	CEL3B_HUMAN	29 kDa	0	0	0	0	0	27	0	0	0	0
Microsomal glutathione S-transferase 3	MGST3_HUMAN	17 kDa	5	4	9	5	0	0	5	0	0	0
Protein-L-isoaspartate(D-aspartate) O-methyltransferase	PIMT_HUMAN	25 kDa	13	4	3	0	0	3	2	0	3	0
Sorbitol dehydrogenase	DHSO_HUMAN	38 kDa	0	1	0	0	21	6	0	0	0	0
Sarcosine dehydrogenase	SARDH_HUMAN	101 kDa	0	0	0	0	25	3	0	0	0	0
1-phosphatidylinositol 4,5-bisphosphate phosphodiesterase beta-1	PLCB1_HUMAN	139 kDa	27	0	0	0	0	1	0	0	0	0
Major vault protein	MVP_HUMAN	99 kDa	0	0	0	0	2	5	1	0	15	6
Cytoplasmic FMR1-interacting protein 2	CYFP2_HUMAN	148 kDa	18	5	0	0	0	0	0	0	4	2
V-type proton ATPase subunit E 1	VATE1_HUMAN	26 kDa	13	12	0	0	0	1	0	0	2	1
Immunoglobulin superfamily member 8	IGSF8_HUMAN	65 kDa	10	19	0	0	0	0	0	0	0	0
Beta-synuclein	SYUB_HUMAN	14 kDa	18	11	0	0	0	0	0	0	0	0
Dynamin-1-like protein	DNM1L_HUMAN	82 kDa	18	8	3	0	0	0	0	0	0	0
Dynactin subunit 1	DCTN1_HUMAN	142 kDa	14	6	1	0	2	4	0	0	2	0
Chymotrypsin-like elastase family member 2A	CEL2A_HUMAN	29 kDa	0	0	0	0	0	29	0	0	0	0
Radixin	RADI_HUMAN	69 kDa	0	8	3	0	3	0	0	0	8	8
AP-2 complex subunit alpha-1	AP2A1_HUMAN	108 kDa	17	11	0	0	0	0	0	0	0	2
Band 4.1-like protein 3	E41L3_HUMAN	121 kDa	11	19	0	0	0	0	0	0	0	0
Synaptic vesicle glycoprotein 2A	SV2A_HUMAN	83 kDa	19	11	0	0	0	0	0	0	0	0
Microtubule-associated protein 1A	MAP1A_HUMAN	305 kDa	20	10	0	0	0	0	0	0	0	0
Hypoxanthine-guanine phosphoribosyltransferase	HPRT_HUMAN	25 kDa	11	8	0	0	4	3	0	0	4	0
Troponin I, cardiac muscle	TNNI3_HUMAN	24 kDa	0	0	30	0	0	0	0	0	0	0
Methylmalonyl-CoA mutase	MUTA_HUMAN	83 kDa	0	0	3	0	15	12	0	0	0	0
Serpin I2	SPI2_HUMAN	46 kDa	0	0	0	0	0	28	0	0	2	0
Amiloride-sensitive amine oxidase [copper-containing]	AOC1_HUMAN	85 kDa	0	0	0	0	30	0	0	0	0	0
Transgelin-3	TAGL3_HUMAN	22 kDa	14	17	0	0	0	0	0	0	0	0
Calcium/calmodulin-dependent protein kinase type II subunit beta	KCC2B_HUMAN	73 kDa	20	11	0	0	0	0	0	0	0	0
Short-chain specific acyl-CoA dehydrogenase	ACADS_HUMAN	44 kDa	0	0	10	2	13	6	0	0	0	0
Heat shock-related 70 kDa protein 2	HSP72_HUMAN	70 kDa	0	0	0	0	0	0	0	0	15	17
Alpha-crystallin B chain	CRYAB_HUMAN	20 kDa	6	7	8	5	0	0	4	0	0	2
Heterogeneous nuclear ribonucleoprotein Q	HNRPQ_HUMAN	70 kDa	4	5	0	0	3	11	0	0	7	2
Alpha-adducin	ADDA_HUMAN	81 kDa	13	17	0	0	0	0	0	0	1	1
V-type proton ATPase 116 kDa subunit a isoform 1	VPP1_HUMAN	96 kDa	19	13	0	0	0	0	0	0	0	0
Mitochondrial amidoxime reducing component 2	MARC2_HUMAN	38 kDa	3	0	4	2	11	7	0	0	5	0
Protein disulfide-isomerase A2	PDIA2_HUMAN	58 kDa	0	0	0	0	0	32	0	0	0	0
Aldo-keto reductase family 1 member C3	AK1C3_HUMAN	37 kDa	0	0	0	0	21	11	0	0	0	0
Reticulon-4	RTN4_HUMAN	130 kDa	9	5	0	4	6	0	1	0	5	3
Heat shock protein beta-6	HSPB6_HUMAN	17 kDa	0	0	5	11	0	0	9	0	8	0
4-hydroxyphenylpyruvate dioxygenase	HPPD_HUMAN	45 kDa	0	0	0	0	33	0	0	0	0	0
Poly(rC)-binding protein 2	PCBP2_HUMAN	39 kDa	1	5	0	0	0	12	1	0	12	3
Aldo-keto reductase family 1 member B10	AK1BA_HUMAN	36 kDa	0	0	5	0	1	0	19	0	7	2
Neurofascin	NFASC_HUMAN	150 kDa	21	13	0	0	0	0	0	0	0	0
Cofilin-2	COF2_HUMAN	19 kDa	4	9	6	0	6	0	0	0	9	0
Aldehyde dehydrogenase X	AL1B1_HUMAN	57 kDa	0	0	0	1	28	4	1	0	0	0

Description	Accession	MW	Raw spectral counts									
			Frontal cortex	Cerebellum	Right ventricle	Mesentric lymph node	Liver	Pancreas	Proximal bile duct	Breast	Ovary	Clitoris
Phosphoenolpyruvate carboxykinase [GTP]	PCKGM_HUMAN	71 kDa	0	0	0	0	33	1	0	0	0	0
Serpin H1	SERPH_HUMAN	46 kDa	0	0	0	11	0	0	1	0	21	2
Delta-1-pyrroline-5-carboxylate dehydrogenase	AL4A1_HUMAN	62 kDa	0	0	4	0	25	6	0	0	0	0
Cytochrome P450 2A13	CP2AD_HUMAN	57 kDa	0	0	0	0	36	0	0	0	0	0
Desmoplakin	DESP_HUMAN	332 kDa	4	0	0	0	2	7	0	0	0	25
Myosin light chain 3	MYL3_HUMAN	22 kDa	0	0	38	0	0	0	0	0	0	0
ATP-citrate synthase	ACLY_HUMAN	121 kDa	0	6	0	16	0	10	2	0	1	4
Dimethylglycine dehydrogenase	M2GD_HUMAN	97 kDa	0	0	0	0	39	0	0	0	0	0
Myosin-14	MYH14_HUMAN	228 kDa	0	0	2	0	13	11	0	0	0	14
Heat shock 70 kDa protein 12A	HS12A_HUMAN	75 kDa	17	12	0	2	0	0	0	0	7	2
Calcium/calmodulin-dependent protein kinase type II subunit alpha	KCC2A_HUMAN	54 kDa	34	6	0	0	0	0	0	0	0	0
Sarcalumenin	SRCA_HUMAN	101 kDa	0	0	40	0	0	0	0	0	0	0
Beta-soluble NSF attachment protein	SNAB_HUMAN	34 kDa	12	29	0	0	0	0	0	0	0	0
Potassium-transporting ATPase alpha chain 2	AT12A_HUMAN	116 kDa	17	24	0	0	0	0	0	0	0	0
Clathrin coat assembly protein AP180	AP180_HUMAN	93 kDa	23	18	0	0	0	0	0	0	0	0
Tenascin-X	TENX_HUMAN	464 kDa	0	0	3	2	0	0	0	0	4	34
Serine/threonine-protein phosphatase 2B catalytic subunit	PP2BA_HUMAN	59 kDa	24	19	0	0	0	0	0	0	0	0
Creatine kinase S-type	KCRS_HUMAN	48 kDa	0	0	43	0	0	0	0	0	0	0
Potassium-transporting ATPase alpha chain 1	ATP4A_HUMAN	114 kDa	0	44	0	0	0	0	0	0	0	0
Sodium/potassium-transporting ATPase subunit beta-1	AT1B1_HUMAN	35 kDa	15	25	3	0	0	0	0	0	2	0
Medium-chain specific acyl-CoA dehydrogenase	ACADM_HUMAN	47 kDa	0	0	18	6	10	5	6	0	0	0
Glycine amidinotransferase	GATM_HUMAN	48 kDa	0	0	0	0	11	30	0	0	4	0
Inositol 1,4,5-trisphosphate receptor type 1	ITPR1_HUMAN	314 kDa	0	46	0	0	0	0	0	0	0	0
Putative tubulin-like protein alpha-4B	TBA4B_HUMAN	28 kDa	0	46	0	0	0	0	0	0	0	0
Dihydropyrimidinase-related protein 1	DPYL1_HUMAN	62 kDa	23	23	0	0	0	0	0	0	0	0
Tenascin-R	TENR_HUMAN	150 kDa	31	15	0	0	0	0	0	0	0	0
Short/branched chain specific acyl-CoA dehydrogenase	ACDSB_HUMAN	47 kDa	0	0	3	0	40	3	0	0	0	0
Dynamin-3	DYN3_HUMAN	98 kDa	23	24	0	0	0	0	0	0	0	0
Carboxypeptidase B	CBPB1_HUMAN	47 kDa	0	0	0	0	0	47	0	0	0	0
Cytochrome c	CYC_HUMAN	12 kDa	5	9	10	5	6	4	4	0	3	2
Trypsin-2	TRY2_HUMAN	26 kDa	0	0	0	0	0	48	0	0	0	0
Aldehyde oxidase	AOXA_HUMAN	148 kDa	1	0	0	0	47	1	0	0	0	0
Betaine--homocysteine S-methyltransferase 1	BHMT1_HUMAN	45 kDa	0	0	0	0	49	0	0	0	0	0
Liver carboxylesterase 1	EST1_HUMAN	63 kDa	0	0	0	0	49	0	0	0	0	0
Carbonyl reductase [NADPH] 1	CBR1_HUMAN	30 kDa	7	9	4	0	12	8	2	0	4	4
Vesicle-associated membrane protein 2	VAMP2_HUMAN	13 kDa	30	10	1	0	0	5	0	0	1	3
Microtubule-associated protein tau	TAU_HUMAN	79 kDa	30	20	0	0	0	0	0	0	0	0
LIM domain-binding protein 3	LDB3_HUMAN	77 kDa	0	0	50	0	0	0	0	0	0	0
Neurofilament heavy polypeptide	NFH_HUMAN	112 kDa	9	39	0	0	0	0	0	0	0	3
Pancreatic triacylglycerol lipase	LIPP_HUMAN	51 kDa	0	0	0	0	0	51	0	0	0	0
Myosin-13	MYH13_HUMAN	224 kDa	0	0	52	0	0	0	0	0	0	0
Myosin regulatory light chain 2, ventricular/cardiac muscle isoform	MLRV_HUMAN	19 kDa	0	0	51	1	0	0	0	0	0	0
Pyruvate carboxylase	PYC_HUMAN	130 kDa	0	3	0	17	32	1	0	0	0	0
Bile salt-activated lipase	CEL_HUMAN	79 kDa	0	0	0	0	0	54	0	0	0	0
Fructose-bisphosphate aldolase B	ALDOB_HUMAN	39 kDa	0	0	0	0	41	13	0	0	0	0
Calcium-binding mitochondrial carrier protein Aralar2	CMC2_HUMAN	74 kDa	0	0	9	0	45	1	0	0	0	0
Plasma membrane calcium-transporting ATPase 3	AT2B3_HUMAN	134 kDa	22	35	0	0	0	0	0	0	0	0
ATP-dependent 6-phosphofructokinase, muscle type	PFKAM_HUMAN	85 kDa	4	18	34	0	0	0	0	0	1	0
Syntaxin-1A	STX1A_HUMAN	33 kDa	45	12	0	0	0	0	0	0	0	0
2',3'-cyclic-nucleotide 3'-phosphodiesterase	CN37_HUMAN	48 kDa	25	34	0	0	0	0	0	0	0	0
Synaptotagmin-1	SYT1_HUMAN	48 kDa	27	32	0	0	0	0	0	0	0	0
Amine oxidase [flavin-containing] A	AOFA_HUMAN	60 kDa	0	4	8	8	21	11	0	0	8	0

Description	Accession	MW	Raw spectral counts									
			Frontal cortex	Cerebellum	Right ventricle	Mesentric lymph node	Liver	Pancreas	Proximal bile duct	Breast	Ovary	Clitoris
C-1-tetrahydrofolate synthase, cytoplasmic	C1TC_HUMAN	102 kDa	1	1	0	0	51	4	0	0	2	2
Visinin-like protein 1	VISL1_HUMAN	22 kDa	24	37	0	0	0	0	0	0	0	0
Apoptosis-inducing factor 1	AIFM1_HUMAN	67 kDa	0	4	29	0	21	6	0	0	1	0
Amine oxidase [flavin-containing] B	AOFB_HUMAN	59 kDa	5	7	1	4	24	10	2	0	9	0
Epoxide hydrolase 1	HYEP_HUMAN	53 kDa	0	0	0	6	39	9	5	0	1	4
Myelin proteolipid protein	MYPR_HUMAN	30 kDa	27	36	0	0	0	0	0	0	0	1
AP-2 complex subunit alpha-2	AP2A2_HUMAN	104 kDa	35	20	0	0	5	1	0	0	2	1
Glutathione S-transferase A1	GSTA1_HUMAN	26 kDa	0	0	0	0	50	3	4	0	4	4
Plasma membrane calcium-transporting ATPase 4	AT2B4_HUMAN	138 kDa	31	34	0	0	0	0	0	0	0	0
Ankyrin-2	ANK2_HUMAN	434 kDa	29	33	3	0	0	0	0	0	0	0
V-type proton ATPase catalytic subunit A	VATA_HUMAN	68 kDa	35	29	0	0	1	3	0	0	0	0
Synaptosomal-associated protein 25	SNP25_HUMAN	23 kDa	48	20	0	0	0	0	0	0	0	0
Neural cell adhesion molecule 1	NCAM1_HUMAN	95 kDa	38	29	0	0	0	0	2	0	0	0
Creatine kinase M-type	KCRM_HUMAN	43 kDa	1	1	67	0	0	0	0	0	0	0
Contactin-1	CNTN1_HUMAN	113 kDa	38	26	3	0	0	2	1	0	0	0
Myosin-4	MYH4_HUMAN	223 kDa	0	0	70	0	0	0	0	0	0	0
Excitatory amino acid transporter 2	EAA2_HUMAN	62 kDa	61	10	0	0	0	0	0	0	0	0
Myosin-3	MYH3_HUMAN	224 kDa	0	0	71	0	0	0	0	0	0	0
Myosin regulatory light chain 12B	ML12B_HUMAN	20 kDa	3	0	4	0	11	7	29	0	15	4
Excitatory amino acid transporter 1	EAA1_HUMAN	60 kDa	27	46	0	0	0	0	0	0	0	0
Aspartate aminotransferase, cytoplasmic	AATC_HUMAN	46 kDa	9	25	15	2	15	3	2	0	1	2
Synapsin-2	SYN2_HUMAN	63 kDa	46	28	0	0	0	0	0	0	0	0
Peripherin	PERI_HUMAN	54 kDa	0	0	0	0	0	0	49	0	0	30
Alpha-amylase 1	AMY1_HUMAN	58 kDa	0	0	0	0	0	79	0	0	0	0
Plasma membrane calcium-transporting ATPase 2	AT2B2_HUMAN	137 kDa	28	53	0	0	0	0	0	0	0	0
Microtubule-associated protein 2	MTAP2_HUMAN	200 kDa	77	4	0	0	0	0	0	0	0	0
Rab GDP dissociation inhibitor alpha	GDIA_HUMAN	51 kDa	32	28	0	0	0	0	6	0	9	7
Carboxypeptidase A1	CBPA1_HUMAN	47 kDa	0	0	0	0	0	83	0	0	0	0
Heat shock 70 kDa protein 6	HSP76_HUMAN	71 kDa	0	20	6	0	0	16	9	0	17	18
Lamin-B2	LMNB2_HUMAN	68 kDa	3	29	0	0	0	4	0	0	46	7
Plasma membrane calcium-transporting ATPase 1	AT2B1_HUMAN	139 kDa	44	42	1	0	0	0	0	0	1	1
Alpha-internexin	AINX_HUMAN	55 kDa	28	63	0	0	0	0	0	0	0	0
Spectrin beta chain, non-erythrocytic 2	SPTN2_HUMAN	271 kDa	21	67	0	0	6	0	0	0	0	0
Myosin-1	MYH1_HUMAN	223 kDa	0	0	94	0	0	0	0	0	0	0
Alcohol dehydrogenase 4	ADH4_HUMAN	40 kDa	0	0	0	0	95	0	0	0	0	0
Neurofilament light polypeptide	NFL_HUMAN	62 kDa	15	76	0	0	0	0	0	0	0	5
Microtubule-associated protein 1B	MAP1B_HUMAN	271 kDa	61	32	0	0	0	0	3	0	1	1
Neurofilament medium polypeptide	NFM_HUMAN	102 kDa	17	73	0	0	0	0	0	0	0	10
Guanine nucleotide-binding protein G(o) subunit alpha	GNAO_HUMAN	40 kDa	43	42	2	3	0	1	3	0	4	2
Aldo-keto reductase family 1 member C1	AK1C1_HUMAN	37 kDa	6	2	5	16	28	13	20	0	5	8
Pancreatic alpha-amylase	AMYP_HUMAN	58 kDa	0	0	0	0	0	105	0	0	0	0
Gamma-enolase	ENOG_HUMAN	47 kDa	29	69	0	0	0	0	0	0	0	8
Synapsin-1	SYN1_HUMAN	74 kDa	55	51	0	0	0	0	0	0	0	0
Histone H2A.Z	H2AZ_HUMAN	14 kDa	0	14	0	0	0	12	22	0	45	16
Tubulin alpha-8 chain	TBA8_HUMAN	50 kDa	0	110	0	0	0	0	0	0	0	0
Alpha-actinin-2	ACTN2_HUMAN	104 kDa	16	0	97	0	0	0	0	0	0	0
Syntaxin-1B	STX1B_HUMAN	33 kDa	60	58	0	0	0	0	0	0	0	0
Histone H2AX	H2AX_HUMAN	15 kDa	11	14	5	0	8	15	23	0	36	17
Myosin-binding protein C, cardiac-type	MYPC3_HUMAN	141 kDa	0	0	131	0	0	0	0	0	0	0
Alcohol dehydrogenase 1C	ADH1G_HUMAN	40 kDa	0	0	0	12	126	0	0	0	0	0
Hemoglobin subunit gamma-1	HBG1_HUMAN	16 kDa	0	0	0	122	17	0	0	0	0	0
Terminal uridylyltransferase 4	TUT4_HUMAN	185 kDa	10	14	8	31	11	9	31	0	17	9

Description	Accession	MW	Raw spectral counts									
			Frontal cortex	Cerebellum	Right ventricle	Mesentric lymph node	Liver	Pancreas	Proximal bile duct	Breast	Ovary	Clitoris
Sarcoplasmic/endoplasmic reticulum calcium ATPase 2	AT2A2_HUMAN	115 kDa	15	12	107	0	1	9	0	0	0	0
Glial fibrillary acidic protein	GFAP_HUMAN	50 kDa	37	111	0	0	0	0	0	0	0	0
Myosin-10	MYH10_HUMAN	229 kDa	15	11	0	0	24	15	0	0	56	28
Tropomyosin beta chain	TPM2_HUMAN	33 kDa	0	0	27	33	0	0	52	0	41	0
Histone H2B type 1-C/E/F/G/I	H2B1C_HUMAN	14 kDa	0	20	0	0	9	19	51	0	47	34
Tubulin beta-8 chain	TBB8_HUMAN	50 kDa	62	107	0	0	0	0	0	0	24	0
Desmin	DESM_HUMAN	54 kDa	0	0	17	23	0	0	128	0	33	0
Tubulin beta-6 chain	TBB6_HUMAN	50 kDa	85	134	0	0	0	0	0	0	0	0
Syntaxin-binding protein 1	STXB1_HUMAN	68 kDa	75	147	0	0	0	0	0	0	0	0
Alcohol dehydrogenase 1B	ADH1B_HUMAN	40 kDa	0	0	0	16	200	3	2	0	0	2
Creatine kinase B-type	KCRB_HUMAN	43 kDa	53	130	10	4	2	0	16	0	1	10
Sodium/potassium-transporting ATPase subunit alpha-2	AT1A2_HUMAN	112 kDa	117	140	23	0	0	0	0	0	0	0
Sodium/potassium-transporting ATPase subunit alpha-3	AT1A3_HUMAN	112 kDa	158	174	40	0	0	0	0	0	6	11
Myosin-6	MYH6_HUMAN	224 kDa	0	0	424	0	0	0	0	0	0	0
Tubulin beta-3 chain	TBB3_HUMAN	50 kDa	145	175	0	0	0	0	118	0	0	0
Carbamoyl-phosphate synthase [ammonia]	CPSM_HUMAN	165 kDa	0	0	0	0	477	0	0	0	0	0
Tubulin beta-2A chain	TBB2A_HUMAN	50 kDa	163	204	0	0	0	0	133	0	0	0
Myosin-7	MYH7_HUMAN	223 kDa	1	0	552	0	0	1	0	0	0	0
Actin, alpha skeletal muscle	ACTS_HUMAN	42 kDa	0	0	95	261	0	0	349	0	143	0
Actin, aortic smooth muscle	ACTA_HUMAN	42 kDa	0	0	0	268	0	0	382	0	138	138
